# Development of a rapid profiling method for the analysis of polar analytes in urine using HILIC–MS and ion mobility enabled HILIC–MS

**DOI:** 10.1007/s11306-019-1474-9

**Published:** 2019-01-22

**Authors:** Adam M. King, Lauren G. Mullin, Ian D. Wilson, Muireann Coen, Paul D. Rainville, Robert S. Plumb, Lee A. Gethings, Garth Maker, Robert Trengove

**Affiliations:** 1 Waters Corporation, SK9 4AX Wilmslow, Cheshire UK; 2 Separations Science and Metabolomics laboratory, Murdoch University, South Street, 6150 Murdoch, WA Australia; 3 Waters Corporation, 01757 Milford, MA USA; 4 Computational and Systems Medicine, Department of Surgery and Cancer, Faculty of Medicine, Imperial College London, London, UK; 5 Discovery Safety, Drug Safety and Metabolism, IMED Biotech Unit, AstraZeneca, 1 Francis Crick Avenue, CB2 0RE Cambridge, UK; 6 Medical and Molecular Sciences, School of Veterinary and Life Sciences, Murdoch University, South Street, 6150 Murdoch, WA Australia

**Keywords:** Hydrophilic interaction chromatography, Metabolic phenotyping, IMS, LC–MS/MS

## Abstract

**Introduction:**

As large scale metabolic phenotyping is increasingly employed in preclinical studies and in the investigation of human health and disease the current LC–MS/MS profiling methodologies adopted for large sample sets can result in lengthy analysis times, putting strain on available resources. As a result of these pressures rapid methods of untargeted analysis may have value where large numbers of samples require screening.

**Objectives:**

To develop, characterise and evaluate a rapid UHP-HILIC-MS-based method for the analysis of polar metabolites in rat urine and then extend the capabilities of this approach by the addition of IMS to the system.

**Methods:**

A rapid untargeted HILIC LC–MS/MS profiling method for the analysis of small polar molecules has been developed. The 3.3 min separation used a Waters BEH amide (1 mm ID) analytical column on a Waters Synapt G2-Si Q-Tof enabled with ion mobility spectrometry (IMS). The methodology, was applied to the metabolic profiling of a series of rodent urine samples from vehicle-treated control rats and animals administered tienilic acid. The same separation was subsequently linked to IMS and MS to evaluate the benefits that IMS might provide for metabolome characterisation.

**Results:**

The rapid HILIC–MS method was successfully applied to rapid analysis of rat urine and found, based on the data generated from the data acquired for the pooled quality control samples analysed at regular intervals throughout the analysis, to be robust. Peak area and retention times for the compounds detected in these samples showed good reproducibility across the batch. When used to profile the urine samples obtained from vehicle-dosed control and those administered tienilic acid the HILIC-MS method detected 3007 mass/retention time features. Analysis of the same samples using HILIC–IMS–MS enabled the detection of 6711 features. Provisional metabolite identification for a number of compounds was performed using the high collision energy MS/MS information compared against the Metlin MS/MS database and, in addition, both calculated and measured CCS values from an experimentally derived CCS database.

**Conclusion:**

A rapid metabolic profiling method for the analysis of polar metabolites has been developed. The method has the advantages of speed and both reducing sample and solvent consumption compared to conventional profiling methods. The addition of IMS added an additional dimension for feature detection and the identification of metabolites.

**Electronic supplementary material:**

The online version of this article (10.1007/s11306-019-1474-9) contains supplementary material, which is available to authorized users.

## Introduction

The application of untargeted metabolic phenotyping (metabolomics/metabonomics) to large scale preclinical metabolism/toxicological (Clarke and Haselden 2008; Lindon et al. 2005; Wurtz et al. 2017), and clinical or epidemiological (Dunn et al. 2015; Lewis et al. 2016) investigations has delivered new insights into the underlying biology of health, toxicity and disease. The key analytical tools employed in metabolic profiling are currently nuclear magnetic resonance (NMR) spectroscopy and mass spectrometry (MS), with the latter either via direct infusion (DIMS) or coupled to a chromatographic separation technique such as gas (GC–MS) or high performance liquid/supercritical fluid chromatography (LC–MS/SFC–MS) or alternatively a separation based on capillary electrophoresis (CE–MS) (Begou et al. 2017; Putri et al. 2013). In LC, the performance improvements in metabolic profiling delivered by ultra (high) performance liquid chromatography (U(H)PLC) (Wilson et al. 2005), in terms of resolution, speed and sensitivity (a three- to fivefold increase), have resulted in analysis times in the range of 10–20 min per sample (Dunn et al. 2011; Lewis et al. 2016; Paglia et al. 2012) compared to 40–60 min for conventional HPLC (Clayton et al. 1998). This increase in throughput is generally acceptable for small-scale animal studies, where sample numbers are typically in the 100–500 range. However, the numerical challenge provided by the need for higher throughput in drug discovery programs and larger studies (> 500) such as those associated biobanking/epidemiology etc., would clearly benefit from a more efficient solution with the ability to perform rapid and reproducible acquisitions to help address the analysis time burden associated with the metabolic profiling of large cohorts. Thus, current methods for the analysis of studies consisting of 1000+ samples, can lead to an LC–MS instrument operating constantly for over 7 days (based on a 10-min LC methodology) in order to acquire the data in a single batch. This, in itself, can produce difficulties in logistical management for laboratories with limited staff and instrumentation. Additionally, lengthy analytical runs, or running large studies in multiple batches, can lead to a variation in ion response when looking at data across different days and increase the possibility of errors in data processing.

Reduced run time for this type of analysis (1–2 min per sample) has been achieved by eliminating the chromatography step and analysing the sample using DIMS (e.g. Chekmeneva et al. 2015). However, this approach can result in ion suppression, missing analytes and over estimation of metabolites due to isobaric interferences (Gray et al. 2016). An alternative approach to DIMS is to use a short (5 cm) UHPLC column with elevated mobile phase linear velocities and rapid gradients. This approach has been employed by the DMPK community for over 15 years for the high throughput quantification of drugs and metabolites in serum and plasma (Plumb et al. 2001). More recently this approach has been demonstrated in metabolic profiling studies for the analysis of moderately polar metabolites using reversed-phase (RP) UHPLC–MS for both 2.1 (Plumb et al. 2005) and 1 mm id columns (Gray et al. 2016).

However, the biological matrices utilised in metabolomic studies encompass many different classes of compounds, which consequently exhibit a broad range of chemical properties. Complete coverage of all of these compounds using RP separations can be difficult especially for polar analytes that are poorly retained. These polar compounds e.g. carbohydrates, organic acids and some biogenic amines, tend to elute in RPLC at the beginning of the gradient, often within the solvent front. This lack of retention results in inaccurate identification/quantification of these polar compounds due to analyte co-elution and interference/ion suppression with background ions/unretained salts etc. The adoption of hydrophilic interaction liquid chromatography (HILIC) has enabled many polar compounds to be analysed (Chauve et al. 2010) and, as a result, the dual analysis of samples such as urine using HILIC for the metabolic phenotyping of biofluids has become commonplace (Creek et al. 2011; Spagou et al. 2011) reviewed in e.g. (Spagou et al. 2010; Tang et al. 2016). Indeed the combination of RPLC and HILIC has begun to be used as standard practice where comprehensive profiles are required (Contrepois et al. 2015; Gika et al. 2008; Paris et al. 2016; Sun et al. 2017). Other modes of separation employed in the analysis of extremely polar compounds have proven to be reliable and reproducible, but in the case of e.g., normal phase and ion-exchange chromatography, compatibility issues with mass spectrometry and electrospray ionisation, due to the solvents and additives used, have made these techniques less attractive than HILIC for high throughput metabolomic studies.

In addition, advances in instrumentation have resulted in increasing interest in the application of ion mobility spectrometry (IMS) to metabolic phenotyping (Harry et al. 2008; Malkar et al. 2013; Rainville et al. 2017; Zhang et al. 2018) and lipidomics (Paglia et al. 2015a, b; Paglia and Astarita 2017; Shah et al. 2013).

Here we demonstrate a similar approach to the reversed-phase rapid microbore metabolic profiling (RAMMP) method described previously (Gray et al. 2016) for the analysis of polar metabolites in urine using HILIC–MS, either alone or coupled to IMS-enabled MS data acquisition (HILIC–IMS–MS). The information obtained from IMS separation can also be used to generate collision cross section (CCS) values representative of the interaction between the surface area of the ion and the drift gas (Paglia et al. 2015a). The resulting methodology was employed for the monitoring of the urinary metabolome resulting following the administration of the model human liver toxin tienilic acid (TA) to the rat.

## Experimental

### Chemicals and reagents

Water was obtained from an in-house Milli Q filtration system Millipore (Billercia MA, USA). LC–MS grade acetonitrile, methanol, isopropanol and formic acid were purchased from Sigma Aldrich (Gillingham, UK). Tienilic acid and its isomer were synthesised as described elsewhere (Rademacher et al. 2012). The LC–MS QC standard mixture, containing sulfadimethoxine, sulfaguanidine and leucine encephalin (used here as a system suitability test) was obtained from Waters Corporation (Milford MA, USA) and the major mix IMS/Tof calibration kit was obtained from Waters Corp (Wilmslow, UK).

### Biological sample preparation for HILIC separation

Urine samples were obtained from male Sprague–Dawley rats (supplied by Charles River Laboratories (Portage Ml), 250–300 g in weight) dosed intravenously with either 250 mg/kg of TA or vehicle, as described in detail elsewhere (Coen et al. 2012). Urine samples were collected at three time points covering the periods 0–2, 2–6 and 6–24 h post dose. These urine samples were then diluted 1:9 v/v with LCMS grade water, then centrifuged at 13,000*g* to remove any particulates before 900 µL of acetonitrile was added to 100 µL of the supernatant. A quality control (QC) sample was generated by pooling aliquots of each sample and analysed after every 10 samples throughout the course of the analytical batch (Gika et al. 2008). All the samples were thawed once prior to processing and QC generation. The individual study samples were injected in triplicate to make a total study batch containing 134 injections (not including pre-analysis system conditioning injections). The study was performed at Michigan State University and subject to institutional ethical approval from an ethics committee and conducted subject to national guidelines for the conduct of animal studies.

### Rapid HILIC–MS/MS and HILIC–IMS–MS/MS method

Chromatography was performed on a Waters ACQUITY I-Class chromatography system (Waters Corp, MA, USA), with the rapid microbore HILIC separation of the polar metabolites performed on a Waters BEH HILIC amide column (1.7 µm) with an internal column diameter of 1.0 mm and an overall length of 5 cm. A gradient elution profile was employed using mobile phases consisting of LC–MS grade deionised water and acetonitrile (Fisher Scientific, Loughborough UK) for phases A and B respectively, both containing 0.1% (v/v) formic acid. The flow rate was maintained throughout the separation at 0.2 mL/min with a column temperature of 50 °C and an injection volume of 0.2 µL. The separation was performed over 2.33 min with a starting composition of 99% mobile phase B. This composition was held at the acquisition start for 0.03 min before decreasing to 50% B over the next 2.30 min. The mobile phase composition was returned to starting conditions (99% B) over 0.04 min, followed by a column re-equilibration phase of 0.96 min, giving a total acquisition cycle of 3.33 min. The liquid chromatography conditions were generated by scaling the gradient, sample injection volume (3.5 µL) and flow rate (0.4 mL/min) from a conventional HILIC separation developed by Paglia et al. (2012) which employed a 10 min gradient elution profile and a 2.1 × 150 mm column. Mobile phase composition and column stationary phase was maintained.

Mass spectrometry data were acquired using a Waters Synapt G2-Si (Waters Corp, Wilmslow, UK) set to collect the data in continuum format using electrospray ionisation (ESI) in positive ionisation mode, over the mass range of m/z 50–1200. Capillary, sampling cone, and extraction cone voltages were set to 1.5 kV, 30 V and 5 V respectively, with the source temperature set to 120 °C and desolvation temperature 500 °C. Gas flow rates were set at 800 L/h for the desolvation gas and 50 L/h for the cone gas, and the mass spectrometer was set to acquire in sensitivity mode with a scan time of 0.1 s. Fragment ion information was acquired using collision induced dissociation and a collision energy ramp from 20 to 40 V. In order to decrease broadening of the chromatographic peaks, the internal diameter of the tubing connecting the column to the electrospray probe was reduced to 0.0025 inches and a reduced flow (50 µL) capillary probe was used. Real time lockmass correction was achieved by infusing leucine enkephalin at 10 µL/min through a lockspray probe and acquired every 30 s.

Additionally, each sample was also analysed with the ion mobility function of the Synapt G2-Si enabled. The ion mobility T-wave velocity was set to 650 m/s, with a pulse height of 40 V and nitrogen was used as the drift gas, set to a flow rate of 180 mL/min. Calibration of the ion mobility function for determining compound collision cross section was achieved using the Waters major mix IMS/Tof calibration kit.

### Data processing

All multivariate raw data were processed using Progenesis QI (Nonlinear dynamics, Newcastle UK) which performed run alignment, peak picking, adduct deconvolution and eventual feature (linked mass/retention time pairs) database searching. Principle component analysis (PCA), using Pareto scaling, was performed using Ezinfo (Umetrics, Umeå SE) in order to visualise group separation, and orthogonal partial least squared discriminate analysis (OPLS-DA) to identify significant features. In the assessment of chromatographic performance, specific target compounds were extracted and integrated using TargetLynx (Waters Corporation, Milford USA). The databases used to generate potential identifications were the Metlin MS/MS (Scripps Institute, CA, USA) and the human metabolome database (HMDB) (Wishart et al. 2018) with precursor and fragmentation ion accuracy set to 10 ppm. For CCS measurements, the IROA CCS database (Waters Corporation, Milford USA) with a tolerance to the database value of 2.5% was used.

## Results and discussion

### Chromatographic method development

The aim of the present study was to develop a rapid HILIC-based separation for the analysis of polar compounds to complement the rapid RP-microbore method previously described by Gray et al. (2016) which demonstrated the utility of a rapid method for the metabolic profiling of samples such as urine. For this a HILIC method used for the analysis of polar biological compounds on a conventional 2.1 × 150 mm analytical column (Paglia et al. 2012) was adapted for a 1.0 × 50 mm microbore column. The acquisition time, gradient and profile were geometrically scaled in order to maintain the same separation and retention characteristics using the smaller column compared to the original separation. The mobile phase linear velocity was increased providing more column volumes of solvent to define the gradient, leading to improved peak capacity in addition to maintaining the appropriate column volumes of mobile phase required for re-equilibration. This is an important consideration with HILIC-based methods as the long column re-equilibration times often needed in this LC mode can significantly extend the time required for sample analysis. In addition, reproducibility between samples can be greatly affected if a sufficient number of column volumes of mobile phase have not been used in the gradient re-equilibration phase.

**Fig. 1:**
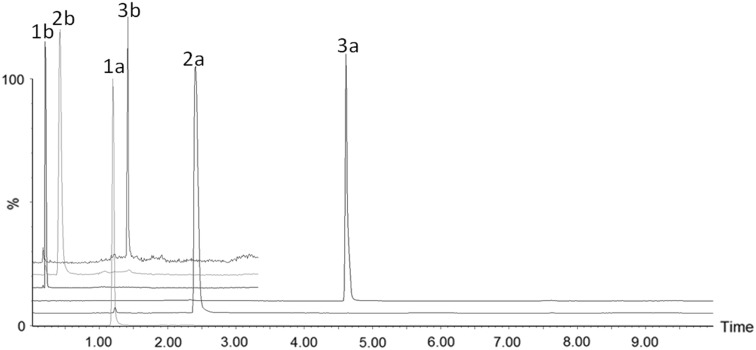
Overlaid XIC chromatograms from (a) the conventional 10 min HILIC analysis and (b) the 3.33 min. Rapid analysis of three system suitability standards (1) sulfadimethoxine (*m*/*z* = 311.08) (2) sulfaguanidine (*m*/*z* = 215.06) (3) leucine enkephalin (*m*/*z* = 556.28) demonstrating the conservation of the HILIC retention mechanism

The data displayed in Fig. 1 demonstrates the scaling of the previous HILIC based separation (Paglia et al. 2012) from a 12-min method on a conventional 2.1 mm column to 3.3 min, on a 1 mm id column, using the Rapid HILIC method (using three probe analytes present in the LC–MS QC sample mixture (1) sulfadimethoxine (*m*/*z* = 311.08), (2) sulfaguanidine (*m*/*z* = 215.06), (3) leucine enkephalin (*m*/*z* = 556.28). As can be seen from the resulting mass chromatograms, the relative retention of the three compounds has been conserved in the Rapid method (see also supplementary Table S1) providing an average peak width of approximately 3.5 s at the base. This Rapid HILIC method delivered narrower peaks than the conventional HILIC method, as would be expected from chromatographic theory, where peak width is directly proportional to column length, thus halving the column length should deliver narrower peaks. After confirming that the rapid method had been successfully scaled down by analysing the LC–MS QC mixture, the Rapid HILIC method was then applied in the analysis of biological samples ultimately enabling the analysis of the 134 samples in the sample set in ca. 7 h (compared to ca. 1 day for the conventional method). Despite the reduction in run time with this Rapid HILIC method, adequate retention of polar metabolites in rat urine was still achieved, as demonstrated in Figure S1, which shows extracted ion chromatograms of endogenous compounds tentatively identified as a hexose sugar (most probably glucose), 1- or 3-methylhistidine, kynurenic acid and creatinine.

In order to rapidly assess the robustness of the chromatographic separation within an analytical batch, a selection of endogenous compounds (Table 1) known to be present in urine that possessed different chromatographic properties and eluted across the gradient, were used to monitor changes in retention time, integrated peak area and peak asymmetry. Each compound was integrated from all of the pooled quality control samples used to monitor the analysis of the batch of 134 sample injections. The coefficient of variance (% CV) of retention time, peak area and asymmetry was calculated for each of these compounds, as shown in Table 1. The system showed excellent reproducibility, with coefficients of variation for peak retention between e.g., 0.31% (tryptophan) and 6.3% (hexose sugar). The LC peak symmetry varied between 0.71 and 3.24, which highlights the range of chromatographic peak shapes associated with this mode of LC, and serves to illustrate the complexity of the HILIC based separation mechanism. Thus peak shape in HILIC is influenced by pH, buffer concentration, solvent composition, temperature and the ion-exchange capability of the stationary phase, making it difficult to optimize the methodology for all the different types of analyte present in complex biological matrices. These limited data, however, indicate that despite the rapid gradient and short re-equilibration time, the system produced excellent run-to-run reproducibility (e.g. see Figure S2).

**Table 1:** Values of chromatographic performance: retention time deviation, integrated peak area* variability and peak asymmetry values for selected endogenous compounds present in rat urine obtained from pooled QC injections (n = 17) across the rapid HILIC–MS analytical batch

Possible ID	*m/z*	Retention time			Area			Peak asymmetry		
	[M+H]^+^	Mean (n = 10)	Std dev	%CV	Mean (n = 10)	Std dev	%CV	Mean (n = 10)	Std dev	%CV
Kynurenic acid	190.04	1.22	0.01	0.53	4896.49	1000.24	20.43	3.24	0.16	4.89
Alanine	90.03	1.53	0.01	0.60	7.94	1.72	21.65	0.99	0.29	29.35
Tryptophan	205.07	1.26	0.00	0.31	519.64	128.62	24.75	1.63	0.22	13.38
Hexose sugar	181.07	0.29	0.02	6.23	140.01	29.95	21.39	1.47	0.22	14.87
Threonine	120.06	1.49	0.01	0.43	15.09	3.83	25.40	1.43	0.33	22.91
Creatinine	114.07	1.39	0.00	0.33	4747.41	241.92	5.10	0.71	0.10	14.54
Indole	118.06	0.34	0.01	2.49	561.51	90.98	16.20	1.34	0.23	17.40
Leucine	132.10	1.48	0.00	0.33	404.50	72.16	17.84	2.09	0.49	23.27
1- or 3 -methylhistidine	170.06	0.53	0.02	2.90	447.30	117.79	26.33	1.49	0.33	22.29

See also Table S2 for further information on integrated peak area* variability on all the features showing a CV of less than 30% in the data obtained for the 17 QC samples analysed in this study

### Biological samples

The Rapid HILIC/MS methodology was applied to the analysis of rat urine samples following the intravenous administration of either dose vehicle or tienilic acid as described elsewhere (Coen et al. 2012). The system showed excellent tolerance to the analysis of rat urine (which was diluted 1:9 with acetonitrile), as demonstrated by the stability of the retention time, peak area and peak asymmetry data provided in Table 1 for a selection of the detected features. Analysis of the resulting data using multivariate analysis (PCA) revealed a total of 3007 LC–MS features in the samples, with an average peak width of 6.6 s at the base, resulting in a peak capacity of 28 for the 3-min separation. Based on bioanalytical guidance provided by the FDA (FDA 2001) a % CV of < 15–20% has been widely regarded as the standard for acceptance of data for drug bioanalysis using targeted LC–MS assays. However, for biomarkers the FDA Guidance acceptance criteria are somewhat less strict, and pragmatically have generally been increased for metabolic phenotyping to allow a % CV of 20–30% for untargeted metabolomic profiling methods (Brunius et al. 2016; Dunn et al. 2012). It should be noted that, as we have reported elsewhere, the CV of detected peaks is response dependent (e.g. see Gika et al. 2008) with less intense peaks showing lower repeatability (see Figure S3a). Using these criteria some 750 of the features detected by HILIC–MS in the QC samples had CVs of less than 30%, with 575 having CVs of less than 20% and 429 with CVs of less than 15% (77 and 57% of these totals respectively). These data are provided in Table S2. Whilst the number of detected features is undoubtedly lower than that obtained with conventional HILIC LC–MS analysis this is entirely to be expected [see e.g., the previously reported results for the RAMMP reversed-phase RP method compared to a conventional separation (Gray et al. 2016)]. However, the PCA data showed a clear separation of the tienilic acid samples from those of the controls. The drug metabolite profile in these samples has been previously investigated (Coen et al. 2012; King et al. 2018) and examination of the HILIC–MS data for the presence of either drug or drug-related signals [easily identifiable due to the characteristic chlorine isotope pattern provided by the presence of 2 chlorine atoms present as substituents on the drug (King et al. 2018)] showed that they were sufficiently hydrophobic to have eluted close to, or in, the solvent front and therefore did not contribute to the PCA separation. The results from OPLS-DA of these data are shown in Figure S4 demonstrating the group separation between TA- and vehicle-dosed animals with intergroup separation across the y-axis for the TA-dosed animals. This corresponds, to some extent, to the sample collection time point with the TA-dosed animals following a time (and animal) dependent metabolic trajectory over the 24 h time course of the study. The inter-animal differences highlighted indicates a, not unexpected, degree of variability in the response of these animals to drug treatment. However, by 24 h post dose all of the animals appeared to occupy a similar “metabolic” space. The related S-plot is shown in Figure S5 highlighting those features of greatest significance to the group discrimination between the groups.

Critical to successful metabolic profiling is the acquisition of searchable precursor and product ion MS data, enabling drug, drug metabolite and endogenous compound identification by either database searching or classical de novo data interpretation. Modern database searching tools employ a scoring algorithm to grade quality of the data match (Benton et al. 2008). Therefore, any metabolic profiling analytical system should provide acceptable MS (MS/MS) data quality, which in turn relies on the capability of the LC system to minimise analyte co-elution. A selection of endogenous ions were used to assess the analytical performance of the HILIC separation (Table 1) in addition to undergoing database searches using Metlin MS/MS (Scripps Institute, CA, USA) and the HMDB, in order to generate potential compound identifications.

**Fig. 2:**
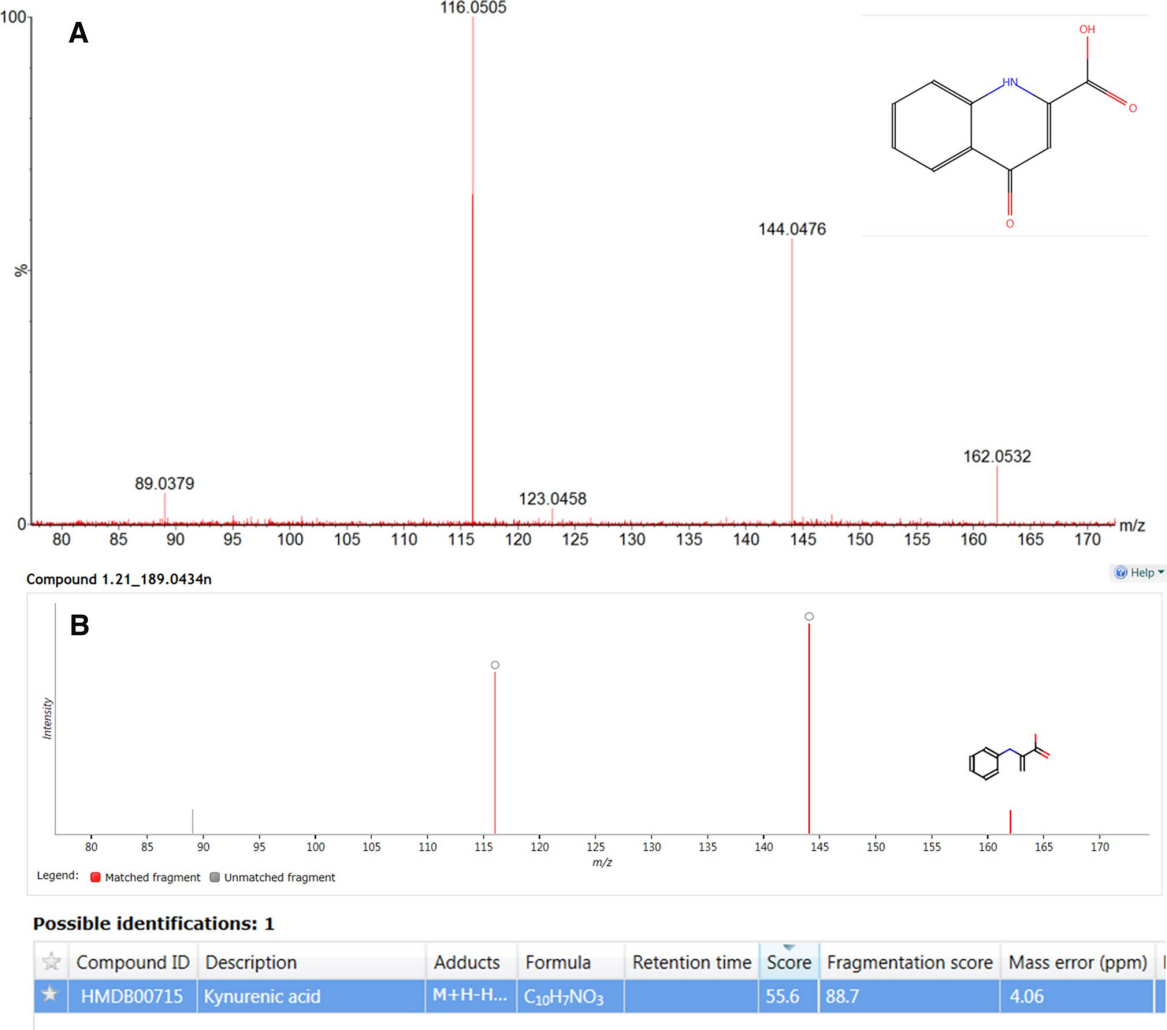
**a** High collision energy mass spectrum of *m*/*z* 190.0550 m/z at a retention time of 1.21 min, with fragment ions, **b** Progenesis QI output of corresponding feature (nominal mass of 189.0434 Da) at a retention time of 1.21 min with HMDB database search result of kynurenic acid

The extracted MS/MS spectrum for *m*/*z* 190.04 ion, with the database ID of kynurenic, acid is displayed in Fig. 2a, and shows excellent spectral quality data which was easily interpretable and resulted in an experimental to theoretical fragment ion score of 88.7 (Fig. 2b). Similar good-quality high collision energy spectral data were obtained for compounds provisionally identified as a hexose sugar e.g. glucose (Figure S6) and either 1- or 3-methylhistidine (Figure S7).

### Ion mobility spectrometry (IMS) enabled HILIC–MS (HILIC–IMS–MS)

As previously described, the shorter column length and analysis time employed in the RAMMP methodology inevitably results in reduction in the number of features detected in the sample when compared to standard 10 to 20 min analytical methodologies (Gray et al. 2016). Some signal loss can be attributed to ion suppression in the source as a result of analyte co-elution and matrix effects. However, some of the reduction in detected features may also be due to co-elution of compounds, including isobars, and the “masking” of signals from low signal intensity metabolites by more intense signals from co-eluting compounds.

**Fig. 3:**
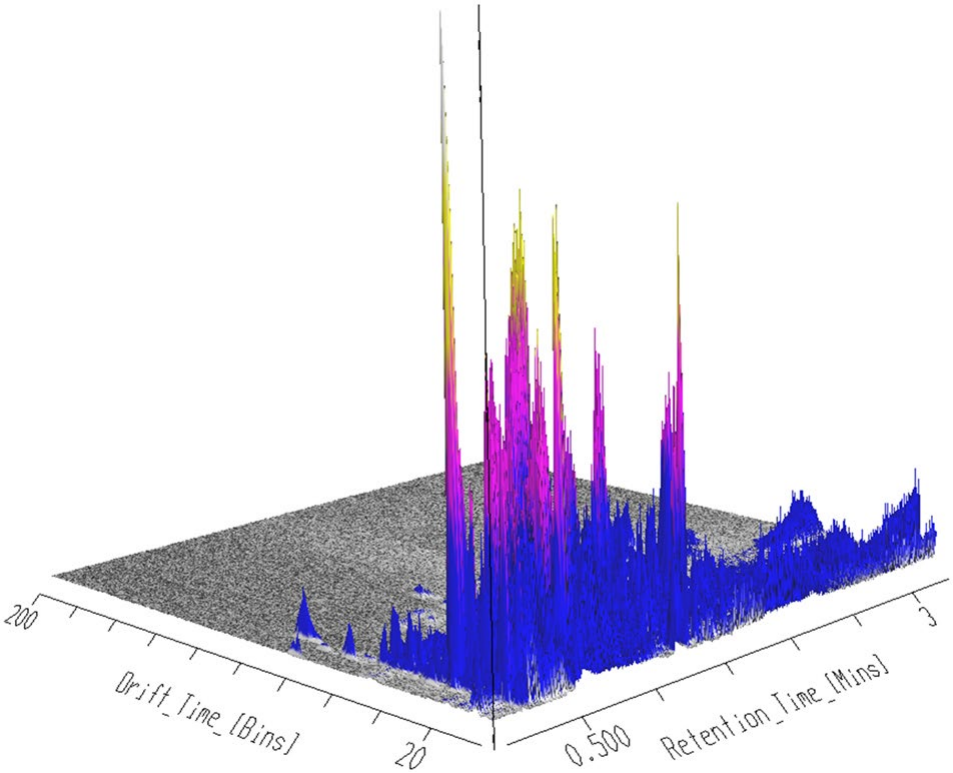
A 3D plot of a pooled rat urine QC showing chromatographic retention time, IMS drift time separation and ion intensity. Co-eluting ions have been separated by the IMS with some at a lower intensity otherwise hidden by more intense species

The millisecond time scale of IMS separations is conveniently in-between those of chromatography (seconds) and TOF–MS, where spectral data is acquired in microsecond packets. This allows IMS enabled MS to facilitate an extra dimension of resolution to the analytical process as well as providing “cleaner” mass spectra for individual analytes by reducing the number of co-eluting interferences. Previously we have shown that, for the same reversed-phase LC conditions, UHPLC–IMS–MS delivered approximately 30–50% more detected features than UHPLC–MS alone (Rainville et al. 2017). As we have previously noted, this cannot be because of a reduction in matrix effects as this separation occurs post ESI, but must rather be due to the “unmasking” of spectra from minor components previously hidden by the dominant component eluting from the column at that particular retention time (Rainville et al. 2017). To evaluate the impact of IMS on the Rapid HILIC separation the rat urine samples were reanalysed on the same LC–MS platform with IMS enabled. The resulting Rapid HILIC IMS–MS/MS analysis of the urine QC samples, which resulted in the detection of some 6711 features, is illustrated in Fig. 3, which shows a 3-D plot where the IMS drift time is plotted against retention time and analyte response. This graphically illustrates that numerous ions that co-eluted under the LC/MS conditions used were resolved by IMS, and thus are uniquely detected by the combination of LC–IMS–MS/MS. A further illustration of this enhanced separation is shown by employing a 2D ion map (Figure S8) where the complexity of the data can be clearly observed.

Using the criteria of a CV of 30% or less a total of 950 features present in the QC samples were acceptable for HILIC–IMS–MS, with 526 having CVs of less than 20% and 211with a CV of less than 15% (55 and 22% of the total respectively). These data are provided in Table S3. This increased variability in response seen with the IMS data, compared to the conventional MS results, could be attributed to the introduction of variability in ion transmission through the ion mobility cell or, as discussed earlier, to the natural increase in variability resulting from an increase in the number of low intensity minor features detected (Gika et al. 2008) (see Figure S3b). The resulting PCA and OPLS-DA of the data derived from this analysis again showed good separation of the test and control animals (see Figure S9). Once again the spread in the data for the TA-dosed animals reflects time-related changes in response to drug administration similar to those seen with HILIC–MS.

**Fig. 4:**
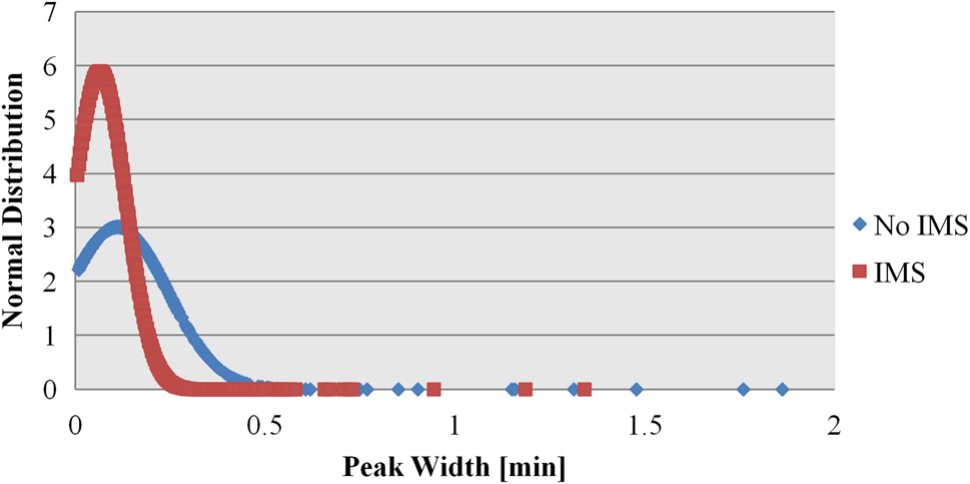
Distribution of feature peak width from the Rapid HILIC acquisition with and without IMS

A careful inspection of these data revealed that the average observed peak width seen with the combined LC–IMS–MS separation was 3.8 s compared to 6.6 s for LC–MS alone (Fig. 4), giving an increase in LC peak capacity from 28 to 51. The added capacity to separate analytes and increase system peak capacity should provide improved mass spectral data thus giving higher confidence in metabolite identification and resulting in fewer false positives. The reduction in observed LC peak width for the IMS-enable analysis can probably be attributed to the resolution of co-eluting isobaric metabolites. It is also noteworthy that, even though IMS–TOF–MS is widely reported to be less sensitive than standard TOF–MS, the multivariate statistical analysis of the IMS-enabled data set resulted in an increase in detected features from the 3007 by LC–MS to 6711 using LC–IMS–MS.

**Fig. 5:**
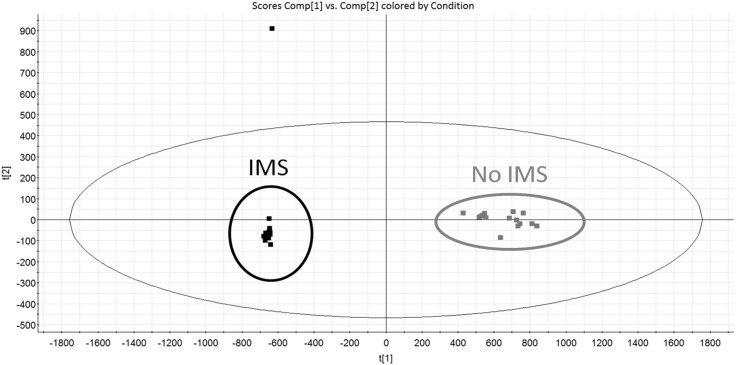
Principal Component Analysis comparing the pooled quality control rat urine from the Rapid HILIC assay acquired with and without ion mobility highlighting the improved QC clustering with IMS data

The IMS and non-IMS data from the QC samples were subjected to PCA with the result displayed in Fig. 5. Here we can see that the two data groups are clearly separated with, in addition, apparent improved analytical robustness indicated by the tighter clustering of the IMS QC samples when compared to non-IMS QCs. The features detected in the metabolic profiling analysis were subjected to a database search based on precursor ion *m*/*z* and fragment ion information. The potential identity of these molecules was then further supported by comparing the experimentally derived ion mobility CCS data with that of the Waters IROA CCS database, built using CCS values generated from authentic compound standards. As an example, the data shown in Figure S10 illustrates the database search for 4-hydroxy-2-quinolinecarboxlic acid (kynurenic acid), where the experimentally determined CCS result is within 0.32 of the database value, with additional CCS values presented in Table 2. These experimentally determined CCS measurements are within 5% of the CCS values from the experimentally derived CCS database and those of the theoretical CCS values (Zhou et al. 2016). The added capacity to separate metabolites, and thereby increase peak capacity, provides higher confidence in metabolite identification and should result in fewer false positives.

**Table 2:** Measured CCS values for endogenous target compounds (§) in addition to the predicted CCS measurement (*) generated from the MetCCS predictor (http://www.metabolomics-shanghai.org/MetCCS/) (Zhou et al. 2016), with the difference in CCS result of the measured values compared to experimentally derived metabolic profiling CCS library (ǂ) and the predicted CCS value

Possible ID	*m/z*	CCS			
	[M+H]^+^	CCS (Å2) (measured^§^)	ΔCCS (Å2) (measured—DB^ǂ^)	CCS (Å2) (predicted*)	ΔCCS (Å2) (measured—predicted)
Kynurenic acid	190.04	134.9	0.32	139.7	− 4.8
Alanine	90.03	–	–	117.4	
Tryptophan	205.07	143.3	− 1.74	144.7	− 1.4
Hexose sugar (glucose)	181.07	133.4	–	136.7	− 3.3
Threonine	120.06	–	–	122.0	
Creatinine	114.07	120.4	− 1.03	119.6	0.8
Indole	118.06	122.1	− 0.25	124.7	− 2.6
Leucine	132.10	124.9	− 3.14	130.2	− 5.3
1- or 3- methylhistidine	170.06	132.1	–	138.8	-5.7

Based on the levels of compound identification criteria described by the Metabolomic Standards Initiative (MSI) (Sumner et al. 2007), kynurenic acid, tryptophan, creatinine, indole and leucine have been identified to level 2, with MS/MS and CCS database matches. The availability of two orthogonal sets of data, based on physicochemical properties, for comparison against both reference MS/MS and CCS libraries, as well as one built internally from experimental data, has increased the confidence in the resulting potential identifications. In order to achieve an identification at level 1, complementary data generated from an authentic standard is required, especially if the compound of interest is a potential biomarker candidate.

It is clear from the increasing number of applications of LC–IMS in metabolic phenotyping (Harry et al. 2008; Mairinger et al. 2018; Malkar et al. 2013; Rainville et al. 2017) and lipidomics (Hinz et al. 2018; Paglia et al. 2015a; Shah et al. 2013) that the combination of LC, IMS and MS has much to offer for both analyte separation and identification, and this can be expected to be an area of keen interest for the future.

## Conclusion

HILIC based microbore UPLC–MS performed using short columns, with rapid gradients and elevated mobile phase linear velocities, provides a suitable analytical platform with which to perform high-throughput endogenous metabolic profiling of polar metabolites in urine. Compared to a conventional UPLC–HILIC/MS method, the developed Rapid HILIC method provided a fourfold reduction in analysis time, a 75% reduction in solvent use, and an 18-fold reduction in sample consumption over a conventional HILIC UPLC method, whilst maintaining the ability to detect critical discriminating features between different sample groups. Combining both Rapid HILIC- and RP-RAMMP methods would provide an efficient and rapid means of screening large numbers of samples, especially when coupled with IMS mobility and MS to increase feature detection and improve metabolite identification.

## Electronic supplementary material

Below is the link to the electronic supplementary material.

Supplementary material 1 (DOCX 3241 KB)

## Data Availability

The Human metabolome database used in this study is available via http://www.hmdb.ca/ and the MetCCS predictor tool is available via http://www.metabolomics-shanghai.org/MetCCS/.
