# Supplementary material for: Development of a rapid profiling method for the analysis of polar analytes in urine using HILIC–MS and ion mobility enabled HILIC–MS
Source: Metabolomics. 2019 Jan 22;15(2):17. doi: 10.1007/s11306-019-1474-9 (PMC6342856; doi:10.1007/s11306-019-1474-9)
Supplement: Supplementary file 1 — Supplementary material 1 (DOCX 3241 KB) [file 11306_2019_1474_MOESM1_ESM.docx]

**Supplementary information**

Development of a Rapid Profiling Method For The Analysis of Polar Analytes in Urine Using HILIC-MS and Ion Mobility Enabled HILIC-MS.

Adam M King^1,2*^, Lauren G Mullin^3^, Ian D Wilson^4^, Muireann Coen ^4,5^, Paul D Rainville^2,3^, Robert S Plumb^2,3^, Lee Gethings^1^, Garth Maker^6^, Robert Trengove^2^.

1. Waters Corporation, Wilmslow, Cheshire, SK9 4AX, UK
2. Separations Science and Metabolomics laboratory, Murdoch University, South Street, Murdoch, WA 6150, Australia
3. Waters Corporation, Milford, MA, 01757, USA
4. Computational and Systems Medicine, Department of Surgery and Cancer, Faculty of Medicine, Imperial College London, U.K.
5. Discovery Safety, Drug Safety and Metabolism, IMED Biotech Unit, AstraZeneca, 1 Francis Crick Avenue, Cambridge CB2 0RE, UK
6. Medical and Molecular Sciences, School of Veterinary and Life Sciences, Murdoch University, South Street, Murdoch, WA 6150, Australia.

*Authors for correspondence: [adam_king@waters.com](mailto:adam_king@waters.com)

i.wilson@imperial.ac.uk

**Supplementary Figures S1-S9**


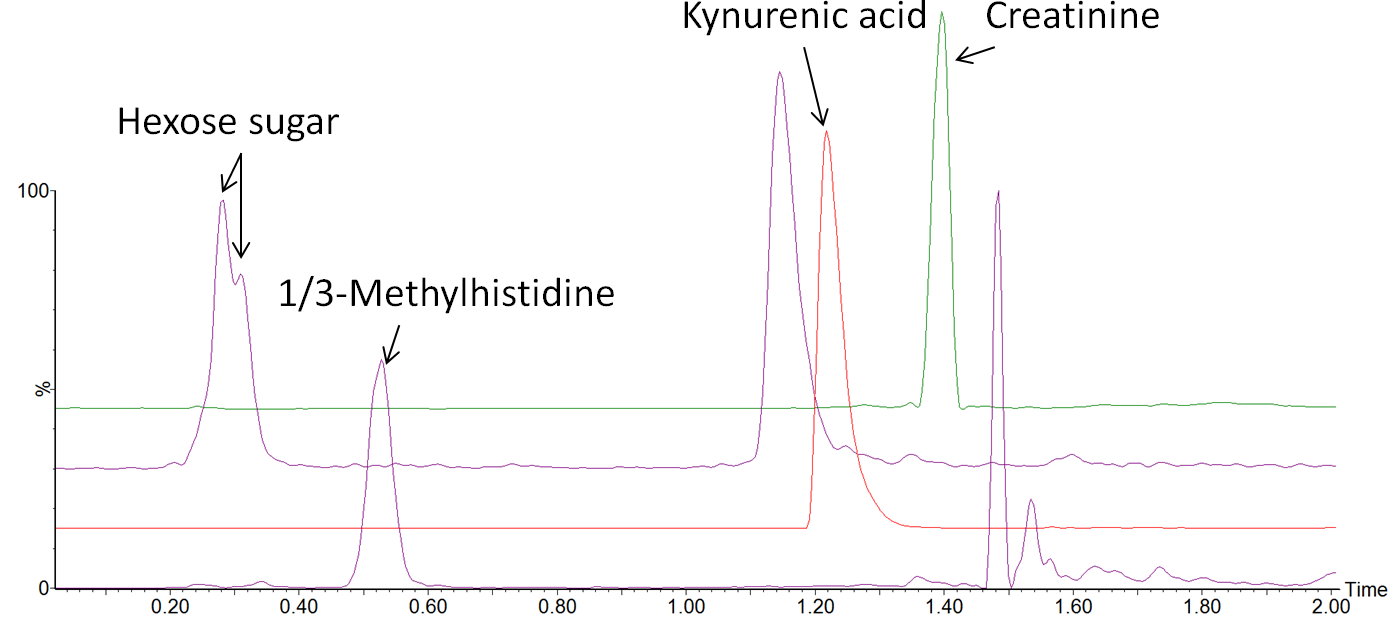


**Figure S1.** Extracted Ion Chromatograms of four endogenous compounds, a hexose sugar (e.g. glucose, galactose), 1- or 3- methylhistidine, kynurenic acid and creatinine from a pooled rat urine quality control sample.


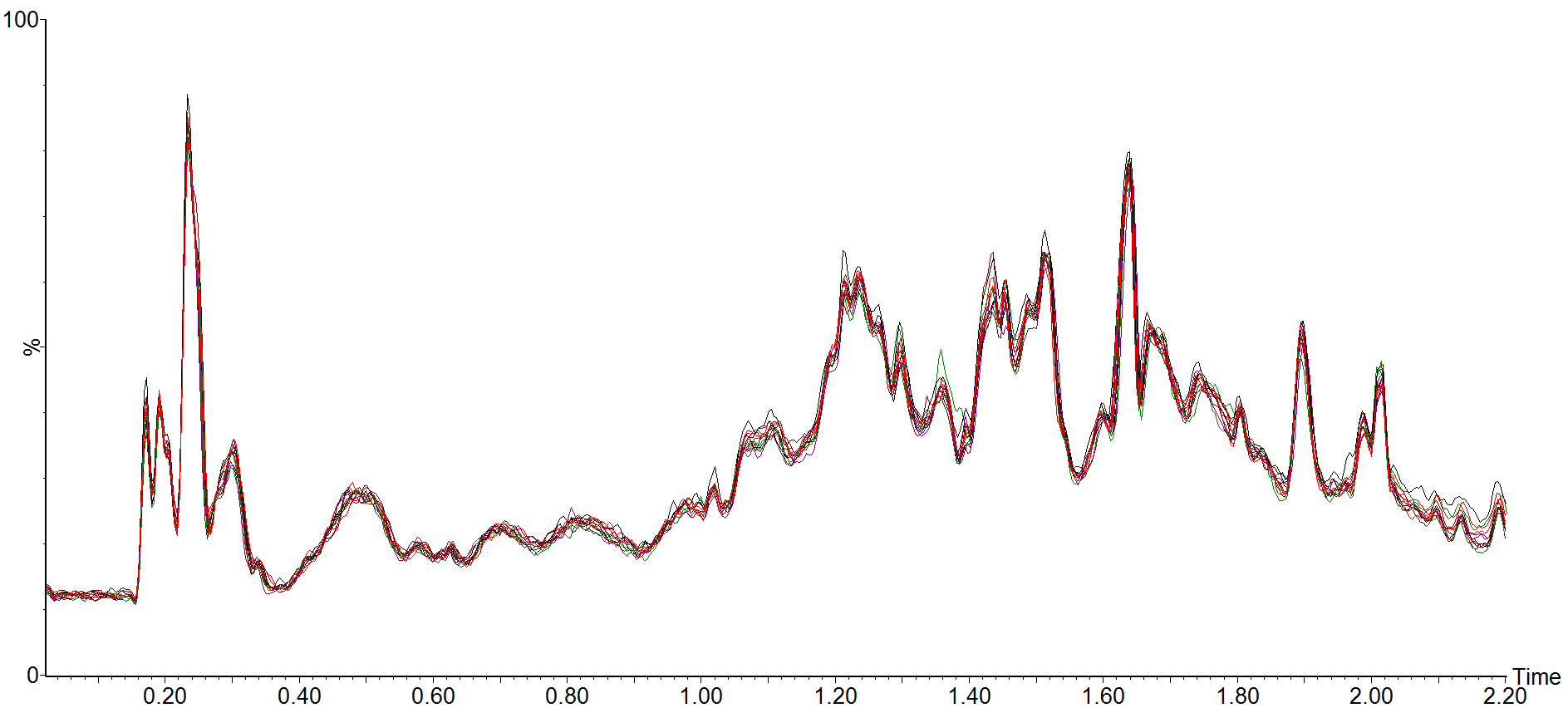


**Figure S2.** Overlaid Total ion current (TIC) chromatograms of a pooled rat urine quality control (QC) samples taken across the whole batch of 134 sample injections (including QCs).


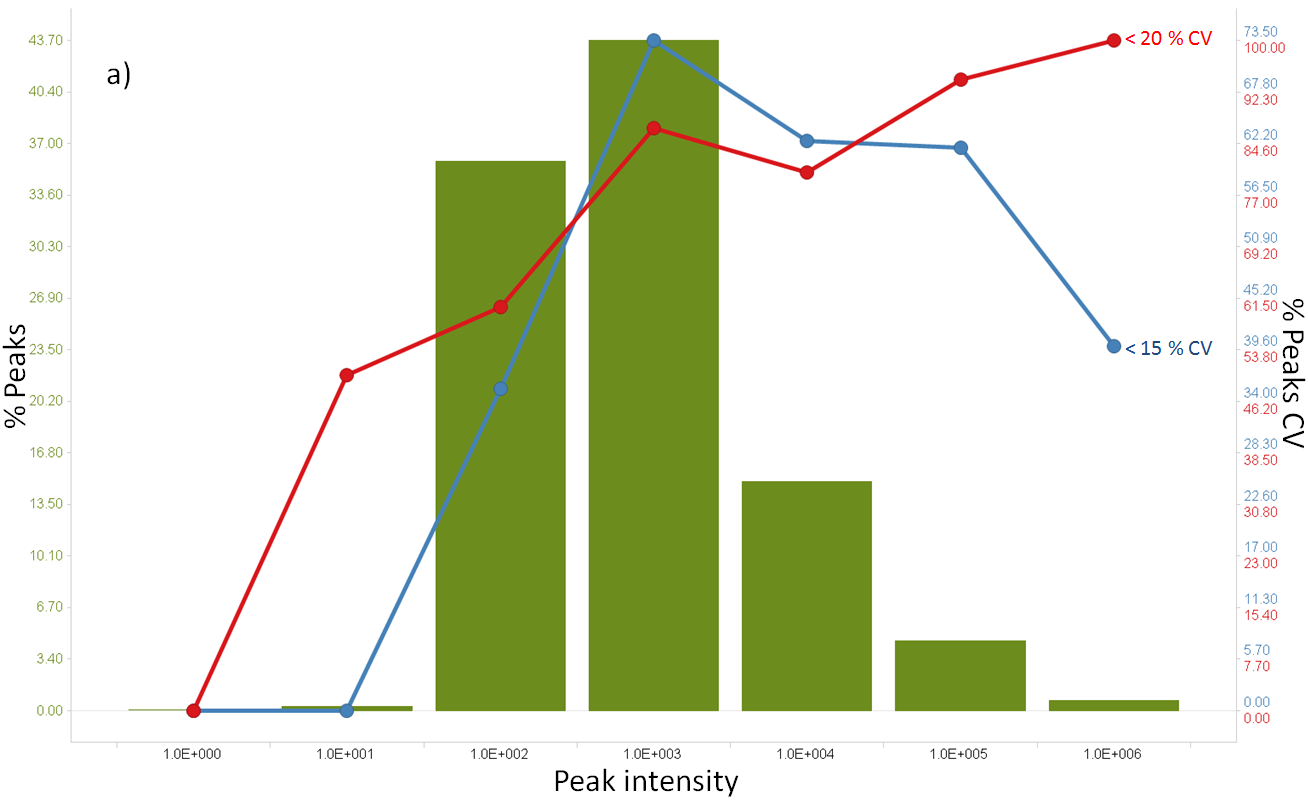

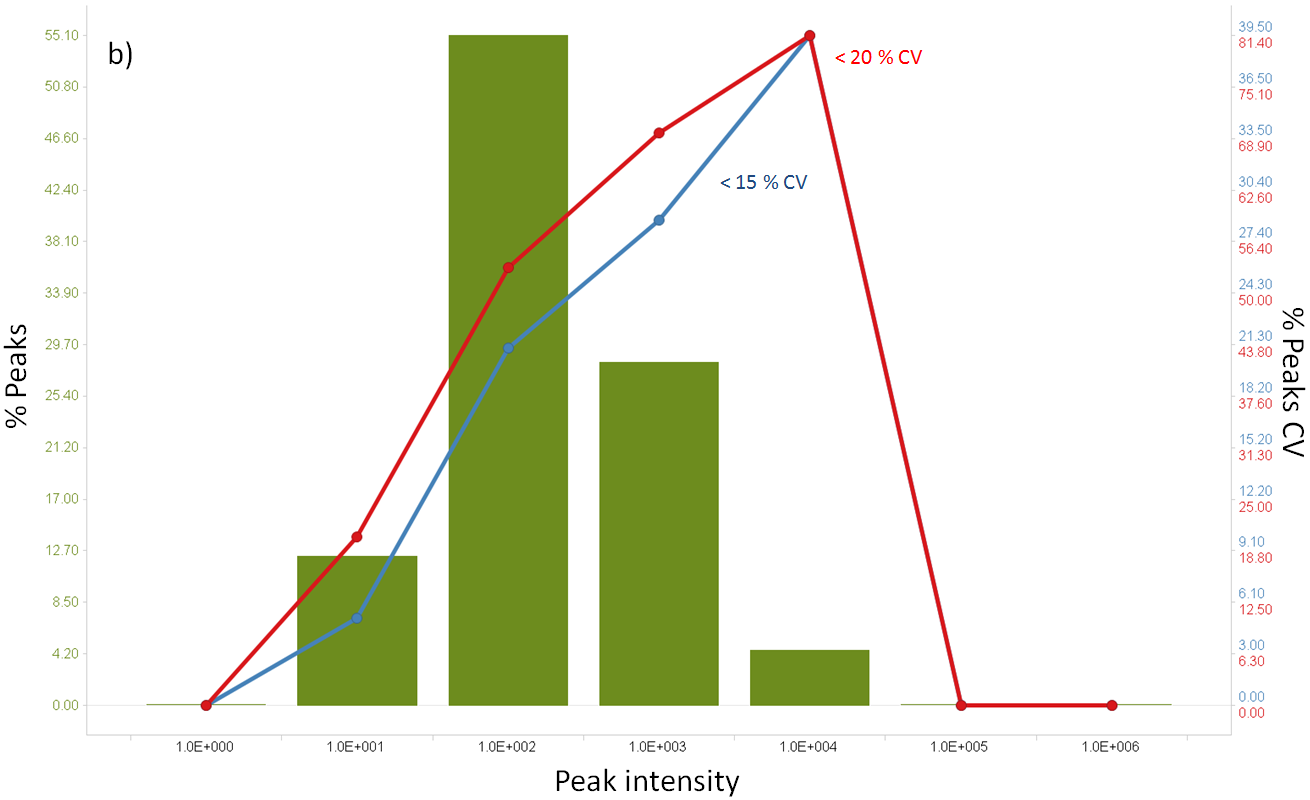


**Figure S3.** Feature intensity distribution for a) the Rapid HILIC-MS and b) the Rapid HILIC-IMS-MS methods. Green bars represent the percentage of features in the respective QC data with CVs of < 30% at each intensity. The red line represents the percentage of features showing CVs of < 20 % and blue line shows the percentage of features at < 15 % CV.

**
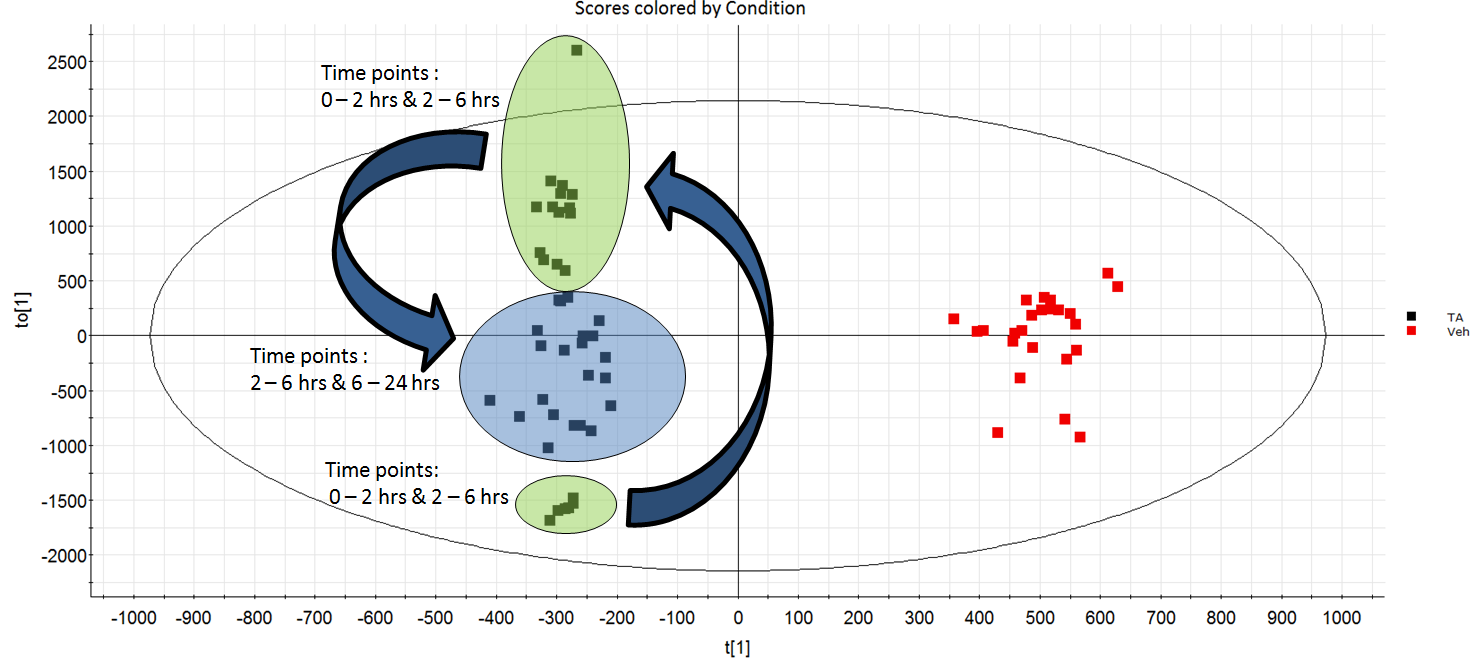
**

**Figure S4.** An OPLS-DA plot of rat urine Rapid HILIC-MS data obtained from tienilic acid (TA) treated and control animals (black symbols are tienilic acid dosed animal and red symbols animals treated with vehicle alone).


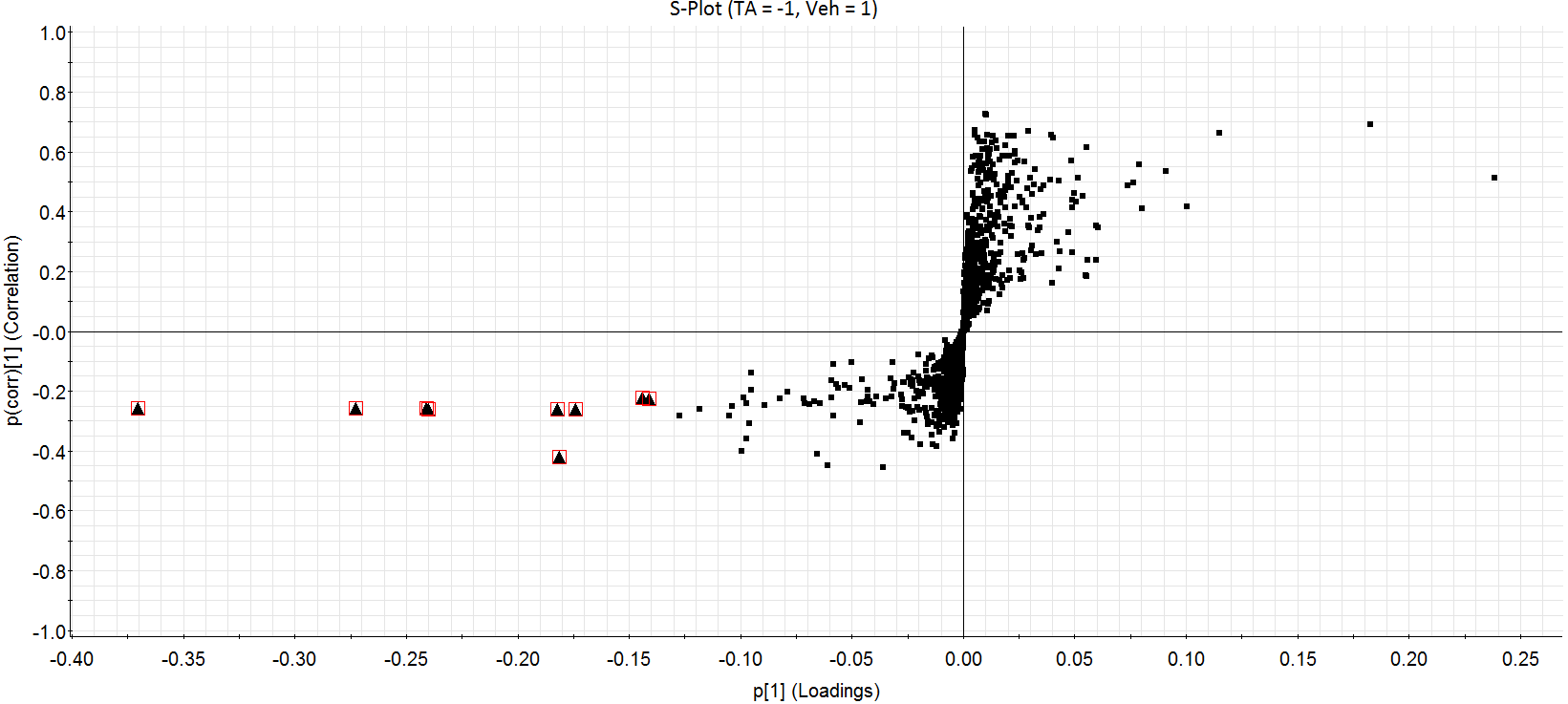


**Figure S5.** An S-plot showing significant features in Rapid HILIC-MS for TA treated compared to control animals dosed with the vehicle. Features of interest for future studies are indicated in the triangles outlined in red.


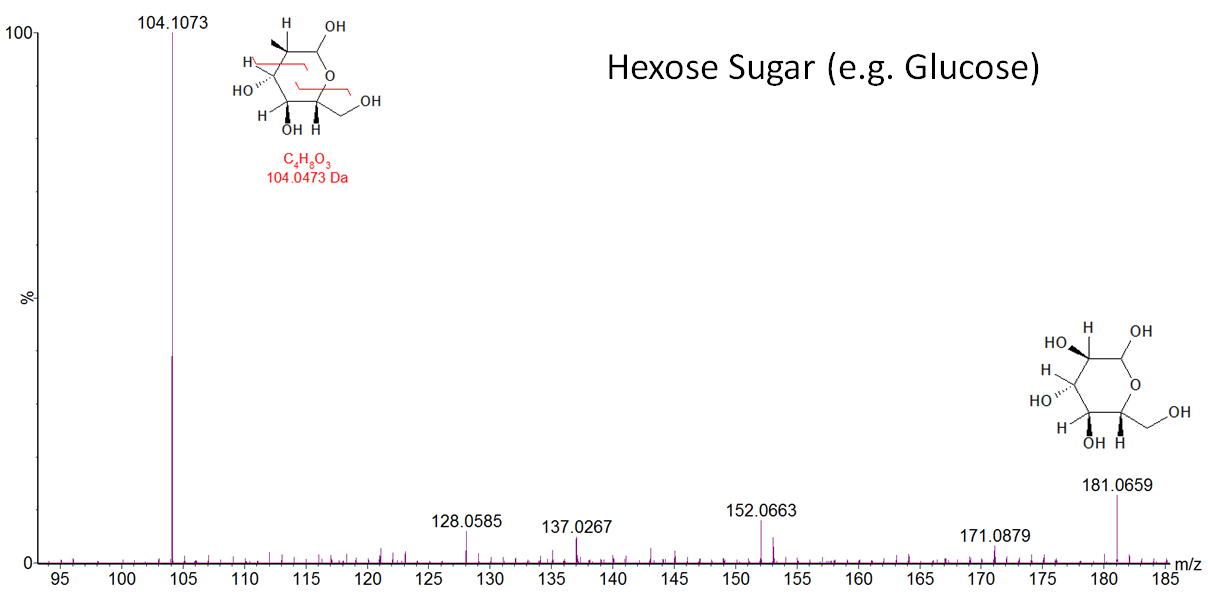


**Figure S6.** High collision energy mass spectrum of feature 181.0659 *m/z* at a retention time of 0.29 minutes with possible identifications of carbohydrates glucose or another hexose with predicted fragmentation.


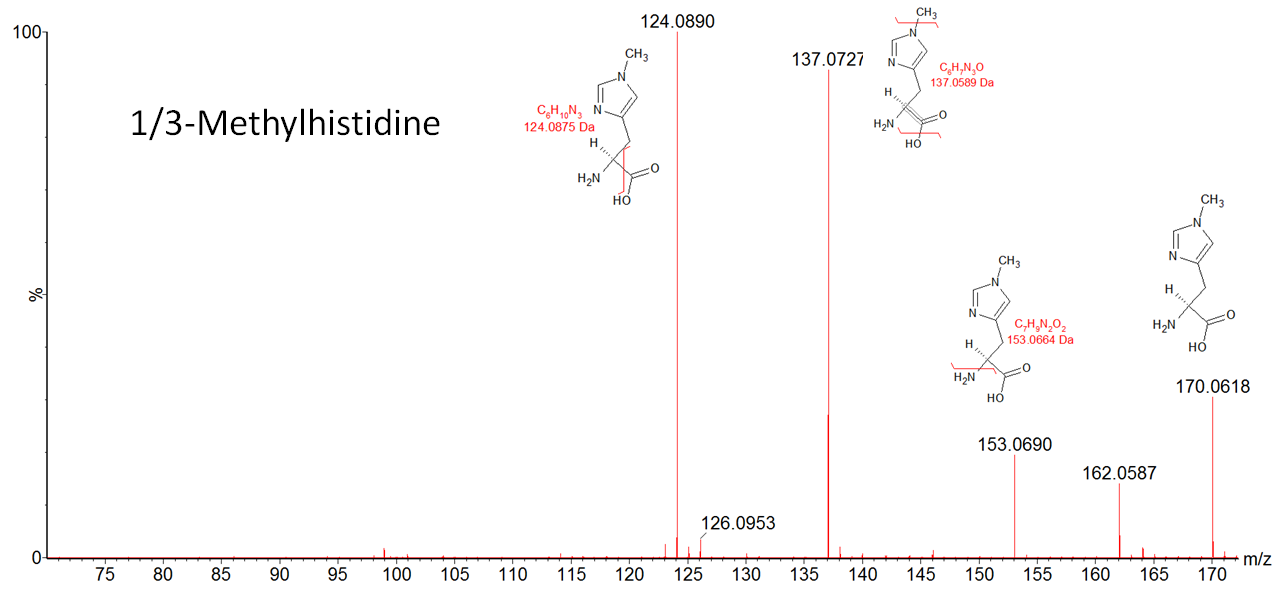


**Figure S7.** High collision energy mass spectrum of feature 170.0618 *m/z* at a retention time of 0.53 minutes with possible identification of 1- or 3-methylhisitidine and predicted fragmentation.

**Figure S8.** 2D ion map of a pooled rat urine QC showing chromatographic retention time and IMS drift time separation.


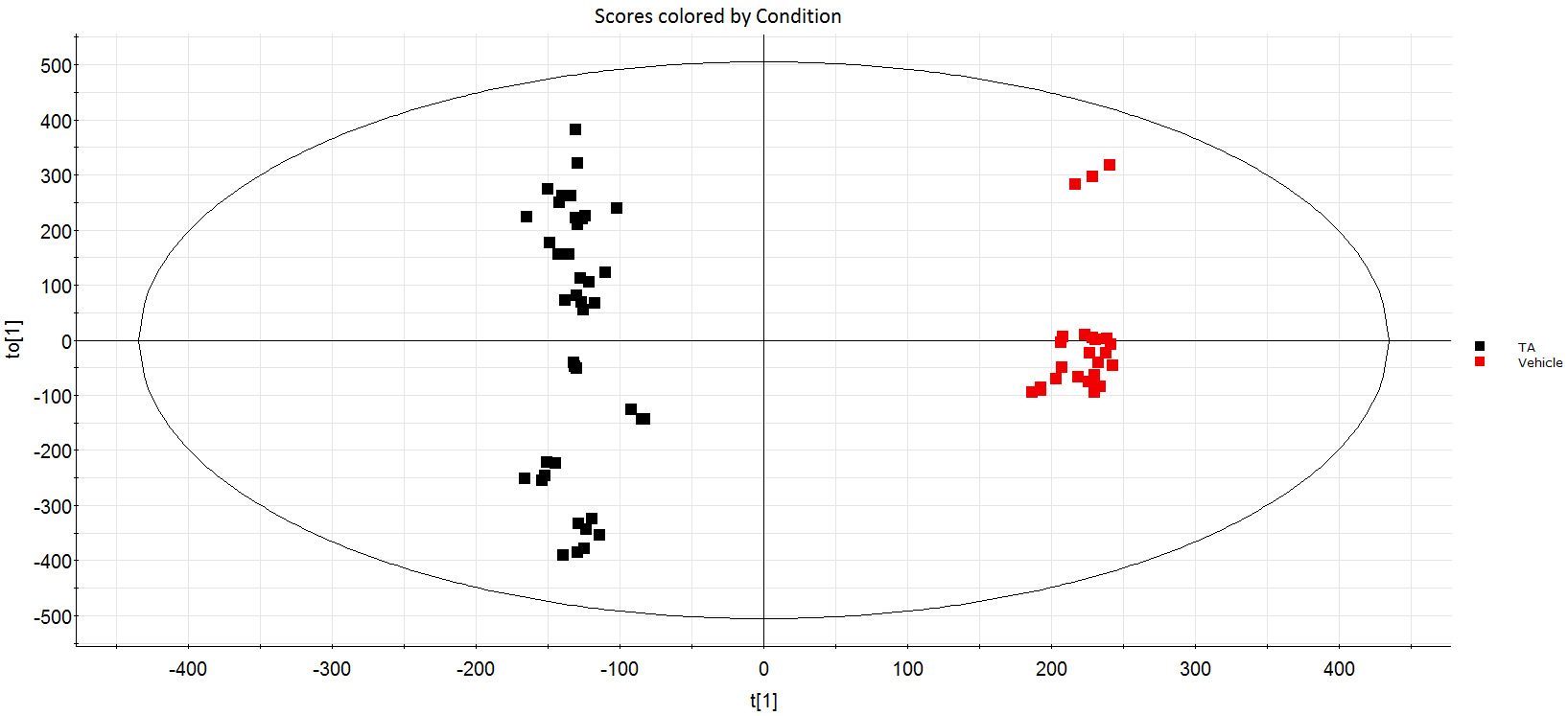


**Figure S9.** An OPLS-DA plot of rat urine Rapid HILIC-IMS-MS data obtained from tienilic acid (TA) treated and control animals (black symbols are tienilic acid dosed animal and red symbols animals treated with vehicle alone).


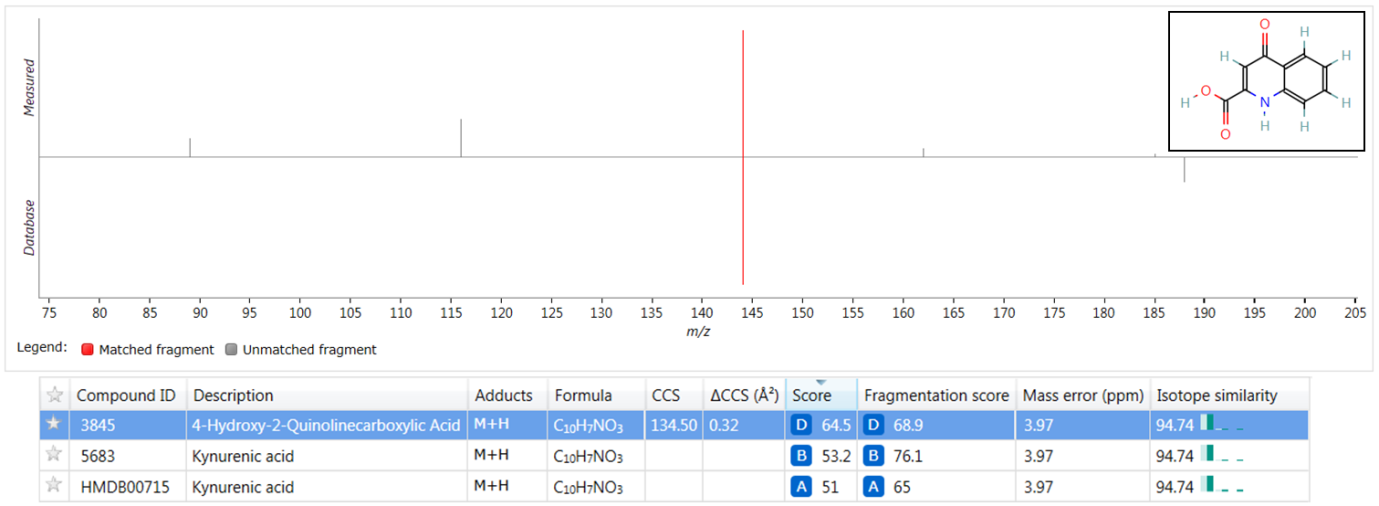


**Figure S10.** Waters IROA CCS database search for peak eluting at 1.20 minutes with *m/z* value of 190.0506 and CCS value of 134.50, alongside additional confirmatory HMDB and Metlin MS/MS database search results. The result of each database query provided kynurenic acid as the potential metabolite ID.

**Supplementary Tables Sl-S3**

**Table S1**. Relative retention time comparison of standards analysed using the standard HILIC separation and the Rapid HILIC assay.

|  | Rapid HILIC | | HILIC | |
| --- | --- | --- | --- | --- |
|  | RT | RRT to sulfadimethoxine | RT | RRT to sulfadimethoxine |
| Sulfadimethoxine | 0.20 | - | 1.20 | - |
| Sulfaguanidine | 0.45 | 2.25 | 2.40 | 2.00 |
| Leucine Enkephalin | 1.45 | 7.25 | 4.65 | 3.88 |

**Table S2.** List of features detected with %CV of abundance across QC sample injections during the Rapid HILIC MS analysis.

|  |  | Normalised abundance | | | | | | | | | | | | | | | | |  |  |  |  |
| --- | --- | --- | --- | --- | --- | --- | --- | --- | --- | --- | --- | --- | --- | --- | --- | --- | --- | --- | --- | --- | --- | --- |
| m/z | RT (min) | QC 1 | QC 2 | QC 3 | QC 4 | QC 5 | QC 6 | QC 7 | QC 8 | QC 9 | QC 10 | QC 11 | QC 12 | QC 13 | QC 14 | QC 15 | QC 16 | QC 17 | CV% | CV < 15 | CV < 20 | CV <= 30 |
| 64.9278 | 0.15 | 2.89E+01 | 2.85E+01 | 2.93E+01 | 2.52E+01 | 2.98E+01 | 8.24E+00 | 2.82E+01 | 2.87E+01 | 2.62E+01 | 1.75E+01 | 1.97E+01 | 2.59E+01 | 2.58E+01 | 1.75E+01 | 2.04E+01 | 1.53E+01 | 1.91E+01 | 26.7 |  |  | x |
| 103.9568 | 0.16 | 2.75E+04 | 2.46E+04 | 2.59E+04 | 2.53E+04 | 2.66E+04 | 2.51E+04 | 2.86E+04 | 2.90E+04 | 2.75E+04 | 2.68E+04 | 3.09E+04 | 3.23E+04 | 3.45E+04 | 3.15E+04 | 3.27E+04 | 3.50E+04 | 3.39E+04 | 12.0 | x | x | x |
| 105.9550 | 0.16 | 1.22E+04 | 1.10E+04 | 1.15E+04 | 1.11E+04 | 1.18E+04 | 1.09E+04 | 1.27E+04 | 1.28E+04 | 1.22E+04 | 1.17E+04 | 1.35E+04 | 1.42E+04 | 1.51E+04 | 1.38E+04 | 1.43E+04 | 1.54E+04 | 1.48E+04 | 11.6 | x | x | x |
| 144.9834 | 0.16 | 1.60E+04 | 1.41E+04 | 1.47E+04 | 1.40E+04 | 1.47E+04 | 1.38E+04 | 1.60E+04 | 1.60E+04 | 1.51E+04 | 1.48E+04 | 1.69E+04 | 1.77E+04 | 1.84E+04 | 1.70E+04 | 1.75E+04 | 1.88E+04 | 1.82E+04 | 10.2 | x | x | x |
| 146.9815 | 0.16 | 6.77E+03 | 5.97E+03 | 6.20E+03 | 5.85E+03 | 6.18E+03 | 5.53E+03 | 6.75E+03 | 6.69E+03 | 6.35E+03 | 5.99E+03 | 7.00E+03 | 7.28E+03 | 7.51E+03 | 7.00E+03 | 7.24E+03 | 7.69E+03 | 7.39E+03 | 9.7 | x | x | x |
| 186.0108 | 0.16 | 1.56E+02 | 1.23E+02 | 1.34E+02 | 1.32E+02 | 1.28E+02 | 1.06E+02 | 1.33E+02 | 1.28E+02 | 1.19E+02 | 1.25E+02 | 1.18E+02 | 1.05E+02 | 1.22E+02 | 1.15E+02 | 1.06E+02 | 1.06E+02 | 1.08E+02 | 11.2 | x | x | x |
| 355.0722 | 0.17 | 1.51E+02 | 2.00E+02 | 2.57E+02 | 4.06E+02 | 2.63E+02 | 1.76E+02 | 3.33E+02 | 3.46E+02 | 3.17E+02 | 2.56E+02 | 2.86E+02 | 2.92E+02 | 2.73E+02 | 3.05E+02 | 2.86E+02 | 3.20E+02 | 3.15E+02 | 22.4 |  |  | x |
| 536.1695 | 0.17 | 2.09E+03 | 1.95E+03 | 1.89E+03 | 1.88E+03 | 1.94E+03 | 1.04E+03 | 1.91E+03 | 1.92E+03 | 1.75E+03 | 1.53E+03 | 1.79E+03 | 1.92E+03 | 1.84E+03 | 1.73E+03 | 1.70E+03 | 1.78E+03 | 1.64E+03 | 13.0 | x | x | x |
| 610.1896 | 0.17 | 8.07E+03 | 7.34E+03 | 7.20E+03 | 7.23E+03 | 7.67E+03 | 5.14E+03 | 7.79E+03 | 7.77E+03 | 7.22E+03 | 6.65E+03 | 7.66E+03 | 8.20E+03 | 8.05E+03 | 7.91E+03 | 7.95E+03 | 8.28E+03 | 7.96E+03 | 10.0 | x | x | x |
| 637.3107 | 0.17 | 1.70E+02 | 1.55E+02 | 1.55E+02 | 1.52E+02 | 1.55E+02 | 9.41E+01 | 1.79E+02 | 1.60E+02 | 1.54E+02 | 1.21E+02 | 1.58E+02 | 1.70E+02 | 1.28E+02 | 1.33E+02 | 1.32E+02 | 1.61E+02 | 1.34E+02 | 14.4 | x | x | x |
| 659.2905 | 0.17 | 3.01E+01 | 2.56E+01 | 2.69E+01 | 1.92E+01 | 3.29E+01 | 8.46E+00 | 3.68E+01 | 2.23E+01 | 3.61E+01 | 2.87E+01 | 2.69E+01 | 3.10E+01 | 3.22E+01 | 2.61E+01 | 2.36E+01 | 2.18E+01 | 3.12E+01 | 25.4 |  |  | x |
| 684.2084 | 0.17 | 7.32E+02 | 6.44E+02 | 6.06E+02 | 6.05E+02 | 6.55E+02 | 2.94E+02 | 6.38E+02 | 6.28E+02 | 5.72E+02 | 4.51E+02 | 5.58E+02 | 6.09E+02 | 5.20E+02 | 5.62E+02 | 5.54E+02 | 5.15E+02 | 5.24E+02 | 17.0 |  | x | x |
| 337.1083 | 0.18 | 1.72E+01 | 1.71E+01 | 1.69E+01 | 1.25E+01 | 2.00E+01 | 5.90E+00 | 1.60E+01 | 1.59E+01 | 1.85E+01 | 1.76E+01 | 1.53E+01 | 1.12E+01 | 1.66E+01 | 1.25E+01 | 1.72E+01 | 3.99E+00 | 1.56E+01 | 29.3 |  |  | x |
| 158.1551 | 0.19 | 4.89E+01 | 4.47E+01 | 4.75E+01 | 4.76E+01 | 4.47E+01 | 3.24E+01 | 4.43E+01 | 4.87E+01 | 4.52E+01 | 4.33E+01 | 4.58E+01 | 4.33E+01 | 5.15E+01 | 4.59E+01 | 4.76E+01 | 4.44E+01 | 4.31E+01 | 8.9 | x | x | x |
| 213.1472 | 0.19 | 2.43E+01 | 2.50E+01 | 2.36E+01 | 3.47E+01 | 3.14E+01 | 2.91E+01 | 3.48E+01 | 3.71E+01 | 3.86E+01 | 3.86E+01 | 3.60E+01 | 4.21E+01 | 4.41E+01 | 4.11E+01 | 3.40E+01 | 4.31E+01 | 4.18E+01 | 18.9 |  | x | x |
| 229.1432 | 0.19 | 4.55E+03 | 4.37E+03 | 4.43E+03 | 4.92E+03 | 5.66E+03 | 6.15E+03 | 6.04E+03 | 6.32E+03 | 6.19E+03 | 6.19E+03 | 7.36E+03 | 8.18E+03 | 8.68E+03 | 8.30E+03 | 7.90E+03 | 8.70E+03 | 8.67E+03 | 23.6 |  |  | x |
| 239.0910 | 0.19 | 2.12E+01 | 2.16E+01 | 2.24E+01 | 2.82E+01 | 2.91E+01 | 1.78E+01 | 2.65E+01 | 4.16E+01 | 2.95E+01 | 2.96E+01 | 2.69E+01 | 3.26E+01 | 2.37E+01 | 2.47E+01 | 2.54E+01 | 2.50E+01 | 2.06E+01 | 21.0 |  |  | x |
| 245.1172 | 0.19 | 3.12E+02 | 3.91E+02 | 4.22E+02 | 3.65E+02 | 3.62E+02 | 7.21E+02 | 3.51E+02 | 4.49E+02 | 4.51E+02 | 3.84E+02 | 4.14E+02 | 5.01E+02 | 6.42E+02 | 4.42E+02 | 5.62E+02 | 6.07E+02 | 5.83E+02 | 24.8 |  |  | x |
| 257.1410 | 0.19 | 2.46E+01 | 2.61E+01 | 3.17E+01 | 4.07E+01 | 3.47E+01 | 2.56E+01 | 3.22E+01 | 3.80E+01 | 3.83E+01 | 3.11E+01 | 3.79E+01 | 4.18E+01 | 3.86E+01 | 3.35E+01 | 3.56E+01 | 4.09E+01 | 3.96E+01 | 15.9 |  | x | x |
| 326.3802 | 0.19 | 1.26E+02 | 1.35E+02 | 1.36E+02 | 1.43E+02 | 1.42E+02 | 1.66E+02 | 1.49E+02 | 1.54E+02 | 1.46E+02 | 1.11E+02 | 1.47E+02 | 1.76E+02 | 1.91E+02 | 1.68E+02 | 2.10E+02 | 1.99E+02 | 1.88E+02 | 17.3 |  | x | x |
| 340.3595 | 0.19 | 1.40E+01 | 1.86E+01 | 1.70E+01 | 1.64E+01 | 2.23E+01 | 1.57E+01 | 1.83E+01 | 1.47E+01 | 1.98E+01 | 1.11E+01 | 2.07E+01 | 2.01E+01 | 1.95E+01 | 1.61E+01 | 2.31E+01 | 2.47E+01 | 2.36E+01 | 20.0 |  | x | x |
| 377.2216 | 0.19 | 2.50E+01 | 3.11E+01 | 4.20E+01 | 6.40E+01 | 4.26E+01 | 3.80E+01 | 5.17E+01 | 6.03E+01 | 5.48E+01 | 4.53E+01 | 5.65E+01 | 5.13E+01 | 4.86E+01 | 4.96E+01 | 4.82E+01 | 5.08E+01 | 5.76E+01 | 21.1 |  |  | x |
| 171.1009 | 0.20 | 4.83E+01 | 4.05E+01 | 3.73E+01 | 3.94E+01 | 4.73E+01 | 3.63E+01 | 4.92E+01 | 4.78E+01 | 4.37E+01 | 4.33E+01 | 5.13E+01 | 5.92E+01 | 5.15E+01 | 5.28E+01 | 4.43E+01 | 4.24E+01 | 5.21E+01 | 13.3 | x | x | x |
| 153.1400 | 0.20 | 9.19E+00 | 9.64E+00 | 8.52E+00 | 1.04E+01 | 1.13E+01 | 1.25E+01 | 9.89E+00 | 1.01E+01 | 1.44E+01 | 1.23E+01 | 1.16E+01 | 9.61E+00 | 1.54E+01 | 1.82E+01 | 6.41E+00 | 1.68E+01 | 9.58E+00 | 27.0 |  |  | x |
| 284.3321 | 0.20 | 5.52E+01 | 4.91E+01 | 5.93E+01 | 4.38E+01 | 3.94E+01 | 4.50E+01 | 4.66E+01 | 5.44E+01 | 4.27E+01 | 3.29E+01 | 4.93E+01 | 3.93E+01 | 2.77E+01 | 3.21E+01 | 2.49E+01 | 2.68E+01 | 3.93E+01 | 24.7 |  |  | x |
| 306.0779 | 0.20 | 2.10E+02 | 2.01E+02 | 2.07E+02 | 2.39E+02 | 2.27E+02 | 1.41E+02 | 2.38E+02 | 2.09E+02 | 2.19E+02 | 1.96E+02 | 2.24E+02 | 2.45E+02 | 2.03E+02 | 2.11E+02 | 2.15E+02 | 2.26E+02 | 2.25E+02 | 10.9 | x | x | x |
| 304.3016 | 0.20 | 5.74E+01 | 5.84E+01 | 5.55E+01 | 5.01E+01 | 6.31E+01 | 6.43E+01 | 6.15E+01 | 6.64E+01 | 5.76E+01 | 4.51E+01 | 6.23E+01 | 6.29E+01 | 7.19E+01 | 6.27E+01 | 7.28E+01 | 7.85E+01 | 7.09E+01 | 13.4 | x | x | x |
| 332.3332 | 0.20 | 7.73E+01 | 6.70E+01 | 7.60E+01 | 7.70E+01 | 7.13E+01 | 7.78E+01 | 7.82E+01 | 7.72E+01 | 6.98E+01 | 6.61E+01 | 7.11E+01 | 8.37E+01 | 9.43E+01 | 8.12E+01 | 8.24E+01 | 8.62E+01 | 1.04E+02 | 12.2 | x | x | x |
| 98.0245 | 0.21 | 1.55E+01 | 1.49E+01 | 1.72E+01 | 1.55E+01 | 1.57E+01 | 1.38E+01 | 1.87E+01 | 2.18E+01 | 1.92E+01 | 1.74E+01 | 1.75E+01 | 1.43E+01 | 1.47E+01 | 1.80E+01 | 1.48E+01 | 1.97E+01 | 1.53E+01 | 13.4 | x | x | x |
| 126.0559 | 0.21 | 1.11E+02 | 9.74E+01 | 1.03E+02 | 1.07E+02 | 9.86E+01 | 9.46E+01 | 1.30E+02 | 1.16E+02 | 1.11E+02 | 1.10E+02 | 1.19E+02 | 1.14E+02 | 1.06E+02 | 1.23E+02 | 1.07E+02 | 1.22E+02 | 1.10E+02 | 8.6 | x | x | x |
| 148.1130 | 0.21 | 1.85E+01 | 3.11E+01 | 2.34E+01 | 2.56E+01 | 2.21E+01 | 2.45E+01 | 2.60E+01 | 2.91E+01 | 2.75E+01 | 2.42E+01 | 1.90E+01 | 2.00E+01 | 2.56E+01 | 3.89E+01 | 2.53E+01 | 3.30E+01 | 3.52E+01 | 21.2 |  |  | x |
| 151.1241 | 0.21 | 2.86E+02 | 2.78E+02 | 2.72E+02 | 3.13E+02 | 2.91E+02 | 4.48E+02 | 3.63E+02 | 3.90E+02 | 4.04E+02 | 3.92E+02 | 4.38E+02 | 5.27E+02 | 5.64E+02 | 5.46E+02 | 5.05E+02 | 6.05E+02 | 5.54E+02 | 26.6 |  |  | x |
| 157.0851 | 0.21 | 1.13E+01 | 1.05E+01 | 1.37E+01 | 9.73E+00 | 1.41E+01 | 1.35E+01 | 1.12E+01 | 1.41E+01 | 1.51E+01 | 1.62E+01 | 1.61E+01 | 1.61E+01 | 8.13E+00 | 1.09E+01 | 1.48E+01 | 1.60E+01 | 1.28E+01 | 18.9 |  | x | x |
| 202.2178 | 0.21 | 1.60E+02 | 2.00E+02 | 1.78E+02 | 1.89E+02 | 2.34E+02 | 2.53E+02 | 1.76E+02 | 2.02E+02 | 2.06E+02 | 1.84E+02 | 1.87E+02 | 2.07E+02 | 2.66E+02 | 1.99E+02 | 2.02E+02 | 2.09E+02 | 2.39E+02 | 13.8 | x | x | x |
| 164.1435 | 0.21 | 4.99E+01 | 4.72E+01 | 4.76E+01 | 5.78E+01 | 5.57E+01 | 6.24E+01 | 5.50E+01 | 6.12E+01 | 6.27E+01 | 5.45E+01 | 5.75E+01 | 5.63E+01 | 5.64E+01 | 5.92E+01 | 5.34E+01 | 4.07E+01 | 6.15E+01 | 10.9 | x | x | x |
| 178.1594 | 0.21 | 9.29E+01 | 8.61E+01 | 8.06E+01 | 8.68E+01 | 8.63E+01 | 8.45E+01 | 9.09E+01 | 8.39E+01 | 8.62E+01 | 6.76E+01 | 8.46E+01 | 7.78E+01 | 7.41E+01 | 7.68E+01 | 5.77E+01 | 6.68E+01 | 6.30E+01 | 12.9 | x | x | x |
| 227.9708 | 0.21 | 1.73E+01 | 1.57E+01 | 1.44E+01 | 1.01E+01 | 1.46E+01 | 1.44E+01 | 1.44E+01 | 1.23E+01 | 1.25E+01 | 1.05E+01 | 1.56E+01 | 1.51E+01 | 9.20E+00 | 1.06E+01 | 9.08E+00 | 1.01E+01 | 9.38E+00 | 21.2 |  |  | x |
| 277.2152 | 0.21 | 4.05E+01 | 3.79E+01 | 4.36E+01 | 4.03E+01 | 4.57E+01 | 4.66E+01 | 4.18E+01 | 4.16E+01 | 4.07E+01 | 3.05E+01 | 3.73E+01 | 2.77E+01 | 3.00E+01 | 3.18E+01 | 2.73E+01 | 2.57E+01 | 3.14E+01 | 18.7 |  | x | x |
| 273.1685 | 0.21 | 2.76E+02 | 3.15E+02 | 2.90E+02 | 3.61E+02 | 3.67E+02 | 3.19E+02 | 3.73E+02 | 4.01E+02 | 4.05E+02 | 3.51E+02 | 4.24E+02 | 3.89E+02 | 3.43E+02 | 3.16E+02 | 3.39E+02 | 3.61E+02 | 3.52E+02 | 11.5 | x | x | x |
| 356.3541 | 0.21 | 1.32E+02 | 1.09E+02 | 1.24E+02 | 1.11E+02 | 1.12E+02 | 1.22E+02 | 1.31E+02 | 1.17E+02 | 1.08E+02 | 8.12E+01 | 9.79E+01 | 1.23E+02 | 9.38E+01 | 1.16E+02 | 1.22E+02 | 1.02E+02 | 1.45E+02 | 13.5 | x | x | x |
| 331.1350 | 0.21 | 7.37E+01 | 7.01E+01 | 6.83E+01 | 7.13E+01 | 6.69E+01 | 4.73E+01 | 8.46E+01 | 7.53E+01 | 6.70E+01 | 5.86E+01 | 7.61E+01 | 6.39E+01 | 6.76E+01 | 5.97E+01 | 6.31E+01 | 6.16E+01 | 6.73E+01 | 12.4 | x | x | x |
| 358.3691 | 0.21 | 1.46E+01 | 1.28E+01 | 1.49E+01 | 1.25E+01 | 1.36E+01 | 1.48E+01 | 1.86E+01 | 1.30E+01 | 1.08E+01 | 1.29E+01 | 1.48E+01 | 6.24E+00 | 8.23E+00 | 1.66E+01 | 1.26E+01 | 1.06E+01 | 1.74E+01 | 23.6 |  |  | x |
| 425.1529 | 0.21 | 1.25E+03 | 1.21E+03 | 1.29E+03 | 1.11E+03 | 1.26E+03 | 1.54E+03 | 1.29E+03 | 1.29E+03 | 1.25E+03 | 1.06E+03 | 1.22E+03 | 1.20E+03 | 1.12E+03 | 1.03E+03 | 1.10E+03 | 1.13E+03 | 1.24E+03 | 9.9 | x | x | x |
| 387.1947 | 0.21 | 4.64E+02 | 3.90E+02 | 4.23E+02 | 4.50E+02 | 4.27E+02 | 4.20E+02 | 5.12E+02 | 4.86E+02 | 4.58E+02 | 4.06E+02 | 4.83E+02 | 4.75E+02 | 4.48E+02 | 4.76E+02 | 4.97E+02 | 5.19E+02 | 5.24E+02 | 8.6 | x | x | x |
| 102.1285 | 0.22 | 2.39E+02 | 2.20E+02 | 2.25E+02 | 2.43E+02 | 2.53E+02 | 3.17E+02 | 2.68E+02 | 2.77E+02 | 2.82E+02 | 2.69E+02 | 2.88E+02 | 3.24E+02 | 2.91E+02 | 3.34E+02 | 3.13E+02 | 3.37E+02 | 3.55E+02 | 14.3 | x | x | x |
| 233.0948 | 0.22 | 3.09E+01 | 3.46E+01 | 3.48E+01 | 3.36E+01 | 4.09E+01 | 3.12E+01 | 4.01E+01 | 4.00E+01 | 3.70E+01 | 3.71E+01 | 2.97E+01 | 3.64E+01 | 3.55E+01 | 2.98E+01 | 2.58E+01 | 1.59E+01 | 2.97E+01 | 18.5 |  | x | x |
| 130.1600 | 0.22 | 9.83E+00 | 1.16E+01 | 1.17E+01 | 1.14E+01 | 9.04E+00 | 1.27E+01 | 1.17E+01 | 8.99E+00 | 1.16E+01 | 1.05E+01 | 5.99E+00 | 9.27E+00 | 1.05E+01 | 1.67E+01 | 1.08E+01 | 1.06E+01 | 9.20E+00 | 20.5 |  |  | x |
| 136.1131 | 0.22 | 2.86E+02 | 2.97E+02 | 2.94E+02 | 3.28E+02 | 3.16E+02 | 4.18E+02 | 3.43E+02 | 3.61E+02 | 3.65E+02 | 3.47E+02 | 3.84E+02 | 4.64E+02 | 4.77E+02 | 4.77E+02 | 4.68E+02 | 5.35E+02 | 4.74E+02 | 20.2 |  |  | x |
| 194.1913 | 0.22 | 8.82E+01 | 7.93E+01 | 7.29E+01 | 1.00E+02 | 9.15E+01 | 1.15E+02 | 1.04E+02 | 1.15E+02 | 1.07E+02 | 9.04E+01 | 1.14E+02 | 1.41E+02 | 1.48E+02 | 1.70E+02 | 1.43E+02 | 1.39E+02 | 1.54E+02 | 24.5 |  |  | x |
| 187.0954 | 0.22 | 2.07E+01 | 2.11E+01 | 1.71E+01 | 2.14E+01 | 2.50E+01 | 1.75E+01 | 2.27E+01 | 2.68E+01 | 1.97E+01 | 2.66E+01 | 2.40E+01 | 2.47E+01 | 1.92E+01 | 1.92E+01 | 1.65E+01 | 1.62E+01 | 1.92E+01 | 16.3 |  | x | x |
| 215.1268 | 0.22 | 1.64E+02 | 1.52E+02 | 1.44E+02 | 1.87E+02 | 2.01E+02 | 1.49E+02 | 1.77E+02 | 2.15E+02 | 1.97E+02 | 1.82E+02 | 1.91E+02 | 1.87E+02 | 1.65E+02 | 1.66E+02 | 1.54E+02 | 1.58E+02 | 1.72E+02 | 11.6 | x | x | x |
| 301.0973 | 0.22 | 9.72E+01 | 8.77E+01 | 9.66E+01 | 9.85E+01 | 9.99E+01 | 3.70E+01 | 1.07E+02 | 1.01E+02 | 1.01E+02 | 7.88E+01 | 9.68E+01 | 9.93E+01 | 7.63E+01 | 7.69E+01 | 5.68E+01 | 8.26E+01 | 7.14E+01 | 21.5 |  |  | x |
| 302.1531 | 0.22 | 2.00E+01 | 2.97E+01 | 2.29E+01 | 1.83E+01 | 3.63E+01 | 1.17E+01 | 2.11E+01 | 2.76E+01 | 2.20E+01 | 1.99E+01 | 2.18E+01 | 2.37E+01 | 1.68E+01 | 1.75E+01 | 1.88E+01 | 1.91E+01 | 1.42E+01 | 27.4 |  |  | x |
| 411.2477 | 0.22 | 1.25E+02 | 1.28E+02 | 1.40E+02 | 1.22E+02 | 1.32E+02 | 1.03E+02 | 1.39E+02 | 1.41E+02 | 1.56E+02 | 1.03E+02 | 1.31E+02 | 1.18E+02 | 1.13E+02 | 1.12E+02 | 1.05E+02 | 1.23E+02 | 1.02E+02 | 12.7 | x | x | x |
| 128.0523 | 0.23 | 6.15E+01 | 6.39E+01 | 5.75E+01 | 6.22E+01 | 6.14E+01 | 4.99E+01 | 6.33E+01 | 6.84E+01 | 6.20E+01 | 5.54E+01 | 7.00E+01 | 5.35E+01 | 5.38E+01 | 4.73E+01 | 6.11E+01 | 6.18E+01 | 5.22E+01 | 10.7 | x | x | x |
| 122.0974 | 0.23 | 1.15E+03 | 1.22E+03 | 1.16E+03 | 1.37E+03 | 1.49E+03 | 1.95E+03 | 1.46E+03 | 1.57E+03 | 1.54E+03 | 1.63E+03 | 1.72E+03 | 1.87E+03 | 1.69E+03 | 1.65E+03 | 1.73E+03 | 1.76E+03 | 1.84E+03 | 15.4 |  | x | x |
| 161.0736 | 0.23 | 6.99E+01 | 7.01E+01 | 7.46E+01 | 7.57E+01 | 7.24E+01 | 5.37E+01 | 8.30E+01 | 8.52E+01 | 7.81E+01 | 6.60E+01 | 6.64E+01 | 6.38E+01 | 4.95E+01 | 6.16E+01 | 4.52E+01 | 5.84E+01 | 4.72E+01 | 18.3 |  | x | x |
| 219.0798 | 0.23 | 1.61E+02 | 1.51E+02 | 1.40E+02 | 1.63E+02 | 1.68E+02 | 1.58E+02 | 1.91E+02 | 1.79E+02 | 1.78E+02 | 1.47E+02 | 1.72E+02 | 1.49E+02 | 1.37E+02 | 1.60E+02 | 1.09E+02 | 1.31E+02 | 1.24E+02 | 13.9 | x | x | x |
| 241.1063 | 0.23 | 2.18E+02 | 2.13E+02 | 2.00E+02 | 2.13E+02 | 2.14E+02 | 1.10E+02 | 2.22E+02 | 2.33E+02 | 2.17E+02 | 1.73E+02 | 2.10E+02 | 1.72E+02 | 1.20E+02 | 1.10E+02 | 1.28E+02 | 1.08E+02 | 9.64E+01 | 28.7 |  |  | x |
| 244.1350 | 0.23 | 9.96E+02 | 9.87E+02 | 9.59E+02 | 1.12E+03 | 1.17E+03 | 1.29E+03 | 1.22E+03 | 1.27E+03 | 1.28E+03 | 1.22E+03 | 1.32E+03 | 1.32E+03 | 1.52E+03 | 1.45E+03 | 1.33E+03 | 1.53E+03 | 1.39E+03 | 13.6 | x | x | x |
| 249.1140 | 0.23 | 1.04E+02 | 1.01E+02 | 8.88E+01 | 9.93E+01 | 9.97E+01 | 5.82E+01 | 1.09E+02 | 9.42E+01 | 1.05E+02 | 8.97E+01 | 9.09E+01 | 9.00E+01 | 6.50E+01 | 6.23E+01 | 6.47E+01 | 8.17E+01 | 4.52E+01 | 22.4 |  |  | x |
| 315.0890 | 0.23 | 7.26E+01 | 6.30E+01 | 6.95E+01 | 6.72E+01 | 6.73E+01 | 5.35E+01 | 8.15E+01 | 7.62E+01 | 7.65E+01 | 5.66E+01 | 5.93E+01 | 7.51E+01 | 5.77E+01 | 7.88E+01 | 7.29E+01 | 8.44E+01 | 7.38E+01 | 13.1 | x | x | x |
| 330.9619 | 0.23 | 1.41E+02 | 1.26E+02 | 1.23E+02 | 1.32E+02 | 1.35E+02 | 1.01E+02 | 1.36E+02 | 1.32E+02 | 1.32E+02 | 1.23E+02 | 1.25E+02 | 1.47E+02 | 1.29E+02 | 1.46E+02 | 1.48E+02 | 1.29E+02 | 1.41E+02 | 8.7 | x | x | x |
| 332.9592 | 0.23 | 8.11E+01 | 7.76E+01 | 6.51E+01 | 7.80E+01 | 8.37E+01 | 5.49E+01 | 7.83E+01 | 7.46E+01 | 7.90E+01 | 6.08E+01 | 7.53E+01 | 8.89E+01 | 7.05E+01 | 7.68E+01 | 7.22E+01 | 8.04E+01 | 7.50E+01 | 11.1 | x | x | x |
| 335.2173 | 0.23 | 1.03E+02 | 1.04E+02 | 1.04E+02 | 9.94E+01 | 1.14E+02 | 8.06E+01 | 1.21E+02 | 1.27E+02 | 1.24E+02 | 1.07E+02 | 1.11E+02 | 1.01E+02 | 9.20E+01 | 9.21E+01 | 9.34E+01 | 7.99E+01 | 8.61E+01 | 13.8 | x | x | x |
| 323.1783 | 0.23 | 1.77E+02 | 1.83E+02 | 1.76E+02 | 1.73E+02 | 1.84E+02 | 1.20E+02 | 1.98E+02 | 2.16E+02 | 2.05E+02 | 1.72E+02 | 1.87E+02 | 1.85E+02 | 1.55E+02 | 1.59E+02 | 1.40E+02 | 1.60E+02 | 1.40E+02 | 14.4 | x | x | x |
| 351.2051 | 0.23 | 6.56E+01 | 8.44E+01 | 7.65E+01 | 6.96E+01 | 8.73E+01 | 6.32E+01 | 8.35E+01 | 7.78E+01 | 7.37E+01 | 7.07E+01 | 8.06E+01 | 8.56E+01 | 8.01E+01 | 8.13E+01 | 5.97E+01 | 8.61E+01 | 6.97E+01 | 11.2 | x | x | x |
| 357.1675 | 0.23 | 2.77E+01 | 3.07E+01 | 3.04E+01 | 2.03E+01 | 3.61E+01 | 2.85E+01 | 4.01E+01 | 3.74E+01 | 4.12E+01 | 2.88E+01 | 3.30E+01 | 3.34E+01 | 2.56E+01 | 2.90E+01 | 2.42E+01 | 2.77E+01 | 2.16E+01 | 19.7 |  | x | x |
| 333.1880 | 0.23 | 4.70E+01 | 5.50E+01 | 4.53E+01 | 4.24E+01 | 6.23E+01 | 3.53E+01 | 5.70E+01 | 5.59E+01 | 5.97E+01 | 3.98E+01 | 4.52E+01 | 5.14E+01 | 4.21E+01 | 2.53E+01 | 2.35E+01 | 3.30E+01 | 4.18E+01 | 25.4 |  |  | x |
| 381.2567 | 0.23 | 5.49E+01 | 5.81E+01 | 4.67E+01 | 5.21E+01 | 6.04E+01 | 3.52E+01 | 6.49E+01 | 6.97E+01 | 6.02E+01 | 5.46E+01 | 6.22E+01 | 5.89E+01 | 4.31E+01 | 4.70E+01 | 3.42E+01 | 4.26E+01 | 4.89E+01 | 19.3 |  | x | x |
| 395.2362 | 0.23 | 1.50E+02 | 1.65E+02 | 1.53E+02 | 1.45E+02 | 1.76E+02 | 1.16E+02 | 1.74E+02 | 1.86E+02 | 1.73E+02 | 1.57E+02 | 1.88E+02 | 1.84E+02 | 1.56E+02 | 1.41E+02 | 1.29E+02 | 1.46E+02 | 1.37E+02 | 13.2 | x | x | x |
| 393.2202 | 0.23 | 1.20E+02 | 1.29E+02 | 1.29E+02 | 1.38E+02 | 1.41E+02 | 1.00E+02 | 1.39E+02 | 1.46E+02 | 1.49E+02 | 1.27E+02 | 1.40E+02 | 1.37E+02 | 1.13E+02 | 1.19E+02 | 1.01E+02 | 1.06E+02 | 1.12E+02 | 12.4 | x | x | x |
| 205.1184 | 0.24 | 1.05E+02 | 9.53E+01 | 1.05E+02 | 9.81E+01 | 1.10E+02 | 6.88E+01 | 1.19E+02 | 1.17E+02 | 1.24E+02 | 9.75E+01 | 1.21E+02 | 9.39E+01 | 8.49E+01 | 7.82E+01 | 7.04E+01 | 7.82E+01 | 6.66E+01 | 19.7 |  | x | x |
| 120.0458 | 0.24 | 2.69E+01 | 2.78E+01 | 2.84E+01 | 2.37E+01 | 2.65E+01 | 2.61E+01 | 3.43E+01 | 3.33E+01 | 2.86E+01 | 1.85E+01 | 3.62E+01 | 2.47E+01 | 2.71E+01 | 2.55E+01 | 2.11E+01 | 2.97E+01 | 2.79E+01 | 16.2 |  | x | x |
| 134.0978 | 0.24 | 1.79E+01 | 2.32E+01 | 2.40E+01 | 2.04E+01 | 1.42E+01 | 1.05E+01 | 1.90E+01 | 1.79E+01 | 2.06E+01 | 1.90E+01 | 1.95E+01 | 1.53E+01 | 2.04E+01 | 2.88E+01 | 2.20E+01 | 1.22E+01 | 1.68E+01 | 23.5 |  |  | x |
| 144.0490 | 0.24 | 1.77E+01 | 1.92E+01 | 1.99E+01 | 1.96E+01 | 1.90E+01 | 1.77E+01 | 2.24E+01 | 2.19E+01 | 2.04E+01 | 1.98E+01 | 1.79E+01 | 2.16E+01 | 2.28E+01 | 2.28E+01 | 1.54E+01 | 2.04E+01 | 2.23E+01 | 10.5 | x | x | x |
| 149.1048 | 0.24 | 5.40E+01 | 5.26E+01 | 5.64E+01 | 5.14E+01 | 5.79E+01 | 4.45E+01 | 5.56E+01 | 5.91E+01 | 5.69E+01 | 5.16E+01 | 5.91E+01 | 5.85E+01 | 5.10E+01 | 4.79E+01 | 4.63E+01 | 4.85E+01 | 3.66E+01 | 11.7 | x | x | x |
| 114.0924 | 0.24 | 1.06E+02 | 9.82E+01 | 1.20E+02 | 1.19E+02 | 1.24E+02 | 1.05E+02 | 1.25E+02 | 1.34E+02 | 1.39E+02 | 1.34E+02 | 1.31E+02 | 1.36E+02 | 1.04E+02 | 1.26E+02 | 1.07E+02 | 1.09E+02 | 1.17E+02 | 10.7 | x | x | x |
| 156.0839 | 0.24 | 1.59E+02 | 1.63E+02 | 1.77E+02 | 1.49E+02 | 1.70E+02 | 1.17E+02 | 1.69E+02 | 1.93E+02 | 1.73E+02 | 1.65E+02 | 1.81E+02 | 1.83E+02 | 1.47E+02 | 1.62E+02 | 1.57E+02 | 1.71E+02 | 2.04E+02 | 11.7 | x | x | x |
| 181.0994 | 0.24 | 4.42E+01 | 6.42E+01 | 6.09E+01 | 6.13E+01 | 7.43E+01 | 3.92E+01 | 6.39E+01 | 7.65E+01 | 6.92E+01 | 7.29E+01 | 6.27E+01 | 6.95E+01 | 5.89E+01 | 6.56E+01 | 7.02E+01 | 6.69E+01 | 7.11E+01 | 15.3 |  | x | x |
| 170.1110 | 0.24 | 4.63E+01 | 4.29E+01 | 4.37E+01 | 4.06E+01 | 4.13E+01 | 3.27E+01 | 4.66E+01 | 4.24E+01 | 3.55E+01 | 3.14E+01 | 3.36E+01 | 3.17E+01 | 2.85E+01 | 3.25E+01 | 2.63E+01 | 2.83E+01 | 3.60E+01 | 18.0 |  | x | x |
| 199.0831 | 0.24 | 6.15E+02 | 6.81E+02 | 6.64E+02 | 6.56E+02 | 7.38E+02 | 5.22E+02 | 7.50E+02 | 7.74E+02 | 7.64E+02 | 7.86E+02 | 7.62E+02 | 8.29E+02 | 7.42E+02 | 7.09E+02 | 6.98E+02 | 8.11E+02 | 8.15E+02 | 11.0 | x | x | x |
| 200.0870 | 0.24 | 7.51E+01 | 8.13E+01 | 8.86E+01 | 7.60E+01 | 8.66E+01 | 5.39E+01 | 9.18E+01 | 9.87E+01 | 9.11E+01 | 8.00E+01 | 8.38E+01 | 1.02E+02 | 7.66E+01 | 9.08E+01 | 7.46E+01 | 7.76E+01 | 7.79E+01 | 13.5 | x | x | x |
| 186.1136 | 0.24 | 3.27E+02 | 3.32E+02 | 3.31E+02 | 3.66E+02 | 4.01E+02 | 3.27E+02 | 4.39E+02 | 3.92E+02 | 4.02E+02 | 3.90E+02 | 4.75E+02 | 4.92E+02 | 3.66E+02 | 4.21E+02 | 2.99E+02 | 3.76E+02 | 3.65E+02 | 13.9 | x | x | x |
| 195.1162 | 0.24 | 2.46E+02 | 2.32E+02 | 2.37E+02 | 2.36E+02 | 2.64E+02 | 1.74E+02 | 2.71E+02 | 2.57E+02 | 2.32E+02 | 2.25E+02 | 2.52E+02 | 2.23E+02 | 2.03E+02 | 2.03E+02 | 1.77E+02 | 1.99E+02 | 1.96E+02 | 12.9 | x | x | x |
| 225.1119 | 0.24 | 1.86E+02 | 1.89E+02 | 1.91E+02 | 1.90E+02 | 2.06E+02 | 1.41E+02 | 2.07E+02 | 2.06E+02 | 2.11E+02 | 1.86E+02 | 2.02E+02 | 2.04E+02 | 1.70E+02 | 1.67E+02 | 1.60E+02 | 1.60E+02 | 1.45E+02 | 12.3 | x | x | x |
| 231.0948 | 0.24 | 2.48E+02 | 2.79E+02 | 2.85E+02 | 2.75E+02 | 3.19E+02 | 2.01E+02 | 3.47E+02 | 3.45E+02 | 3.16E+02 | 2.74E+02 | 3.11E+02 | 3.10E+02 | 2.62E+02 | 2.79E+02 | 2.45E+02 | 2.71E+02 | 2.65E+02 | 13.1 | x | x | x |
| 307.1354 | 0.24 | 5.30E+02 | 5.71E+02 | 5.38E+02 | 5.45E+02 | 6.51E+02 | 4.81E+02 | 6.43E+02 | 7.32E+02 | 7.01E+02 | 7.12E+02 | 7.20E+02 | 7.55E+02 | 6.15E+02 | 6.28E+02 | 5.45E+02 | 5.66E+02 | 6.11E+02 | 13.2 | x | x | x |
| 309.0911 | 0.24 | 3.93E+02 | 4.67E+02 | 4.53E+02 | 4.11E+02 | 5.26E+02 | 4.19E+02 | 5.32E+02 | 5.69E+02 | 5.76E+02 | 5.39E+02 | 6.01E+02 | 6.23E+02 | 4.99E+02 | 4.68E+02 | 4.68E+02 | 4.89E+02 | 4.76E+02 | 13.3 | x | x | x |
| 271.0621 | 0.24 | 1.90E+02 | 1.78E+02 | 1.46E+02 | 1.93E+02 | 2.00E+02 | 1.68E+02 | 2.03E+02 | 1.93E+02 | 1.99E+02 | 1.70E+02 | 2.01E+02 | 1.87E+02 | 1.88E+02 | 2.32E+02 | 1.48E+02 | 1.75E+02 | 1.44E+02 | 12.6 | x | x | x |
| 275.1203 | 0.24 | 8.05E+01 | 9.01E+01 | 9.88E+01 | 1.14E+02 | 1.10E+02 | 9.26E+01 | 1.21E+02 | 1.36E+02 | 1.29E+02 | 1.09E+02 | 1.22E+02 | 1.32E+02 | 1.16E+02 | 1.13E+02 | 1.01E+02 | 1.28E+02 | 1.23E+02 | 14.0 | x | x | x |
| 285.0780 | 0.24 | 1.44E+04 | 1.42E+04 | 1.37E+04 | 1.52E+04 | 1.73E+04 | 2.06E+04 | 1.79E+04 | 1.90E+04 | 1.89E+04 | 2.00E+04 | 2.11E+04 | 2.25E+04 | 2.02E+04 | 2.17E+04 | 2.01E+04 | 2.10E+04 | 2.06E+04 | 14.9 | x | x | x |
| 321.1633 | 0.24 | 1.40E+02 | 1.48E+02 | 1.38E+02 | 1.30E+02 | 1.49E+02 | 8.73E+01 | 1.59E+02 | 1.61E+02 | 1.63E+02 | 1.24E+02 | 1.44E+02 | 1.52E+02 | 1.13E+02 | 1.15E+02 | 7.66E+01 | 9.14E+01 | 1.01E+02 | 21.2 |  |  | x |
| 295.1519 | 0.24 | 1.43E+02 | 1.64E+02 | 1.74E+02 | 1.44E+02 | 1.72E+02 | 1.02E+02 | 1.73E+02 | 1.86E+02 | 1.64E+02 | 1.47E+02 | 1.67E+02 | 1.74E+02 | 1.48E+02 | 1.47E+02 | 1.10E+02 | 1.34E+02 | 1.38E+02 | 15.2 |  | x | x |
| 298.0056 | 0.24 | 5.93E+02 | 9.59E+02 | 8.67E+02 | 8.38E+02 | 1.20E+03 | 6.20E+02 | 1.18E+03 | 1.49E+03 | 1.52E+03 | 1.60E+03 | 1.64E+03 | 1.83E+03 | 1.62E+03 | 1.51E+03 | 1.56E+03 | 1.59E+03 | 1.55E+03 | 29.9 |  |  | x |
| 340.1324 | 0.24 | 2.66E+01 | 3.80E+01 | 3.97E+01 | 2.52E+01 | 3.14E+01 | 2.61E+01 | 3.10E+01 | 2.13E+01 | 4.71E+01 | 2.54E+01 | 3.47E+01 | 3.13E+01 | 4.07E+01 | 2.71E+01 | 1.72E+01 | 2.18E+01 | 1.48E+01 | 29.5 |  |  | x |
| 367.1914 | 0.24 | 4.62E+01 | 4.64E+01 | 4.77E+01 | 4.69E+01 | 5.30E+01 | 3.46E+01 | 4.19E+01 | 5.31E+01 | 5.07E+01 | 3.69E+01 | 4.07E+01 | 4.71E+01 | 2.63E+01 | 3.99E+01 | 2.83E+01 | 2.44E+01 | 2.80E+01 | 23.3 |  |  | x |
| 430.0212 | 0.24 | 5.19E+02 | 5.70E+02 | 5.98E+02 | 5.49E+02 | 6.55E+02 | 4.59E+02 | 6.74E+02 | 6.90E+02 | 6.82E+02 | 6.67E+02 | 6.99E+02 | 8.06E+02 | 6.55E+02 | 6.96E+02 | 7.09E+02 | 7.11E+02 | 7.47E+02 | 13.4 | x | x | x |
| 397.2505 | 0.24 | 1.01E+02 | 1.25E+02 | 1.05E+02 | 1.06E+02 | 1.27E+02 | 8.43E+01 | 1.28E+02 | 1.35E+02 | 1.36E+02 | 1.07E+02 | 1.32E+02 | 1.44E+02 | 1.47E+02 | 1.28E+02 | 1.39E+02 | 1.32E+02 | 1.47E+02 | 14.4 | x | x | x |
| 432.0254 | 0.24 | 4.73E+01 | 4.86E+01 | 4.65E+01 | 4.10E+01 | 4.79E+01 | 2.88E+01 | 4.66E+01 | 4.84E+01 | 4.61E+01 | 4.53E+01 | 5.10E+01 | 5.21E+01 | 4.51E+01 | 4.40E+01 | 4.03E+01 | 3.70E+01 | 4.50E+01 | 12.4 | x | x | x |
| 428.0148 | 0.24 | 8.63E+02 | 9.35E+02 | 9.90E+02 | 9.12E+02 | 1.06E+03 | 7.91E+02 | 1.12E+03 | 1.11E+03 | 1.14E+03 | 1.10E+03 | 1.19E+03 | 1.35E+03 | 1.14E+03 | 1.16E+03 | 1.26E+03 | 1.25E+03 | 1.27E+03 | 14.0 | x | x | x |
| 205.0637 | 0.25 | 2.00E+02 | 2.08E+02 | 2.05E+02 | 2.14E+02 | 2.06E+02 | 1.02E+02 | 2.31E+02 | 2.12E+02 | 2.08E+02 | 1.91E+02 | 2.02E+02 | 2.31E+02 | 1.76E+02 | 1.98E+02 | 1.72E+02 | 2.07E+02 | 1.98E+02 | 14.7 | x | x | x |
| 116.1079 | 0.25 | 1.07E+03 | 9.93E+02 | 1.02E+03 | 1.07E+03 | 1.11E+03 | 1.34E+03 | 1.15E+03 | 1.22E+03 | 1.12E+03 | 1.17E+03 | 1.23E+03 | 1.16E+03 | 9.41E+02 | 8.98E+02 | 8.69E+02 | 9.93E+02 | 9.91E+02 | 11.8 | x | x | x |
| 164.0726 | 0.25 | 1.07E+02 | 1.05E+02 | 1.17E+02 | 1.09E+02 | 1.16E+02 | 9.47E+01 | 1.19E+02 | 1.19E+02 | 1.15E+02 | 1.17E+02 | 1.21E+02 | 1.27E+02 | 1.22E+02 | 1.04E+02 | 9.88E+01 | 1.13E+02 | 1.08E+02 | 7.7 | x | x | x |
| 203.0839 | 0.25 | 6.74E+01 | 6.72E+01 | 6.41E+01 | 6.15E+01 | 7.71E+01 | 3.81E+01 | 8.20E+01 | 7.72E+01 | 7.00E+01 | 6.83E+01 | 6.23E+01 | 7.39E+01 | 6.27E+01 | 5.74E+01 | 5.54E+01 | 5.35E+01 | 5.86E+01 | 16.3 |  | x | x |
| 255.2039 | 0.25 | 1.24E+02 | 9.93E+01 | 9.70E+01 | 1.16E+02 | 1.12E+02 | 3.19E+01 | 1.24E+02 | 1.22E+02 | 1.26E+02 | 8.08E+01 | 1.38E+02 | 1.10E+02 | 9.14E+01 | 8.78E+01 | 6.44E+01 | 9.96E+01 | 5.24E+01 | 28.9 |  |  | x |
| 255.0675 | 0.25 | 3.36E+04 | 3.18E+04 | 3.26E+04 | 3.63E+04 | 3.65E+04 | 4.06E+04 | 3.80E+04 | 3.95E+04 | 3.76E+04 | 3.71E+04 | 4.15E+04 | 4.41E+04 | 4.61E+04 | 4.27E+04 | 4.46E+04 | 4.76E+04 | 4.62E+04 | 12.4 | x | x | x |
| 257.0946 | 0.25 | 1.96E+02 | 1.93E+02 | 1.93E+02 | 1.95E+02 | 2.09E+02 | 1.24E+02 | 2.18E+02 | 2.20E+02 | 2.08E+02 | 1.73E+02 | 2.03E+02 | 2.06E+02 | 1.93E+02 | 1.80E+02 | 1.74E+02 | 1.90E+02 | 1.96E+02 | 11.5 | x | x | x |
| 139.1236 | 0.26 | 4.79E+01 | 4.74E+01 | 4.45E+01 | 3.93E+01 | 4.29E+01 | 2.84E+01 | 4.76E+01 | 4.57E+01 | 4.07E+01 | 3.51E+01 | 4.34E+01 | 3.66E+01 | 3.69E+01 | 3.81E+01 | 2.92E+01 | 3.15E+01 | 3.36E+01 | 16.2 |  | x | x |
| 154.1351 | 0.26 | 1.98E+01 | 2.33E+01 | 2.32E+01 | 2.38E+01 | 2.30E+01 | 1.69E+01 | 2.09E+01 | 2.47E+01 | 1.80E+01 | 1.86E+01 | 2.03E+01 | 2.63E+01 | 2.19E+01 | 1.83E+01 | 1.61E+01 | 1.63E+01 | 1.72E+01 | 15.5 |  | x | x |
| 158.0283 | 0.26 | 2.23E+03 | 2.03E+03 | 2.11E+03 | 2.24E+03 | 2.25E+03 | 2.98E+03 | 2.52E+03 | 2.53E+03 | 2.44E+03 | 2.48E+03 | 2.75E+03 | 3.02E+03 | 3.02E+03 | 2.94E+03 | 3.01E+03 | 3.35E+03 | 3.32E+03 | 15.8 |  | x | x |
| 167.1182 | 0.26 | 1.86E+02 | 1.61E+02 | 1.82E+02 | 1.80E+02 | 1.74E+02 | 1.54E+02 | 1.91E+02 | 1.92E+02 | 1.86E+02 | 1.72E+02 | 1.88E+02 | 2.14E+02 | 1.99E+02 | 2.25E+02 | 1.69E+02 | 2.22E+02 | 2.00E+02 | 10.5 | x | x | x |
| 350.2337 | 0.26 | 2.71E+02 | 3.50E+02 | 3.20E+02 | 3.25E+02 | 3.63E+02 | 7.11E+01 | 3.59E+02 | 4.06E+02 | 4.00E+02 | 3.39E+02 | 3.56E+02 | 4.21E+02 | 4.60E+02 | 3.89E+02 | 4.17E+02 | 4.80E+02 | 5.08E+02 | 26.6 |  |  | x |
| 118.1232 | 0.27 | 1.55E+01 | 1.06E+01 | 1.29E+01 | 1.41E+01 | 1.04E+01 | 1.06E+01 | 1.40E+01 | 1.08E+01 | 1.55E+01 | 1.32E+01 | 1.63E+01 | 1.71E+01 | 1.29E+01 | 1.45E+01 | 8.97E+00 | 1.65E+01 | 1.36E+01 | 18.1 |  | x | x |
| 166.1205 | 0.27 | 9.12E+01 | 8.13E+01 | 8.16E+01 | 9.05E+01 | 9.27E+01 | 4.13E+01 | 8.95E+01 | 7.95E+01 | 9.03E+01 | 7.41E+01 | 8.50E+01 | 7.73E+01 | 6.47E+01 | 6.02E+01 | 5.75E+01 | 9.20E+01 | 5.44E+01 | 20.4 |  |  | x |
| 181.0631 | 0.27 | 5.63E+02 | 5.38E+02 | 5.69E+02 | 5.10E+02 | 5.38E+02 | 5.43E+02 | 5.73E+02 | 5.67E+02 | 5.26E+02 | 5.22E+02 | 5.50E+02 | 5.87E+02 | 5.49E+02 | 5.42E+02 | 5.44E+02 | 6.05E+02 | 6.21E+02 | 5.3 | x | x | x |
| 310.2025 | 0.27 | 1.12E+02 | 1.12E+02 | 1.20E+02 | 1.09E+02 | 1.15E+02 | 1.07E+02 | 1.12E+02 | 1.20E+02 | 1.34E+02 | 1.15E+02 | 1.28E+02 | 1.36E+02 | 1.29E+02 | 1.04E+02 | 8.50E+01 | 1.05E+02 | 1.04E+02 | 11.1 | x | x | x |
| 312.2178 | 0.27 | 9.17E+01 | 1.21E+02 | 1.19E+02 | 1.20E+02 | 1.41E+02 | 3.84E+01 | 1.24E+02 | 1.35E+02 | 1.39E+02 | 1.14E+02 | 1.21E+02 | 1.44E+02 | 1.74E+02 | 1.08E+02 | 1.18E+02 | 1.37E+02 | 1.64E+02 | 24.0 |  |  | x |
| 353.2304 | 0.27 | 4.41E+01 | 3.53E+01 | 3.79E+01 | 3.95E+01 | 3.19E+01 | 1.81E+01 | 4.97E+01 | 4.42E+01 | 3.52E+01 | 2.97E+01 | 4.11E+01 | 4.01E+01 | 2.33E+01 | 3.80E+01 | 4.00E+01 | 4.56E+01 | 4.05E+01 | 21.4 |  |  | x |
| 108.0809 | 0.28 | 3.35E+01 | 3.21E+01 | 3.49E+01 | 3.63E+01 | 2.84E+01 | 2.91E+01 | 3.02E+01 | 4.12E+01 | 3.33E+01 | 3.10E+01 | 3.05E+01 | 4.08E+01 | 2.85E+01 | 3.11E+01 | 1.78E+01 | 2.29E+01 | 2.08E+01 | 20.2 |  |  | x |
| 146.0612 | 0.28 | 1.20E+02 | 1.07E+02 | 1.13E+02 | 1.00E+02 | 1.16E+02 | 1.29E+02 | 1.16E+02 | 1.16E+02 | 1.12E+02 | 9.92E+01 | 1.14E+02 | 1.16E+02 | 1.09E+02 | 1.08E+02 | 1.03E+02 | 1.07E+02 | 1.11E+02 | 6.7 | x | x | x |
| 217.1059 | 0.28 | 1.07E+03 | 1.12E+03 | 1.09E+03 | 1.22E+03 | 1.41E+03 | 1.29E+03 | 1.41E+03 | 1.56E+03 | 1.49E+03 | 1.46E+03 | 1.61E+03 | 1.62E+03 | 1.53E+03 | 1.47E+03 | 1.36E+03 | 1.49E+03 | 1.41E+03 | 12.6 | x | x | x |
| 118.0662 | 0.29 | 8.27E+02 | 7.88E+02 | 8.02E+02 | 8.21E+02 | 8.46E+02 | 9.42E+02 | 8.99E+02 | 8.81E+02 | 8.40E+02 | 8.30E+02 | 8.97E+02 | 9.42E+02 | 9.14E+02 | 9.63E+02 | 9.53E+02 | 1.03E+03 | 1.00E+03 | 8.1 | x | x | x |
| 305.1587 | 0.29 | 1.60E+02 | 1.72E+02 | 1.72E+02 | 1.96E+02 | 1.98E+02 | 1.93E+02 | 2.15E+02 | 2.35E+02 | 2.35E+02 | 2.18E+02 | 2.44E+02 | 3.08E+02 | 2.65E+02 | 2.33E+02 | 2.38E+02 | 2.85E+02 | 2.98E+02 | 19.3 |  | x | x |
| 77.0393 | 0.30 | 2.61E+01 | 3.74E+01 | 3.87E+01 | 3.64E+01 | 3.08E+01 | 9.19E+00 | 3.30E+01 | 3.11E+01 | 2.93E+01 | 2.78E+01 | 2.94E+01 | 3.02E+01 | 2.79E+01 | 3.42E+01 | 3.17E+01 | 2.75E+01 | 2.50E+01 | 22.1 |  |  | x |
| 105.0344 | 0.30 | 1.90E+02 | 1.68E+02 | 1.57E+02 | 1.56E+02 | 1.60E+02 | 2.00E+02 | 1.75E+02 | 1.77E+02 | 1.72E+02 | 1.60E+02 | 1.87E+02 | 1.80E+02 | 1.56E+02 | 1.86E+02 | 1.68E+02 | 1.83E+02 | 1.86E+02 | 7.8 | x | x | x |
| 133.0873 | 0.30 | 1.70E+02 | 1.67E+02 | 1.64E+02 | 1.70E+02 | 1.93E+02 | 1.07E+02 | 2.05E+02 | 2.05E+02 | 1.82E+02 | 1.63E+02 | 1.83E+02 | 1.99E+02 | 1.97E+02 | 1.87E+02 | 1.80E+02 | 2.11E+02 | 2.03E+02 | 13.6 | x | x | x |
| 148.1339 | 0.30 | 4.18E+02 | 3.57E+02 | 3.72E+02 | 3.81E+02 | 3.93E+02 | 4.85E+02 | 4.18E+02 | 4.14E+02 | 4.05E+02 | 3.85E+02 | 4.49E+02 | 4.64E+02 | 4.91E+02 | 4.41E+02 | 4.38E+02 | 4.95E+02 | 4.66E+02 | 10.1 | x | x | x |
| 327.2035 | 0.31 | 2.47E+02 | 2.03E+02 | 2.21E+02 | 2.24E+02 | 2.28E+02 | 2.36E+02 | 2.46E+02 | 2.34E+02 | 2.25E+02 | 1.94E+02 | 2.10E+02 | 2.44E+02 | 2.00E+02 | 2.08E+02 | 2.27E+02 | 2.34E+02 | 2.07E+02 | 7.4 | x | x | x |
| 371.2294 | 0.31 | 1.97E+02 | 1.71E+02 | 1.89E+02 | 1.87E+02 | 1.70E+02 | 2.00E+02 | 1.96E+02 | 1.88E+02 | 1.74E+02 | 1.69E+02 | 1.77E+02 | 2.06E+02 | 1.53E+02 | 1.77E+02 | 1.55E+02 | 1.78E+02 | 1.73E+02 | 8.3 | x | x | x |
| 432.2826 | 0.31 | 8.88E+01 | 7.21E+01 | 7.72E+01 | 7.87E+01 | 7.09E+01 | 8.23E+01 | 9.46E+01 | 8.26E+01 | 6.75E+01 | 6.78E+01 | 7.06E+01 | 9.03E+01 | 6.70E+01 | 7.27E+01 | 7.44E+01 | 8.40E+01 | 8.02E+01 | 10.9 | x | x | x |
| 104.1076 | 0.32 | 9.74E+03 | 9.71E+03 | 9.93E+03 | 1.04E+04 | 1.07E+04 | 1.36E+04 | 1.11E+04 | 1.14E+04 | 1.09E+04 | 1.23E+04 | 1.20E+04 | 1.28E+04 | 1.26E+04 | 1.24E+04 | 1.24E+04 | 1.32E+04 | 1.31E+04 | 10.9 | x | x | x |
| 118.1234 | 0.32 | 9.14E+01 | 8.37E+01 | 8.92E+01 | 8.39E+01 | 8.45E+01 | 1.08E+02 | 9.19E+01 | 9.14E+01 | 9.03E+01 | 8.27E+01 | 9.11E+01 | 9.57E+01 | 8.30E+01 | 9.55E+01 | 8.65E+01 | 1.05E+02 | 8.88E+01 | 7.9 | x | x | x |
| 415.2558 | 0.32 | 4.96E+01 | 4.58E+01 | 4.80E+01 | 4.68E+01 | 3.27E+01 | 3.37E+01 | 4.71E+01 | 4.91E+01 | 4.76E+01 | 2.20E+01 | 4.43E+01 | 3.51E+01 | 3.11E+01 | 3.00E+01 | 3.83E+01 | 3.64E+01 | 2.38E+01 | 23.4 |  |  | x |
| 476.3089 | 0.32 | 5.76E+01 | 5.71E+01 | 5.35E+01 | 5.91E+01 | 5.54E+01 | 5.25E+01 | 5.15E+01 | 5.53E+01 | 4.94E+01 | 4.21E+01 | 5.20E+01 | 6.17E+01 | 6.29E+01 | 4.23E+01 | 3.89E+01 | 4.91E+01 | 2.55E+01 | 18.4 |  | x | x |
| 174.0566 | 0.33 | 2.31E+02 | 1.99E+02 | 2.05E+02 | 2.28E+02 | 2.35E+02 | 3.08E+02 | 2.79E+02 | 2.67E+02 | 2.62E+02 | 2.51E+02 | 2.93E+02 | 3.17E+02 | 3.08E+02 | 3.28E+02 | 3.03E+02 | 3.52E+02 | 3.45E+02 | 17.1 |  | x | x |
| 218.1377 | 0.33 | 4.00E+01 | 3.81E+01 | 3.81E+01 | 3.02E+01 | 3.31E+01 | 2.24E+01 | 3.73E+01 | 3.69E+01 | 2.90E+01 | 2.39E+01 | 4.69E+01 | 2.59E+01 | 2.90E+01 | 2.15E+01 | 2.68E+01 | 3.54E+01 | 2.79E+01 | 22.1 |  |  | x |
| 176.0738 | 0.34 | 2.75E+01 | 2.40E+01 | 2.70E+01 | 2.85E+01 | 2.71E+01 | 1.13E+01 | 3.00E+01 | 2.61E+01 | 3.18E+01 | 1.76E+01 | 2.82E+01 | 2.21E+01 | 2.46E+01 | 1.96E+01 | 1.50E+01 | 3.78E+01 | 2.19E+01 | 26.1 |  |  | x |
| 162.0924 | 0.34 | 1.13E+02 | 9.49E+01 | 1.03E+02 | 8.76E+01 | 1.25E+02 | 7.63E+01 | 1.02E+02 | 1.06E+02 | 1.21E+02 | 8.42E+01 | 9.85E+01 | 1.18E+02 | 9.80E+01 | 1.12E+02 | 1.02E+02 | 1.34E+02 | 1.13E+02 | 14.3 | x | x | x |
| 205.0623 | 0.34 | 9.28E+01 | 9.11E+01 | 8.26E+01 | 9.99E+01 | 1.07E+02 | 1.10E+02 | 1.15E+02 | 1.17E+02 | 1.15E+02 | 9.91E+01 | 1.09E+02 | 1.13E+02 | 1.16E+02 | 1.24E+02 | 1.15E+02 | 1.32E+02 | 1.26E+02 | 12.0 | x | x | x |
| 239.1433 | 0.34 | 3.16E+02 | 2.98E+02 | 3.00E+02 | 3.13E+02 | 3.39E+02 | 3.62E+02 | 3.71E+02 | 3.79E+02 | 3.53E+02 | 3.05E+02 | 3.71E+02 | 3.75E+02 | 3.34E+02 | 3.38E+02 | 3.75E+02 | 4.34E+02 | 4.04E+02 | 10.9 | x | x | x |
| 253.1552 | 0.34 | 5.50E+01 | 5.06E+01 | 4.84E+01 | 4.64E+01 | 5.67E+01 | 7.02E+01 | 6.23E+01 | 6.98E+01 | 5.87E+01 | 4.99E+01 | 6.80E+01 | 7.04E+01 | 5.34E+01 | 6.33E+01 | 5.29E+01 | 6.82E+01 | 7.46E+01 | 15.1 |  | x | x |
| 267.1345 | 0.34 | 1.01E+02 | 8.74E+01 | 9.26E+01 | 9.76E+01 | 1.12E+02 | 9.05E+01 | 1.15E+02 | 1.22E+02 | 1.20E+02 | 1.17E+02 | 1.11E+02 | 1.35E+02 | 1.20E+02 | 1.35E+02 | 1.22E+02 | 1.56E+02 | 1.41E+02 | 16.2 |  | x | x |
| 289.1180 | 0.34 | 9.39E+01 | 8.47E+01 | 8.64E+01 | 8.21E+01 | 9.50E+01 | 7.56E+01 | 8.56E+01 | 9.50E+01 | 8.45E+01 | 6.13E+01 | 8.91E+01 | 1.01E+02 | 7.39E+01 | 5.95E+01 | 6.62E+01 | 7.82E+01 | 6.65E+01 | 15.3 |  | x | x |
| 124.0405 | 0.35 | 9.47E+01 | 8.57E+01 | 9.60E+01 | 1.16E+02 | 1.10E+02 | 9.42E+01 | 1.26E+02 | 1.37E+02 | 1.33E+02 | 1.26E+02 | 1.47E+02 | 1.81E+02 | 1.77E+02 | 1.76E+02 | 1.73E+02 | 1.95E+02 | 2.03E+02 | 27.5 |  |  | x |
| 281.1508 | 0.35 | 2.03E+02 | 1.79E+02 | 1.87E+02 | 1.84E+02 | 2.07E+02 | 2.03E+02 | 2.23E+02 | 2.24E+02 | 2.14E+02 | 2.10E+02 | 2.42E+02 | 2.46E+02 | 2.29E+02 | 2.34E+02 | 2.77E+02 | 3.20E+02 | 3.07E+02 | 17.6 |  | x | x |
| 160.1344 | 0.35 | 2.64E+02 | 2.34E+02 | 2.24E+02 | 2.33E+02 | 2.12E+02 | 3.53E+02 | 2.45E+02 | 2.35E+02 | 2.37E+02 | 2.10E+02 | 2.33E+02 | 2.49E+02 | 2.80E+02 | 2.72E+02 | 2.76E+02 | 3.35E+02 | 3.04E+02 | 15.8 |  | x | x |
| 146.1185 | 0.38 | 7.59E+01 | 7.16E+01 | 6.73E+01 | 7.41E+01 | 6.18E+01 | 1.02E+02 | 6.04E+01 | 5.95E+01 | 5.20E+01 | 3.97E+01 | 6.56E+01 | 5.99E+01 | 5.25E+01 | 4.16E+01 | 5.42E+01 | 5.50E+01 | 4.30E+01 | 24.9 |  |  | x |
| 293.1550 | 0.38 | 9.30E+01 | 6.00E+01 | 7.69E+01 | 7.11E+01 | 7.17E+01 | 7.42E+01 | 9.46E+01 | 8.46E+01 | 9.12E+01 | 8.07E+01 | 9.77E+01 | 1.06E+02 | 9.60E+01 | 1.16E+02 | 1.31E+02 | 1.39E+02 | 1.43E+02 | 25.5 |  |  | x |
| 123.0565 | 0.39 | 4.33E+02 | 4.27E+02 | 4.22E+02 | 4.55E+02 | 4.68E+02 | 5.51E+02 | 5.01E+02 | 5.26E+02 | 4.92E+02 | 4.80E+02 | 5.37E+02 | 5.77E+02 | 5.66E+02 | 5.66E+02 | 5.72E+02 | 6.12E+02 | 5.61E+02 | 11.5 | x | x | x |
| 323.1660 | 0.39 | 2.58E+01 | 2.89E+01 | 2.42E+01 | 3.08E+01 | 2.84E+01 | 0.00E+00 | 3.40E+01 | 3.47E+01 | 2.77E+01 | 3.40E+01 | 3.51E+01 | 3.62E+01 | 3.03E+01 | 3.49E+01 | 4.07E+01 | 3.72E+01 | 3.41E+01 | 29.5 |  |  | x |
| 130.0661 | 0.43 | 1.57E+03 | 1.47E+03 | 1.52E+03 | 1.56E+03 | 1.57E+03 | 1.79E+03 | 1.57E+03 | 1.62E+03 | 1.49E+03 | 1.44E+03 | 1.61E+03 | 1.64E+03 | 1.59E+03 | 1.55E+03 | 1.51E+03 | 1.62E+03 | 1.55E+03 | 5.1 | x | x | x |
| 162.0564 | 0.46 | 2.88E+03 | 2.69E+03 | 2.77E+03 | 3.05E+03 | 3.32E+03 | 3.62E+03 | 3.49E+03 | 3.74E+03 | 3.64E+03 | 3.59E+03 | 4.04E+03 | 4.32E+03 | 4.58E+03 | 4.64E+03 | 4.10E+03 | 4.61E+03 | 4.63E+03 | 17.9 |  | x | x |
| 211.1093 | 0.47 | 7.80E+02 | 7.65E+02 | 7.66E+02 | 8.20E+02 | 8.12E+02 | 7.07E+02 | 8.30E+02 | 8.72E+02 | 8.38E+02 | 7.95E+02 | 8.44E+02 | 9.36E+02 | 8.85E+02 | 8.83E+02 | 8.48E+02 | 9.19E+02 | 9.24E+02 | 7.5 | x | x | x |
| 126.0925 | 0.51 | 1.58E+02 | 1.50E+02 | 1.57E+02 | 1.60E+02 | 1.81E+02 | 1.78E+02 | 1.81E+02 | 1.98E+02 | 1.84E+02 | 1.96E+02 | 1.89E+02 | 2.12E+02 | 1.87E+02 | 1.99E+02 | 1.80E+02 | 2.16E+02 | 2.18E+02 | 11.1 | x | x | x |
| 185.1293 | 0.51 | 1.89E+02 | 1.84E+02 | 1.78E+02 | 1.84E+02 | 1.94E+02 | 1.82E+02 | 2.10E+02 | 2.19E+02 | 2.02E+02 | 2.23E+02 | 2.10E+02 | 2.31E+02 | 2.22E+02 | 2.04E+02 | 1.76E+02 | 2.20E+02 | 2.13E+02 | 8.8 | x | x | x |
| 170.0612 | 0.53 | 6.78E+02 | 6.27E+02 | 6.72E+02 | 6.65E+02 | 7.84E+02 | 3.96E+02 | 8.41E+02 | 8.72E+02 | 8.63E+02 | 8.79E+02 | 8.98E+02 | 8.74E+02 | 8.62E+02 | 8.54E+02 | 6.61E+02 | 8.72E+02 | 7.89E+02 | 17.5 |  | x | x |
| 164.0716 | 0.54 | 1.07E+02 | 8.24E+01 | 8.71E+01 | 7.20E+01 | 8.19E+01 | 1.10E+02 | 8.83E+01 | 7.26E+01 | 7.45E+01 | 6.83E+01 | 6.57E+01 | 5.86E+01 | 6.35E+01 | 5.42E+01 | 4.44E+01 | 6.21E+01 | 4.93E+01 | 25.0 |  |  | x |
| 176.0924 | 0.56 | 2.17E+01 | 1.76E+01 | 2.85E+01 | 1.07E+01 | 1.60E+01 | 1.26E+01 | 2.04E+01 | 2.01E+01 | 2.11E+01 | 2.50E+01 | 1.39E+01 | 2.45E+01 | 2.13E+01 | 1.81E+01 | 1.59E+01 | 2.62E+01 | 1.58E+01 | 25.6 |  |  | x |
| 153.0665 | 0.56 | 9.21E+02 | 8.53E+02 | 8.81E+02 | 9.14E+02 | 1.03E+03 | 1.13E+03 | 1.12E+03 | 1.17E+03 | 1.17E+03 | 1.25E+03 | 1.25E+03 | 1.33E+03 | 1.43E+03 | 1.38E+03 | 1.26E+03 | 1.52E+03 | 1.41E+03 | 17.4 |  | x | x |
| 215.0786 | 0.56 | 1.03E+02 | 9.85E+01 | 9.84E+01 | 1.08E+02 | 8.57E+01 | 4.30E+01 | 1.04E+02 | 1.03E+02 | 9.82E+01 | 1.05E+02 | 9.55E+01 | 8.81E+01 | 1.03E+02 | 8.90E+01 | 7.29E+01 | 8.32E+01 | 7.05E+01 | 18.3 |  | x | x |
| 92.0502 | 0.57 | 2.98E+01 | 2.39E+01 | 2.76E+01 | 2.34E+01 | 3.09E+01 | 2.17E+01 | 3.22E+01 | 3.72E+01 | 3.44E+01 | 3.10E+01 | 2.43E+01 | 3.25E+01 | 4.01E+01 | 3.84E+01 | 3.31E+01 | 3.88E+01 | 2.85E+01 | 18.3 |  | x | x |
| 136.0401 | 0.57 | 3.39E+03 | 3.27E+03 | 3.37E+03 | 3.37E+03 | 3.91E+03 | 4.27E+03 | 4.10E+03 | 4.36E+03 | 4.29E+03 | 4.71E+03 | 4.54E+03 | 5.16E+03 | 6.13E+03 | 5.53E+03 | 5.29E+03 | 6.59E+03 | 6.03E+03 | 22.6 |  |  | x |
| 190.1229 | 0.59 | 7.40E+01 | 6.99E+01 | 7.66E+01 | 7.54E+01 | 6.29E+01 | 9.50E+00 | 6.81E+01 | 5.56E+01 | 6.47E+01 | 6.27E+01 | 6.39E+01 | 7.26E+01 | 8.21E+01 | 7.06E+01 | 7.74E+01 | 7.51E+01 | 7.14E+01 | 24.2 |  |  | x |
| 270.0804 | 0.59 | 1.52E+02 | 1.40E+02 | 1.48E+02 | 1.46E+02 | 1.39E+02 | 1.49E+02 | 1.43E+02 | 1.46E+02 | 1.33E+02 | 1.44E+02 | 1.28E+02 | 1.27E+02 | 1.49E+02 | 1.44E+02 | 1.41E+02 | 1.58E+02 | 1.49E+02 | 5.7 | x | x | x |
| 179.0243 | 0.63 | 6.76E+02 | 5.44E+02 | 5.93E+02 | 6.30E+02 | 5.63E+02 | 5.12E+02 | 5.94E+02 | 6.01E+02 | 5.43E+02 | 5.87E+02 | 5.33E+02 | 4.95E+02 | 4.24E+02 | 4.52E+02 | 4.06E+02 | 4.20E+02 | 3.43E+02 | 17.2 |  | x | x |
| 319.1275 | 0.63 | 2.52E+02 | 2.45E+02 | 2.57E+02 | 2.17E+02 | 2.85E+02 | 4.32E+02 | 2.42E+02 | 2.48E+02 | 2.39E+02 | 2.49E+02 | 2.41E+02 | 2.56E+02 | 2.94E+02 | 2.34E+02 | 2.10E+02 | 2.49E+02 | 2.41E+02 | 19.0 |  | x | x |
| 105.9546 | 0.64 | 1.26E+04 | 1.08E+04 | 1.18E+04 | 1.26E+04 | 1.24E+04 | 2.61E+04 | 1.32E+04 | 1.34E+04 | 1.31E+04 | 1.57E+04 | 1.49E+04 | 1.59E+04 | 1.65E+04 | 1.60E+04 | 1.59E+04 | 1.69E+04 | 1.73E+04 | 23.2 |  |  | x |
| 144.9832 | 0.64 | 5.07E+04 | 4.48E+04 | 4.65E+04 | 5.03E+04 | 5.11E+04 | 7.64E+04 | 5.47E+04 | 5.55E+04 | 5.44E+04 | 6.06E+04 | 6.16E+04 | 6.63E+04 | 6.81E+04 | 6.67E+04 | 6.73E+04 | 7.18E+04 | 7.22E+04 | 16.3 |  | x | x |
| 146.9814 | 0.64 | 2.09E+04 | 1.83E+04 | 1.91E+04 | 2.05E+04 | 2.09E+04 | 3.10E+04 | 2.23E+04 | 2.25E+04 | 2.21E+04 | 2.43E+04 | 2.49E+04 | 2.69E+04 | 2.70E+04 | 2.67E+04 | 2.68E+04 | 2.85E+04 | 2.88E+04 | 15.4 |  | x | x |
| 260.1044 | 0.64 | 9.05E+01 | 1.05E+02 | 9.84E+01 | 9.75E+01 | 1.06E+02 | 0.00E+00 | 9.66E+01 | 1.03E+02 | 8.51E+01 | 9.04E+01 | 6.93E+01 | 1.04E+02 | 1.09E+02 | 7.86E+01 | 7.29E+01 | 7.98E+01 | 8.33E+01 | 29.4 |  |  | x |
| 103.9566 | 0.65 | 8.96E+04 | 8.07E+04 | 8.43E+04 | 9.37E+04 | 9.50E+04 | 1.43E+05 | 1.01E+05 | 1.04E+05 | 1.02E+05 | 1.14E+05 | 1.16E+05 | 1.25E+05 | 1.32E+05 | 1.27E+05 | 1.30E+05 | 1.38E+05 | 1.40E+05 | 18.2 |  | x | x |
| 220.1188 | 0.65 | 1.17E+03 | 1.10E+03 | 1.10E+03 | 1.01E+03 | 9.40E+02 | 6.57E+02 | 9.75E+02 | 9.56E+02 | 8.58E+02 | 9.68E+02 | 8.50E+02 | 9.24E+02 | 1.01E+03 | 8.38E+02 | 9.51E+02 | 9.95E+02 | 8.71E+02 | 12.6 | x | x | x |
| 151.1439 | 0.66 | 9.44E+01 | 6.81E+01 | 7.72E+01 | 7.55E+01 | 6.52E+01 | 8.29E+01 | 7.87E+01 | 8.38E+01 | 6.91E+01 | 7.38E+01 | 7.18E+01 | 6.99E+01 | 6.14E+01 | 5.64E+01 | 7.01E+01 | 6.51E+01 | 5.73E+01 | 13.7 | x | x | x |
| 335.1015 | 0.67 | 3.12E+02 | 2.74E+02 | 2.94E+02 | 3.03E+02 | 3.45E+02 | 7.61E+01 | 3.08E+02 | 3.58E+02 | 3.41E+02 | 4.10E+02 | 3.63E+02 | 4.24E+02 | 4.39E+02 | 4.93E+02 | 4.31E+02 | 4.15E+02 | 4.61E+02 | 27.2 |  |  | x |
| 289.9300 | 0.73 | 1.20E+03 | 1.22E+03 | 1.35E+03 | 1.41E+03 | 1.61E+03 | 1.91E+03 | 1.66E+03 | 1.92E+03 | 1.80E+03 | 2.16E+03 | 1.91E+03 | 2.19E+03 | 2.45E+03 | 2.27E+03 | 2.30E+03 | 2.39E+03 | 2.30E+03 | 22.2 |  |  | x |
| 291.9274 | 0.73 | 9.32E+01 | 9.77E+01 | 1.05E+02 | 9.91E+01 | 1.22E+02 | 1.34E+02 | 1.23E+02 | 1.24E+02 | 1.24E+02 | 1.51E+02 | 1.34E+02 | 1.45E+02 | 1.52E+02 | 1.42E+02 | 1.59E+02 | 1.61E+02 | 1.30E+02 | 16.5 |  | x | x |
| 540.8958 | 0.73 | 1.28E+02 | 1.38E+02 | 1.50E+02 | 1.45E+02 | 1.78E+02 | 1.75E+02 | 1.87E+02 | 2.21E+02 | 2.19E+02 | 2.43E+02 | 2.10E+02 | 2.33E+02 | 2.58E+02 | 2.64E+02 | 2.55E+02 | 2.50E+02 | 2.50E+02 | 22.5 |  |  | x |
| 212.9842 | 0.74 | 7.75E+02 | 7.10E+02 | 7.70E+02 | 9.35E+02 | 9.11E+02 | 1.02E+03 | 1.02E+03 | 1.07E+03 | 1.00E+03 | 1.13E+03 | 1.07E+03 | 1.11E+03 | 1.23E+03 | 1.22E+03 | 1.06E+03 | 1.16E+03 | 1.16E+03 | 15.2 |  | x | x |
| 214.9827 | 0.74 | 2.50E+02 | 2.19E+02 | 2.36E+02 | 2.90E+02 | 2.93E+02 | 2.98E+02 | 2.96E+02 | 3.13E+02 | 2.96E+02 | 3.18E+02 | 3.10E+02 | 3.18E+02 | 3.54E+02 | 3.42E+02 | 2.92E+02 | 3.12E+02 | 3.26E+02 | 11.8 | x | x | x |
| 254.0109 | 0.74 | 3.71E+02 | 3.27E+02 | 3.52E+02 | 4.16E+02 | 4.00E+02 | 4.42E+02 | 4.56E+02 | 4.70E+02 | 4.35E+02 | 5.04E+02 | 4.89E+02 | 4.98E+02 | 5.52E+02 | 5.51E+02 | 4.83E+02 | 5.09E+02 | 5.07E+02 | 14.5 | x | x | x |
| 256.0089 | 0.74 | 1.16E+02 | 1.03E+02 | 1.13E+02 | 1.27E+02 | 1.35E+02 | 1.13E+02 | 1.41E+02 | 1.50E+02 | 1.39E+02 | 1.45E+02 | 1.47E+02 | 1.45E+02 | 1.53E+02 | 1.55E+02 | 1.14E+02 | 1.48E+02 | 1.34E+02 | 12.3 | x | x | x |
| 315.9286 | 0.74 | 8.36E+01 | 7.14E+01 | 9.09E+01 | 9.89E+01 | 9.50E+01 | 9.31E+01 | 1.15E+02 | 1.13E+02 | 9.01E+01 | 1.38E+02 | 1.14E+02 | 1.28E+02 | 1.24E+02 | 1.05E+02 | 1.16E+02 | 1.19E+02 | 1.19E+02 | 16.6 |  | x | x |
| 320.9241 | 0.83 | 6.30E+01 | 5.97E+01 | 7.73E+01 | 5.62E+01 | 5.56E+01 | 0.00E+00 | 6.00E+01 | 7.07E+01 | 6.54E+01 | 7.22E+01 | 7.13E+01 | 6.77E+01 | 5.21E+01 | 5.79E+01 | 6.54E+01 | 6.10E+01 | 5.52E+01 | 28.3 |  |  | x |
| 172.0409 | 0.88 | 1.32E+02 | 1.06E+02 | 1.19E+02 | 1.32E+02 | 1.37E+02 | 1.96E+02 | 1.34E+02 | 1.35E+02 | 1.21E+02 | 1.37E+02 | 1.36E+02 | 1.39E+02 | 1.26E+02 | 1.39E+02 | 8.39E+01 | 9.04E+01 | 7.73E+01 | 21.5 |  |  | x |
| 300.0855 | 0.90 | 4.63E+02 | 4.37E+02 | 4.80E+02 | 4.63E+02 | 5.16E+02 | 5.40E+02 | 5.11E+02 | 5.50E+02 | 5.24E+02 | 5.96E+02 | 5.29E+02 | 6.11E+02 | 6.02E+02 | 5.78E+02 | 5.74E+02 | 5.93E+02 | 5.69E+02 | 10.0 | x | x | x |
| 350.9351 | 0.90 | 1.24E+03 | 1.17E+03 | 1.26E+03 | 1.16E+03 | 1.31E+03 | 1.29E+03 | 1.32E+03 | 1.38E+03 | 1.31E+03 | 1.51E+03 | 1.40E+03 | 1.53E+03 | 1.54E+03 | 1.49E+03 | 1.51E+03 | 1.59E+03 | 1.44E+03 | 9.8 | x | x | x |
| 320.9244 | 0.97 | 4.57E+01 | 3.34E+01 | 4.04E+01 | 3.26E+01 | 4.32E+01 | 2.08E+01 | 3.79E+01 | 4.51E+01 | 3.39E+01 | 4.31E+01 | 3.16E+01 | 3.34E+01 | 2.44E+01 | 3.38E+01 | 3.94E+01 | 4.22E+01 | 2.57E+01 | 20.8 |  |  | x |
| 335.0230 | 0.98 | 1.91E+02 | 1.66E+02 | 1.63E+02 | 1.73E+02 | 1.63E+02 | 6.87E+01 | 1.63E+02 | 1.65E+02 | 1.48E+02 | 1.64E+02 | 1.29E+02 | 1.43E+02 | 1.16E+02 | 8.89E+01 | 1.03E+02 | 1.06E+02 | 8.05E+01 | 26.8 |  |  | x |
| 410.9355 | 0.98 | 1.07E+03 | 9.80E+02 | 1.08E+03 | 1.09E+03 | 1.12E+03 | 8.95E+02 | 1.22E+03 | 1.25E+03 | 1.19E+03 | 1.38E+03 | 1.25E+03 | 1.31E+03 | 1.34E+03 | 1.26E+03 | 1.31E+03 | 1.37E+03 | 1.32E+03 | 11.8 | x | x | x |
| 309.9193 | 1.00 | 4.40E+01 | 4.76E+01 | 5.71E+01 | 4.73E+01 | 5.30E+01 | 6.47E+00 | 5.38E+01 | 5.92E+01 | 5.33E+01 | 5.77E+01 | 5.31E+01 | 4.95E+01 | 5.60E+01 | 4.89E+01 | 4.94E+01 | 5.72E+01 | 3.78E+01 | 25.0 |  |  | x |
| 406.9971 | 1.00 | 3.47E+01 | 2.89E+01 | 3.53E+01 | 3.06E+01 | 3.52E+01 | 3.06E+01 | 3.59E+01 | 2.87E+01 | 3.12E+01 | 3.78E+01 | 3.61E+01 | 3.07E+01 | 3.05E+01 | 3.04E+01 | 2.54E+01 | 3.73E+01 | 2.58E+01 | 12.0 | x | x | x |
| 421.0126 | 1.00 | 2.39E+02 | 2.35E+02 | 2.48E+02 | 2.42E+02 | 2.48E+02 | 2.15E+02 | 2.61E+02 | 2.46E+02 | 2.59E+02 | 2.75E+02 | 2.56E+02 | 2.49E+02 | 2.56E+02 | 2.35E+02 | 2.27E+02 | 2.41E+02 | 2.37E+02 | 5.7 | x | x | x |
| 322.9396 | 1.02 | 8.59E+02 | 7.84E+02 | 8.75E+02 | 8.80E+02 | 7.92E+02 | 1.06E+03 | 9.39E+02 | 9.47E+02 | 9.29E+02 | 9.59E+02 | 9.45E+02 | 9.09E+02 | 9.30E+02 | 8.76E+02 | 9.65E+02 | 9.42E+02 | 9.82E+02 | 7.4 | x | x | x |
| 324.9365 | 1.02 | 9.64E+01 | 8.33E+01 | 9.24E+01 | 8.16E+01 | 7.98E+01 | 1.10E+02 | 9.28E+01 | 8.76E+01 | 7.89E+01 | 9.37E+01 | 9.30E+01 | 7.74E+01 | 6.47E+01 | 7.18E+01 | 7.93E+01 | 8.41E+01 | 7.55E+01 | 12.7 | x | x | x |
| 327.0483 | 1.02 | 3.13E+02 | 2.86E+02 | 3.32E+02 | 3.22E+02 | 2.80E+02 | 3.86E+02 | 3.34E+02 | 3.28E+02 | 3.16E+02 | 3.24E+02 | 3.05E+02 | 3.11E+02 | 3.07E+02 | 2.93E+02 | 2.90E+02 | 3.23E+02 | 2.99E+02 | 7.8 | x | x | x |
| 282.9083 | 1.04 | 1.21E+03 | 1.14E+03 | 1.23E+03 | 1.28E+03 | 1.10E+03 | 1.61E+03 | 1.46E+03 | 1.50E+03 | 1.40E+03 | 1.59E+03 | 1.48E+03 | 1.56E+03 | 1.59E+03 | 1.33E+03 | 1.55E+03 | 1.65E+03 | 1.56E+03 | 12.4 | x | x | x |
| 307.0447 | 1.04 | 4.80E+01 | 3.59E+01 | 3.47E+01 | 3.96E+01 | 2.96E+01 | 6.43E+01 | 4.39E+01 | 4.57E+01 | 3.85E+01 | 4.39E+01 | 3.23E+01 | 3.36E+01 | 2.96E+01 | 3.45E+01 | 3.32E+01 | 1.64E+01 | 3.02E+01 | 27.5 |  |  | x |
| 284.9066 | 1.05 | 1.07E+02 | 9.34E+01 | 1.07E+02 | 9.07E+01 | 8.62E+01 | 1.16E+02 | 1.19E+02 | 1.21E+02 | 9.75E+01 | 1.18E+02 | 1.08E+02 | 1.12E+02 | 9.83E+01 | 1.04E+02 | 1.02E+02 | 1.03E+02 | 9.71E+01 | 9.8 | x | x | x |
| 321.0645 | 1.06 | 4.32E+02 | 4.25E+02 | 4.35E+02 | 4.37E+02 | 3.56E+02 | 5.30E+02 | 4.81E+02 | 4.59E+02 | 4.32E+02 | 4.87E+02 | 4.45E+02 | 4.74E+02 | 4.73E+02 | 3.89E+02 | 4.65E+02 | 4.56E+02 | 4.69E+02 | 8.7 | x | x | x |
| 352.9488 | 1.06 | 1.76E+02 | 1.70E+02 | 1.77E+02 | 1.58E+02 | 1.34E+02 | 1.88E+02 | 1.75E+02 | 1.73E+02 | 1.63E+02 | 1.84E+02 | 1.55E+02 | 1.67E+02 | 1.58E+02 | 1.19E+02 | 1.36E+02 | 1.47E+02 | 1.46E+02 | 11.9 | x | x | x |
| 222.0198 | 1.07 | 3.32E+02 | 3.12E+02 | 3.14E+02 | 3.48E+02 | 2.99E+02 | 3.72E+02 | 3.42E+02 | 3.78E+02 | 3.28E+02 | 3.65E+02 | 3.46E+02 | 3.64E+02 | 3.61E+02 | 3.16E+02 | 3.24E+02 | 3.83E+02 | 3.35E+02 | 7.4 | x | x | x |
| 475.9763 | 1.07 | 1.51E+01 | 1.78E+01 | 1.75E+01 | 1.66E+01 | 1.60E+01 | 1.78E+01 | 1.39E+01 | 1.39E+01 | 2.45E+01 | 1.20E+01 | 1.63E+01 | 2.55E+01 | 1.35E+01 | 1.41E+01 | 1.49E+01 | 1.30E+01 | 2.23E+01 | 23.5 |  |  | x |
| 299.1004 | 1.08 | 9.09E+02 | 8.87E+02 | 8.59E+02 | 8.61E+02 | 7.64E+02 | 9.79E+02 | 9.03E+02 | 9.40E+02 | 8.35E+02 | 9.62E+02 | 8.76E+02 | 9.77E+02 | 9.68E+02 | 8.11E+02 | 9.34E+02 | 9.63E+02 | 9.12E+02 | 6.9 | x | x | x |
| 375.0249 | 1.08 | 5.46E+03 | 5.45E+03 | 5.74E+03 | 6.01E+03 | 5.34E+03 | 8.26E+03 | 6.96E+03 | 7.28E+03 | 6.93E+03 | 8.29E+03 | 7.76E+03 | 8.47E+03 | 9.39E+03 | 7.21E+03 | 9.23E+03 | 1.02E+04 | 1.01E+04 | 21.5 |  |  | x |
| 425.1576 | 1.08 | 1.27E+02 | 1.22E+02 | 1.23E+02 | 1.37E+02 | 1.18E+02 | 1.45E+02 | 1.50E+02 | 1.50E+02 | 1.48E+02 | 1.55E+02 | 1.44E+02 | 1.42E+02 | 1.44E+02 | 1.31E+02 | 1.49E+02 | 1.44E+02 | 1.34E+02 | 8.2 | x | x | x |
| 618.9703 | 1.08 | 6.24E+01 | 6.08E+01 | 6.36E+01 | 6.36E+01 | 4.99E+01 | 6.87E+01 | 7.43E+01 | 7.16E+01 | 6.55E+01 | 8.58E+01 | 7.77E+01 | 8.40E+01 | 8.06E+01 | 5.75E+01 | 8.55E+01 | 9.50E+01 | 8.37E+01 | 16.9 |  | x | x |
| 711.0877 | 1.08 | 1.28E+03 | 1.30E+03 | 1.37E+03 | 1.39E+03 | 1.14E+03 | 1.69E+03 | 1.72E+03 | 1.79E+03 | 1.71E+03 | 2.01E+03 | 1.87E+03 | 2.12E+03 | 2.22E+03 | 1.65E+03 | 2.40E+03 | 2.50E+03 | 2.39E+03 | 23.7 |  |  | x |
| 157.0972 | 1.09 | 8.39E+01 | 8.12E+01 | 7.58E+01 | 7.45E+01 | 5.81E+01 | 5.15E+01 | 7.12E+01 | 7.22E+01 | 6.48E+01 | 6.41E+01 | 5.78E+01 | 4.97E+01 | 5.66E+01 | 4.94E+01 | 5.07E+01 | 4.95E+01 | 4.51E+01 | 19.9 |  | x | x |
| 239.1007 | 1.09 | 1.77E+03 | 1.58E+03 | 1.56E+03 | 1.69E+03 | 1.43E+03 | 3.53E+03 | 1.71E+03 | 1.72E+03 | 1.61E+03 | 1.90E+03 | 1.72E+03 | 1.85E+03 | 1.93E+03 | 1.69E+03 | 1.80E+03 | 1.68E+03 | 1.70E+03 | 25.3 |  |  | x |
| 255.0746 | 1.09 | 7.11E+03 | 6.71E+03 | 6.69E+03 | 6.98E+03 | 6.10E+03 | 7.72E+03 | 7.28E+03 | 7.51E+03 | 7.03E+03 | 8.28E+03 | 7.81E+03 | 8.45E+03 | 9.20E+03 | 7.61E+03 | 8.80E+03 | 9.48E+03 | 9.08E+03 | 12.7 | x | x | x |
| 697.0725 | 1.10 | 3.80E+02 | 3.76E+02 | 4.00E+02 | 3.99E+02 | 3.30E+02 | 4.66E+02 | 4.48E+02 | 4.67E+02 | 4.33E+02 | 5.23E+02 | 4.58E+02 | 5.24E+02 | 5.14E+02 | 3.43E+02 | 5.06E+02 | 5.71E+02 | 5.33E+02 | 15.9 |  | x | x |
| 361.0095 | 1.11 | 2.44E+03 | 2.24E+03 | 2.39E+03 | 2.52E+03 | 2.39E+03 | 3.38E+03 | 2.88E+03 | 2.97E+03 | 2.82E+03 | 3.30E+03 | 3.13E+03 | 3.34E+03 | 3.61E+03 | 2.76E+03 | 3.50E+03 | 3.78E+03 | 3.69E+03 | 16.7 |  | x | x |
| 683.0566 | 1.11 | 2.52E+02 | 2.16E+02 | 2.30E+02 | 2.13E+02 | 2.17E+02 | 3.10E+02 | 3.07E+02 | 3.07E+02 | 2.76E+02 | 3.05E+02 | 2.97E+02 | 3.10E+02 | 3.20E+02 | 2.27E+02 | 3.22E+02 | 3.53E+02 | 3.35E+02 | 16.5 |  | x | x |
| 282.1201 | 1.12 | 1.02E+04 | 9.50E+03 | 1.00E+04 | 1.09E+04 | 1.03E+04 | 1.11E+04 | 1.12E+04 | 1.18E+04 | 1.12E+04 | 1.27E+04 | 1.20E+04 | 1.31E+04 | 1.51E+04 | 1.14E+04 | 1.46E+04 | 1.53E+04 | 1.42E+04 | 15.3 |  | x | x |
| 136.1125 | 1.12 | 1.21E+03 | 1.13E+03 | 1.21E+03 | 1.24E+03 | 1.21E+03 | 1.21E+03 | 1.28E+03 | 1.31E+03 | 1.21E+03 | 1.41E+03 | 1.34E+03 | 1.33E+03 | 1.32E+03 | 1.18E+03 | 1.35E+03 | 1.40E+03 | 1.25E+03 | 6.3 | x | x | x |
| 370.0802 | 1.12 | 5.70E+01 | 5.88E+01 | 5.43E+01 | 5.10E+01 | 4.39E+01 | 4.06E+01 | 4.86E+01 | 4.40E+01 | 3.29E+01 | 4.01E+01 | 4.31E+01 | 3.47E+01 | 4.53E+01 | 1.97E+01 | 3.69E+01 | 3.88E+01 | 4.02E+01 | 22.3 |  |  | x |
| 103.9564 | 1.13 | 2.06E+05 | 1.93E+05 | 2.01E+05 | 2.17E+05 | 2.10E+05 | 2.36E+05 | 2.37E+05 | 2.40E+05 | 2.28E+05 | 2.76E+05 | 2.58E+05 | 2.79E+05 | 2.92E+05 | 2.72E+05 | 2.87E+05 | 3.08E+05 | 3.08E+05 | 15.2 |  | x | x |
| 105.9545 | 1.13 | 8.62E+04 | 8.08E+04 | 8.45E+04 | 9.09E+04 | 8.77E+04 | 9.68E+04 | 9.89E+04 | 1.00E+05 | 9.52E+04 | 1.15E+05 | 1.07E+05 | 1.16E+05 | 1.21E+05 | 1.12E+05 | 1.19E+05 | 1.28E+05 | 1.27E+05 | 14.9 | x | x | x |
| 144.9830 | 1.13 | 1.16E+05 | 1.07E+05 | 1.10E+05 | 1.17E+05 | 1.13E+05 | 1.24E+05 | 1.28E+05 | 1.28E+05 | 1.21E+05 | 1.47E+05 | 1.37E+05 | 1.47E+05 | 1.50E+05 | 1.43E+05 | 1.49E+05 | 1.60E+05 | 1.58E+05 | 13.1 | x | x | x |
| 146.9812 | 1.13 | 4.59E+04 | 4.24E+04 | 4.40E+04 | 4.62E+04 | 4.46E+04 | 4.77E+04 | 5.06E+04 | 5.06E+04 | 4.78E+04 | 5.74E+04 | 5.37E+04 | 5.78E+04 | 5.85E+04 | 5.55E+04 | 5.78E+04 | 6.13E+04 | 6.17E+04 | 12.3 | x | x | x |
| 298.0972 | 1.13 | 2.66E+01 | 2.73E+01 | 2.71E+01 | 2.37E+01 | 2.18E+01 | 1.23E+01 | 2.69E+01 | 2.42E+01 | 1.94E+01 | 2.08E+01 | 1.97E+01 | 1.85E+01 | 1.28E+01 | 2.01E+01 | 1.54E+01 | 2.23E+01 | 1.82E+01 | 22.5 |  |  | x |
| 346.9936 | 1.13 | 1.60E+02 | 1.42E+02 | 1.52E+02 | 1.50E+02 | 1.69E+02 | 1.78E+02 | 1.74E+02 | 1.70E+02 | 1.57E+02 | 1.99E+02 | 1.90E+02 | 1.76E+02 | 1.73E+02 | 1.44E+02 | 1.63E+02 | 1.74E+02 | 1.76E+02 | 9.2 | x | x | x |
| 72.0815 | 1.14 | 9.32E+01 | 8.96E+01 | 8.33E+01 | 9.89E+01 | 9.76E+01 | 9.04E+01 | 1.01E+02 | 1.07E+02 | 9.31E+01 | 9.93E+01 | 9.77E+01 | 1.02E+02 | 1.15E+02 | 9.12E+01 | 1.07E+02 | 1.15E+02 | 1.13E+02 | 9.4 | x | x | x |
| 97.0767 | 1.14 | 2.52E+01 | 1.92E+01 | 1.89E+01 | 2.03E+01 | 2.08E+01 | 2.51E+01 | 2.27E+01 | 2.11E+01 | 2.35E+01 | 2.16E+01 | 2.28E+01 | 1.99E+01 | 1.68E+01 | 1.33E+01 | 1.48E+01 | 1.13E+01 | 1.67E+01 | 20.2 |  |  | x |
| 122.0969 | 1.14 | 1.86E+04 | 1.74E+04 | 1.83E+04 | 1.92E+04 | 1.92E+04 | 2.19E+04 | 2.06E+04 | 2.11E+04 | 1.99E+04 | 2.34E+04 | 2.20E+04 | 2.36E+04 | 2.48E+04 | 2.30E+04 | 2.40E+04 | 2.54E+04 | 2.52E+04 | 11.9 | x | x | x |
| 143.0822 | 1.14 | 1.50E+04 | 1.40E+04 | 1.44E+04 | 1.57E+04 | 1.55E+04 | 1.67E+04 | 1.63E+04 | 1.70E+04 | 1.63E+04 | 1.87E+04 | 1.76E+04 | 1.88E+04 | 2.07E+04 | 1.79E+04 | 1.99E+04 | 2.16E+04 | 2.07E+04 | 13.3 | x | x | x |
| 84.0811 | 1.15 | 3.38E+02 | 3.46E+02 | 3.48E+02 | 3.82E+02 | 3.98E+02 | 4.98E+02 | 3.77E+02 | 4.04E+02 | 3.79E+02 | 4.17E+02 | 3.84E+02 | 3.81E+02 | 4.06E+02 | 3.47E+02 | 3.89E+02 | 4.01E+02 | 3.94E+02 | 9.5 | x | x | x |
| 524.8797 | 1.16 | 2.36E+02 | 2.67E+02 | 2.47E+02 | 2.31E+02 | 2.43E+02 | 1.63E+02 | 2.34E+02 | 2.43E+02 | 2.20E+02 | 2.19E+02 | 2.22E+02 | 2.16E+02 | 2.43E+02 | 1.69E+02 | 2.55E+02 | 2.54E+02 | 2.28E+02 | 12.0 | x | x | x |
| 377.0060 | 1.16 | 1.88E+02 | 1.69E+02 | 1.77E+02 | 1.81E+02 | 1.91E+02 | 2.25E+02 | 2.18E+02 | 2.12E+02 | 1.98E+02 | 2.37E+02 | 2.01E+02 | 2.31E+02 | 2.10E+02 | 1.87E+02 | 1.90E+02 | 1.99E+02 | 2.21E+02 | 9.8 | x | x | x |
| 522.8796 | 1.16 | 3.83E+02 | 4.09E+02 | 3.88E+02 | 3.59E+02 | 3.38E+02 | 2.62E+02 | 3.62E+02 | 3.50E+02 | 3.23E+02 | 3.06E+02 | 3.29E+02 | 3.32E+02 | 3.79E+02 | 2.54E+02 | 3.54E+02 | 3.64E+02 | 2.97E+02 | 12.5 | x | x | x |
| 250.9825 | 1.17 | 1.57E+02 | 1.43E+02 | 1.45E+02 | 1.57E+02 | 1.48E+02 | 1.49E+02 | 1.59E+02 | 1.67E+02 | 1.48E+02 | 1.69E+02 | 1.65E+02 | 1.63E+02 | 1.86E+02 | 1.68E+02 | 1.68E+02 | 1.64E+02 | 1.81E+02 | 7.5 | x | x | x |
| 284.3309 | 1.18 | 2.01E+02 | 1.76E+02 | 1.97E+02 | 1.94E+02 | 1.95E+02 | 1.57E+02 | 2.13E+02 | 1.98E+02 | 1.88E+02 | 2.01E+02 | 1.73E+02 | 1.80E+02 | 1.80E+02 | 1.51E+02 | 1.55E+02 | 1.47E+02 | 1.29E+02 | 13.1 | x | x | x |
| 182.1280 | 1.19 | 4.48E+02 | 3.98E+02 | 3.88E+02 | 4.16E+02 | 3.98E+02 | 4.94E+02 | 4.11E+02 | 4.20E+02 | 3.93E+02 | 4.12E+02 | 4.17E+02 | 3.90E+02 | 4.08E+02 | 2.93E+02 | 3.92E+02 | 4.04E+02 | 3.53E+02 | 10.1 | x | x | x |
| 264.9995 | 1.19 | 1.19E+03 | 1.10E+03 | 1.17E+03 | 1.31E+03 | 1.30E+03 | 1.32E+03 | 1.37E+03 | 1.42E+03 | 1.40E+03 | 1.44E+03 | 1.48E+03 | 1.55E+03 | 1.75E+03 | 1.43E+03 | 1.62E+03 | 1.70E+03 | 1.63E+03 | 13.0 | x | x | x |
| 266.9974 | 1.19 | 1.46E+02 | 1.32E+02 | 1.28E+02 | 1.40E+02 | 1.23E+02 | 1.73E+02 | 1.33E+02 | 1.35E+02 | 1.42E+02 | 1.25E+02 | 1.30E+02 | 1.50E+02 | 1.28E+02 | 1.16E+02 | 1.26E+02 | 1.35E+02 | 1.27E+02 | 9.8 | x | x | x |
| 311.1006 | 1.19 | 2.40E+02 | 2.76E+02 | 2.17E+02 | 1.99E+02 | 1.99E+02 | 1.82E+02 | 1.85E+02 | 1.99E+02 | 1.85E+02 | 1.76E+02 | 1.65E+02 | 1.79E+02 | 1.71E+02 | 1.18E+02 | 1.63E+02 | 1.76E+02 | 1.36E+02 | 19.5 |  | x | x |
| 366.9656 | 1.19 | 2.18E+01 | 1.65E+01 | 1.98E+01 | 2.24E+01 | 1.96E+01 | 1.81E+01 | 2.44E+01 | 1.62E+01 | 2.22E+01 | 2.16E+01 | 2.86E+01 | 2.40E+01 | 1.69E+01 | 1.17E+01 | 2.30E+01 | 1.38E+01 | 1.71E+01 | 21.5 |  |  | x |
| 439.1338 | 1.19 | 2.06E+01 | 2.56E+01 | 2.70E+01 | 1.50E+01 | 2.58E+01 | 3.05E+01 | 2.73E+01 | 1.92E+01 | 2.54E+01 | 1.57E+01 | 2.33E+01 | 2.86E+01 | 2.30E+01 | 1.39E+01 | 2.00E+01 | 1.72E+01 | 2.58E+01 | 22.5 |  |  | x |
| 150.0779 | 1.20 | 2.47E+03 | 2.41E+03 | 2.38E+03 | 2.51E+03 | 2.54E+03 | 3.10E+03 | 2.64E+03 | 2.84E+03 | 2.68E+03 | 3.17E+03 | 2.95E+03 | 3.28E+03 | 3.36E+03 | 2.91E+03 | 3.28E+03 | 3.57E+03 | 3.48E+03 | 13.5 | x | x | x |
| 443.1664 | 1.20 | 1.05E+02 | 9.56E+01 | 1.06E+02 | 1.04E+02 | 1.01E+02 | 1.05E+02 | 1.05E+02 | 1.16E+02 | 1.10E+02 | 1.11E+02 | 1.06E+02 | 1.34E+02 | 1.30E+02 | 1.01E+02 | 1.22E+02 | 1.08E+02 | 1.31E+02 | 10.2 | x | x | x |
| 116.0500 | 1.21 | 6.22E+01 | 6.28E+01 | 6.14E+01 | 6.52E+01 | 6.32E+01 | 7.19E+01 | 7.06E+01 | 7.42E+01 | 7.51E+01 | 7.12E+01 | 7.01E+01 | 7.93E+01 | 8.07E+01 | 6.37E+01 | 7.82E+01 | 6.97E+01 | 7.67E+01 | 9.1 | x | x | x |
| 144.0451 | 1.21 | 4.05E+03 | 4.02E+03 | 4.22E+03 | 4.79E+03 | 4.90E+03 | 5.80E+03 | 4.96E+03 | 5.44E+03 | 5.32E+03 | 6.02E+03 | 5.68E+03 | 6.47E+03 | 6.88E+03 | 5.68E+03 | 6.49E+03 | 6.89E+03 | 6.62E+03 | 17.2 |  | x | x |
| 185.0713 | 1.21 | 1.69E+03 | 1.60E+03 | 1.60E+03 | 1.75E+03 | 1.77E+03 | 2.11E+03 | 1.89E+03 | 1.96E+03 | 1.93E+03 | 2.18E+03 | 2.04E+03 | 2.28E+03 | 2.30E+03 | 2.00E+03 | 2.22E+03 | 2.32E+03 | 2.23E+03 | 12.3 | x | x | x |
| 190.0504 | 1.21 | 1.32E+04 | 1.23E+04 | 1.27E+04 | 1.42E+04 | 1.44E+04 | 1.82E+04 | 1.51E+04 | 1.62E+04 | 1.57E+04 | 1.80E+04 | 1.72E+04 | 1.87E+04 | 1.98E+04 | 1.77E+04 | 1.88E+04 | 2.03E+04 | 1.96E+04 | 15.6 |  | x | x |
| 246.0208 | 1.21 | 1.41E+02 | 1.49E+02 | 1.33E+02 | 1.29E+02 | 1.22E+02 | 1.29E+02 | 1.24E+02 | 1.29E+02 | 1.22E+02 | 1.15E+02 | 1.10E+02 | 1.42E+02 | 1.27E+02 | 1.01E+02 | 1.13E+02 | 1.30E+02 | 1.14E+02 | 9.9 | x | x | x |
| 336.9205 | 1.21 | 6.79E+01 | 4.81E+01 | 6.37E+01 | 5.71E+01 | 5.62E+01 | 6.40E+01 | 6.89E+01 | 6.33E+01 | 6.15E+01 | 6.24E+01 | 6.23E+01 | 6.56E+01 | 5.46E+01 | 5.01E+01 | 6.18E+01 | 5.97E+01 | 5.54E+01 | 9.7 | x | x | x |
| 352.9493 | 1.21 | 1.17E+02 | 1.06E+02 | 1.16E+02 | 1.26E+02 | 1.23E+02 | 1.13E+02 | 1.22E+02 | 1.29E+02 | 1.18E+02 | 1.22E+02 | 1.24E+02 | 1.23E+02 | 1.38E+02 | 9.51E+01 | 1.17E+02 | 1.59E+02 | 1.28E+02 | 11.0 | x | x | x |
| 174.1243 | 1.22 | 1.67E+04 | 1.58E+04 | 1.55E+04 | 1.65E+04 | 1.66E+04 | 2.25E+04 | 1.66E+04 | 1.83E+04 | 1.77E+04 | 2.00E+04 | 1.91E+04 | 2.03E+04 | 2.14E+04 | 1.91E+04 | 2.00E+04 | 2.22E+04 | 2.16E+04 | 12.3 | x | x | x |
| 229.1549 | 1.22 | 3.66E+04 | 3.52E+04 | 3.47E+04 | 3.71E+04 | 3.55E+04 | 4.07E+04 | 3.68E+04 | 3.96E+04 | 3.80E+04 | 4.20E+04 | 4.05E+04 | 4.36E+04 | 4.69E+04 | 4.08E+04 | 4.27E+04 | 4.79E+04 | 4.55E+04 | 10.2 | x | x | x |
| 413.1566 | 1.22 | 9.80E+01 | 8.92E+01 | 9.73E+01 | 9.07E+01 | 8.64E+01 | 9.02E+01 | 9.29E+01 | 9.43E+01 | 8.76E+01 | 8.73E+01 | 8.28E+01 | 8.71E+01 | 9.20E+01 | 9.01E+01 | 1.05E+02 | 1.01E+02 | 8.75E+01 | 6.4 | x | x | x |
| 436.1600 | 1.22 | 2.15E+02 | 1.98E+02 | 2.11E+02 | 1.82E+02 | 1.86E+02 | 1.65E+02 | 1.83E+02 | 1.84E+02 | 1.71E+02 | 1.63E+02 | 1.51E+02 | 1.44E+02 | 1.49E+02 | 1.10E+02 | 1.23E+02 | 1.04E+02 | 1.04E+02 | 22.0 |  |  | x |
| 457.0904 | 1.22 | 8.34E+03 | 8.01E+03 | 8.50E+03 | 8.88E+03 | 9.30E+03 | 1.09E+04 | 9.99E+03 | 1.04E+04 | 9.96E+03 | 1.16E+04 | 1.07E+04 | 1.22E+04 | 1.27E+04 | 1.09E+04 | 1.24E+04 | 1.31E+04 | 1.29E+04 | 15.7 |  | x | x |
| 241.1551 | 1.23 | 5.15E+03 | 4.95E+03 | 4.65E+03 | 4.74E+03 | 4.61E+03 | 4.70E+03 | 4.61E+03 | 4.99E+03 | 4.71E+03 | 5.00E+03 | 4.81E+03 | 4.80E+03 | 4.68E+03 | 4.11E+03 | 4.53E+03 | 5.01E+03 | 4.85E+03 | 5.0 | x | x | x |
| 283.0611 | 1.23 | 1.12E+02 | 1.09E+02 | 1.07E+02 | 1.19E+02 | 1.14E+02 | 1.44E+02 | 9.99E+01 | 1.18E+02 | 1.21E+02 | 1.07E+02 | 1.17E+02 | 1.30E+02 | 1.25E+02 | 1.03E+02 | 1.27E+02 | 1.34E+02 | 1.16E+02 | 9.9 | x | x | x |
| 338.9344 | 1.23 | 1.96E+03 | 1.83E+03 | 1.91E+03 | 2.09E+03 | 2.03E+03 | 2.14E+03 | 2.10E+03 | 2.27E+03 | 2.17E+03 | 2.34E+03 | 2.30E+03 | 2.43E+03 | 2.63E+03 | 2.12E+03 | 2.54E+03 | 2.66E+03 | 2.64E+03 | 11.7 | x | x | x |
| 406.0115 | 1.23 | 3.38E+01 | 3.06E+01 | 4.68E+01 | 3.40E+01 | 3.82E+01 | 3.61E+01 | 2.62E+01 | 2.93E+01 | 3.40E+01 | 2.63E+01 | 3.13E+01 | 2.42E+01 | 3.00E+01 | 2.67E+01 | 3.11E+01 | 3.56E+01 | 2.55E+01 | 17.8 |  | x | x |
| 473.0852 | 1.23 | 1.78E+02 | 1.83E+02 | 1.90E+02 | 1.88E+02 | 1.84E+02 | 2.13E+02 | 1.96E+02 | 1.98E+02 | 1.92E+02 | 2.15E+02 | 1.87E+02 | 2.24E+02 | 2.27E+02 | 1.98E+02 | 2.07E+02 | 1.96E+02 | 1.84E+02 | 7.5 | x | x | x |
| 124.0875 | 1.24 | 1.11E+04 | 1.01E+04 | 1.04E+04 | 1.15E+04 | 1.16E+04 | 1.39E+04 | 1.25E+04 | 1.29E+04 | 1.24E+04 | 1.44E+04 | 1.38E+04 | 1.46E+04 | 1.52E+04 | 1.50E+04 | 1.47E+04 | 1.57E+04 | 1.52E+04 | 13.7 | x | x | x |
| 127.0397 | 1.24 | 3.77E+02 | 3.80E+02 | 3.79E+02 | 3.72E+02 | 3.67E+02 | 3.84E+02 | 3.63E+02 | 3.78E+02 | 3.68E+02 | 4.07E+02 | 3.73E+02 | 3.88E+02 | 3.83E+02 | 3.08E+02 | 3.96E+02 | 3.85E+02 | 3.52E+02 | 5.7 | x | x | x |
| 341.0274 | 1.24 | 2.53E+03 | 2.46E+03 | 2.38E+03 | 2.41E+03 | 2.45E+03 | 2.81E+03 | 2.58E+03 | 2.73E+03 | 2.68E+03 | 3.01E+03 | 2.86E+03 | 3.14E+03 | 3.27E+03 | 2.86E+03 | 3.24E+03 | 3.33E+03 | 3.23E+03 | 11.7 | x | x | x |
| 326.1096 | 1.24 | 1.67E+02 | 1.67E+02 | 1.43E+02 | 1.45E+02 | 1.22E+02 | 1.26E+02 | 1.21E+02 | 1.34E+02 | 1.49E+02 | 1.29E+02 | 1.17E+02 | 1.20E+02 | 1.25E+02 | 1.02E+02 | 1.21E+02 | 1.28E+02 | 1.02E+02 | 14.4 | x | x | x |
| 348.8581 | 1.24 | 1.67E+01 | 1.61E+01 | 1.34E+01 | 1.72E+01 | 1.96E+01 | 1.73E+01 | 1.64E+01 | 1.93E+01 | 2.18E+01 | 2.19E+01 | 1.40E+01 | 1.53E+01 | 1.89E+01 | 2.02E+01 | 1.22E+01 | 8.90E+00 | 2.63E+01 | 23.7 |  |  | x |
| 366.0589 | 1.24 | 1.73E+03 | 1.68E+03 | 1.75E+03 | 1.74E+03 | 1.80E+03 | 2.10E+03 | 1.86E+03 | 1.98E+03 | 1.96E+03 | 2.14E+03 | 2.05E+03 | 2.23E+03 | 2.35E+03 | 1.95E+03 | 2.25E+03 | 2.25E+03 | 2.21E+03 | 10.8 | x | x | x |
| 378.9837 | 1.24 | 2.56E+03 | 2.23E+03 | 2.19E+03 | 2.34E+03 | 2.36E+03 | 2.79E+03 | 2.25E+03 | 2.54E+03 | 2.47E+03 | 2.45E+03 | 2.58E+03 | 2.53E+03 | 2.87E+03 | 2.68E+03 | 2.49E+03 | 2.59E+03 | 2.62E+03 | 7.5 | x | x | x |
| 396.0684 | 1.25 | 5.98E+01 | 5.11E+01 | 5.80E+01 | 5.24E+01 | 5.51E+01 | 5.21E+01 | 4.72E+01 | 5.89E+01 | 5.78E+01 | 4.32E+01 | 6.32E+01 | 6.24E+01 | 5.69E+01 | 4.73E+01 | 5.34E+01 | 5.13E+01 | 5.70E+01 | 10.1 | x | x | x |
| 479.1230 | 1.25 | 1.77E+02 | 1.58E+02 | 1.69E+02 | 1.53E+02 | 1.47E+02 | 1.80E+02 | 1.68E+02 | 1.58E+02 | 1.43E+02 | 1.60E+02 | 1.51E+02 | 1.51E+02 | 1.39E+02 | 1.28E+02 | 1.27E+02 | 1.48E+02 | 1.25E+02 | 10.8 | x | x | x |
| 455.0763 | 1.25 | 4.06E+01 | 3.21E+01 | 3.75E+01 | 2.81E+01 | 2.75E+01 | 3.80E+01 | 3.40E+01 | 2.66E+01 | 2.50E+01 | 2.85E+01 | 2.67E+01 | 1.21E+01 | 1.09E+01 | 2.58E+01 | 2.53E+01 | 2.79E+01 | 2.06E+01 | 29.1 |  |  | x |
| 523.0032 | 1.25 | 1.45E+02 | 1.28E+02 | 1.48E+02 | 1.36E+02 | 1.38E+02 | 1.21E+02 | 1.68E+02 | 1.57E+02 | 1.42E+02 | 1.66E+02 | 1.48E+02 | 1.90E+02 | 1.80E+02 | 1.42E+02 | 1.57E+02 | 1.55E+02 | 1.36E+02 | 11.9 | x | x | x |
| 145.0481 | 1.27 | 4.09E+01 | 2.82E+01 | 2.98E+01 | 2.86E+01 | 2.16E+01 | 3.51E+01 | 3.66E+01 | 3.03E+01 | 2.29E+01 | 4.58E+01 | 3.25E+01 | 2.90E+01 | 1.97E+01 | 2.57E+01 | 2.74E+01 | 2.51E+01 | 3.26E+01 | 22.5 |  |  | x |
| 198.1576 | 1.27 | 1.14E+02 | 9.66E+01 | 1.11E+02 | 1.09E+02 | 1.03E+02 | 1.09E+02 | 9.93E+01 | 1.08E+02 | 9.76E+01 | 8.61E+01 | 8.36E+01 | 8.16E+01 | 7.98E+01 | 7.18E+01 | 5.41E+01 | 6.57E+01 | 6.40E+01 | 20.7 |  |  | x |
| 270.0857 | 1.27 | 1.11E+03 | 1.11E+03 | 1.06E+03 | 1.04E+03 | 1.03E+03 | 1.14E+03 | 1.01E+03 | 1.09E+03 | 1.10E+03 | 1.12E+03 | 1.11E+03 | 1.19E+03 | 1.20E+03 | 1.10E+03 | 1.18E+03 | 1.22E+03 | 1.17E+03 | 5.5 | x | x | x |
| 277.1025 | 1.27 | 1.49E+02 | 1.25E+02 | 1.47E+02 | 1.37E+02 | 1.24E+02 | 1.60E+02 | 1.44E+02 | 1.37E+02 | 1.27E+02 | 1.45E+02 | 1.33E+02 | 1.32E+02 | 1.33E+02 | 1.20E+02 | 1.23E+02 | 1.24E+02 | 1.13E+02 | 9.2 | x | x | x |
| 308.0416 | 1.27 | 1.43E+03 | 1.27E+03 | 1.28E+03 | 1.34E+03 | 1.33E+03 | 1.45E+03 | 1.22E+03 | 1.43E+03 | 1.36E+03 | 1.36E+03 | 1.39E+03 | 1.37E+03 | 1.50E+03 | 1.56E+03 | 1.23E+03 | 1.34E+03 | 1.25E+03 | 6.9 | x | x | x |
| 362.9934 | 1.27 | 1.48E+03 | 1.32E+03 | 1.45E+03 | 1.54E+03 | 1.64E+03 | 2.02E+03 | 1.47E+03 | 1.77E+03 | 1.77E+03 | 1.79E+03 | 1.84E+03 | 1.92E+03 | 2.10E+03 | 2.16E+03 | 1.76E+03 | 1.92E+03 | 1.81E+03 | 13.7 | x | x | x |
| 427.1363 | 1.27 | 8.85E+01 | 8.73E+01 | 9.70E+01 | 7.44E+01 | 8.32E+01 | 7.64E+01 | 7.01E+01 | 7.97E+01 | 7.47E+01 | 8.10E+01 | 7.22E+01 | 7.56E+01 | 7.40E+01 | 5.91E+01 | 7.17E+01 | 7.04E+01 | 7.05E+01 | 11.4 | x | x | x |
| 441.2592 | 1.27 | 2.50E+02 | 2.25E+02 | 2.31E+02 | 2.35E+02 | 2.37E+02 | 1.89E+02 | 2.32E+02 | 1.95E+02 | 2.11E+02 | 2.21E+02 | 2.17E+02 | 2.24E+02 | 1.70E+02 | 1.47E+02 | 2.24E+02 | 1.94E+02 | 1.64E+02 | 13.8 | x | x | x |
| 121.0407 | 1.28 | 7.64E+01 | 7.81E+01 | 7.78E+01 | 7.80E+01 | 8.14E+01 | 1.04E+02 | 8.55E+01 | 7.93E+01 | 8.85E+01 | 8.57E+01 | 8.40E+01 | 1.02E+02 | 1.01E+02 | 1.01E+02 | 8.96E+01 | 1.02E+02 | 9.10E+01 | 11.3 | x | x | x |
| 324.9188 | 1.28 | 2.66E+02 | 2.21E+02 | 2.29E+02 | 2.41E+02 | 2.62E+02 | 3.08E+02 | 2.56E+02 | 2.92E+02 | 2.80E+02 | 2.92E+02 | 2.72E+02 | 3.07E+02 | 3.19E+02 | 3.06E+02 | 2.88E+02 | 2.98E+02 | 2.97E+02 | 10.4 | x | x | x |
| 362.8744 | 1.28 | 2.16E+01 | 1.66E+01 | 1.88E+01 | 2.04E+01 | 2.16E+01 | 2.42E+01 | 2.16E+01 | 2.02E+01 | 2.60E+01 | 2.12E+01 | 2.46E+01 | 2.70E+01 | 2.75E+01 | 3.42E+01 | 1.64E+01 | 3.17E+01 | 2.42E+01 | 20.7 |  |  | x |
| 377.0044 | 1.28 | 1.10E+02 | 9.16E+01 | 9.25E+01 | 9.97E+01 | 9.24E+01 | 1.01E+02 | 9.36E+01 | 1.06E+02 | 1.03E+02 | 9.81E+01 | 1.16E+02 | 1.07E+02 | 1.19E+02 | 1.14E+02 | 1.01E+02 | 1.19E+02 | 9.88E+01 | 9.0 | x | x | x |
| 391.0167 | 1.28 | 2.74E+02 | 2.50E+02 | 2.46E+02 | 2.42E+02 | 2.45E+02 | 2.30E+02 | 2.20E+02 | 2.57E+02 | 2.58E+02 | 2.36E+02 | 2.54E+02 | 2.53E+02 | 2.95E+02 | 2.49E+02 | 2.35E+02 | 2.63E+02 | 2.78E+02 | 7.4 | x | x | x |
| 511.0084 | 1.28 | 2.28E+01 | 2.59E+01 | 2.06E+01 | 1.68E+01 | 2.66E+01 | 1.39E+01 | 2.57E+01 | 2.27E+01 | 2.11E+01 | 2.45E+01 | 1.97E+01 | 2.58E+01 | 2.74E+01 | 2.58E+01 | 2.61E+01 | 2.21E+01 | 1.23E+01 | 20.2 |  |  | x |
| 209.0577 | 1.30 | 2.68E+03 | 2.21E+03 | 2.45E+03 | 2.73E+03 | 2.61E+03 | 3.79E+03 | 2.93E+03 | 2.95E+03 | 2.79E+03 | 3.22E+03 | 3.09E+03 | 3.18E+03 | 3.16E+03 | 3.22E+03 | 3.04E+03 | 3.33E+03 | 3.24E+03 | 12.5 | x | x | x |
| 256.0698 | 1.30 | 3.40E+02 | 3.30E+02 | 3.47E+02 | 3.10E+02 | 3.00E+02 | 3.04E+02 | 2.89E+02 | 2.97E+02 | 2.96E+02 | 3.11E+02 | 2.78E+02 | 3.01E+02 | 3.14E+02 | 2.74E+02 | 2.93E+02 | 2.94E+02 | 2.83E+02 | 6.7 | x | x | x |
| 294.0260 | 1.30 | 6.22E+02 | 5.85E+02 | 5.52E+02 | 6.31E+02 | 6.03E+02 | 6.36E+02 | 6.11E+02 | 6.67E+02 | 6.29E+02 | 6.17E+02 | 6.65E+02 | 6.73E+02 | 7.41E+02 | 7.66E+02 | 6.75E+02 | 7.83E+02 | 6.87E+02 | 9.5 | x | x | x |
| 362.9894 | 1.30 | 1.63E+03 | 1.42E+03 | 1.35E+03 | 1.54E+03 | 1.45E+03 | 1.76E+03 | 1.65E+03 | 1.74E+03 | 1.63E+03 | 1.80E+03 | 1.77E+03 | 1.77E+03 | 2.00E+03 | 2.14E+03 | 1.82E+03 | 2.11E+03 | 1.85E+03 | 12.9 | x | x | x |
| 396.0695 | 1.30 | 5.01E+01 | 4.56E+01 | 5.70E+01 | 5.32E+01 | 4.58E+01 | 5.36E+01 | 3.45E+01 | 4.71E+01 | 5.38E+01 | 3.14E+01 | 4.42E+01 | 5.82E+01 | 5.17E+01 | 4.11E+01 | 3.35E+01 | 4.36E+01 | 4.70E+01 | 17.2 |  | x | x |
| 431.0974 | 1.30 | 9.34E+02 | 8.69E+02 | 9.04E+02 | 9.28E+02 | 9.15E+02 | 1.06E+03 | 9.46E+02 | 9.77E+02 | 8.76E+02 | 9.62E+02 | 8.81E+02 | 9.66E+02 | 8.82E+02 | 7.93E+02 | 8.51E+02 | 8.38E+02 | 8.16E+02 | 7.3 | x | x | x |
| 461.1134 | 1.30 | 1.91E+02 | 1.88E+02 | 1.84E+02 | 1.90E+02 | 1.89E+02 | 1.99E+02 | 1.85E+02 | 2.02E+02 | 1.85E+02 | 1.74E+02 | 1.57E+02 | 1.80E+02 | 1.73E+02 | 1.65E+02 | 1.59E+02 | 1.51E+02 | 1.47E+02 | 9.3 | x | x | x |
| 499.0641 | 1.30 | 3.01E+02 | 3.08E+02 | 3.22E+02 | 3.20E+02 | 2.93E+02 | 3.16E+02 | 3.74E+02 | 3.71E+02 | 3.19E+02 | 3.73E+02 | 3.48E+02 | 3.76E+02 | 3.31E+02 | 3.34E+02 | 3.35E+02 | 3.53E+02 | 3.85E+02 | 8.5 | x | x | x |
| 507.0095 | 1.30 | 6.16E+02 | 5.89E+02 | 6.34E+02 | 6.35E+02 | 6.68E+02 | 7.07E+02 | 7.47E+02 | 7.58E+02 | 7.21E+02 | 8.13E+02 | 7.68E+02 | 8.46E+02 | 8.62E+02 | 8.63E+02 | 8.31E+02 | 8.44E+02 | 8.38E+02 | 12.6 | x | x | x |
| 590.9743 | 1.30 | 8.23E+01 | 7.94E+01 | 7.41E+01 | 8.31E+01 | 8.79E+01 | 7.93E+01 | 9.18E+01 | 9.61E+01 | 9.30E+01 | 9.64E+01 | 8.93E+01 | 9.73E+01 | 1.02E+02 | 9.72E+01 | 9.24E+01 | 8.81E+01 | 9.59E+01 | 8.7 | x | x | x |
| 74.0718 | 1.31 | 9.78E+00 | 9.72E+00 | 1.10E+01 | 1.31E+01 | 1.21E+01 | 4.44E+00 | 8.07E+00 | 9.77E+00 | 1.18E+01 | 8.11E+00 | 9.62E+00 | 1.46E+01 | 1.37E+01 | 1.46E+01 | 1.78E+01 | 1.48E+01 | 8.80E+00 | 28.9 |  |  | x |
| 276.9186 | 1.31 | 2.75E+01 | 2.93E+01 | 3.27E+01 | 3.61E+01 | 2.67E+01 | 3.29E+01 | 3.44E+01 | 2.94E+01 | 2.74E+01 | 1.99E+01 | 2.83E+01 | 2.69E+01 | 2.13E+01 | 2.90E+01 | 2.86E+01 | 2.63E+01 | 2.93E+01 | 14.4 | x | x | x |
| 406.0488 | 1.31 | 2.13E+02 | 1.59E+02 | 1.69E+02 | 1.79E+02 | 1.64E+02 | 1.66E+02 | 1.61E+02 | 1.70E+02 | 1.69E+02 | 1.51E+02 | 1.53E+02 | 1.64E+02 | 1.88E+02 | 1.90E+02 | 1.41E+02 | 1.41E+02 | 1.27E+02 | 12.4 | x | x | x |
| 436.0433 | 1.31 | 5.27E+01 | 3.97E+01 | 3.47E+01 | 4.80E+01 | 4.44E+01 | 4.38E+01 | 3.89E+01 | 4.19E+01 | 3.23E+01 | 3.91E+01 | 2.69E+01 | 3.73E+01 | 2.26E+01 | 3.59E+01 | 2.21E+01 | 2.84E+01 | 1.86E+01 | 26.7 |  |  | x |
| 461.1762 | 1.31 | 8.25E+01 | 8.01E+01 | 7.99E+01 | 8.54E+01 | 9.14E+01 | 9.09E+01 | 8.27E+01 | 9.97E+01 | 8.87E+01 | 9.61E+01 | 8.89E+01 | 9.04E+01 | 9.46E+01 | 8.71E+01 | 9.63E+01 | 1.08E+02 | 8.00E+01 | 8.7 | x | x | x |
| 92.0500 | 1.32 | 1.52E+02 | 1.39E+02 | 1.52E+02 | 1.47E+02 | 1.42E+02 | 1.74E+02 | 1.37E+02 | 1.53E+02 | 1.43E+02 | 1.57E+02 | 1.40E+02 | 1.48E+02 | 1.40E+02 | 1.48E+02 | 1.25E+02 | 1.43E+02 | 1.50E+02 | 7.1 | x | x | x |
| 94.0656 | 1.32 | 1.23E+03 | 1.18E+03 | 1.24E+03 | 1.28E+03 | 1.31E+03 | 1.57E+03 | 1.36E+03 | 1.42E+03 | 1.35E+03 | 1.52E+03 | 1.42E+03 | 1.55E+03 | 1.55E+03 | 1.48E+03 | 1.44E+03 | 1.54E+03 | 1.50E+03 | 9.0 | x | x | x |
| 109.0764 | 1.32 | 1.05E+02 | 9.33E+01 | 1.06E+02 | 1.09E+02 | 9.97E+01 | 1.06E+02 | 1.01E+02 | 1.04E+02 | 1.05E+02 | 1.11E+02 | 8.82E+01 | 9.91E+01 | 9.90E+01 | 9.49E+01 | 9.43E+01 | 9.25E+01 | 9.04E+01 | 6.8 | x | x | x |
| 160.1327 | 1.32 | 4.82E+03 | 4.50E+03 | 4.73E+03 | 5.16E+03 | 5.31E+03 | 6.82E+03 | 5.48E+03 | 5.90E+03 | 5.68E+03 | 6.43E+03 | 6.10E+03 | 6.65E+03 | 7.08E+03 | 6.90E+03 | 6.80E+03 | 7.47E+03 | 7.28E+03 | 15.7 |  | x | x |
| 169.0990 | 1.32 | 1.17E+02 | 1.08E+02 | 1.11E+02 | 1.08E+02 | 9.38E+01 | 8.37E+01 | 8.85E+01 | 1.05E+02 | 8.44E+01 | 7.70E+01 | 7.44E+01 | 8.48E+01 | 1.10E+02 | 7.15E+01 | 7.88E+01 | 7.94E+01 | 7.48E+01 | 16.8 |  | x | x |
| 137.0715 | 1.32 | 1.56E+05 | 1.46E+05 | 1.52E+05 | 1.66E+05 | 1.65E+05 | 2.06E+05 | 1.78E+05 | 1.84E+05 | 1.76E+05 | 2.09E+05 | 1.95E+05 | 2.12E+05 | 2.29E+05 | 2.15E+05 | 2.19E+05 | 2.36E+05 | 2.34E+05 | 15.4 |  | x | x |
| 168.1130 | 1.32 | 6.41E+02 | 5.61E+02 | 5.91E+02 | 6.26E+02 | 6.18E+02 | 7.08E+02 | 6.32E+02 | 6.56E+02 | 6.37E+02 | 6.48E+02 | 6.24E+02 | 6.59E+02 | 6.90E+02 | 6.14E+02 | 6.74E+02 | 6.86E+02 | 6.79E+02 | 5.9 | x | x | x |
| 230.0356 | 1.32 | 4.16E+02 | 3.50E+02 | 4.04E+02 | 4.44E+02 | 4.23E+02 | 5.54E+02 | 4.44E+02 | 4.60E+02 | 4.35E+02 | 4.43E+02 | 4.47E+02 | 4.59E+02 | 4.99E+02 | 4.45E+02 | 4.31E+02 | 4.59E+02 | 4.53E+02 | 9.3 | x | x | x |
| 232.0338 | 1.32 | 1.23E+02 | 9.57E+01 | 1.20E+02 | 1.26E+02 | 1.21E+02 | 1.47E+02 | 1.27E+02 | 1.29E+02 | 1.24E+02 | 1.21E+02 | 1.11E+02 | 1.28E+02 | 1.47E+02 | 1.18E+02 | 1.18E+02 | 1.29E+02 | 1.06E+02 | 10.2 | x | x | x |
| 420.0272 | 1.32 | 1.34E+02 | 1.13E+02 | 1.07E+02 | 1.11E+02 | 1.00E+02 | 9.00E+01 | 1.22E+02 | 1.00E+02 | 1.08E+02 | 7.52E+01 | 8.57E+01 | 8.97E+01 | 1.24E+02 | 1.18E+02 | 8.79E+01 | 8.74E+01 | 1.04E+02 | 15.5 |  | x | x |
| 469.0537 | 1.32 | 8.30E+02 | 8.00E+02 | 8.11E+02 | 8.27E+02 | 8.40E+02 | 9.81E+02 | 9.07E+02 | 9.50E+02 | 9.08E+02 | 9.94E+02 | 9.42E+02 | 1.06E+03 | 1.06E+03 | 9.46E+02 | 1.01E+03 | 1.05E+03 | 1.06E+03 | 9.8 | x | x | x |
| 162.1131 | 1.33 | 7.50E+02 | 6.97E+02 | 7.10E+02 | 7.25E+02 | 6.73E+02 | 7.17E+02 | 6.90E+02 | 7.16E+02 | 6.78E+02 | 7.36E+02 | 6.96E+02 | 7.39E+02 | 7.80E+02 | 7.30E+02 | 7.33E+02 | 7.90E+02 | 7.56E+02 | 4.5 | x | x | x |
| 234.9639 | 1.33 | 4.28E+02 | 3.77E+02 | 4.29E+02 | 4.16E+02 | 4.86E+02 | 7.33E+02 | 5.17E+02 | 5.40E+02 | 5.32E+02 | 5.59E+02 | 5.69E+02 | 5.84E+02 | 6.56E+02 | 8.43E+02 | 6.93E+02 | 7.93E+02 | 8.15E+02 | 24.9 |  |  | x |
| 259.2019 | 1.33 | 2.25E+02 | 1.93E+02 | 1.86E+02 | 2.10E+02 | 1.94E+02 | 2.06E+02 | 2.12E+02 | 2.14E+02 | 2.10E+02 | 2.12E+02 | 1.94E+02 | 2.14E+02 | 2.33E+02 | 2.06E+02 | 1.97E+02 | 2.01E+02 | 1.84E+02 | 6.4 | x | x | x |
| 262.1643 | 1.33 | 6.52E+01 | 6.73E+01 | 7.99E+01 | 8.77E+01 | 9.18E+01 | 1.14E+02 | 8.23E+01 | 9.34E+01 | 9.49E+01 | 8.80E+01 | 8.68E+01 | 1.07E+02 | 1.10E+02 | 8.66E+01 | 1.06E+02 | 1.11E+02 | 1.12E+02 | 16.2 |  | x | x |
| 236.9621 | 1.33 | 2.54E+01 | 2.26E+01 | 1.96E+01 | 2.19E+01 | 3.11E+01 | 4.68E+01 | 2.88E+01 | 2.96E+01 | 3.04E+01 | 2.79E+01 | 2.72E+01 | 2.88E+01 | 3.36E+01 | 4.14E+01 | 3.85E+01 | 3.65E+01 | 2.34E+01 | 24.2 |  |  | x |
| 411.0672 | 1.33 | 5.17E+01 | 2.36E+01 | 3.16E+01 | 3.35E+01 | 2.62E+01 | 2.06E+01 | 3.82E+01 | 3.71E+01 | 2.32E+01 | 3.27E+01 | 3.32E+01 | 2.32E+01 | 3.46E+01 | 3.91E+01 | 2.26E+01 | 2.80E+01 | 2.76E+01 | 25.6 |  |  | x |
| 430.9638 | 1.33 | 5.55E+01 | 4.29E+01 | 4.66E+01 | 4.50E+01 | 5.21E+01 | 8.73E+01 | 5.97E+01 | 6.16E+01 | 5.26E+01 | 5.84E+01 | 6.50E+01 | 5.90E+01 | 7.20E+01 | 8.93E+01 | 7.09E+01 | 8.12E+01 | 7.89E+01 | 22.8 |  |  | x |
| 343.0215 | 1.34 | 9.11E+01 | 9.13E+01 | 8.78E+01 | 6.75E+01 | 8.45E+01 | 8.32E+01 | 8.42E+01 | 9.23E+01 | 8.46E+01 | 8.26E+01 | 7.92E+01 | 6.33E+01 | 7.77E+01 | 8.84E+01 | 7.01E+01 | 8.11E+01 | 6.19E+01 | 11.9 | x | x | x |
| 350.0864 | 1.34 | 6.01E+02 | 5.36E+02 | 5.51E+02 | 5.65E+02 | 4.96E+02 | 5.45E+02 | 5.21E+02 | 5.43E+02 | 5.74E+02 | 5.18E+02 | 4.76E+02 | 5.47E+02 | 5.04E+02 | 5.17E+02 | 4.55E+02 | 4.80E+02 | 4.28E+02 | 8.5 | x | x | x |
| 318.9250 | 1.34 | 9.40E+01 | 7.63E+01 | 7.84E+01 | 7.26E+01 | 7.96E+01 | 1.18E+02 | 9.35E+01 | 9.73E+01 | 8.94E+01 | 9.32E+01 | 8.67E+01 | 7.81E+01 | 8.70E+01 | 1.04E+02 | 8.44E+01 | 1.04E+02 | 9.80E+01 | 13.1 | x | x | x |
| 372.2377 | 1.34 | 3.14E+02 | 2.84E+02 | 2.64E+02 | 2.65E+02 | 2.54E+02 | 2.96E+02 | 2.61E+02 | 2.62E+02 | 2.49E+02 | 2.54E+02 | 2.30E+02 | 2.48E+02 | 2.60E+02 | 2.18E+02 | 2.23E+02 | 2.40E+02 | 2.38E+02 | 9.7 | x | x | x |
| 414.9896 | 1.34 | 2.29E+02 | 2.37E+02 | 3.60E+02 | 3.85E+02 | 4.31E+02 | 6.08E+02 | 2.42E+02 | 4.29E+02 | 4.25E+02 | 2.87E+02 | 3.74E+02 | 4.27E+02 | 4.85E+02 | 4.07E+02 | 4.43E+02 | 4.59E+02 | 3.99E+02 | 25.1 |  |  | x |
| 213.1584 | 1.35 | 5.81E+01 | 5.27E+01 | 6.33E+01 | 5.03E+01 | 4.24E+01 | 3.80E+01 | 5.06E+01 | 4.68E+01 | 4.22E+01 | 3.45E+01 | 3.64E+01 | 3.80E+01 | 4.04E+01 | 2.68E+01 | 2.92E+01 | 4.31E+01 | 2.97E+01 | 24.0 |  |  | x |
| 153.0412 | 1.35 | 5.19E+01 | 4.03E+01 | 4.36E+01 | 3.87E+01 | 3.62E+01 | 4.09E+01 | 4.16E+01 | 4.02E+01 | 4.01E+01 | 4.12E+01 | 4.00E+01 | 3.34E+01 | 3.79E+01 | 2.91E+01 | 4.16E+01 | 2.67E+01 | 2.97E+01 | 15.7 |  | x | x |
| 181.1069 | 1.35 | 8.82E+02 | 7.95E+02 | 8.52E+02 | 8.76E+02 | 8.57E+02 | 1.04E+03 | 9.11E+02 | 9.11E+02 | 8.69E+02 | 9.56E+02 | 9.24E+02 | 9.44E+02 | 9.57E+02 | 8.21E+02 | 8.64E+02 | 9.48E+02 | 8.63E+02 | 6.6 | x | x | x |
| 208.9367 | 1.35 | 2.51E+02 | 2.34E+02 | 2.32E+02 | 2.54E+02 | 2.61E+02 | 2.70E+02 | 2.94E+02 | 3.12E+02 | 2.95E+02 | 3.34E+02 | 3.00E+02 | 3.37E+02 | 3.36E+02 | 2.80E+02 | 2.79E+02 | 3.04E+02 | 2.62E+02 | 11.8 | x | x | x |
| 271.9763 | 1.35 | 2.58E+01 | 2.32E+01 | 1.76E+01 | 2.24E+01 | 2.32E+01 | 1.92E+01 | 3.10E+01 | 2.56E+01 | 2.31E+01 | 3.03E+01 | 2.70E+01 | 2.29E+01 | 1.61E+01 | 1.97E+01 | 2.57E+01 | 2.70E+01 | 2.53E+01 | 17.1 |  | x | x |
| 336.0709 | 1.35 | 6.48E+02 | 5.75E+02 | 5.76E+02 | 6.20E+02 | 6.31E+02 | 6.79E+02 | 6.58E+02 | 6.94E+02 | 7.09E+02 | 7.20E+02 | 7.26E+02 | 7.08E+02 | 7.84E+02 | 7.96E+02 | 7.06E+02 | 7.63E+02 | 6.84E+02 | 9.4 | x | x | x |
| 378.9110 | 1.35 | 1.50E+02 | 1.47E+02 | 1.55E+02 | 1.61E+02 | 1.73E+02 | 1.74E+02 | 1.73E+02 | 1.91E+02 | 1.97E+02 | 2.14E+02 | 1.86E+02 | 2.24E+02 | 2.28E+02 | 1.86E+02 | 1.71E+02 | 1.68E+02 | 1.67E+02 | 13.5 | x | x | x |
| 511.9972 | 1.35 | 2.14E+02 | 2.15E+02 | 2.16E+02 | 2.31E+02 | 2.52E+02 | 3.03E+02 | 2.47E+02 | 2.45E+02 | 2.74E+02 | 3.09E+02 | 2.44E+02 | 3.11E+02 | 3.39E+02 | 2.78E+02 | 2.94E+02 | 3.36E+02 | 2.87E+02 | 15.2 |  | x | x |
| 513.9947 | 1.35 | 1.39E+02 | 1.37E+02 | 1.43E+02 | 1.52E+02 | 1.57E+02 | 1.79E+02 | 1.61E+02 | 1.64E+02 | 1.69E+02 | 1.97E+02 | 1.41E+02 | 1.93E+02 | 2.02E+02 | 1.72E+02 | 1.79E+02 | 1.98E+02 | 1.61E+02 | 12.9 | x | x | x |
| 311.1603 | 1.36 | 6.22E+02 | 6.06E+02 | 5.97E+02 | 5.92E+02 | 5.51E+02 | 6.09E+02 | 5.31E+02 | 5.54E+02 | 5.48E+02 | 6.06E+02 | 5.33E+02 | 6.18E+02 | 7.12E+02 | 6.32E+02 | 6.27E+02 | 6.58E+02 | 5.94E+02 | 7.8 | x | x | x |
| 392.0738 | 1.36 | 2.08E+01 | 2.27E+01 | 2.67E+01 | 3.01E+01 | 2.41E+01 | 2.45E+01 | 2.37E+01 | 2.52E+01 | 2.90E+01 | 2.24E+01 | 2.86E+01 | 3.11E+01 | 2.27E+01 | 2.56E+01 | 2.06E+01 | 2.15E+01 | 1.87E+01 | 14.5 | x | x | x |
| 416.8628 | 1.36 | 7.71E+01 | 6.39E+01 | 5.83E+01 | 6.48E+01 | 7.30E+01 | 3.61E+01 | 7.16E+01 | 7.15E+01 | 7.26E+01 | 7.10E+01 | 5.98E+01 | 6.20E+01 | 6.12E+01 | 7.12E+01 | 4.46E+01 | 4.59E+01 | 3.85E+01 | 20.8 |  |  | x |
| 126.0668 | 1.37 | 1.00E+01 | 9.44E+00 | 1.16E+01 | 1.02E+01 | 1.14E+01 | 1.06E+01 | 9.63E+00 | 1.11E+01 | 1.31E+01 | 1.08E+01 | 1.07E+01 | 1.15E+01 | 9.27E+00 | 1.39E+01 | 1.18E+01 | 8.94E+00 | 8.29E+00 | 13.5 | x | x | x |
| 373.8596 | 1.37 | 6.17E+01 | 6.28E+01 | 6.25E+01 | 5.93E+01 | 6.02E+01 | 5.29E+01 | 7.18E+01 | 7.67E+01 | 7.24E+01 | 8.52E+01 | 6.98E+01 | 6.79E+01 | 6.98E+01 | 7.48E+01 | 3.71E+01 | 8.37E+01 | 5.32E+01 | 18.1 |  | x | x |
| 485.0483 | 1.37 | 2.71E+01 | 2.76E+01 | 3.07E+01 | 2.76E+01 | 2.67E+01 | 3.16E+01 | 3.16E+01 | 3.69E+01 | 3.66E+01 | 2.41E+01 | 2.53E+01 | 4.23E+01 | 3.15E+01 | 2.72E+01 | 3.09E+01 | 2.74E+01 | 2.54E+01 | 16.1 |  | x | x |
| 114.0668 | 1.38 | 4.94E+03 | 4.26E+03 | 4.65E+03 | 5.44E+03 | 5.51E+03 | 8.25E+03 | 5.87E+03 | 6.18E+03 | 6.02E+03 | 6.99E+03 | 6.79E+03 | 7.26E+03 | 8.21E+03 | 7.62E+03 | 8.21E+03 | 8.87E+03 | 8.69E+03 | 22.0 |  |  | x |
| 175.9882 | 1.38 | 2.31E+01 | 2.23E+01 | 2.44E+01 | 2.79E+01 | 2.87E+01 | 2.99E+01 | 2.33E+01 | 1.99E+01 | 2.87E+01 | 2.31E+01 | 2.09E+01 | 2.26E+01 | 1.90E+01 | 2.21E+01 | 1.71E+01 | 1.56E+01 | 1.32E+01 | 20.9 |  |  | x |
| 289.0478 | 1.38 | 3.20E+03 | 3.04E+03 | 3.14E+03 | 3.40E+03 | 3.29E+03 | 3.98E+03 | 3.23E+03 | 3.29E+03 | 3.21E+03 | 3.52E+03 | 3.32E+03 | 3.57E+03 | 3.90E+03 | 3.44E+03 | 3.70E+03 | 3.87E+03 | 3.82E+03 | 8.5 | x | x | x |
| 291.0463 | 1.38 | 1.17E+03 | 1.11E+03 | 1.16E+03 | 1.20E+03 | 1.12E+03 | 1.31E+03 | 1.11E+03 | 1.11E+03 | 1.11E+03 | 1.15E+03 | 1.10E+03 | 1.17E+03 | 1.26E+03 | 1.11E+03 | 1.18E+03 | 1.22E+03 | 1.18E+03 | 5.1 | x | x | x |
| 425.0845 | 1.38 | 5.50E+01 | 5.88E+01 | 5.10E+01 | 4.21E+01 | 4.22E+01 | 3.02E+01 | 3.09E+01 | 3.26E+01 | 3.75E+01 | 3.12E+01 | 3.21E+01 | 2.52E+01 | 3.71E+01 | 2.86E+01 | 2.56E+01 | 2.60E+01 | 2.29E+01 | 30.0 |  |  | x |
| 307.0449 | 1.39 | 1.60E+02 | 1.35E+02 | 1.28E+02 | 1.17E+02 | 1.20E+02 | 1.29E+02 | 1.25E+02 | 1.31E+02 | 1.33E+02 | 1.60E+02 | 1.28E+02 | 1.28E+02 | 1.05E+02 | 1.27E+02 | 1.15E+02 | 1.21E+02 | 1.02E+02 | 12.0 | x | x | x |
| 126.0917 | 1.40 | 1.51E+01 | 1.26E+01 | 1.55E+01 | 2.24E+01 | 1.62E+01 | 2.17E+01 | 1.45E+01 | 1.73E+01 | 1.60E+01 | 1.46E+01 | 1.57E+01 | 1.53E+01 | 1.64E+01 | 1.15E+01 | 1.72E+01 | 1.16E+01 | 1.46E+01 | 18.4 |  | x | x |
| 149.0462 | 1.40 | 1.89E+01 | 1.54E+01 | 2.04E+01 | 2.06E+01 | 2.16E+01 | 2.48E+01 | 1.70E+01 | 2.06E+01 | 1.87E+01 | 1.91E+01 | 2.24E+01 | 1.95E+01 | 2.20E+01 | 2.36E+01 | 2.54E+01 | 3.67E+01 | 3.31E+01 | 24.4 |  |  | x |
| 165.9309 | 1.40 | 5.96E+01 | 5.58E+01 | 6.39E+01 | 6.20E+01 | 7.24E+01 | 6.27E+01 | 7.00E+01 | 7.23E+01 | 6.92E+01 | 7.93E+01 | 7.54E+01 | 6.79E+01 | 7.42E+01 | 7.01E+01 | 5.78E+01 | 6.16E+01 | 5.85E+01 | 10.4 | x | x | x |
| 172.1183 | 1.40 | 4.47E+01 | 4.71E+01 | 4.82E+01 | 4.85E+01 | 4.39E+01 | 5.64E+01 | 4.86E+01 | 4.78E+01 | 4.26E+01 | 5.44E+01 | 4.69E+01 | 4.43E+01 | 4.38E+01 | 4.18E+01 | 4.32E+01 | 3.95E+01 | 3.98E+01 | 9.9 | x | x | x |
| 189.9786 | 1.40 | 4.41E+01 | 4.17E+01 | 4.37E+01 | 4.87E+01 | 4.55E+01 | 3.33E+01 | 3.60E+01 | 4.22E+01 | 4.14E+01 | 4.19E+01 | 4.00E+01 | 3.38E+01 | 3.73E+01 | 3.57E+01 | 3.78E+01 | 3.88E+01 | 3.15E+01 | 11.8 | x | x | x |
| 202.0270 | 1.40 | 4.52E+01 | 3.88E+01 | 4.16E+01 | 4.38E+01 | 4.56E+01 | 5.89E+01 | 3.83E+01 | 4.82E+01 | 4.17E+01 | 5.70E+01 | 4.47E+01 | 4.43E+01 | 4.73E+01 | 4.30E+01 | 4.54E+01 | 3.97E+01 | 3.89E+01 | 12.9 | x | x | x |
| 203.8870 | 1.40 | 9.16E+02 | 9.01E+02 | 9.59E+02 | 9.36E+02 | 1.01E+03 | 1.12E+03 | 1.05E+03 | 1.12E+03 | 1.09E+03 | 1.18E+03 | 1.12E+03 | 1.24E+03 | 1.31E+03 | 1.22E+03 | 1.21E+03 | 1.28E+03 | 1.21E+03 | 11.7 | x | x | x |
| 243.9450 | 1.40 | 2.66E+01 | 3.19E+01 | 3.24E+01 | 3.64E+01 | 2.95E+01 | 2.03E+01 | 2.91E+01 | 3.16E+01 | 2.95E+01 | 2.46E+01 | 2.71E+01 | 3.23E+01 | 3.67E+01 | 3.19E+01 | 3.15E+01 | 3.74E+01 | 3.42E+01 | 14.5 | x | x | x |
| 246.8921 | 1.40 | 6.09E+02 | 5.40E+02 | 5.14E+02 | 5.25E+02 | 5.34E+02 | 7.89E+02 | 6.65E+02 | 6.40E+02 | 6.03E+02 | 7.52E+02 | 6.43E+02 | 6.58E+02 | 6.65E+02 | 8.60E+02 | 6.82E+02 | 8.13E+02 | 7.79E+02 | 16.0 |  | x | x |
| 280.1652 | 1.40 | 3.37E+02 | 2.88E+02 | 3.08E+02 | 3.27E+02 | 2.95E+02 | 2.88E+02 | 3.03E+02 | 2.79E+02 | 2.94E+02 | 2.97E+02 | 2.78E+02 | 2.60E+02 | 2.93E+02 | 2.71E+02 | 2.63E+02 | 2.86E+02 | 2.52E+02 | 7.6 | x | x | x |
| 290.1507 | 1.40 | 7.82E+01 | 6.77E+01 | 7.40E+01 | 8.04E+01 | 7.14E+01 | 7.84E+01 | 6.49E+01 | 7.67E+01 | 8.07E+01 | 7.95E+01 | 7.73E+01 | 7.88E+01 | 7.99E+01 | 7.21E+01 | 8.40E+01 | 7.98E+01 | 7.57E+01 | 6.5 | x | x | x |
| 378.0586 | 1.40 | 4.32E+02 | 3.83E+02 | 3.86E+02 | 4.38E+02 | 4.12E+02 | 6.13E+02 | 4.39E+02 | 4.72E+02 | 4.58E+02 | 5.20E+02 | 4.83E+02 | 4.86E+02 | 5.37E+02 | 4.75E+02 | 4.81E+02 | 5.38E+02 | 4.54E+02 | 12.4 | x | x | x |
| 345.0005 | 1.40 | 6.70E+01 | 6.36E+01 | 6.12E+01 | 6.23E+01 | 6.49E+01 | 6.14E+01 | 6.94E+01 | 6.07E+01 | 6.55E+01 | 6.99E+01 | 6.06E+01 | 5.94E+01 | 6.80E+01 | 6.39E+01 | 5.61E+01 | 7.18E+01 | 6.03E+01 | 6.7 | x | x | x |
| 379.9661 | 1.40 | 6.69E+01 | 5.26E+01 | 5.85E+01 | 5.14E+01 | 5.66E+01 | 5.53E+01 | 6.32E+01 | 5.47E+01 | 4.97E+01 | 5.53E+01 | 4.25E+01 | 4.87E+01 | 5.40E+01 | 4.61E+01 | 4.21E+01 | 3.96E+01 | 4.57E+01 | 14.3 | x | x | x |
| 386.0439 | 1.40 | 1.35E+02 | 1.60E+02 | 1.20E+02 | 1.33E+02 | 1.18E+02 | 1.26E+02 | 1.35E+02 | 1.44E+02 | 1.38E+02 | 1.42E+02 | 1.24E+02 | 1.51E+02 | 1.53E+02 | 1.41E+02 | 1.44E+02 | 1.56E+02 | 1.57E+02 | 9.3 | x | x | x |
| 414.8182 | 1.40 | 9.13E+01 | 7.66E+01 | 7.79E+01 | 6.87E+01 | 8.76E+01 | 8.35E+01 | 9.01E+01 | 9.82E+01 | 8.39E+01 | 1.05E+02 | 9.41E+01 | 9.08E+01 | 8.54E+01 | 1.06E+02 | 8.58E+01 | 9.59E+01 | 8.72E+01 | 10.9 | x | x | x |
| 458.7713 | 1.40 | 5.41E+01 | 4.79E+01 | 4.89E+01 | 4.52E+01 | 3.51E+01 | 2.56E+01 | 4.40E+01 | 3.78E+01 | 3.05E+01 | 3.19E+01 | 2.81E+01 | 2.67E+01 | 2.24E+01 | 3.81E+01 | 2.09E+01 | 2.07E+01 | 3.58E+01 | 29.7 |  |  | x |
| 167.0918 | 1.41 | 1.01E+03 | 9.09E+02 | 9.91E+02 | 1.09E+03 | 1.10E+03 | 1.44E+03 | 1.12E+03 | 1.18E+03 | 1.11E+03 | 1.26E+03 | 1.21E+03 | 1.32E+03 | 1.46E+03 | 1.36E+03 | 1.44E+03 | 1.59E+03 | 1.53E+03 | 16.4 |  | x | x |
| 196.8572 | 1.41 | 2.49E+01 | 2.22E+01 | 2.26E+01 | 2.48E+01 | 2.70E+01 | 3.01E+01 | 3.06E+01 | 2.22E+01 | 2.31E+01 | 2.73E+01 | 2.14E+01 | 3.00E+01 | 2.49E+01 | 1.72E+01 | 2.48E+01 | 1.79E+01 | 1.94E+01 | 16.7 |  | x | x |
| 214.1191 | 1.41 | 4.71E+01 | 3.70E+01 | 3.62E+01 | 4.41E+01 | 3.98E+01 | 4.39E+01 | 4.48E+01 | 4.78E+01 | 3.47E+01 | 4.16E+01 | 4.01E+01 | 3.63E+01 | 4.21E+01 | 2.58E+01 | 3.63E+01 | 3.79E+01 | 3.43E+01 | 14.0 | x | x | x |
| 245.1607 | 1.41 | 6.33E+03 | 6.02E+03 | 6.16E+03 | 6.63E+03 | 6.63E+03 | 8.57E+03 | 6.79E+03 | 7.09E+03 | 6.80E+03 | 7.78E+03 | 7.49E+03 | 7.97E+03 | 9.18E+03 | 8.34E+03 | 9.06E+03 | 9.85E+03 | 9.70E+03 | 16.4 |  | x | x |
| 254.1499 | 1.41 | 3.23E+01 | 2.85E+01 | 4.03E+01 | 3.48E+01 | 3.50E+01 | 4.37E+01 | 3.30E+01 | 3.56E+01 | 3.04E+01 | 3.12E+01 | 3.76E+01 | 3.26E+01 | 4.47E+01 | 3.37E+01 | 3.89E+01 | 3.15E+01 | 3.79E+01 | 12.9 | x | x | x |
| 263.0972 | 1.41 | 9.75E+01 | 9.47E+01 | 1.09E+02 | 1.22E+02 | 1.23E+02 | 1.36E+02 | 1.14E+02 | 1.13E+02 | 1.19E+02 | 1.13E+02 | 1.07E+02 | 1.29E+02 | 1.27E+02 | 1.04E+02 | 1.16E+02 | 1.25E+02 | 1.25E+02 | 9.8 | x | x | x |
| 272.7885 | 1.41 | 2.19E+02 | 1.99E+02 | 2.07E+02 | 2.07E+02 | 2.15E+02 | 2.72E+02 | 2.15E+02 | 2.22E+02 | 2.18E+02 | 2.33E+02 | 2.14E+02 | 2.33E+02 | 2.44E+02 | 2.42E+02 | 2.25E+02 | 2.55E+02 | 2.20E+02 | 8.3 | x | x | x |
| 276.8503 | 1.41 | 1.35E+01 | 9.42E+00 | 1.18E+01 | 1.52E+01 | 1.05E+01 | 9.36E+00 | 1.17E+01 | 1.56E+01 | 1.49E+01 | 1.41E+01 | 1.26E+01 | 1.00E+01 | 1.08E+01 | 1.44E+01 | 1.11E+01 | 1.36E+01 | 1.65E+01 | 17.9 |  | x | x |
| 284.1196 | 1.41 | 2.94E+01 | 2.84E+01 | 2.66E+01 | 3.08E+01 | 2.87E+01 | 2.90E+01 | 2.43E+01 | 2.30E+01 | 2.96E+01 | 2.47E+01 | 2.45E+01 | 2.07E+01 | 2.49E+01 | 1.19E+01 | 3.17E+01 | 2.47E+01 | 2.27E+01 | 18.4 |  | x | x |
| 301.0540 | 1.41 | 7.48E+01 | 6.42E+01 | 8.15E+01 | 8.06E+01 | 7.88E+01 | 8.61E+01 | 6.50E+01 | 6.77E+01 | 7.12E+01 | 7.57E+01 | 7.09E+01 | 7.26E+01 | 7.85E+01 | 6.85E+01 | 6.70E+01 | 8.23E+01 | 5.52E+01 | 10.9 | x | x | x |
| 320.8248 | 1.41 | 5.95E+01 | 4.50E+01 | 5.38E+01 | 5.58E+01 | 5.64E+01 | 9.31E+01 | 6.01E+01 | 6.09E+01 | 5.89E+01 | 6.26E+01 | 5.95E+01 | 5.90E+01 | 6.87E+01 | 7.72E+01 | 6.28E+01 | 7.33E+01 | 8.48E+01 | 18.6 |  | x | x |
| 344.7226 | 1.41 | 3.86E+02 | 3.47E+02 | 3.50E+02 | 3.90E+02 | 3.99E+02 | 4.53E+02 | 3.73E+02 | 3.92E+02 | 3.91E+02 | 4.20E+02 | 4.10E+02 | 4.07E+02 | 4.06E+02 | 4.13E+02 | 3.98E+02 | 4.34E+02 | 3.90E+02 | 6.7 | x | x | x |
| 356.7497 | 1.41 | 4.77E+01 | 4.02E+01 | 4.77E+01 | 4.82E+01 | 4.82E+01 | 5.14E+01 | 4.13E+01 | 5.34E+01 | 4.49E+01 | 5.44E+01 | 4.10E+01 | 4.68E+01 | 3.75E+01 | 4.43E+01 | 4.48E+01 | 4.81E+01 | 4.89E+01 | 9.9 | x | x | x |
| 322.8227 | 1.41 | 4.96E+01 | 3.84E+01 | 4.49E+01 | 4.33E+01 | 3.99E+01 | 6.02E+01 | 4.45E+01 | 5.95E+01 | 3.97E+01 | 4.89E+01 | 3.68E+01 | 3.49E+01 | 4.38E+01 | 6.23E+01 | 3.75E+01 | 4.65E+01 | 4.73E+01 | 18.1 |  | x | x |
| 388.9255 | 1.41 | 5.58E+02 | 5.02E+02 | 5.11E+02 | 5.30E+02 | 5.23E+02 | 5.41E+02 | 5.30E+02 | 5.07E+02 | 4.90E+02 | 5.31E+02 | 4.87E+02 | 5.39E+02 | 5.70E+02 | 4.64E+02 | 5.36E+02 | 5.91E+02 | 5.71E+02 | 6.2 | x | x | x |
| 390.9237 | 1.41 | 5.32E+02 | 4.76E+02 | 5.17E+02 | 5.23E+02 | 5.17E+02 | 5.40E+02 | 5.15E+02 | 5.12E+02 | 4.77E+02 | 5.15E+02 | 4.92E+02 | 5.19E+02 | 5.49E+02 | 4.45E+02 | 5.34E+02 | 5.39E+02 | 5.60E+02 | 5.6 | x | x | x |
| 392.9217 | 1.41 | 7.89E+01 | 7.65E+01 | 7.05E+01 | 7.67E+01 | 7.32E+01 | 6.35E+01 | 7.69E+01 | 6.15E+01 | 5.90E+01 | 5.08E+01 | 5.72E+01 | 5.48E+01 | 7.56E+01 | 4.54E+01 | 5.41E+01 | 4.66E+01 | 5.16E+01 | 18.5 |  | x | x |
| 394.7575 | 1.41 | 2.01E+01 | 1.46E+01 | 1.53E+01 | 1.38E+01 | 1.43E+01 | 3.04E+01 | 1.69E+01 | 1.53E+01 | 2.00E+01 | 1.40E+01 | 1.54E+01 | 1.57E+01 | 1.91E+01 | 1.95E+01 | 1.48E+01 | 1.67E+01 | 2.28E+01 | 24.0 |  |  | x |
| 438.7127 | 1.41 | 2.41E+02 | 2.14E+02 | 2.31E+02 | 2.55E+02 | 2.36E+02 | 2.65E+02 | 2.25E+02 | 2.61E+02 | 2.16E+02 | 2.34E+02 | 2.28E+02 | 2.16E+02 | 2.37E+02 | 2.36E+02 | 2.10E+02 | 2.46E+02 | 2.27E+02 | 6.8 | x | x | x |
| 432.6791 | 1.41 | 6.32E+01 | 5.62E+01 | 5.49E+01 | 5.90E+01 | 5.97E+01 | 6.68E+01 | 6.89E+01 | 6.08E+01 | 5.78E+01 | 5.73E+01 | 5.35E+01 | 6.39E+01 | 6.46E+01 | 6.46E+01 | 5.23E+01 | 6.36E+01 | 5.32E+01 | 8.4 | x | x | x |
| 492.5848 | 1.41 | 5.77E+02 | 5.64E+02 | 5.75E+02 | 5.76E+02 | 5.82E+02 | 6.89E+02 | 5.67E+02 | 6.13E+02 | 5.86E+02 | 6.47E+02 | 5.60E+02 | 6.08E+02 | 6.23E+02 | 6.57E+02 | 6.15E+02 | 5.74E+02 | 5.71E+02 | 6.2 | x | x | x |
| 494.5856 | 1.41 | 1.49E+03 | 1.33E+03 | 1.38E+03 | 1.44E+03 | 1.48E+03 | 1.77E+03 | 1.47E+03 | 1.59E+03 | 1.50E+03 | 1.64E+03 | 1.53E+03 | 1.69E+03 | 1.66E+03 | 1.71E+03 | 1.60E+03 | 1.67E+03 | 1.59E+03 | 7.8 | x | x | x |
| 506.6125 | 1.41 | 4.88E+02 | 4.47E+02 | 4.59E+02 | 4.83E+02 | 4.72E+02 | 5.76E+02 | 4.58E+02 | 5.17E+02 | 5.00E+02 | 5.03E+02 | 4.72E+02 | 4.97E+02 | 5.20E+02 | 5.35E+02 | 4.88E+02 | 5.07E+02 | 4.74E+02 | 6.4 | x | x | x |
| 508.6094 | 1.41 | 8.53E+01 | 8.08E+01 | 7.48E+01 | 9.21E+01 | 9.82E+01 | 1.06E+02 | 1.02E+02 | 1.00E+02 | 9.08E+01 | 9.12E+01 | 8.37E+01 | 8.72E+01 | 1.09E+02 | 8.83E+01 | 9.54E+01 | 1.01E+02 | 9.03E+01 | 10.0 | x | x | x |
| 522.6720 | 1.41 | 7.68E+01 | 7.65E+01 | 7.87E+01 | 7.71E+01 | 8.72E+01 | 7.15E+01 | 7.66E+01 | 7.15E+01 | 7.13E+01 | 7.88E+01 | 7.62E+01 | 7.69E+01 | 9.00E+01 | 7.97E+01 | 7.76E+01 | 7.33E+01 | 8.95E+01 | 7.3 | x | x | x |
| 642.5868 | 1.41 | 5.09E+01 | 5.13E+01 | 5.42E+01 | 5.11E+01 | 5.67E+01 | 6.26E+01 | 5.69E+01 | 5.45E+01 | 4.63E+01 | 5.80E+01 | 4.95E+01 | 5.76E+01 | 6.58E+01 | 4.84E+01 | 4.82E+01 | 6.67E+01 | 5.00E+01 | 11.2 | x | x | x |
| 822.3998 | 1.41 | 8.68E+01 | 7.66E+01 | 9.85E+01 | 1.02E+02 | 8.33E+01 | 7.80E+01 | 8.40E+01 | 7.27E+01 | 6.29E+01 | 7.54E+01 | 6.72E+01 | 6.39E+01 | 7.30E+01 | 5.78E+01 | 6.10E+01 | 6.11E+01 | 6.80E+01 | 17.2 |  | x | x |
| 868.3723 | 1.41 | 1.15E+02 | 9.81E+01 | 1.18E+02 | 1.04E+02 | 1.07E+02 | 9.13E+01 | 8.40E+01 | 8.78E+01 | 8.81E+01 | 9.00E+01 | 7.29E+01 | 6.55E+01 | 7.06E+01 | 5.07E+01 | 8.64E+01 | 6.52E+01 | 7.00E+01 | 21.7 |  |  | x |
| 896.3330 | 1.41 | 2.77E+02 | 2.40E+02 | 2.67E+02 | 2.69E+02 | 2.79E+02 | 2.39E+02 | 2.59E+02 | 2.63E+02 | 2.02E+02 | 2.73E+02 | 2.23E+02 | 2.54E+02 | 2.45E+02 | 2.29E+02 | 2.25E+02 | 1.96E+02 | 1.94E+02 | 11.6 | x | x | x |
| 888.3047 | 1.41 | 2.90E+02 | 2.68E+02 | 3.01E+02 | 2.49E+02 | 2.80E+02 | 2.70E+02 | 2.78E+02 | 2.81E+02 | 2.14E+02 | 2.74E+02 | 2.23E+02 | 2.32E+02 | 2.31E+02 | 1.99E+02 | 2.32E+02 | 2.18E+02 | 2.17E+02 | 12.6 | x | x | x |
| 894.3286 | 1.41 | 8.36E+01 | 7.85E+01 | 8.08E+01 | 8.93E+01 | 7.45E+01 | 8.59E+01 | 7.73E+01 | 7.46E+01 | 6.46E+01 | 7.66E+01 | 6.28E+01 | 6.74E+01 | 6.17E+01 | 6.79E+01 | 8.14E+01 | 7.24E+01 | 5.81E+01 | 12.2 | x | x | x |
| 980.2923 | 1.41 | 2.41E+02 | 2.10E+02 | 2.11E+02 | 2.24E+02 | 2.10E+02 | 1.94E+02 | 2.14E+02 | 2.03E+02 | 1.64E+02 | 2.02E+02 | 1.71E+02 | 1.62E+02 | 1.78E+02 | 1.84E+02 | 1.57E+02 | 1.58E+02 | 1.14E+02 | 16.6 |  | x | x |
| 982.2955 | 1.41 | 6.00E+01 | 3.82E+01 | 3.93E+01 | 4.86E+01 | 4.70E+01 | 4.00E+01 | 5.21E+01 | 4.91E+01 | 4.34E+01 | 3.40E+01 | 3.47E+01 | 4.23E+01 | 2.51E+01 | 5.39E+01 | 3.64E+01 | 1.56E+01 | 3.51E+01 | 26.5 |  |  | x |
| 1054.2210 | 1.41 | 1.51E+02 | 1.24E+02 | 1.62E+02 | 1.27E+02 | 1.03E+02 | 1.14E+02 | 1.20E+02 | 1.06E+02 | 7.92E+01 | 9.56E+01 | 9.76E+01 | 9.11E+01 | 7.09E+01 | 8.54E+01 | 9.02E+01 | 4.54E+01 | 6.76E+01 | 29.2 |  |  | x |
| 81.0450 | 1.42 | 1.16E+01 | 1.45E+01 | 1.50E+01 | 1.25E+01 | 1.56E+01 | 1.13E+01 | 1.49E+01 | 1.45E+01 | 1.45E+01 | 1.09E+01 | 1.05E+01 | 1.10E+01 | 7.00E+00 | 1.49E+01 | 9.21E+00 | 1.02E+01 | 4.75E+00 | 25.8 |  |  | x |
| 116.8913 | 1.42 | 7.43E+01 | 6.90E+01 | 7.52E+01 | 7.08E+01 | 7.22E+01 | 8.14E+01 | 7.34E+01 | 7.63E+01 | 6.75E+01 | 7.50E+01 | 7.14E+01 | 7.52E+01 | 7.30E+01 | 6.83E+01 | 6.38E+01 | 7.01E+01 | 5.54E+01 | 8.1 | x | x | x |
| 112.8963 | 1.42 | 3.61E+03 | 3.51E+03 | 3.51E+03 | 3.74E+03 | 3.75E+03 | 4.66E+03 | 3.85E+03 | 4.06E+03 | 3.83E+03 | 4.44E+03 | 4.14E+03 | 4.53E+03 | 4.78E+03 | 4.53E+03 | 4.54E+03 | 4.90E+03 | 4.77E+03 | 11.5 | x | x | x |
| 114.8936 | 1.42 | 1.36E+03 | 1.31E+03 | 1.31E+03 | 1.37E+03 | 1.38E+03 | 1.67E+03 | 1.39E+03 | 1.48E+03 | 1.40E+03 | 1.62E+03 | 1.48E+03 | 1.63E+03 | 1.71E+03 | 1.61E+03 | 1.56E+03 | 1.71E+03 | 1.66E+03 | 9.5 | x | x | x |
| 150.0781 | 1.42 | 3.44E+02 | 3.32E+02 | 3.47E+02 | 3.45E+02 | 3.51E+02 | 4.13E+02 | 3.58E+02 | 3.79E+02 | 3.74E+02 | 4.04E+02 | 3.68E+02 | 4.24E+02 | 4.25E+02 | 4.04E+02 | 3.67E+02 | 4.11E+02 | 3.82E+02 | 8.0 | x | x | x |
| 186.8280 | 1.42 | 4.86E+01 | 3.81E+01 | 4.53E+01 | 4.48E+01 | 4.77E+01 | 5.11E+01 | 4.21E+01 | 4.80E+01 | 4.68E+01 | 4.14E+01 | 4.00E+01 | 5.29E+01 | 4.72E+01 | 4.48E+01 | 4.00E+01 | 4.47E+01 | 3.68E+01 | 10.0 | x | x | x |
| 231.1707 | 1.42 | 4.07E+03 | 4.06E+03 | 3.98E+03 | 4.06E+03 | 4.00E+03 | 4.89E+03 | 4.02E+03 | 4.19E+03 | 3.96E+03 | 4.53E+03 | 4.28E+03 | 4.66E+03 | 5.12E+03 | 4.73E+03 | 4.96E+03 | 5.40E+03 | 5.33E+03 | 11.2 | x | x | x |
| 260.7615 | 1.42 | 2.47E+03 | 2.32E+03 | 2.33E+03 | 2.51E+03 | 2.55E+03 | 3.26E+03 | 2.64E+03 | 2.83E+03 | 2.72E+03 | 3.11E+03 | 2.89E+03 | 3.22E+03 | 3.23E+03 | 3.23E+03 | 3.08E+03 | 3.36E+03 | 3.16E+03 | 12.4 | x | x | x |
| 484.5570 | 1.42 | 2.33E+03 | 2.16E+03 | 2.17E+03 | 2.33E+03 | 2.39E+03 | 2.96E+03 | 2.42E+03 | 2.63E+03 | 2.52E+03 | 2.83E+03 | 2.69E+03 | 2.93E+03 | 2.81E+03 | 2.96E+03 | 2.68E+03 | 2.98E+03 | 2.82E+03 | 10.8 | x | x | x |
| 266.7534 | 1.42 | 1.13E+02 | 1.13E+02 | 1.13E+02 | 1.07E+02 | 1.18E+02 | 1.33E+02 | 1.10E+02 | 1.22E+02 | 1.16E+02 | 1.31E+02 | 1.18E+02 | 1.30E+02 | 1.15E+02 | 1.26E+02 | 1.03E+02 | 1.15E+02 | 1.05E+02 | 7.7 | x | x | x |
| 262.7588 | 1.42 | 3.25E+03 | 3.15E+03 | 3.12E+03 | 3.36E+03 | 3.43E+03 | 4.42E+03 | 3.54E+03 | 3.82E+03 | 3.70E+03 | 4.19E+03 | 3.97E+03 | 4.35E+03 | 4.38E+03 | 4.44E+03 | 4.19E+03 | 4.57E+03 | 4.46E+03 | 13.2 | x | x | x |
| 338.6885 | 1.42 | 2.17E+03 | 2.07E+03 | 2.07E+03 | 2.24E+03 | 2.30E+03 | 2.97E+03 | 2.33E+03 | 2.54E+03 | 2.44E+03 | 2.75E+03 | 2.61E+03 | 2.83E+03 | 2.79E+03 | 2.90E+03 | 2.60E+03 | 2.97E+03 | 2.80E+03 | 12.2 | x | x | x |
| 334.6940 | 1.42 | 1.62E+03 | 1.56E+03 | 1.55E+03 | 1.67E+03 | 1.73E+03 | 2.17E+03 | 1.73E+03 | 1.89E+03 | 1.79E+03 | 2.05E+03 | 1.93E+03 | 2.08E+03 | 2.05E+03 | 2.14E+03 | 1.94E+03 | 2.16E+03 | 2.03E+03 | 11.3 | x | x | x |
| 336.6912 | 1.42 | 3.24E+03 | 3.10E+03 | 3.11E+03 | 3.37E+03 | 3.49E+03 | 4.54E+03 | 3.56E+03 | 3.87E+03 | 3.77E+03 | 4.28E+03 | 4.04E+03 | 4.43E+03 | 4.41E+03 | 4.51E+03 | 4.16E+03 | 4.66E+03 | 4.41E+03 | 13.7 | x | x | x |
| 340.6860 | 1.42 | 3.61E+02 | 3.47E+02 | 3.63E+02 | 3.84E+02 | 3.86E+02 | 4.51E+02 | 3.64E+02 | 4.35E+02 | 4.09E+02 | 4.36E+02 | 4.29E+02 | 4.55E+02 | 4.70E+02 | 4.63E+02 | 4.15E+02 | 4.64E+02 | 4.18E+02 | 9.8 | x | x | x |
| 342.6828 | 1.42 | 3.05E+01 | 2.96E+01 | 2.85E+01 | 3.46E+01 | 3.16E+01 | 3.58E+01 | 3.18E+01 | 3.31E+01 | 3.04E+01 | 2.73E+01 | 2.37E+01 | 3.36E+01 | 3.18E+01 | 3.62E+01 | 2.73E+01 | 2.71E+01 | 3.22E+01 | 10.9 | x | x | x |
| 346.7202 | 1.42 | 5.35E+02 | 4.85E+02 | 4.84E+02 | 5.27E+02 | 5.59E+02 | 6.65E+02 | 5.53E+02 | 5.65E+02 | 5.35E+02 | 5.89E+02 | 5.86E+02 | 5.90E+02 | 6.38E+02 | 6.20E+02 | 5.61E+02 | 6.02E+02 | 5.79E+02 | 8.6 | x | x | x |
| 408.6259 | 1.42 | 2.10E+02 | 2.04E+02 | 2.25E+02 | 2.21E+02 | 2.10E+02 | 2.30E+02 | 2.04E+02 | 2.15E+02 | 2.21E+02 | 2.24E+02 | 2.03E+02 | 2.17E+02 | 2.26E+02 | 2.14E+02 | 1.92E+02 | 2.06E+02 | 2.01E+02 | 5.0 | x | x | x |
| 410.6236 | 1.42 | 4.97E+02 | 4.41E+02 | 4.61E+02 | 4.76E+02 | 4.97E+02 | 5.78E+02 | 4.61E+02 | 5.32E+02 | 4.97E+02 | 5.49E+02 | 4.98E+02 | 5.35E+02 | 5.29E+02 | 5.52E+02 | 4.87E+02 | 5.26E+02 | 4.85E+02 | 7.3 | x | x | x |
| 412.6210 | 1.42 | 3.52E+02 | 3.30E+02 | 3.41E+02 | 3.68E+02 | 3.61E+02 | 4.33E+02 | 3.33E+02 | 4.02E+02 | 3.67E+02 | 4.04E+02 | 3.67E+02 | 3.95E+02 | 3.51E+02 | 4.00E+02 | 3.36E+02 | 3.85E+02 | 3.17E+02 | 8.7 | x | x | x |
| 414.6183 | 1.42 | 1.12E+02 | 9.62E+01 | 1.06E+02 | 1.19E+02 | 1.17E+02 | 1.08E+02 | 1.02E+02 | 1.08E+02 | 1.03E+02 | 1.13E+02 | 1.05E+02 | 9.51E+01 | 7.63E+01 | 1.00E+02 | 7.87E+01 | 9.77E+01 | 7.85E+01 | 12.8 | x | x | x |
| 424.6472 | 1.42 | 2.96E+01 | 2.35E+01 | 2.60E+01 | 2.33E+01 | 2.22E+01 | 1.83E+01 | 2.65E+01 | 2.75E+01 | 2.37E+01 | 2.80E+01 | 1.89E+01 | 2.20E+01 | 2.17E+01 | 2.78E+01 | 8.36E+00 | 1.64E+01 | 6.56E+00 | 29.9 |  |  | x |
| 486.5543 | 1.42 | 1.37E+03 | 1.27E+03 | 1.25E+03 | 1.36E+03 | 1.41E+03 | 1.75E+03 | 1.35E+03 | 1.54E+03 | 1.49E+03 | 1.64E+03 | 1.58E+03 | 1.67E+03 | 1.64E+03 | 1.73E+03 | 1.51E+03 | 1.71E+03 | 1.59E+03 | 10.7 | x | x | x |
| 488.5516 | 1.42 | 6.65E+02 | 6.29E+02 | 6.38E+02 | 6.74E+02 | 6.79E+02 | 8.30E+02 | 6.35E+02 | 7.45E+02 | 7.39E+02 | 7.74E+02 | 7.53E+02 | 7.88E+02 | 7.63E+02 | 8.36E+02 | 6.88E+02 | 7.93E+02 | 7.42E+02 | 9.2 | x | x | x |
| 496.5834 | 1.42 | 6.48E+02 | 5.99E+02 | 6.25E+02 | 6.59E+02 | 6.68E+02 | 8.04E+02 | 6.46E+02 | 7.21E+02 | 7.05E+02 | 7.18E+02 | 7.18E+02 | 7.39E+02 | 7.59E+02 | 7.94E+02 | 7.29E+02 | 7.84E+02 | 7.34E+02 | 8.5 | x | x | x |
| 482.5595 | 1.42 | 3.48E+02 | 3.08E+02 | 3.20E+02 | 3.37E+02 | 3.44E+02 | 3.75E+02 | 3.30E+02 | 3.64E+02 | 3.52E+02 | 3.89E+02 | 3.65E+02 | 3.87E+02 | 3.51E+02 | 3.59E+02 | 3.10E+02 | 3.62E+02 | 3.22E+02 | 7.1 | x | x | x |
| 490.5488 | 1.42 | 2.35E+02 | 2.11E+02 | 2.16E+02 | 2.18E+02 | 2.36E+02 | 2.55E+02 | 2.07E+02 | 2.45E+02 | 2.39E+02 | 2.36E+02 | 2.21E+02 | 2.43E+02 | 2.33E+02 | 2.68E+02 | 1.90E+02 | 2.26E+02 | 2.13E+02 | 8.3 | x | x | x |
| 562.4841 | 1.42 | 2.70E+02 | 2.56E+02 | 2.62E+02 | 2.57E+02 | 2.74E+02 | 3.12E+02 | 2.59E+02 | 2.80E+02 | 2.71E+02 | 2.95E+02 | 2.80E+02 | 3.01E+02 | 2.79E+02 | 3.12E+02 | 2.68E+02 | 2.84E+02 | 2.64E+02 | 6.5 | x | x | x |
| 566.5063 | 1.42 | 6.81E+01 | 6.48E+01 | 6.29E+01 | 5.93E+01 | 6.74E+01 | 7.99E+01 | 6.26E+01 | 6.73E+01 | 6.85E+01 | 6.68E+01 | 6.90E+01 | 7.43E+01 | 7.24E+01 | 7.40E+01 | 6.37E+01 | 6.75E+01 | 6.94E+01 | 7.4 | x | x | x |
| 578.5474 | 1.42 | 1.14E+02 | 1.06E+02 | 1.16E+02 | 1.15E+02 | 1.14E+02 | 1.30E+02 | 1.13E+02 | 1.16E+02 | 1.17E+02 | 1.22E+02 | 1.17E+02 | 1.18E+02 | 1.26E+02 | 1.28E+02 | 1.21E+02 | 1.32E+02 | 1.15E+02 | 5.8 | x | x | x |
| 568.5179 | 1.42 | 2.07E+02 | 1.86E+02 | 1.95E+02 | 2.09E+02 | 2.00E+02 | 2.29E+02 | 1.93E+02 | 2.23E+02 | 2.10E+02 | 2.28E+02 | 2.07E+02 | 2.20E+02 | 2.18E+02 | 2.16E+02 | 1.93E+02 | 1.93E+02 | 2.15E+02 | 6.3 | x | x | x |
| 556.4919 | 1.42 | 9.09E+01 | 8.49E+01 | 8.86E+01 | 8.38E+01 | 8.66E+01 | 1.02E+02 | 9.36E+01 | 8.63E+01 | 7.44E+01 | 9.98E+01 | 7.04E+01 | 1.02E+02 | 7.91E+01 | 1.02E+02 | 6.01E+01 | 6.30E+01 | 6.16E+01 | 16.9 |  | x | x |
| 558.4895 | 1.42 | 4.98E+02 | 4.75E+02 | 4.82E+02 | 4.97E+02 | 4.97E+02 | 5.81E+02 | 4.83E+02 | 5.52E+02 | 5.19E+02 | 5.46E+02 | 5.37E+02 | 5.46E+02 | 5.36E+02 | 5.84E+02 | 4.92E+02 | 5.23E+02 | 5.05E+02 | 6.5 | x | x | x |
| 570.5160 | 1.42 | 4.14E+02 | 3.78E+02 | 3.96E+02 | 3.89E+02 | 3.96E+02 | 4.91E+02 | 4.15E+02 | 4.32E+02 | 4.00E+02 | 4.31E+02 | 4.11E+02 | 4.41E+02 | 4.63E+02 | 4.74E+02 | 4.11E+02 | 4.47E+02 | 4.13E+02 | 7.4 | x | x | x |
| 646.4461 | 1.42 | 2.53E+02 | 2.26E+02 | 2.34E+02 | 2.42E+02 | 2.34E+02 | 2.49E+02 | 2.27E+02 | 2.46E+02 | 2.39E+02 | 2.40E+02 | 2.41E+02 | 2.61E+02 | 2.36E+02 | 2.74E+02 | 2.40E+02 | 2.55E+02 | 2.49E+02 | 5.0 | x | x | x |
| 580.5453 | 1.42 | 1.07E+02 | 1.13E+02 | 1.22E+02 | 1.17E+02 | 1.21E+02 | 1.24E+02 | 1.05E+02 | 1.19E+02 | 1.12E+02 | 1.24E+02 | 1.06E+02 | 1.25E+02 | 1.47E+02 | 1.31E+02 | 1.19E+02 | 1.37E+02 | 1.20E+02 | 9.1 | x | x | x |
| 640.4228 | 1.42 | 8.93E+01 | 1.03E+02 | 9.84E+01 | 1.04E+02 | 1.04E+02 | 9.87E+01 | 9.04E+01 | 9.90E+01 | 8.97E+01 | 9.54E+01 | 9.15E+01 | 1.18E+02 | 1.03E+02 | 1.01E+02 | 8.63E+01 | 1.06E+02 | 9.08E+01 | 8.2 | x | x | x |
| 634.4199 | 1.42 | 4.15E+02 | 4.07E+02 | 4.62E+02 | 4.22E+02 | 4.38E+02 | 4.87E+02 | 4.38E+02 | 4.54E+02 | 4.47E+02 | 4.90E+02 | 4.42E+02 | 4.98E+02 | 4.43E+02 | 5.12E+02 | 4.48E+02 | 4.68E+02 | 4.59E+02 | 6.4 | x | x | x |
| 638.4140 | 1.42 | 1.84E+02 | 1.61E+02 | 1.85E+02 | 1.57E+02 | 1.74E+02 | 1.84E+02 | 1.82E+02 | 1.84E+02 | 1.96E+02 | 1.88E+02 | 1.69E+02 | 1.88E+02 | 1.94E+02 | 2.05E+02 | 1.55E+02 | 1.73E+02 | 1.88E+02 | 7.7 | x | x | x |
| 704.3565 | 1.42 | 6.81E+01 | 6.38E+01 | 7.32E+01 | 5.65E+01 | 5.07E+01 | 7.31E+01 | 5.60E+01 | 6.21E+01 | 6.75E+01 | 5.88E+01 | 5.67E+01 | 6.82E+01 | 5.21E+01 | 6.59E+01 | 5.21E+01 | 7.26E+01 | 6.09E+01 | 12.2 | x | x | x |
| 636.4171 | 1.42 | 3.93E+02 | 4.19E+02 | 4.17E+02 | 3.85E+02 | 3.75E+02 | 4.33E+02 | 3.85E+02 | 4.11E+02 | 4.19E+02 | 4.21E+02 | 3.90E+02 | 4.55E+02 | 4.18E+02 | 4.35E+02 | 4.16E+02 | 4.36E+02 | 4.16E+02 | 5.2 | x | x | x |
| 736.4403 | 1.42 | 1.39E+02 | 1.05E+02 | 1.34E+02 | 1.38E+02 | 1.37E+02 | 1.23E+02 | 1.29E+02 | 1.33E+02 | 1.16E+02 | 1.30E+02 | 1.23E+02 | 1.48E+02 | 1.49E+02 | 1.25E+02 | 1.34E+02 | 1.41E+02 | 1.20E+02 | 8.7 | x | x | x |
| 710.3506 | 1.42 | 6.39E+02 | 6.36E+02 | 6.79E+02 | 6.71E+02 | 6.81E+02 | 7.71E+02 | 6.76E+02 | 7.27E+02 | 6.68E+02 | 8.06E+02 | 7.22E+02 | 8.32E+02 | 7.74E+02 | 7.92E+02 | 7.60E+02 | 7.70E+02 | 7.67E+02 | 8.4 | x | x | x |
| 706.3556 | 1.42 | 3.14E+02 | 2.88E+02 | 3.06E+02 | 2.89E+02 | 2.93E+02 | 3.20E+02 | 3.16E+02 | 3.23E+02 | 3.15E+02 | 3.33E+02 | 3.06E+02 | 3.50E+02 | 3.12E+02 | 3.05E+02 | 2.87E+02 | 2.90E+02 | 2.94E+02 | 5.7 | x | x | x |
| 708.3532 | 1.42 | 5.88E+02 | 5.68E+02 | 5.94E+02 | 5.99E+02 | 5.90E+02 | 6.94E+02 | 6.30E+02 | 6.36E+02 | 6.28E+02 | 7.15E+02 | 6.39E+02 | 7.08E+02 | 6.91E+02 | 7.13E+02 | 6.47E+02 | 7.16E+02 | 7.06E+02 | 8.0 | x | x | x |
| 746.4693 | 1.42 | 4.52E+01 | 5.07E+01 | 4.57E+01 | 5.16E+01 | 4.26E+01 | 3.25E+01 | 5.16E+01 | 4.57E+01 | 3.93E+01 | 3.94E+01 | 5.15E+01 | 5.59E+01 | 4.56E+01 | 3.85E+01 | 4.36E+01 | 3.61E+01 | 3.44E+01 | 15.3 |  | x | x |
| 712.3479 | 1.42 | 4.46E+02 | 4.17E+02 | 4.57E+02 | 4.30E+02 | 4.34E+02 | 4.72E+02 | 4.40E+02 | 4.65E+02 | 4.48E+02 | 5.04E+02 | 4.56E+02 | 4.99E+02 | 4.70E+02 | 4.96E+02 | 4.37E+02 | 5.00E+02 | 4.71E+02 | 5.8 | x | x | x |
| 714.3517 | 1.42 | 2.33E+02 | 2.31E+02 | 2.25E+02 | 2.02E+02 | 2.18E+02 | 2.22E+02 | 2.20E+02 | 2.11E+02 | 2.17E+02 | 2.41E+02 | 2.05E+02 | 2.10E+02 | 2.33E+02 | 2.17E+02 | 2.18E+02 | 2.00E+02 | 2.38E+02 | 5.6 | x | x | x |
| 716.5158 | 1.42 | 6.05E+01 | 5.15E+01 | 4.89E+01 | 5.05E+01 | 4.45E+01 | 4.15E+01 | 5.30E+01 | 5.28E+01 | 4.30E+01 | 6.59E+01 | 6.33E+01 | 4.60E+01 | 4.35E+01 | 3.68E+01 | 5.30E+01 | 5.87E+01 | 3.43E+01 | 17.9 |  | x | x |
| 738.4386 | 1.42 | 1.06E+02 | 1.00E+02 | 1.14E+02 | 1.02E+02 | 9.81E+01 | 1.15E+02 | 1.06E+02 | 1.07E+02 | 9.62E+01 | 1.12E+02 | 9.77E+01 | 8.29E+01 | 1.05E+02 | 1.05E+02 | 1.10E+02 | 1.01E+02 | 9.77E+01 | 7.5 | x | x | x |
| 790.2968 | 1.42 | 8.97E+01 | 8.60E+01 | 8.13E+01 | 6.96E+01 | 8.08E+01 | 7.75E+01 | 7.84E+01 | 8.41E+01 | 7.83E+01 | 7.55E+01 | 7.41E+01 | 7.21E+01 | 8.15E+01 | 8.51E+01 | 6.60E+01 | 8.43E+01 | 5.40E+01 | 11.2 | x | x | x |
| 740.4367 | 1.42 | 8.59E+01 | 7.15E+01 | 8.80E+01 | 7.72E+01 | 7.94E+01 | 7.00E+01 | 6.63E+01 | 8.25E+01 | 6.57E+01 | 5.02E+01 | 6.43E+01 | 7.60E+01 | 7.27E+01 | 3.97E+01 | 6.05E+01 | 5.54E+01 | 5.76E+01 | 19.0 |  | x | x |
| 782.2854 | 1.42 | 2.07E+02 | 2.10E+02 | 2.44E+02 | 2.07E+02 | 1.99E+02 | 2.14E+02 | 1.98E+02 | 2.06E+02 | 2.06E+02 | 2.17E+02 | 1.98E+02 | 2.15E+02 | 2.23E+02 | 2.03E+02 | 1.92E+02 | 2.08E+02 | 1.86E+02 | 6.4 | x | x | x |
| 824.3972 | 1.42 | 1.48E+02 | 1.18E+02 | 1.51E+02 | 1.44E+02 | 1.30E+02 | 9.61E+01 | 1.08E+02 | 1.09E+02 | 1.12E+02 | 9.99E+01 | 9.83E+01 | 1.11E+02 | 1.12E+02 | 1.04E+02 | 7.52E+01 | 5.88E+01 | 1.00E+02 | 21.6 |  |  | x |
| 786.2806 | 1.42 | 8.90E+01 | 7.74E+01 | 7.27E+01 | 7.27E+01 | 7.64E+01 | 7.51E+01 | 7.58E+01 | 8.59E+01 | 6.96E+01 | 7.90E+01 | 6.45E+01 | 8.68E+01 | 6.50E+01 | 6.38E+01 | 6.93E+01 | 7.30E+01 | 7.07E+01 | 10.1 | x | x | x |
| 792.3091 | 1.42 | 6.57E+01 | 5.23E+01 | 6.12E+01 | 4.98E+01 | 5.93E+01 | 6.97E+01 | 4.65E+01 | 5.44E+01 | 5.33E+01 | 6.83E+01 | 4.50E+01 | 6.43E+01 | 6.12E+01 | 5.34E+01 | 4.35E+01 | 4.64E+01 | 4.91E+01 | 15.2 |  | x | x |
| 806.3449 | 1.42 | 4.70E+01 | 4.47E+01 | 4.81E+01 | 5.08E+01 | 3.76E+01 | 5.16E+01 | 4.31E+01 | 5.29E+01 | 3.62E+01 | 4.59E+01 | 3.80E+01 | 4.06E+01 | 4.51E+01 | 3.06E+01 | 3.27E+01 | 4.33E+01 | 4.10E+01 | 15.0 | x | x | x |
| 856.2211 | 1.42 | 1.05E+02 | 1.04E+02 | 1.13E+02 | 1.03E+02 | 9.92E+01 | 9.72E+01 | 1.04E+02 | 9.57E+01 | 9.72E+01 | 9.42E+01 | 6.38E+01 | 9.49E+01 | 7.98E+01 | 7.45E+01 | 6.86E+01 | 7.25E+01 | 8.06E+01 | 16.1 |  | x | x |
| 784.2827 | 1.42 | 1.67E+02 | 1.65E+02 | 1.72E+02 | 1.54E+02 | 1.75E+02 | 1.69E+02 | 1.45E+02 | 1.79E+02 | 1.80E+02 | 1.73E+02 | 1.62E+02 | 1.90E+02 | 1.76E+02 | 1.51E+02 | 1.60E+02 | 1.75E+02 | 1.66E+02 | 6.8 | x | x | x |
| 820.3985 | 1.42 | 2.08E+02 | 2.00E+02 | 2.39E+02 | 2.22E+02 | 2.06E+02 | 2.26E+02 | 1.95E+02 | 2.01E+02 | 1.86E+02 | 1.99E+02 | 1.82E+02 | 1.83E+02 | 2.02E+02 | 2.05E+02 | 1.83E+02 | 1.81E+02 | 1.36E+02 | 11.5 | x | x | x |
| 862.2176 | 1.42 | 3.80E+01 | 3.51E+01 | 4.98E+01 | 3.87E+01 | 4.22E+01 | 3.90E+01 | 3.47E+01 | 2.58E+01 | 2.19E+01 | 2.04E+01 | 1.90E+01 | 2.99E+01 | 3.02E+01 | 3.15E+01 | 1.95E+01 | 2.93E+01 | 3.28E+01 | 27.2 |  |  | x |
| 880.2796 | 1.42 | 6.65E+01 | 6.24E+01 | 6.35E+01 | 6.08E+01 | 5.29E+01 | 6.53E+01 | 5.50E+01 | 5.38E+01 | 5.02E+01 | 6.17E+01 | 4.52E+01 | 7.13E+01 | 5.63E+01 | 6.76E+01 | 5.37E+01 | 6.07E+01 | 6.33E+01 | 11.6 | x | x | x |
| 882.2877 | 1.42 | 5.54E+01 | 5.62E+01 | 5.06E+01 | 5.50E+01 | 4.85E+01 | 4.58E+01 | 5.11E+01 | 5.11E+01 | 4.97E+01 | 5.32E+01 | 5.78E+01 | 4.95E+01 | 4.93E+01 | 4.74E+01 | 3.88E+01 | 5.49E+01 | 3.54E+01 | 11.8 | x | x | x |
| 884.2997 | 1.42 | 8.77E+01 | 7.89E+01 | 7.94E+01 | 8.71E+01 | 8.85E+01 | 9.39E+01 | 8.65E+01 | 8.40E+01 | 6.69E+01 | 7.25E+01 | 6.41E+01 | 7.34E+01 | 8.34E+01 | 7.74E+01 | 5.49E+01 | 6.16E+01 | 5.17E+01 | 16.4 |  | x | x |
| 892.3184 | 1.42 | 7.20E+01 | 6.80E+01 | 7.17E+01 | 7.14E+01 | 6.91E+01 | 7.00E+01 | 7.15E+01 | 5.63E+01 | 6.81E+01 | 5.57E+01 | 6.12E+01 | 4.33E+01 | 4.96E+01 | 6.80E+01 | 4.13E+01 | 4.73E+01 | 5.89E+01 | 17.4 |  | x | x |
| 934.1486 | 1.42 | 1.90E+02 | 1.79E+02 | 1.77E+02 | 1.67E+02 | 1.60E+02 | 1.54E+02 | 1.69E+02 | 1.62E+02 | 1.64E+02 | 1.77E+02 | 1.59E+02 | 1.69E+02 | 1.54E+02 | 1.59E+02 | 1.60E+02 | 1.64E+02 | 1.42E+02 | 6.8 | x | x | x |
| 890.3070 | 1.42 | 1.16E+02 | 1.07E+02 | 1.29E+02 | 1.21E+02 | 1.13E+02 | 9.94E+01 | 1.01E+02 | 1.02E+02 | 8.96E+01 | 1.01E+02 | 9.45E+01 | 9.43E+01 | 7.91E+01 | 9.10E+01 | 7.48E+01 | 8.74E+01 | 7.56E+01 | 15.7 |  | x | x |
| 932.1503 | 1.42 | 5.09E+01 | 4.78E+01 | 7.02E+01 | 5.96E+01 | 4.73E+01 | 5.24E+01 | 5.36E+01 | 5.26E+01 | 4.37E+01 | 4.95E+01 | 3.87E+01 | 4.87E+01 | 5.08E+01 | 4.99E+01 | 5.22E+01 | 4.66E+01 | 3.28E+01 | 16.0 |  | x | x |
| 952.2103 | 1.42 | 5.76E+01 | 4.95E+01 | 3.97E+01 | 4.61E+01 | 4.23E+01 | 5.59E+01 | 4.66E+01 | 4.40E+01 | 4.93E+01 | 5.78E+01 | 4.85E+01 | 5.14E+01 | 4.07E+01 | 4.31E+01 | 4.11E+01 | 5.25E+01 | 4.48E+01 | 12.2 | x | x | x |
| 956.2218 | 1.42 | 4.85E+01 | 4.07E+01 | 4.76E+01 | 4.39E+01 | 4.30E+01 | 4.57E+01 | 3.12E+01 | 3.68E+01 | 3.92E+01 | 4.33E+01 | 3.62E+01 | 3.88E+01 | 4.23E+01 | 3.98E+01 | 3.98E+01 | 2.96E+01 | 2.99E+01 | 14.3 | x | x | x |
| 960.2377 | 1.42 | 8.68E+01 | 8.47E+01 | 9.00E+01 | 8.48E+01 | 7.15E+01 | 8.48E+01 | 9.44E+01 | 8.77E+01 | 7.55E+01 | 7.69E+01 | 7.08E+01 | 7.38E+01 | 7.69E+01 | 8.17E+01 | 7.68E+01 | 7.80E+01 | 7.24E+01 | 8.7 | x | x | x |
| 968.2615 | 1.42 | 7.86E+01 | 6.05E+01 | 6.55E+01 | 7.12E+01 | 7.05E+01 | 5.19E+01 | 7.32E+01 | 5.20E+01 | 5.82E+01 | 6.34E+01 | 6.65E+01 | 5.94E+01 | 7.12E+01 | 5.92E+01 | 6.74E+01 | 7.10E+01 | 4.12E+01 | 14.8 | x | x | x |
| 976.2772 | 1.42 | 1.17E+02 | 9.07E+01 | 1.07E+02 | 1.08E+02 | 9.44E+01 | 8.79E+01 | 1.04E+02 | 1.05E+02 | 9.69E+01 | 8.32E+01 | 5.27E+01 | 7.76E+01 | 8.76E+01 | 8.25E+01 | 6.23E+01 | 7.32E+01 | 6.03E+01 | 20.9 |  |  | x |
| 1028.1395 | 1.42 | 6.50E+02 | 6.34E+02 | 6.40E+02 | 6.11E+02 | 6.28E+02 | 7.03E+02 | 6.30E+02 | 6.75E+02 | 5.90E+02 | 7.26E+02 | 6.52E+02 | 7.64E+02 | 7.06E+02 | 6.93E+02 | 6.47E+02 | 6.73E+02 | 6.83E+02 | 6.7 | x | x | x |
| 972.2655 | 1.42 | 1.15E+02 | 1.08E+02 | 1.02E+02 | 1.04E+02 | 1.01E+02 | 1.09E+02 | 1.01E+02 | 9.40E+01 | 6.24E+01 | 8.99E+01 | 9.54E+01 | 8.82E+01 | 8.74E+01 | 7.73E+01 | 8.57E+01 | 9.36E+01 | 6.83E+01 | 15.2 |  | x | x |
| 1002.0891 | 1.42 | 7.69E+01 | 7.17E+01 | 8.98E+01 | 7.23E+01 | 8.72E+01 | 8.61E+01 | 6.58E+01 | 7.27E+01 | 6.82E+01 | 8.53E+01 | 5.27E+01 | 6.82E+01 | 8.98E+01 | 7.98E+01 | 5.67E+01 | 8.10E+01 | 6.33E+01 | 15.2 |  | x | x |
| 1004.0862 | 1.42 | 3.25E+02 | 3.33E+02 | 3.30E+02 | 2.98E+02 | 3.34E+02 | 3.55E+02 | 3.21E+02 | 3.41E+02 | 3.31E+02 | 3.99E+02 | 3.28E+02 | 4.08E+02 | 3.40E+02 | 3.89E+02 | 3.59E+02 | 3.72E+02 | 3.81E+02 | 8.8 | x | x | x |
| 1010.0801 | 1.42 | 1.04E+03 | 1.02E+03 | 1.03E+03 | 1.00E+03 | 1.04E+03 | 1.14E+03 | 1.01E+03 | 1.07E+03 | 1.03E+03 | 1.19E+03 | 1.03E+03 | 1.21E+03 | 1.09E+03 | 1.12E+03 | 1.08E+03 | 1.16E+03 | 1.11E+03 | 6.0 | x | x | x |
| 1012.0867 | 1.42 | 1.03E+03 | 9.28E+02 | 9.99E+02 | 8.97E+02 | 9.84E+02 | 1.09E+03 | 9.72E+02 | 9.91E+02 | 9.59E+02 | 1.06E+03 | 9.60E+02 | 1.06E+03 | 9.84E+02 | 1.08E+03 | 9.86E+02 | 1.04E+03 | 1.01E+03 | 5.3 | x | x | x |
| 1014.0997 | 1.42 | 1.02E+03 | 9.67E+02 | 1.03E+03 | 9.85E+02 | 1.03E+03 | 1.10E+03 | 1.00E+03 | 1.04E+03 | 9.45E+02 | 1.10E+03 | 9.69E+02 | 1.15E+03 | 1.04E+03 | 1.08E+03 | 1.02E+03 | 1.08E+03 | 1.04E+03 | 5.3 | x | x | x |
| 1016.1077 | 1.42 | 4.94E+02 | 4.81E+02 | 5.03E+02 | 4.59E+02 | 4.91E+02 | 5.87E+02 | 4.88E+02 | 5.16E+02 | 4.94E+02 | 5.62E+02 | 5.13E+02 | 5.87E+02 | 5.49E+02 | 5.53E+02 | 5.10E+02 | 5.85E+02 | 5.71E+02 | 7.9 | x | x | x |
| 1018.1089 | 1.42 | 4.14E+02 | 4.09E+02 | 4.23E+02 | 4.01E+02 | 4.44E+02 | 4.96E+02 | 4.27E+02 | 4.35E+02 | 4.05E+02 | 5.02E+02 | 4.39E+02 | 5.01E+02 | 4.73E+02 | 4.79E+02 | 4.83E+02 | 4.40E+02 | 4.85E+02 | 7.9 | x | x | x |
| 1006.0829 | 1.42 | 1.08E+03 | 1.08E+03 | 1.14E+03 | 1.02E+03 | 1.12E+03 | 1.23E+03 | 1.09E+03 | 1.20E+03 | 1.08E+03 | 1.29E+03 | 1.11E+03 | 1.29E+03 | 1.15E+03 | 1.19E+03 | 1.19E+03 | 1.22E+03 | 1.24E+03 | 6.8 | x | x | x |
| 1008.0806 | 1.42 | 1.29E+03 | 1.23E+03 | 1.31E+03 | 1.23E+03 | 1.27E+03 | 1.51E+03 | 1.29E+03 | 1.37E+03 | 1.30E+03 | 1.56E+03 | 1.36E+03 | 1.55E+03 | 1.42E+03 | 1.50E+03 | 1.38E+03 | 1.53E+03 | 1.49E+03 | 8.4 | x | x | x |
| 1030.1432 | 1.42 | 2.34E+02 | 2.22E+02 | 2.42E+02 | 2.41E+02 | 2.39E+02 | 2.56E+02 | 2.31E+02 | 2.37E+02 | 2.26E+02 | 2.65E+02 | 2.15E+02 | 2.71E+02 | 2.84E+02 | 2.67E+02 | 2.55E+02 | 2.43E+02 | 2.56E+02 | 7.6 | x | x | x |
| 1020.1114 | 1.42 | 5.32E+02 | 5.29E+02 | 5.44E+02 | 5.22E+02 | 5.26E+02 | 5.98E+02 | 5.15E+02 | 5.45E+02 | 4.95E+02 | 6.21E+02 | 5.44E+02 | 5.86E+02 | 5.45E+02 | 5.47E+02 | 5.62E+02 | 6.24E+02 | 5.82E+02 | 6.6 | x | x | x |
| 1040.1733 | 1.42 | 1.84E+02 | 1.54E+02 | 1.50E+02 | 1.56E+02 | 1.51E+02 | 1.87E+02 | 1.57E+02 | 1.65E+02 | 1.37E+02 | 1.63E+02 | 1.34E+02 | 1.51E+02 | 1.74E+02 | 1.66E+02 | 1.46E+02 | 1.56E+02 | 1.50E+02 | 9.1 | x | x | x |
| 87.0447 | 1.43 | 9.08E+01 | 9.88E+01 | 9.27E+01 | 8.43E+01 | 8.64E+01 | 9.71E+01 | 8.19E+01 | 9.19E+01 | 7.81E+01 | 9.34E+01 | 7.79E+01 | 8.75E+01 | 8.69E+01 | 8.26E+01 | 6.88E+01 | 7.31E+01 | 5.90E+01 | 12.4 | x | x | x |
| 146.0929 | 1.43 | 1.55E+03 | 1.56E+03 | 1.52E+03 | 1.51E+03 | 1.51E+03 | 1.72E+03 | 1.60E+03 | 1.67E+03 | 1.59E+03 | 1.87E+03 | 1.74E+03 | 1.97E+03 | 1.90E+03 | 1.88E+03 | 1.84E+03 | 1.94E+03 | 1.92E+03 | 9.9 | x | x | x |
| 181.0261 | 1.43 | 2.17E+01 | 2.14E+01 | 1.85E+01 | 2.27E+01 | 2.48E+01 | 2.82E+01 | 2.16E+01 | 2.44E+01 | 1.97E+01 | 2.32E+01 | 2.09E+01 | 2.07E+01 | 2.44E+01 | 1.94E+01 | 2.50E+01 | 1.70E+01 | 1.60E+01 | 14.4 | x | x | x |
| 216.0198 | 1.43 | 3.67E+02 | 3.36E+02 | 3.79E+02 | 3.96E+02 | 4.38E+02 | 5.40E+02 | 3.94E+02 | 4.28E+02 | 4.18E+02 | 4.38E+02 | 4.27E+02 | 4.71E+02 | 5.73E+02 | 4.70E+02 | 4.55E+02 | 4.67E+02 | 4.50E+02 | 13.4 | x | x | x |
| 218.0179 | 1.43 | 1.11E+02 | 9.74E+01 | 1.18E+02 | 1.30E+02 | 1.22E+02 | 1.57E+02 | 1.25E+02 | 1.28E+02 | 1.24E+02 | 1.20E+02 | 1.30E+02 | 1.30E+02 | 1.62E+02 | 1.29E+02 | 1.13E+02 | 1.18E+02 | 1.21E+02 | 12.2 | x | x | x |
| 235.0366 | 1.43 | 1.56E+01 | 1.53E+01 | 1.49E+01 | 1.52E+01 | 1.41E+01 | 1.08E+01 | 1.22E+01 | 1.19E+01 | 1.32E+01 | 1.93E+01 | 1.31E+01 | 1.18E+01 | 1.54E+01 | 1.34E+01 | 1.23E+01 | 6.71E+00 | 1.18E+01 | 20.1 |  |  | x |
| 251.1390 | 1.43 | 3.71E+01 | 3.68E+01 | 3.77E+01 | 3.60E+01 | 3.66E+01 | 3.01E+01 | 3.64E+01 | 3.02E+01 | 2.58E+01 | 3.00E+01 | 2.89E+01 | 2.67E+01 | 3.19E+01 | 3.31E+01 | 3.65E+01 | 3.98E+01 | 2.17E+01 | 15.4 |  | x | x |
| 273.0484 | 1.43 | 1.47E+02 | 1.42E+02 | 1.40E+02 | 1.58E+02 | 1.59E+02 | 2.05E+02 | 1.57E+02 | 1.59E+02 | 1.56E+02 | 1.69E+02 | 1.49E+02 | 1.75E+02 | 2.00E+02 | 1.77E+02 | 1.78E+02 | 1.77E+02 | 1.76E+02 | 11.3 | x | x | x |
| 275.0676 | 1.43 | 1.58E+02 | 1.36E+02 | 1.52E+02 | 1.64E+02 | 1.87E+02 | 2.36E+02 | 1.69E+02 | 1.85E+02 | 1.71E+02 | 1.62E+02 | 1.75E+02 | 1.89E+02 | 2.30E+02 | 1.71E+02 | 1.77E+02 | 1.80E+02 | 1.62E+02 | 14.2 | x | x | x |
| 112.0871 | 1.44 | 1.07E+02 | 1.02E+02 | 1.06E+02 | 1.05E+02 | 1.12E+02 | 1.35E+02 | 1.13E+02 | 1.14E+02 | 1.20E+02 | 1.19E+02 | 1.11E+02 | 1.34E+02 | 1.21E+02 | 1.35E+02 | 1.13E+02 | 1.27E+02 | 1.28E+02 | 9.1 | x | x | x |
| 154.0977 | 1.44 | 1.54E+03 | 1.50E+03 | 1.63E+03 | 1.64E+03 | 1.72E+03 | 2.16E+03 | 1.74E+03 | 1.87E+03 | 1.83E+03 | 2.11E+03 | 1.96E+03 | 2.19E+03 | 2.21E+03 | 2.17E+03 | 2.13E+03 | 2.27E+03 | 2.20E+03 | 13.7 | x | x | x |
| 207.1131 | 1.44 | 5.20E+01 | 5.15E+01 | 4.52E+01 | 4.84E+01 | 5.29E+01 | 4.97E+01 | 4.43E+01 | 4.86E+01 | 4.05E+01 | 5.82E+01 | 5.09E+01 | 4.75E+01 | 4.67E+01 | 5.31E+01 | 5.47E+01 | 5.67E+01 | 4.12E+01 | 10.1 | x | x | x |
| 217.1547 | 1.44 | 4.63E+01 | 4.89E+01 | 5.06E+01 | 4.26E+01 | 4.39E+01 | 4.14E+01 | 4.21E+01 | 4.15E+01 | 3.83E+01 | 4.43E+01 | 3.07E+01 | 3.04E+01 | 4.16E+01 | 3.61E+01 | 2.94E+01 | 4.09E+01 | 3.27E+01 | 15.9 |  | x | x |
| 267.1249 | 1.44 | 3.04E+02 | 3.09E+02 | 2.61E+02 | 2.50E+02 | 2.35E+02 | 2.42E+02 | 2.58E+02 | 2.43E+02 | 2.41E+02 | 3.04E+02 | 2.58E+02 | 2.89E+02 | 2.44E+02 | 2.81E+02 | 2.55E+02 | 2.92E+02 | 2.58E+02 | 9.4 | x | x | x |
| 297.1450 | 1.44 | 1.15E+03 | 1.21E+03 | 1.21E+03 | 1.16E+03 | 1.17E+03 | 1.37E+03 | 1.21E+03 | 1.31E+03 | 1.24E+03 | 1.41E+03 | 1.32E+03 | 1.52E+03 | 1.50E+03 | 1.40E+03 | 1.44E+03 | 1.58E+03 | 1.59E+03 | 11.2 | x | x | x |
| 420.0099 | 1.44 | 2.77E+01 | 1.81E+01 | 1.82E+01 | 1.96E+01 | 2.02E+01 | 1.36E+01 | 2.62E+01 | 2.12E+01 | 1.55E+01 | 2.58E+01 | 2.23E+01 | 1.88E+01 | 1.61E+01 | 2.63E+01 | 9.14E+00 | 1.61E+01 | 1.94E+01 | 25.3 |  |  | x |
| 296.0651 | 1.45 | 5.94E+02 | 5.31E+02 | 5.46E+02 | 5.92E+02 | 6.07E+02 | 7.58E+02 | 6.32E+02 | 6.84E+02 | 6.52E+02 | 7.07E+02 | 6.82E+02 | 7.53E+02 | 6.90E+02 | 6.57E+02 | 6.64E+02 | 6.57E+02 | 6.23E+02 | 9.7 | x | x | x |
| 351.1005 | 1.45 | 8.23E+01 | 6.88E+01 | 7.07E+01 | 6.59E+01 | 6.74E+01 | 6.92E+01 | 6.67E+01 | 6.32E+01 | 6.22E+01 | 5.89E+01 | 5.83E+01 | 5.45E+01 | 6.42E+01 | 6.95E+01 | 6.31E+01 | 6.80E+01 | 5.02E+01 | 11.1 | x | x | x |
| 369.1088 | 1.45 | 1.31E+02 | 9.51E+01 | 1.14E+02 | 1.13E+02 | 1.30E+02 | 1.65E+02 | 1.04E+02 | 1.29E+02 | 1.13E+02 | 1.23E+02 | 1.26E+02 | 1.09E+02 | 1.33E+02 | 1.21E+02 | 1.16E+02 | 9.91E+01 | 1.05E+02 | 13.8 | x | x | x |
| 260.0546 | 1.46 | 4.53E+02 | 4.11E+02 | 4.82E+02 | 4.69E+02 | 4.90E+02 | 5.77E+02 | 4.63E+02 | 4.93E+02 | 4.91E+02 | 5.17E+02 | 4.85E+02 | 5.42E+02 | 5.48E+02 | 5.03E+02 | 5.61E+02 | 5.43E+02 | 5.05E+02 | 8.5 | x | x | x |
| 264.0387 | 1.46 | 2.73E+02 | 2.49E+02 | 2.70E+02 | 2.69E+02 | 2.86E+02 | 3.36E+02 | 2.94E+02 | 3.21E+02 | 3.07E+02 | 3.29E+02 | 3.06E+02 | 3.45E+02 | 3.40E+02 | 3.66E+02 | 3.61E+02 | 3.64E+02 | 3.66E+02 | 12.1 | x | x | x |
| 371.1068 | 1.46 | 3.00E+01 | 2.75E+01 | 3.69E+01 | 3.16E+01 | 3.85E+01 | 4.00E+01 | 2.14E+01 | 3.07E+01 | 2.42E+01 | 2.60E+01 | 3.07E+01 | 2.59E+01 | 2.51E+01 | 1.82E+01 | 1.56E+01 | 1.59E+01 | 1.49E+01 | 29.3 |  |  | x |
| 152.0757 | 1.47 | 2.59E+01 | 2.06E+01 | 3.03E+01 | 3.01E+01 | 3.13E+01 | 3.36E+01 | 2.62E+01 | 3.12E+01 | 3.11E+01 | 2.63E+01 | 2.88E+01 | 2.92E+01 | 2.60E+01 | 2.40E+01 | 2.38E+01 | 2.05E+01 | 1.75E+01 | 16.7 |  | x | x |
| 158.1175 | 1.47 | 4.01E+03 | 3.75E+03 | 3.95E+03 | 4.12E+03 | 4.13E+03 | 5.24E+03 | 4.46E+03 | 4.61E+03 | 4.47E+03 | 5.28E+03 | 4.81E+03 | 5.29E+03 | 5.62E+03 | 5.41E+03 | 5.57E+03 | 6.00E+03 | 5.89E+03 | 15.0 | x | x | x |
| 196.0740 | 1.47 | 6.02E+03 | 5.95E+03 | 5.95E+03 | 6.28E+03 | 6.51E+03 | 9.13E+03 | 6.45E+03 | 7.11E+03 | 6.70E+03 | 7.85E+03 | 7.46E+03 | 8.35E+03 | 9.10E+03 | 8.58E+03 | 8.79E+03 | 9.13E+03 | 8.90E+03 | 16.5 |  | x | x |
| 227.1376 | 1.47 | 4.37E+01 | 3.22E+01 | 2.95E+01 | 3.25E+01 | 3.23E+01 | 3.53E+01 | 3.16E+01 | 3.67E+01 | 3.45E+01 | 3.16E+01 | 2.30E+01 | 3.03E+01 | 2.55E+01 | 2.81E+01 | 2.44E+01 | 3.21E+01 | 1.72E+01 | 19.5 |  | x | x |
| 428.6839 | 1.47 | 2.26E+01 | 1.84E+01 | 1.72E+01 | 1.55E+01 | 2.31E+01 | 2.08E+01 | 1.52E+01 | 1.26E+01 | 1.29E+01 | 1.18E+01 | 1.45E+01 | 1.08E+01 | 1.86E+01 | 1.08E+01 | 1.48E+01 | 1.27E+01 | 1.06E+01 | 26.2 |  |  | x |
| 514.6429 | 1.47 | 6.23E+01 | 6.46E+01 | 6.65E+01 | 5.26E+01 | 6.60E+01 | 7.95E+01 | 5.72E+01 | 7.26E+01 | 5.95E+01 | 6.27E+01 | 6.04E+01 | 6.79E+01 | 6.42E+01 | 5.98E+01 | 6.52E+01 | 6.00E+01 | 6.08E+01 | 9.6 | x | x | x |
| 144.1022 | 1.48 | 4.74E+03 | 4.26E+03 | 4.60E+03 | 5.00E+03 | 5.05E+03 | 7.03E+03 | 5.43E+03 | 5.80E+03 | 5.49E+03 | 6.49E+03 | 6.06E+03 | 6.49E+03 | 6.63E+03 | 6.30E+03 | 6.34E+03 | 6.78E+03 | 6.77E+03 | 14.9 | x | x | x |
| 156.0427 | 1.48 | 4.85E+03 | 4.78E+03 | 4.88E+03 | 5.38E+03 | 5.27E+03 | 6.03E+03 | 5.77E+03 | 5.96E+03 | 5.62E+03 | 6.65E+03 | 6.27E+03 | 7.01E+03 | 8.05E+03 | 7.55E+03 | 8.12E+03 | 8.72E+03 | 8.70E+03 | 20.9 |  |  | x |
| 170.0587 | 1.48 | 5.63E+02 | 5.15E+02 | 5.53E+02 | 5.99E+02 | 5.76E+02 | 6.72E+02 | 6.10E+02 | 6.27E+02 | 6.08E+02 | 6.59E+02 | 6.30E+02 | 7.15E+02 | 7.47E+02 | 7.06E+02 | 7.58E+02 | 8.03E+02 | 7.61E+02 | 12.8 | x | x | x |
| 182.0584 | 1.48 | 6.78E+03 | 6.38E+03 | 6.60E+03 | 7.24E+03 | 7.21E+03 | 8.64E+03 | 7.67E+03 | 8.02E+03 | 7.67E+03 | 8.92E+03 | 8.34E+03 | 9.08E+03 | 9.99E+03 | 9.39E+03 | 1.02E+04 | 1.10E+04 | 1.07E+04 | 17.1 |  | x | x |
| 198.1603 | 1.48 | 2.53E+01 | 2.18E+01 | 2.72E+01 | 3.09E+01 | 2.90E+01 | 3.79E+01 | 3.27E+01 | 4.11E+01 | 3.80E+01 | 3.88E+01 | 3.55E+01 | 3.11E+01 | 2.83E+01 | 2.72E+01 | 1.91E+01 | 2.28E+01 | 2.52E+01 | 21.5 |  |  | x |
| 218.9943 | 1.48 | 1.07E+01 | 1.26E+01 | 1.22E+01 | 1.52E+01 | 1.36E+01 | 7.24E+00 | 1.40E+01 | 1.09E+01 | 1.05E+01 | 1.68E+01 | 8.85E+00 | 1.30E+01 | 7.92E+00 | 1.40E+01 | 1.15E+01 | 7.40E+00 | 5.90E+00 | 27.1 |  |  | x |
| 313.1535 | 1.48 | 2.86E+01 | 2.87E+01 | 3.86E+01 | 2.92E+01 | 2.82E+01 | 3.60E+01 | 2.67E+01 | 3.40E+01 | 3.20E+01 | 3.58E+01 | 3.38E+01 | 4.09E+01 | 3.54E+01 | 3.61E+01 | 3.71E+01 | 3.85E+01 | 3.57E+01 | 12.5 | x | x | x |
| 320.9890 | 1.48 | 6.88E+02 | 7.87E+02 | 6.58E+02 | 6.46E+02 | 6.30E+02 | 7.42E+02 | 6.68E+02 | 7.15E+02 | 6.85E+02 | 8.23E+02 | 7.38E+02 | 8.31E+02 | 8.47E+02 | 8.52E+02 | 9.61E+02 | 1.14E+03 | 1.02E+03 | 18.0 |  | x | x |
| 322.9881 | 1.48 | 5.67E+01 | 6.21E+01 | 5.92E+01 | 5.13E+01 | 5.57E+01 | 4.72E+01 | 5.29E+01 | 4.65E+01 | 5.41E+01 | 6.33E+01 | 5.68E+01 | 6.25E+01 | 6.01E+01 | 5.11E+01 | 6.72E+01 | 7.96E+01 | 6.92E+01 | 14.4 | x | x | x |
| 340.1815 | 1.48 | 9.27E+01 | 7.86E+01 | 6.73E+01 | 7.43E+01 | 7.13E+01 | 1.00E+02 | 8.35E+01 | 9.25E+01 | 7.78E+01 | 1.05E+02 | 7.87E+01 | 8.19E+01 | 7.01E+01 | 8.32E+01 | 5.27E+01 | 7.49E+01 | 7.41E+01 | 15.9 |  | x | x |
| 360.9976 | 1.48 | 2.63E+01 | 2.48E+01 | 2.41E+01 | 2.55E+01 | 2.17E+01 | 2.26E+01 | 2.68E+01 | 2.70E+01 | 2.31E+01 | 2.59E+01 | 2.30E+01 | 2.40E+01 | 1.87E+01 | 2.09E+01 | 1.86E+01 | 2.23E+01 | 1.47E+01 | 14.4 | x | x | x |
| 58.0660 | 1.49 | 5.77E+00 | 5.55E+00 | 6.33E+00 | 6.14E+00 | 7.31E+00 | 7.45E+00 | 7.84E+00 | 7.75E+00 | 8.84E+00 | 8.05E+00 | 5.80E+00 | 6.35E+00 | 4.78E+00 | 4.34E+00 | 7.03E+00 | 5.38E+00 | 6.56E+00 | 18.7 |  | x | x |
| 118.0866 | 1.49 | 6.66E+03 | 5.98E+03 | 6.29E+03 | 6.69E+03 | 6.76E+03 | 9.63E+03 | 7.21E+03 | 7.63E+03 | 7.28E+03 | 8.65E+03 | 8.07E+03 | 8.54E+03 | 8.40E+03 | 7.89E+03 | 7.76E+03 | 8.21E+03 | 8.18E+03 | 12.6 | x | x | x |
| 120.0815 | 1.49 | 4.13E+02 | 3.98E+02 | 4.12E+02 | 4.29E+02 | 4.18E+02 | 4.62E+02 | 4.53E+02 | 4.55E+02 | 4.59E+02 | 4.81E+02 | 4.43E+02 | 5.14E+02 | 5.34E+02 | 5.24E+02 | 5.55E+02 | 5.86E+02 | 5.70E+02 | 12.5 | x | x | x |
| 125.0360 | 1.49 | 8.83E+00 | 1.03E+01 | 7.36E+00 | 7.09E+00 | 9.93E+00 | 7.86E+00 | 1.24E+01 | 1.06E+01 | 1.23E+01 | 8.10E+00 | 7.23E+00 | 8.68E+00 | 9.87E+00 | 5.68E+00 | 3.02E+00 | 7.48E+00 | 7.41E+00 | 27.5 |  |  | x |
| 126.0665 | 1.49 | 3.27E+02 | 2.99E+02 | 3.23E+02 | 3.45E+02 | 3.49E+02 | 4.84E+02 | 3.75E+02 | 3.83E+02 | 3.81E+02 | 4.43E+02 | 3.91E+02 | 4.34E+02 | 4.23E+02 | 4.19E+02 | 4.34E+02 | 4.53E+02 | 4.34E+02 | 13.3 | x | x | x |
| 146.0607 | 1.49 | 4.18E+01 | 3.84E+01 | 4.03E+01 | 4.47E+01 | 4.63E+01 | 5.04E+01 | 4.61E+01 | 4.47E+01 | 4.49E+01 | 3.92E+01 | 4.71E+01 | 4.83E+01 | 5.97E+01 | 4.63E+01 | 4.50E+01 | 4.94E+01 | 4.61E+01 | 10.7 | x | x | x |
| 179.0458 | 1.49 | 2.60E+01 | 2.16E+01 | 2.62E+01 | 2.46E+01 | 2.32E+01 | 2.52E+01 | 2.62E+01 | 2.42E+01 | 2.50E+01 | 2.83E+01 | 2.60E+01 | 2.50E+01 | 2.12E+01 | 1.85E+01 | 1.83E+01 | 2.08E+01 | 2.04E+01 | 12.5 | x | x | x |
| 188.0710 | 1.49 | 1.01E+02 | 9.31E+01 | 1.02E+02 | 9.59E+01 | 1.07E+02 | 1.18E+02 | 9.92E+01 | 1.16E+02 | 1.05E+02 | 1.21E+02 | 1.05E+02 | 1.18E+02 | 1.15E+02 | 1.16E+02 | 1.19E+02 | 1.23E+02 | 1.29E+02 | 9.5 | x | x | x |
| 191.0456 | 1.49 | 4.21E+01 | 4.40E+01 | 4.62E+01 | 4.24E+01 | 4.65E+01 | 4.98E+01 | 3.88E+01 | 4.67E+01 | 4.24E+01 | 4.59E+01 | 4.68E+01 | 4.01E+01 | 4.23E+01 | 3.69E+01 | 4.12E+01 | 4.62E+01 | 4.12E+01 | 7.8 | x | x | x |
| 238.0942 | 1.49 | 1.23E+02 | 1.21E+02 | 1.17E+02 | 1.16E+02 | 1.11E+02 | 1.14E+02 | 1.21E+02 | 1.32E+02 | 1.18E+02 | 1.36E+02 | 1.22E+02 | 1.40E+02 | 1.13E+02 | 1.16E+02 | 1.11E+02 | 1.10E+02 | 1.20E+02 | 7.4 | x | x | x |
| 257.1474 | 1.49 | 3.42E+03 | 3.59E+03 | 3.67E+03 | 3.56E+03 | 3.83E+03 | 4.23E+03 | 4.10E+03 | 3.95E+03 | 3.87E+03 | 4.62E+03 | 4.28E+03 | 4.82E+03 | 5.78E+03 | 6.12E+03 | 6.55E+03 | 7.04E+03 | 7.18E+03 | 27.0 |  |  | x |
| 285.1546 | 1.49 | 9.96E+01 | 9.43E+01 | 1.19E+02 | 1.06E+02 | 1.13E+02 | 1.07E+02 | 1.02E+02 | 1.06E+02 | 9.00E+01 | 9.75E+01 | 9.53E+01 | 9.77E+01 | 1.07E+02 | 9.96E+01 | 1.07E+02 | 1.19E+02 | 1.31E+02 | 9.9 | x | x | x |
| 305.0155 | 1.49 | 7.09E+02 | 6.61E+02 | 6.36E+02 | 7.08E+02 | 7.53E+02 | 1.06E+03 | 7.92E+02 | 8.55E+02 | 7.80E+02 | 9.51E+02 | 8.47E+02 | 9.29E+02 | 9.18E+02 | 1.01E+03 | 9.59E+02 | 9.63E+02 | 9.84E+02 | 15.3 |  | x | x |
| 314.0895 | 1.49 | 1.12E+02 | 1.03E+02 | 1.01E+02 | 9.59E+01 | 8.30E+01 | 7.14E+01 | 8.57E+01 | 9.06E+01 | 8.01E+01 | 8.66E+01 | 8.73E+01 | 6.90E+01 | 8.00E+01 | 6.85E+01 | 7.32E+01 | 6.84E+01 | 6.54E+01 | 16.3 |  | x | x |
| 381.1748 | 1.49 | 9.03E+02 | 8.08E+02 | 8.55E+02 | 9.72E+02 | 9.89E+02 | 8.90E+02 | 1.07E+03 | 1.13E+03 | 1.04E+03 | 1.14E+03 | 1.14E+03 | 1.20E+03 | 1.02E+03 | 1.10E+03 | 1.06E+03 | 1.07E+03 | 1.09E+03 | 10.7 | x | x | x |
| 203.0533 | 1.50 | 2.12E+01 | 1.90E+01 | 1.76E+01 | 1.87E+01 | 2.05E+01 | 1.82E+01 | 1.36E+01 | 1.40E+01 | 1.82E+01 | 1.62E+01 | 1.66E+01 | 1.42E+01 | 1.79E+01 | 1.44E+01 | 1.38E+01 | 1.27E+01 | 1.62E+01 | 15.4 |  | x | x |
| 208.0134 | 1.50 | 2.48E+01 | 2.90E+01 | 2.77E+01 | 2.89E+01 | 2.39E+01 | 3.06E+01 | 1.87E+01 | 2.88E+01 | 2.50E+01 | 3.06E+01 | 2.12E+01 | 3.15E+01 | 3.68E+01 | 2.24E+01 | 1.31E+01 | 1.79E+01 | 2.01E+01 | 23.6 |  |  | x |
| 218.9205 | 1.50 | 2.01E+01 | 1.55E+01 | 1.70E+01 | 2.05E+01 | 2.48E+01 | 1.07E+01 | 1.97E+01 | 2.05E+01 | 2.33E+01 | 1.92E+01 | 2.15E+01 | 2.74E+01 | 2.60E+01 | 1.45E+01 | 1.70E+01 | 1.79E+01 | 9.26E+00 | 25.8 |  |  | x |
| 267.0597 | 1.50 | 1.67E+03 | 1.55E+03 | 1.63E+03 | 1.77E+03 | 1.84E+03 | 2.32E+03 | 1.92E+03 | 1.99E+03 | 1.89E+03 | 2.18E+03 | 2.03E+03 | 2.17E+03 | 2.21E+03 | 2.24E+03 | 2.02E+03 | 2.21E+03 | 2.21E+03 | 11.9 | x | x | x |
| 280.0707 | 1.50 | 8.98E+02 | 9.26E+02 | 8.53E+02 | 9.40E+02 | 1.03E+03 | 1.55E+03 | 9.99E+02 | 1.11E+03 | 1.02E+03 | 1.18E+03 | 1.10E+03 | 1.19E+03 | 1.33E+03 | 1.46E+03 | 1.52E+03 | 1.66E+03 | 1.68E+03 | 23.0 |  |  | x |
| 289.0417 | 1.50 | 1.54E+03 | 1.47E+03 | 1.55E+03 | 1.77E+03 | 1.93E+03 | 2.28E+03 | 1.90E+03 | 2.05E+03 | 2.01E+03 | 2.25E+03 | 2.15E+03 | 2.36E+03 | 2.47E+03 | 2.46E+03 | 2.33E+03 | 2.40E+03 | 2.45E+03 | 16.4 |  | x | x |
| 338.0874 | 1.50 | 4.86E+02 | 4.71E+02 | 4.09E+02 | 4.95E+02 | 4.62E+02 | 7.52E+02 | 4.31E+02 | 4.58E+02 | 4.59E+02 | 4.94E+02 | 4.68E+02 | 5.17E+02 | 5.46E+02 | 4.27E+02 | 4.84E+02 | 5.04E+02 | 4.56E+02 | 15.5 |  | x | x |
| 360.0695 | 1.50 | 3.43E+02 | 3.34E+02 | 2.87E+02 | 3.51E+02 | 3.67E+02 | 4.70E+02 | 3.48E+02 | 3.71E+02 | 3.65E+02 | 3.62E+02 | 3.76E+02 | 4.20E+02 | 4.41E+02 | 3.71E+02 | 3.69E+02 | 4.23E+02 | 3.72E+02 | 11.5 | x | x | x |
| 397.0532 | 1.50 | 1.31E+02 | 1.29E+02 | 9.08E+01 | 1.34E+02 | 1.24E+02 | 2.25E+02 | 1.06E+02 | 1.31E+02 | 1.37E+02 | 1.27E+02 | 1.22E+02 | 1.33E+02 | 1.55E+02 | 1.00E+02 | 1.39E+02 | 1.64E+02 | 1.40E+02 | 21.8 |  |  | x |
| 407.1892 | 1.50 | 3.04E+01 | 2.97E+01 | 3.09E+01 | 3.03E+01 | 2.75E+01 | 3.00E+01 | 3.19E+01 | 2.56E+01 | 3.04E+01 | 2.58E+01 | 2.56E+01 | 3.39E+01 | 2.26E+01 | 3.79E+01 | 3.57E+01 | 3.21E+01 | 3.24E+01 | 12.9 | x | x | x |
| 413.9988 | 1.50 | 2.60E+01 | 1.78E+01 | 2.01E+01 | 3.05E+01 | 3.10E+01 | 4.93E+01 | 3.06E+01 | 3.32E+01 | 3.72E+01 | 2.40E+01 | 2.96E+01 | 2.50E+01 | 3.07E+01 | 2.30E+01 | 2.52E+01 | 3.32E+01 | 2.13E+01 | 26.0 |  |  | x |
| 80.9482 | 1.51 | 4.22E+01 | 3.72E+01 | 3.90E+01 | 4.19E+01 | 4.69E+01 | 3.60E+01 | 3.72E+01 | 4.07E+01 | 3.99E+01 | 3.05E+01 | 3.91E+01 | 4.13E+01 | 4.63E+01 | 3.85E+01 | 4.10E+01 | 3.22E+01 | 3.18E+01 | 11.8 | x | x | x |
| 96.0787 | 1.51 | 2.40E+02 | 2.39E+02 | 2.37E+02 | 2.41E+02 | 2.43E+02 | 2.62E+02 | 2.53E+02 | 2.60E+02 | 2.41E+02 | 2.57E+02 | 2.68E+02 | 2.71E+02 | 2.77E+02 | 3.05E+02 | 2.91E+02 | 3.14E+02 | 3.17E+02 | 10.1 | x | x | x |
| 140.0686 | 1.51 | 8.01E+03 | 7.97E+03 | 8.24E+03 | 8.46E+03 | 8.86E+03 | 9.72E+03 | 9.12E+03 | 9.34E+03 | 8.86E+03 | 1.02E+04 | 9.76E+03 | 1.05E+04 | 1.20E+04 | 1.25E+04 | 1.25E+04 | 1.35E+04 | 1.38E+04 | 19.0 |  | x | x |
| 216.9233 | 1.51 | 1.09E+02 | 9.39E+01 | 9.97E+01 | 1.38E+02 | 1.50E+02 | 9.98E+01 | 1.05E+02 | 1.33E+02 | 1.42E+02 | 1.04E+02 | 1.30E+02 | 1.33E+02 | 1.55E+02 | 1.28E+02 | 1.37E+02 | 1.33E+02 | 1.24E+02 | 15.2 |  | x | x |
| 241.9986 | 1.51 | 3.69E+01 | 3.19E+01 | 3.62E+01 | 4.43E+01 | 4.36E+01 | 5.88E+01 | 4.34E+01 | 5.47E+01 | 4.62E+01 | 4.47E+01 | 5.21E+01 | 5.48E+01 | 6.31E+01 | 5.47E+01 | 5.42E+01 | 5.91E+01 | 5.13E+01 | 18.1 |  | x | x |
| 290.8476 | 1.51 | 5.90E+01 | 6.97E+01 | 6.19E+01 | 6.16E+01 | 5.85E+01 | 7.43E+01 | 5.77E+01 | 6.33E+01 | 5.96E+01 | 5.95E+01 | 6.42E+01 | 7.58E+01 | 6.55E+01 | 7.94E+01 | 7.23E+01 | 7.96E+01 | 7.20E+01 | 11.4 | x | x | x |
| 293.1204 | 1.51 | 2.50E+02 | 2.39E+02 | 2.67E+02 | 2.31E+02 | 2.87E+02 | 2.12E+02 | 2.43E+02 | 2.36E+02 | 2.73E+02 | 2.53E+02 | 2.29E+02 | 2.67E+02 | 3.40E+02 | 2.79E+02 | 3.33E+02 | 3.32E+02 | 3.41E+02 | 15.5 |  | x | x |
| 309.1973 | 1.51 | 2.77E+01 | 3.61E+01 | 3.99E+01 | 2.32E+01 | 2.71E+01 | 1.61E+01 | 3.01E+01 | 3.12E+01 | 3.49E+01 | 2.08E+01 | 2.28E+01 | 3.08E+01 | 2.67E+01 | 2.81E+01 | 2.68E+01 | 3.60E+01 | 3.99E+01 | 22.6 |  |  | x |
| 401.1428 | 1.51 | 4.98E+01 | 3.96E+01 | 4.07E+01 | 5.62E+01 | 6.70E+01 | 3.60E+01 | 6.00E+01 | 6.42E+01 | 6.61E+01 | 5.44E+01 | 6.07E+01 | 7.88E+01 | 7.62E+01 | 4.83E+01 | 5.49E+01 | 4.36E+01 | 4.80E+01 | 22.4 |  |  | x |
| 152.0580 | 1.52 | 1.18E+02 | 1.07E+02 | 1.34E+02 | 1.23E+02 | 1.21E+02 | 1.53E+02 | 1.26E+02 | 1.36E+02 | 1.37E+02 | 1.51E+02 | 1.40E+02 | 1.61E+02 | 1.57E+02 | 1.50E+02 | 1.52E+02 | 1.45E+02 | 1.47E+02 | 11.1 | x | x | x |
| 277.1159 | 1.52 | 1.38E+03 | 1.28E+03 | 1.46E+03 | 1.39E+03 | 1.44E+03 | 1.77E+03 | 1.68E+03 | 1.52E+03 | 1.55E+03 | 2.03E+03 | 1.73E+03 | 1.92E+03 | 2.54E+03 | 2.19E+03 | 2.42E+03 | 2.60E+03 | 2.49E+03 | 24.4 |  |  | x |
| 382.0516 | 1.52 | 5.54E+02 | 5.04E+02 | 4.32E+02 | 6.41E+02 | 6.86E+02 | 7.13E+02 | 5.75E+02 | 7.06E+02 | 6.48E+02 | 6.14E+02 | 7.00E+02 | 6.58E+02 | 7.25E+02 | 7.07E+02 | 6.46E+02 | 7.22E+02 | 6.75E+02 | 12.9 | x | x | x |
| 371.2403 | 1.52 | 2.10E+01 | 2.39E+01 | 1.89E+01 | 1.32E+01 | 2.08E+01 | 1.92E+01 | 1.48E+01 | 1.85E+01 | 1.92E+01 | 1.48E+01 | 1.64E+01 | 1.87E+01 | 2.39E+01 | 2.00E+01 | 2.60E+01 | 2.51E+01 | 3.18E+01 | 22.9 |  |  | x |
| 90.0556 | 1.53 | 9.40E+02 | 9.47E+02 | 9.09E+02 | 8.26E+02 | 8.02E+02 | 1.15E+03 | 8.85E+02 | 8.82E+02 | 8.53E+02 | 1.03E+03 | 8.94E+02 | 9.86E+02 | 9.25E+02 | 8.68E+02 | 8.56E+02 | 8.72E+02 | 8.52E+02 | 9.2 | x | x | x |
| 92.0502 | 1.53 | 7.92E+01 | 7.09E+01 | 7.25E+01 | 6.09E+01 | 6.60E+01 | 8.87E+01 | 6.95E+01 | 7.39E+01 | 7.32E+01 | 7.76E+01 | 6.24E+01 | 6.82E+01 | 5.12E+01 | 5.70E+01 | 5.38E+01 | 4.75E+01 | 5.49E+01 | 16.8 |  | x | x |
| 94.0656 | 1.53 | 1.30E+02 | 1.28E+02 | 1.20E+02 | 1.07E+02 | 1.06E+02 | 1.49E+02 | 1.27E+02 | 1.20E+02 | 1.14E+02 | 1.35E+02 | 1.21E+02 | 1.24E+02 | 1.10E+02 | 1.15E+02 | 1.04E+02 | 1.01E+02 | 9.73E+01 | 11.3 | x | x | x |
| 112.0519 | 1.53 | 7.85E+02 | 6.71E+02 | 6.37E+02 | 7.84E+02 | 7.36E+02 | 9.38E+02 | 8.73E+02 | 8.52E+02 | 8.17E+02 | 1.02E+03 | 8.72E+02 | 9.24E+02 | 8.83E+02 | 8.22E+02 | 7.89E+02 | 8.21E+02 | 8.11E+02 | 11.3 | x | x | x |
| 132.0765 | 1.53 | 6.57E+03 | 6.46E+03 | 6.18E+03 | 5.94E+03 | 5.77E+03 | 7.54E+03 | 6.33E+03 | 6.40E+03 | 6.06E+03 | 7.22E+03 | 6.27E+03 | 6.59E+03 | 6.39E+03 | 6.17E+03 | 5.95E+03 | 6.18E+03 | 6.02E+03 | 7.1 | x | x | x |
| 138.0556 | 1.53 | 3.53E+04 | 3.36E+04 | 3.50E+04 | 3.52E+04 | 3.52E+04 | 4.87E+04 | 3.77E+04 | 3.92E+04 | 3.76E+04 | 4.55E+04 | 4.03E+04 | 4.47E+04 | 4.34E+04 | 4.15E+04 | 4.02E+04 | 4.30E+04 | 4.27E+04 | 10.9 | x | x | x |
| 162.1126 | 1.53 | 3.93E+01 | 4.55E+01 | 4.28E+01 | 2.96E+01 | 3.84E+01 | 3.77E+01 | 4.26E+01 | 3.88E+01 | 3.31E+01 | 4.39E+01 | 3.23E+01 | 3.60E+01 | 2.97E+01 | 2.08E+01 | 4.13E+01 | 3.94E+01 | 2.03E+01 | 20.8 |  |  | x |
| 192.0152 | 1.53 | 9.45E+01 | 1.05E+02 | 9.37E+01 | 8.63E+01 | 8.34E+01 | 1.18E+02 | 9.29E+01 | 9.58E+01 | 9.45E+01 | 1.05E+02 | 9.12E+01 | 9.98E+01 | 1.10E+02 | 1.00E+02 | 1.03E+02 | 9.03E+01 | 8.35E+01 | 9.6 | x | x | x |
| 212.1036 | 1.53 | 2.09E+04 | 1.94E+04 | 2.06E+04 | 2.16E+04 | 2.15E+04 | 2.91E+04 | 2.33E+04 | 2.48E+04 | 2.46E+04 | 2.92E+04 | 2.69E+04 | 2.99E+04 | 2.76E+04 | 2.82E+04 | 2.63E+04 | 2.84E+04 | 2.86E+04 | 13.9 | x | x | x |
| 455.1889 | 1.53 | 7.40E+02 | 4.90E+02 | 6.43E+02 | 8.68E+02 | 8.37E+02 | 1.23E+03 | 9.58E+02 | 1.01E+03 | 1.07E+03 | 1.26E+03 | 1.13E+03 | 1.17E+03 | 1.10E+03 | 8.73E+02 | 8.42E+02 | 8.70E+02 | 8.30E+02 | 22.4 |  |  | x |
| 134.0292 | 1.54 | 7.76E+02 | 6.86E+02 | 7.55E+02 | 7.98E+02 | 8.04E+02 | 8.56E+02 | 7.97E+02 | 8.34E+02 | 7.94E+02 | 8.84E+02 | 8.81E+02 | 8.80E+02 | 1.11E+03 | 9.03E+02 | 9.44E+02 | 9.85E+02 | 9.19E+02 | 11.4 | x | x | x |
| 168.1130 | 1.54 | 2.72E+02 | 2.62E+02 | 2.83E+02 | 2.74E+02 | 2.60E+02 | 3.03E+02 | 2.93E+02 | 2.85E+02 | 3.03E+02 | 3.46E+02 | 2.94E+02 | 3.58E+02 | 3.14E+02 | 3.20E+02 | 3.00E+02 | 3.05E+02 | 3.16E+02 | 8.9 | x | x | x |
| 217.1168 | 1.54 | 1.69E+03 | 1.59E+03 | 1.60E+03 | 1.67E+03 | 1.62E+03 | 1.83E+03 | 1.75E+03 | 1.77E+03 | 1.71E+03 | 1.92E+03 | 1.76E+03 | 1.86E+03 | 1.99E+03 | 1.88E+03 | 1.86E+03 | 2.00E+03 | 1.92E+03 | 7.4 | x | x | x |
| 250.0794 | 1.54 | 3.39E+03 | 2.97E+03 | 3.25E+03 | 3.60E+03 | 3.55E+03 | 3.66E+03 | 3.74E+03 | 3.78E+03 | 3.58E+03 | 4.06E+03 | 4.07E+03 | 4.17E+03 | 5.10E+03 | 4.36E+03 | 4.48E+03 | 4.89E+03 | 4.50E+03 | 14.6 | x | x | x |
| 231.1715 | 1.54 | 3.12E+02 | 2.91E+02 | 2.80E+02 | 2.53E+02 | 2.29E+02 | 2.95E+02 | 2.64E+02 | 2.48E+02 | 2.16E+02 | 2.76E+02 | 2.28E+02 | 2.27E+02 | 1.91E+02 | 2.00E+02 | 1.65E+02 | 1.62E+02 | 1.69E+02 | 20.1 |  |  | x |
| 234.0857 | 1.54 | 1.87E+03 | 1.83E+03 | 1.80E+03 | 1.74E+03 | 1.71E+03 | 2.27E+03 | 1.82E+03 | 1.93E+03 | 1.83E+03 | 2.20E+03 | 2.01E+03 | 2.17E+03 | 1.99E+03 | 2.16E+03 | 1.95E+03 | 2.07E+03 | 2.05E+03 | 8.6 | x | x | x |
| 245.1599 | 1.54 | 5.28E+01 | 5.47E+01 | 4.43E+01 | 3.28E+01 | 3.79E+01 | 4.13E+01 | 4.78E+01 | 4.70E+01 | 4.41E+01 | 4.29E+01 | 3.88E+01 | 3.63E+01 | 4.06E+01 | 3.53E+01 | 4.22E+01 | 4.48E+01 | 3.45E+01 | 14.5 | x | x | x |
| 256.0686 | 1.54 | 4.32E+03 | 4.30E+03 | 4.09E+03 | 4.18E+03 | 4.16E+03 | 6.03E+03 | 4.78E+03 | 4.94E+03 | 4.63E+03 | 5.84E+03 | 5.36E+03 | 5.57E+03 | 5.48E+03 | 5.97E+03 | 5.25E+03 | 5.83E+03 | 5.90E+03 | 14.0 | x | x | x |
| 267.1246 | 1.54 | 1.18E+02 | 1.05E+02 | 1.15E+02 | 9.78E+01 | 1.07E+02 | 1.19E+02 | 1.07E+02 | 1.11E+02 | 1.02E+02 | 1.32E+02 | 1.01E+02 | 1.14E+02 | 1.26E+02 | 1.14E+02 | 1.12E+02 | 1.51E+02 | 1.03E+02 | 11.5 | x | x | x |
| 387.1298 | 1.54 | 2.40E+02 | 2.47E+02 | 2.26E+02 | 1.95E+02 | 2.10E+02 | 2.36E+02 | 2.12E+02 | 2.17E+02 | 1.90E+02 | 2.26E+02 | 2.26E+02 | 2.20E+02 | 2.32E+02 | 2.37E+02 | 2.41E+02 | 2.25E+02 | 2.20E+02 | 7.0 | x | x | x |
| 409.1180 | 1.54 | 1.65E+02 | 1.72E+02 | 1.34E+02 | 1.26E+02 | 1.12E+02 | 2.16E+02 | 1.28E+02 | 1.46E+02 | 1.22E+02 | 1.34E+02 | 1.58E+02 | 9.43E+01 | 1.18E+02 | 1.61E+02 | 7.22E+01 | 1.07E+02 | 9.76E+01 | 25.9 |  |  | x |
| 123.0484 | 1.55 | 1.53E+02 | 1.59E+02 | 1.52E+02 | 1.44E+02 | 1.46E+02 | 1.58E+02 | 1.59E+02 | 1.51E+02 | 1.73E+02 | 1.76E+02 | 1.69E+02 | 1.70E+02 | 1.69E+02 | 1.69E+02 | 1.71E+02 | 1.83E+02 | 1.63E+02 | 6.9 | x | x | x |
| 116.0478 | 1.55 | 2.34E+03 | 2.25E+03 | 2.33E+03 | 2.35E+03 | 2.37E+03 | 2.91E+03 | 2.47E+03 | 2.52E+03 | 2.35E+03 | 2.77E+03 | 2.54E+03 | 2.69E+03 | 3.08E+03 | 2.99E+03 | 2.91E+03 | 3.19E+03 | 3.14E+03 | 12.1 | x | x | x |
| 147.0446 | 1.55 | 2.20E+02 | 2.00E+02 | 1.92E+02 | 1.98E+02 | 2.10E+02 | 2.83E+02 | 2.20E+02 | 2.26E+02 | 2.27E+02 | 2.50E+02 | 2.55E+02 | 2.35E+02 | 2.24E+02 | 2.20E+02 | 1.88E+02 | 1.90E+02 | 1.89E+02 | 12.0 | x | x | x |
| 132.0215 | 1.55 | 5.08E+02 | 5.15E+02 | 5.38E+02 | 5.78E+02 | 5.65E+02 | 6.95E+02 | 5.94E+02 | 6.37E+02 | 5.91E+02 | 6.65E+02 | 6.19E+02 | 7.08E+02 | 8.53E+02 | 6.98E+02 | 8.39E+02 | 8.38E+02 | 7.82E+02 | 17.2 |  | x | x |
| 160.0372 | 1.55 | 9.39E+03 | 9.06E+03 | 9.58E+03 | 9.71E+03 | 9.88E+03 | 1.22E+04 | 1.04E+04 | 1.05E+04 | 9.86E+03 | 1.19E+04 | 1.12E+04 | 1.20E+04 | 1.42E+04 | 1.33E+04 | 1.36E+04 | 1.47E+04 | 1.43E+04 | 16.5 |  | x | x |
| 185.1142 | 1.55 | 3.90E+02 | 3.53E+02 | 3.47E+02 | 3.30E+02 | 3.21E+02 | 3.69E+02 | 3.71E+02 | 3.68E+02 | 3.09E+02 | 3.45E+02 | 3.01E+02 | 2.83E+02 | 2.95E+02 | 2.95E+02 | 2.65E+02 | 2.89E+02 | 2.35E+02 | 13.2 | x | x | x |
| 359.2382 | 1.55 | 1.18E+02 | 1.09E+02 | 1.01E+02 | 1.11E+02 | 1.12E+02 | 1.66E+02 | 1.09E+02 | 1.28E+02 | 1.33E+02 | 1.30E+02 | 1.33E+02 | 1.07E+02 | 1.23E+02 | 9.45E+01 | 1.10E+02 | 1.08E+02 | 8.33E+01 | 16.0 |  | x | x |
| 84.0811 | 1.56 | 1.91E+02 | 1.85E+02 | 2.04E+02 | 2.12E+02 | 2.17E+02 | 2.44E+02 | 2.28E+02 | 2.41E+02 | 2.41E+02 | 2.68E+02 | 2.35E+02 | 2.81E+02 | 3.10E+02 | 3.16E+02 | 3.08E+02 | 3.63E+02 | 3.51E+02 | 21.1 |  |  | x |
| 130.0862 | 1.56 | 6.59E+02 | 6.23E+02 | 6.41E+02 | 6.68E+02 | 6.85E+02 | 7.94E+02 | 7.42E+02 | 7.76E+02 | 7.68E+02 | 8.36E+02 | 7.62E+02 | 8.76E+02 | 9.34E+02 | 9.83E+02 | 9.48E+02 | 1.05E+03 | 1.04E+03 | 17.2 |  | x | x |
| 152.0795 | 1.56 | 1.33E+02 | 1.31E+02 | 1.30E+02 | 1.25E+02 | 1.29E+02 | 1.42E+02 | 1.37E+02 | 1.42E+02 | 1.30E+02 | 1.53E+02 | 1.39E+02 | 1.50E+02 | 1.65E+02 | 1.51E+02 | 1.52E+02 | 1.80E+02 | 1.64E+02 | 10.7 | x | x | x |
| 150.0777 | 1.56 | 1.91E+01 | 9.11E+00 | 2.90E+01 | 2.12E+01 | 2.16E+01 | 2.38E+01 | 1.73E+01 | 2.62E+01 | 2.23E+01 | 1.41E+01 | 2.13E+01 | 2.40E+01 | 2.41E+01 | 2.67E+01 | 2.09E+01 | 2.11E+01 | 2.10E+01 | 22.1 |  |  | x |
| 226.0458 | 1.56 | 6.03E+01 | 5.79E+01 | 6.68E+01 | 7.03E+01 | 7.65E+01 | 1.01E+02 | 6.76E+01 | 8.06E+01 | 7.13E+01 | 7.95E+01 | 7.34E+01 | 7.50E+01 | 6.31E+01 | 5.12E+01 | 4.37E+01 | 4.72E+01 | 4.10E+01 | 23.0 |  |  | x |
| 229.1196 | 1.56 | 2.52E+02 | 2.37E+02 | 2.37E+02 | 2.17E+02 | 2.24E+02 | 2.56E+02 | 2.48E+02 | 2.57E+02 | 2.48E+02 | 2.71E+02 | 2.49E+02 | 2.54E+02 | 2.63E+02 | 2.45E+02 | 2.37E+02 | 2.16E+02 | 2.53E+02 | 6.3 | x | x | x |
| 242.0527 | 1.56 | 3.25E+02 | 3.18E+02 | 3.08E+02 | 3.16E+02 | 3.52E+02 | 4.57E+02 | 3.79E+02 | 3.83E+02 | 3.88E+02 | 4.56E+02 | 4.18E+02 | 4.47E+02 | 5.14E+02 | 4.40E+02 | 4.40E+02 | 4.70E+02 | 4.67E+02 | 15.8 |  | x | x |
| 253.0967 | 1.56 | 1.16E+02 | 1.18E+02 | 1.18E+02 | 9.99E+01 | 9.36E+01 | 1.55E+02 | 1.07E+02 | 1.12E+02 | 9.09E+01 | 1.12E+02 | 1.06E+02 | 8.04E+01 | 9.42E+01 | 1.12E+02 | 8.35E+01 | 9.57E+01 | 7.56E+01 | 17.9 |  | x | x |
| 272.1358 | 1.56 | 7.67E+02 | 6.94E+02 | 7.98E+02 | 8.45E+02 | 8.64E+02 | 9.85E+02 | 9.41E+02 | 9.88E+02 | 9.61E+02 | 1.10E+03 | 1.05E+03 | 1.16E+03 | 1.34E+03 | 1.27E+03 | 1.33E+03 | 1.39E+03 | 1.34E+03 | 21.3 |  |  | x |
| 282.1198 | 1.56 | 8.08E+02 | 7.86E+02 | 8.40E+02 | 7.63E+02 | 8.21E+02 | 8.09E+02 | 8.48E+02 | 8.80E+02 | 8.54E+02 | 9.56E+02 | 8.76E+02 | 9.19E+02 | 9.82E+02 | 9.34E+02 | 9.45E+02 | 1.02E+03 | 9.99E+02 | 8.8 | x | x | x |
| 297.0855 | 1.56 | 5.92E+02 | 5.38E+02 | 5.81E+02 | 4.70E+02 | 5.14E+02 | 7.50E+02 | 6.47E+02 | 5.39E+02 | 5.27E+02 | 7.50E+02 | 5.74E+02 | 5.72E+02 | 7.66E+02 | 7.42E+02 | 7.75E+02 | 8.06E+02 | 7.96E+02 | 17.9 |  | x | x |
| 301.0778 | 1.56 | 5.56E+02 | 5.15E+02 | 4.95E+02 | 5.01E+02 | 5.03E+02 | 6.05E+02 | 5.32E+02 | 5.32E+02 | 5.37E+02 | 5.62E+02 | 5.34E+02 | 5.13E+02 | 5.19E+02 | 5.07E+02 | 4.35E+02 | 4.66E+02 | 4.37E+02 | 8.3 | x | x | x |
| 309.1932 | 1.56 | 4.73E+01 | 4.23E+01 | 4.30E+01 | 4.95E+01 | 4.30E+01 | 4.46E+01 | 5.26E+01 | 4.74E+01 | 4.67E+01 | 4.09E+01 | 4.74E+01 | 3.96E+01 | 4.58E+01 | 3.82E+01 | 3.32E+01 | 2.51E+01 | 2.61E+01 | 18.3 |  | x | x |
| 393.1061 | 1.56 | 1.36E+02 | 1.38E+02 | 1.16E+02 | 9.29E+01 | 1.08E+02 | 1.42E+02 | 1.01E+02 | 1.16E+02 | 1.11E+02 | 1.16E+02 | 1.25E+02 | 9.90E+01 | 8.76E+01 | 1.07E+02 | 9.90E+01 | 1.08E+02 | 1.08E+02 | 13.7 | x | x | x |
| 629.3126 | 1.56 | 6.11E+01 | 6.04E+01 | 6.21E+01 | 5.58E+01 | 5.50E+01 | 5.96E+01 | 5.14E+01 | 6.57E+01 | 6.27E+01 | 6.50E+01 | 6.32E+01 | 6.48E+01 | 6.60E+01 | 4.60E+01 | 5.69E+01 | 6.52E+01 | 4.61E+01 | 11.0 | x | x | x |
| 83.0223 | 1.58 | 2.07E+01 | 2.32E+01 | 1.29E+01 | 1.47E+01 | 2.23E+01 | 2.81E+01 | 2.01E+01 | 2.05E+01 | 1.73E+01 | 1.94E+01 | 1.80E+01 | 2.04E+01 | 2.72E+01 | 2.64E+01 | 1.29E+01 | 1.98E+01 | 1.21E+01 | 24.5 |  |  | x |
| 173.0808 | 1.58 | 1.42E+02 | 1.26E+02 | 1.25E+02 | 1.20E+02 | 1.09E+02 | 1.15E+02 | 1.23E+02 | 1.22E+02 | 1.25E+02 | 1.29E+02 | 1.02E+02 | 1.06E+02 | 1.03E+02 | 1.03E+02 | 9.69E+01 | 1.01E+02 | 9.16E+01 | 12.0 | x | x | x |
| 180.0765 | 1.58 | 1.74E+02 | 1.67E+02 | 1.66E+02 | 1.55E+02 | 1.56E+02 | 1.50E+02 | 1.72E+02 | 1.64E+02 | 1.68E+02 | 1.74E+02 | 1.46E+02 | 1.46E+02 | 1.53E+02 | 1.72E+02 | 1.52E+02 | 1.55E+02 | 1.44E+02 | 6.6 | x | x | x |
| 237.0002 | 1.58 | 7.79E+02 | 7.88E+02 | 7.86E+02 | 8.06E+02 | 6.97E+02 | 7.54E+02 | 6.50E+02 | 6.55E+02 | 6.41E+02 | 6.44E+02 | 6.14E+02 | 6.27E+02 | 6.71E+02 | 6.25E+02 | 5.83E+02 | 6.08E+02 | 5.45E+02 | 11.8 | x | x | x |
| 259.0931 | 1.58 | 9.81E+02 | 1.03E+03 | 9.18E+02 | 8.83E+02 | 8.66E+02 | 8.89E+02 | 9.09E+02 | 9.62E+02 | 9.25E+02 | 1.01E+03 | 9.23E+02 | 9.81E+02 | 1.05E+03 | 1.01E+03 | 9.75E+02 | 1.06E+03 | 1.02E+03 | 6.3 | x | x | x |
| 407.1996 | 1.58 | 4.51E+01 | 3.61E+01 | 3.16E+01 | 3.45E+01 | 2.98E+01 | 3.44E+01 | 3.94E+01 | 4.15E+01 | 3.76E+01 | 4.11E+01 | 3.56E+01 | 3.61E+01 | 2.86E+01 | 3.21E+01 | 2.78E+01 | 3.08E+01 | 3.17E+01 | 14.0 | x | x | x |
| 435.0364 | 1.58 | 5.09E+01 | 6.58E+01 | 5.20E+01 | 4.15E+01 | 4.99E+01 | 8.62E+01 | 7.27E+01 | 6.67E+01 | 6.09E+01 | 7.69E+01 | 7.32E+01 | 8.17E+01 | 7.18E+01 | 6.66E+01 | 7.00E+01 | 6.21E+01 | 5.88E+01 | 18.3 |  | x | x |
| 487.1787 | 1.58 | 3.07E+01 | 2.75E+01 | 2.63E+01 | 2.55E+01 | 2.77E+01 | 2.13E+01 | 2.49E+01 | 2.23E+01 | 2.77E+01 | 2.94E+01 | 2.77E+01 | 3.23E+01 | 2.46E+01 | 2.35E+01 | 2.04E+01 | 3.87E+01 | 2.23E+01 | 17.0 |  | x | x |
| 203.0540 | 1.59 | 2.73E+02 | 2.60E+02 | 2.22E+02 | 2.34E+02 | 1.92E+02 | 1.90E+02 | 2.34E+02 | 2.01E+02 | 2.06E+02 | 2.03E+02 | 1.93E+02 | 1.70E+02 | 1.99E+02 | 1.55E+02 | 1.35E+02 | 1.40E+02 | 1.15E+02 | 22.0 |  |  | x |
| 205.0688 | 1.59 | 1.43E+03 | 1.35E+03 | 1.33E+03 | 1.24E+03 | 1.24E+03 | 1.50E+03 | 1.37E+03 | 1.35E+03 | 1.29E+03 | 1.44E+03 | 1.18E+03 | 1.37E+03 | 1.20E+03 | 1.39E+03 | 1.10E+03 | 1.34E+03 | 1.23E+03 | 7.9 | x | x | x |
| 218.1484 | 1.59 | 8.18E+01 | 8.66E+01 | 7.62E+01 | 7.28E+01 | 6.68E+01 | 9.19E+01 | 7.06E+01 | 7.92E+01 | 7.76E+01 | 9.16E+01 | 7.82E+01 | 7.33E+01 | 8.28E+01 | 6.99E+01 | 8.32E+01 | 8.17E+01 | 7.11E+01 | 9.4 | x | x | x |
| 305.1693 | 1.59 | 1.22E+02 | 1.18E+02 | 1.13E+02 | 1.05E+02 | 1.12E+02 | 1.23E+02 | 1.19E+02 | 1.23E+02 | 1.19E+02 | 1.37E+02 | 1.28E+02 | 1.09E+02 | 1.22E+02 | 1.11E+02 | 1.11E+02 | 1.23E+02 | 8.93E+01 | 9.0 | x | x | x |
| 152.0581 | 1.60 | 8.87E+02 | 8.36E+02 | 8.54E+02 | 8.63E+02 | 9.07E+02 | 1.04E+03 | 9.99E+02 | 9.52E+02 | 9.83E+02 | 1.10E+03 | 9.99E+02 | 1.11E+03 | 1.09E+03 | 1.09E+03 | 1.07E+03 | 1.12E+03 | 1.08E+03 | 9.8 | x | x | x |
| 134.0318 | 1.60 | 1.37E+02 | 1.27E+02 | 1.18E+02 | 1.34E+02 | 1.25E+02 | 1.42E+02 | 1.39E+02 | 1.31E+02 | 1.20E+02 | 1.52E+02 | 1.29E+02 | 1.27E+02 | 1.30E+02 | 1.40E+02 | 1.08E+02 | 1.18E+02 | 1.10E+02 | 9.1 | x | x | x |
| 266.0751 | 1.60 | 2.34E+03 | 2.16E+03 | 2.25E+03 | 2.54E+03 | 2.59E+03 | 3.47E+03 | 2.87E+03 | 2.78E+03 | 2.87E+03 | 3.30E+03 | 3.05E+03 | 3.19E+03 | 3.48E+03 | 3.47E+03 | 3.32E+03 | 3.46E+03 | 3.51E+03 | 15.8 |  | x | x |
| 245.0755 | 1.60 | 7.09E+02 | 6.57E+02 | 6.93E+02 | 6.66E+02 | 6.75E+02 | 7.10E+02 | 7.12E+02 | 6.84E+02 | 6.55E+02 | 7.14E+02 | 6.00E+02 | 5.84E+02 | 6.54E+02 | 6.39E+02 | 6.00E+02 | 5.90E+02 | 5.23E+02 | 8.4 | x | x | x |
| 322.0556 | 1.60 | 4.00E+02 | 3.80E+02 | 4.10E+02 | 4.41E+02 | 4.76E+02 | 5.59E+02 | 5.01E+02 | 5.57E+02 | 5.76E+02 | 6.35E+02 | 6.44E+02 | 5.85E+02 | 6.97E+02 | 6.32E+02 | 6.35E+02 | 6.74E+02 | 6.32E+02 | 18.4 |  | x | x |
| 189.0936 | 1.61 | 1.93E+02 | 1.64E+02 | 1.75E+02 | 1.83E+02 | 1.67E+02 | 2.00E+02 | 1.65E+02 | 1.57E+02 | 1.51E+02 | 1.58E+02 | 1.38E+02 | 1.48E+02 | 1.61E+02 | 1.46E+02 | 1.09E+02 | 1.27E+02 | 1.32E+02 | 15.0 | x | x | x |
| 242.0669 | 1.61 | 1.00E+02 | 1.01E+02 | 1.18E+02 | 1.03E+02 | 1.07E+02 | 1.28E+02 | 1.14E+02 | 1.14E+02 | 1.22E+02 | 1.15E+02 | 8.77E+01 | 1.14E+02 | 1.12E+02 | 1.11E+02 | 1.32E+02 | 1.09E+02 | 1.04E+02 | 9.7 | x | x | x |
| 509.1609 | 1.61 | 1.09E+01 | 1.66E+01 | 1.42E+01 | 1.72E+01 | 1.03E+01 | 9.98E+00 | 1.76E+01 | 1.40E+01 | 2.13E+01 | 2.12E+01 | 1.13E+01 | 1.24E+01 | 1.41E+01 | 7.48E+00 | 1.10E+01 | 1.22E+01 | 8.52E+00 | 30.0 |  |  | x |
| 563.2227 | 1.61 | 1.69E+02 | 1.74E+02 | 1.72E+02 | 1.47E+02 | 1.20E+02 | 1.32E+02 | 1.63E+02 | 1.52E+02 | 1.63E+02 | 1.50E+02 | 1.59E+02 | 1.42E+02 | 9.52E+01 | 7.15E+01 | 1.07E+02 | 9.11E+01 | 8.03E+01 | 25.3 |  |  | x |
| 198.0871 | 1.62 | 6.50E+02 | 6.47E+02 | 6.61E+02 | 6.42E+02 | 6.31E+02 | 7.03E+02 | 6.62E+02 | 6.86E+02 | 7.00E+02 | 7.34E+02 | 6.90E+02 | 7.73E+02 | 8.37E+02 | 7.92E+02 | 7.93E+02 | 8.90E+02 | 8.70E+02 | 11.5 | x | x | x |
| 213.1028 | 1.62 | 8.24E+02 | 8.20E+02 | 8.27E+02 | 7.30E+02 | 7.48E+02 | 7.91E+02 | 7.69E+02 | 8.09E+02 | 7.67E+02 | 7.97E+02 | 7.58E+02 | 7.55E+02 | 6.52E+02 | 6.37E+02 | 6.51E+02 | 6.63E+02 | 6.08E+02 | 9.8 | x | x | x |
| 261.0495 | 1.62 | 3.57E+02 | 3.68E+02 | 3.77E+02 | 4.07E+02 | 4.43E+02 | 5.42E+02 | 4.37E+02 | 4.94E+02 | 4.76E+02 | 5.35E+02 | 4.92E+02 | 5.02E+02 | 5.31E+02 | 5.31E+02 | 5.49E+02 | 5.35E+02 | 4.99E+02 | 13.7 | x | x | x |
| 293.1695 | 1.62 | 3.34E+01 | 3.16E+01 | 3.21E+01 | 2.58E+01 | 2.47E+01 | 3.14E+01 | 3.36E+01 | 3.04E+01 | 3.21E+01 | 2.72E+01 | 2.07E+01 | 1.94E+01 | 2.70E+01 | 2.79E+01 | 2.61E+01 | 1.72E+01 | 8.68E+00 | 25.4 |  |  | x |
| 339.1235 | 1.62 | 2.64E+02 | 2.73E+02 | 2.66E+02 | 2.92E+02 | 2.58E+02 | 2.92E+02 | 2.78E+02 | 3.02E+02 | 2.99E+02 | 2.99E+02 | 3.07E+02 | 2.87E+02 | 2.88E+02 | 2.19E+02 | 2.66E+02 | 2.22E+02 | 2.20E+02 | 10.6 | x | x | x |
| 176.0674 | 1.63 | 2.54E+01 | 3.08E+01 | 3.09E+01 | 3.43E+01 | 3.05E+01 | 3.27E+01 | 3.07E+01 | 3.09E+01 | 3.08E+01 | 2.93E+01 | 2.04E+01 | 2.81E+01 | 2.83E+01 | 2.79E+01 | 3.04E+01 | 3.04E+01 | 1.77E+01 | 14.6 | x | x | x |
| 156.0768 | 1.63 | 5.00E+02 | 4.99E+02 | 5.35E+02 | 5.37E+02 | 5.73E+02 | 7.52E+02 | 5.94E+02 | 6.44E+02 | 6.64E+02 | 7.47E+02 | 6.92E+02 | 7.02E+02 | 8.14E+02 | 7.16E+02 | 7.70E+02 | 8.19E+02 | 7.59E+02 | 16.2 |  | x | x |
| 223.1175 | 1.63 | 2.85E+02 | 2.84E+02 | 3.02E+02 | 2.88E+02 | 3.12E+02 | 3.50E+02 | 3.17E+02 | 3.47E+02 | 3.40E+02 | 3.39E+02 | 3.22E+02 | 3.32E+02 | 3.55E+02 | 3.36E+02 | 3.41E+02 | 3.36E+02 | 3.27E+02 | 7.1 | x | x | x |
| 266.1600 | 1.63 | 2.57E+03 | 2.70E+03 | 2.62E+03 | 2.65E+03 | 2.65E+03 | 3.38E+03 | 2.73E+03 | 2.83E+03 | 2.82E+03 | 3.30E+03 | 3.02E+03 | 3.18E+03 | 3.81E+03 | 3.53E+03 | 3.66E+03 | 3.88E+03 | 3.80E+03 | 15.1 |  | x | x |
| 269.1249 | 1.63 | 4.53E+03 | 4.68E+03 | 4.48E+03 | 4.96E+03 | 5.32E+03 | 6.93E+03 | 5.44E+03 | 5.76E+03 | 5.89E+03 | 6.98E+03 | 6.52E+03 | 6.83E+03 | 7.62E+03 | 7.17E+03 | 7.41E+03 | 7.96E+03 | 7.60E+03 | 18.9 |  | x | x |
| 104.0708 | 1.64 | 4.67E+01 | 4.34E+01 | 4.57E+01 | 5.05E+01 | 5.19E+01 | 6.73E+01 | 5.52E+01 | 5.73E+01 | 5.52E+01 | 6.29E+01 | 5.33E+01 | 5.73E+01 | 6.55E+01 | 6.33E+01 | 6.28E+01 | 6.39E+01 | 7.23E+01 | 14.3 | x | x | x |
| 178.0937 | 1.64 | 4.95E+01 | 5.22E+01 | 5.24E+01 | 5.44E+01 | 4.94E+01 | 5.22E+01 | 5.46E+01 | 5.29E+01 | 4.73E+01 | 6.15E+01 | 4.70E+01 | 5.36E+01 | 4.99E+01 | 4.52E+01 | 5.70E+01 | 5.29E+01 | 4.15E+01 | 9.0 | x | x | x |
| 100.0760 | 1.65 | 7.70E+01 | 7.11E+01 | 7.06E+01 | 7.77E+01 | 7.41E+01 | 7.43E+01 | 8.09E+01 | 7.59E+01 | 7.86E+01 | 8.35E+01 | 7.72E+01 | 7.79E+01 | 8.22E+01 | 7.98E+01 | 7.97E+01 | 9.59E+01 | 7.80E+01 | 7.2 | x | x | x |
| 124.0398 | 1.65 | 1.05E+02 | 1.12E+02 | 9.91E+01 | 9.79E+01 | 9.24E+01 | 1.11E+02 | 9.83E+01 | 9.48E+01 | 1.02E+02 | 1.22E+02 | 9.61E+01 | 1.12E+02 | 1.16E+02 | 1.08E+02 | 1.00E+02 | 1.07E+02 | 1.05E+02 | 7.8 | x | x | x |
| 128.0620 | 1.65 | 2.08E+02 | 2.06E+02 | 1.44E+02 | 1.51E+02 | 1.30E+02 | 1.58E+02 | 1.58E+02 | 1.48E+02 | 1.32E+02 | 1.62E+02 | 1.20E+02 | 1.26E+02 | 8.23E+01 | 1.24E+02 | 8.02E+01 | 8.85E+01 | 7.23E+01 | 29.4 |  |  | x |
| 146.0820 | 1.65 | 2.17E+02 | 2.27E+02 | 2.12E+02 | 2.24E+02 | 2.29E+02 | 2.64E+02 | 2.36E+02 | 2.42E+02 | 2.29E+02 | 2.56E+02 | 2.22E+02 | 2.30E+02 | 2.44E+02 | 2.41E+02 | 2.31E+02 | 2.45E+02 | 2.29E+02 | 5.8 | x | x | x |
| 169.0363 | 1.65 | 4.97E+01 | 4.63E+01 | 5.40E+01 | 5.04E+01 | 5.48E+01 | 6.75E+01 | 6.12E+01 | 6.09E+01 | 6.19E+01 | 6.52E+01 | 6.61E+01 | 6.09E+01 | 6.35E+01 | 6.19E+01 | 6.11E+01 | 5.69E+01 | 5.56E+01 | 10.4 | x | x | x |
| 290.1249 | 1.65 | 2.17E+02 | 2.36E+02 | 2.28E+02 | 2.11E+02 | 2.67E+02 | 3.22E+02 | 2.66E+02 | 2.68E+02 | 2.90E+02 | 3.11E+02 | 3.04E+02 | 3.13E+02 | 3.52E+02 | 3.86E+02 | 3.67E+02 | 3.79E+02 | 3.29E+02 | 18.8 |  | x | x |
| 354.1185 | 1.65 | 1.53E+02 | 1.72E+02 | 1.05E+02 | 1.41E+02 | 1.51E+02 | 1.45E+02 | 1.10E+02 | 1.25E+02 | 1.25E+02 | 9.81E+01 | 1.10E+02 | 8.08E+01 | 9.10E+01 | 8.88E+01 | 7.24E+01 | 8.02E+01 | 6.38E+01 | 28.3 |  |  | x |
| 286.1297 | 1.66 | 2.55E+02 | 2.55E+02 | 2.12E+02 | 2.22E+02 | 2.09E+02 | 1.97E+02 | 2.43E+02 | 2.38E+02 | 2.05E+02 | 2.42E+02 | 2.08E+02 | 1.96E+02 | 1.80E+02 | 1.67E+02 | 1.93E+02 | 1.81E+02 | 1.57E+02 | 14.2 | x | x | x |
| 400.1529 | 1.66 | 5.63E+01 | 5.94E+01 | 5.51E+01 | 5.90E+01 | 4.52E+01 | 5.20E+01 | 5.57E+01 | 6.48E+01 | 5.47E+01 | 5.33E+01 | 5.38E+01 | 4.10E+01 | 3.34E+01 | 3.50E+01 | 3.59E+01 | 2.64E+01 | 2.66E+01 | 25.5 |  |  | x |
| 321.0622 | 1.67 | 2.21E+01 | 2.04E+01 | 1.75E+01 | 1.90E+01 | 1.98E+01 | 1.96E+01 | 2.51E+01 | 1.51E+01 | 1.00E+01 | 1.44E+01 | 1.02E+01 | 1.15E+01 | 1.32E+01 | 1.45E+01 | 1.39E+01 | 9.80E+00 | 1.22E+01 | 29.1 |  |  | x |
| 124.0873 | 1.68 | 1.65E+04 | 1.54E+04 | 1.61E+04 | 1.74E+04 | 1.79E+04 | 2.27E+04 | 1.91E+04 | 2.00E+04 | 1.92E+04 | 2.23E+04 | 1.98E+04 | 2.27E+04 | 2.19E+04 | 2.31E+04 | 2.17E+04 | 2.49E+04 | 2.44E+04 | 14.5 | x | x | x |
| 277.0906 | 1.68 | 1.02E+03 | 9.74E+02 | 9.84E+02 | 8.96E+02 | 9.17E+02 | 8.94E+02 | 9.64E+02 | 8.88E+02 | 8.07E+02 | 9.14E+02 | 7.80E+02 | 8.08E+02 | 7.46E+02 | 8.24E+02 | 6.47E+02 | 7.26E+02 | 6.74E+02 | 13.0 | x | x | x |
| 251.0372 | 1.69 | 1.58E+01 | 2.22E+01 | 2.49E+01 | 2.27E+01 | 2.10E+01 | 1.69E+01 | 2.21E+01 | 2.28E+01 | 2.03E+01 | 1.93E+01 | 1.54E+01 | 1.50E+01 | 1.05E+01 | 1.83E+01 | 1.32E+01 | 1.78E+01 | 7.88E+00 | 25.8 |  |  | x |
| 156.1493 | 1.69 | 1.02E+01 | 1.73E+01 | 1.58E+01 | 1.69E+01 | 1.45E+01 | 1.66E+01 | 1.86E+01 | 1.80E+01 | 2.00E+01 | 2.07E+01 | 1.98E+01 | 1.91E+01 | 1.80E+01 | 9.45E+00 | 1.77E+01 | 1.23E+01 | 2.56E+01 | 23.0 |  |  | x |
| 169.9865 | 1.69 | 3.62E+01 | 3.44E+01 | 3.15E+01 | 3.13E+01 | 3.03E+01 | 2.72E+01 | 3.41E+01 | 2.93E+01 | 3.01E+01 | 2.59E+01 | 2.62E+01 | 2.75E+01 | 2.22E+01 | 3.12E+01 | 2.24E+01 | 2.16E+01 | 1.39E+01 | 20.1 |  |  | x |
| 293.0644 | 1.69 | 3.19E+02 | 3.22E+02 | 3.10E+02 | 3.51E+02 | 3.80E+02 | 4.89E+02 | 3.80E+02 | 4.08E+02 | 4.26E+02 | 4.76E+02 | 4.21E+02 | 4.56E+02 | 4.48E+02 | 4.66E+02 | 4.44E+02 | 4.32E+02 | 4.09E+02 | 13.8 | x | x | x |
| 314.1209 | 1.69 | 2.96E+02 | 3.52E+02 | 2.79E+02 | 2.79E+02 | 2.31E+02 | 2.04E+02 | 2.33E+02 | 2.61E+02 | 2.41E+02 | 2.28E+02 | 1.94E+02 | 2.08E+02 | 2.30E+02 | 1.62E+02 | 1.94E+02 | 1.99E+02 | 1.75E+02 | 20.7 |  |  | x |
| 332.9569 | 1.69 | 2.26E+01 | 2.47E+01 | 2.21E+01 | 2.40E+01 | 3.35E+01 | 4.02E+01 | 3.90E+01 | 4.02E+01 | 4.20E+01 | 5.30E+01 | 3.51E+01 | 4.05E+01 | 3.86E+01 | 4.68E+01 | 3.99E+01 | 3.05E+01 | 1.57E+01 | 28.8 |  |  | x |
| 547.1066 | 1.71 | 7.61E+01 | 1.07E+02 | 7.39E+01 | 6.02E+01 | 6.71E+01 | 5.39E+01 | 6.01E+01 | 6.25E+01 | 5.37E+01 | 6.08E+01 | 6.96E+01 | 7.34E+01 | 4.94E+01 | 5.21E+01 | 3.72E+01 | 4.67E+01 | 3.56E+01 | 27.5 |  |  | x |
| 302.1314 | 1.72 | 9.83E+01 | 9.42E+01 | 7.96E+01 | 9.68E+01 | 9.33E+01 | 8.73E+01 | 8.06E+01 | 8.20E+01 | 1.01E+02 | 9.79E+01 | 7.21E+01 | 8.57E+01 | 8.07E+01 | 8.11E+01 | 7.63E+01 | 7.15E+01 | 7.19E+01 | 11.7 | x | x | x |
| 332.1300 | 1.72 | 3.08E+03 | 3.23E+03 | 2.77E+03 | 3.33E+03 | 3.00E+03 | 2.98E+03 | 2.70E+03 | 2.94E+03 | 2.76E+03 | 2.99E+03 | 2.67E+03 | 2.81E+03 | 3.16E+03 | 2.45E+03 | 2.58E+03 | 2.85E+03 | 2.24E+03 | 9.9 | x | x | x |
| 194.1056 | 1.74 | 7.70E+02 | 7.79E+02 | 7.61E+02 | 7.14E+02 | 7.18E+02 | 7.52E+02 | 7.37E+02 | 7.43E+02 | 6.92E+02 | 7.07E+02 | 6.84E+02 | 6.97E+02 | 6.82E+02 | 6.19E+02 | 6.43E+02 | 6.43E+02 | 5.83E+02 | 7.9 | x | x | x |
| 178.1211 | 1.76 | 2.64E+02 | 2.66E+02 | 2.68E+02 | 2.65E+02 | 2.74E+02 | 2.73E+02 | 2.69E+02 | 2.88E+02 | 2.73E+02 | 3.00E+02 | 2.66E+02 | 2.61E+02 | 2.83E+02 | 2.64E+02 | 2.78E+02 | 2.57E+02 | 1.96E+02 | 7.9 | x | x | x |
| 114.0667 | 1.78 | 3.53E+02 | 4.34E+02 | 3.62E+02 | 3.99E+02 | 4.08E+02 | 4.51E+02 | 3.70E+02 | 4.20E+02 | 3.89E+02 | 4.06E+02 | 4.03E+02 | 4.37E+02 | 5.10E+02 | 4.15E+02 | 4.41E+02 | 4.50E+02 | 4.66E+02 | 9.5 | x | x | x |
| 260.0890 | 1.78 | 6.32E+02 | 8.57E+02 | 6.05E+02 | 7.46E+02 | 6.53E+02 | 7.07E+02 | 5.83E+02 | 7.08E+02 | 6.37E+02 | 6.38E+02 | 6.47E+02 | 5.68E+02 | 7.25E+02 | 5.97E+02 | 6.07E+02 | 5.87E+02 | 5.49E+02 | 12.0 | x | x | x |
| 418.1570 | 1.80 | 1.04E+03 | 1.01E+03 | 8.65E+02 | 1.03E+03 | 9.31E+02 | 8.52E+02 | 8.26E+02 | 9.34E+02 | 7.86E+02 | 8.56E+02 | 8.47E+02 | 7.96E+02 | 8.13E+02 | 7.10E+02 | 6.91E+02 | 7.45E+02 | 6.49E+02 | 13.6 | x | x | x |
| 367.1496 | 1.80 | 1.27E+03 | 1.23E+03 | 1.17E+03 | 1.26E+03 | 1.31E+03 | 1.48E+03 | 1.30E+03 | 1.40E+03 | 1.26E+03 | 1.44E+03 | 1.29E+03 | 1.36E+03 | 1.41E+03 | 1.34E+03 | 1.25E+03 | 1.36E+03 | 1.23E+03 | 6.3 | x | x | x |
| 351.0729 | 1.80 | 1.80E+02 | 1.74E+02 | 1.65E+02 | 2.20E+02 | 2.49E+02 | 2.60E+02 | 2.29E+02 | 2.65E+02 | 2.17E+02 | 2.59E+02 | 2.29E+02 | 2.31E+02 | 2.28E+02 | 2.36E+02 | 2.08E+02 | 2.27E+02 | 1.85E+02 | 13.8 | x | x | x |
| 387.1803 | 1.82 | 1.63E+01 | 2.09E+01 | 1.86E+01 | 1.56E+01 | 2.51E+01 | 2.22E+01 | 1.66E+01 | 2.53E+01 | 2.55E+01 | 3.34E+01 | 3.21E+01 | 2.36E+01 | 2.01E+01 | 2.29E+01 | 2.40E+01 | 1.16E+01 | 1.43E+01 | 27.3 |  |  | x |
| 307.1019 | 1.83 | 2.33E+02 | 2.31E+02 | 2.21E+02 | 1.88E+02 | 2.22E+02 | 1.87E+02 | 2.21E+02 | 2.04E+02 | 1.74E+02 | 2.04E+02 | 1.73E+02 | 1.59E+02 | 1.69E+02 | 1.80E+02 | 1.29E+02 | 1.56E+02 | 1.41E+02 | 16.9 |  | x | x |
| 349.1201 | 1.83 | 1.93E+02 | 1.78E+02 | 1.58E+02 | 1.68E+02 | 1.87E+02 | 1.72E+02 | 1.59E+02 | 1.79E+02 | 1.66E+02 | 1.71E+02 | 1.65E+02 | 1.40E+02 | 1.47E+02 | 1.63E+02 | 1.22E+02 | 1.28E+02 | 1.12E+02 | 14.2 | x | x | x |
| 198.1131 | 1.84 | 9.91E+01 | 9.27E+01 | 7.57E+01 | 7.59E+01 | 8.49E+01 | 5.61E+01 | 8.41E+01 | 7.39E+01 | 5.50E+01 | 5.20E+01 | 5.75E+01 | 6.19E+01 | 5.70E+01 | 6.20E+01 | 4.55E+01 | 4.92E+01 | 4.43E+01 | 25.4 |  |  | x |
| 210.0970 | 1.84 | 2.52E+01 | 2.55E+01 | 1.79E+01 | 2.84E+01 | 2.56E+01 | 1.69E+01 | 1.90E+01 | 1.14E+01 | 2.00E+01 | 2.44E+01 | 1.55E+01 | 2.13E+01 | 2.30E+01 | 1.18E+01 | 2.21E+01 | 1.73E+01 | 1.55E+01 | 24.9 |  |  | x |
| 154.1339 | 1.85 | 4.46E+01 | 4.14E+01 | 4.10E+01 | 4.96E+01 | 5.54E+01 | 5.63E+01 | 4.28E+01 | 5.92E+01 | 6.72E+01 | 6.76E+01 | 5.41E+01 | 6.34E+01 | 6.98E+01 | 7.20E+01 | 6.90E+01 | 7.77E+01 | 7.82E+01 | 21.1 |  |  | x |
| 335.0979 | 1.85 | 5.90E+02 | 5.46E+02 | 5.18E+02 | 5.37E+02 | 5.45E+02 | 6.26E+02 | 5.97E+02 | 5.62E+02 | 5.08E+02 | 6.57E+02 | 5.68E+02 | 5.54E+02 | 5.36E+02 | 6.53E+02 | 5.06E+02 | 5.98E+02 | 5.20E+02 | 8.4 | x | x | x |
| 178.0719 | 1.88 | 5.50E+01 | 8.60E+01 | 5.61E+01 | 5.06E+01 | 4.89E+01 | 5.21E+01 | 4.09E+01 | 4.84E+01 | 5.66E+01 | 4.69E+01 | 3.67E+01 | 5.28E+01 | 5.89E+01 | 5.02E+01 | 3.89E+01 | 6.18E+01 | 4.89E+01 | 21.0 |  |  | x |
| 194.1061 | 1.90 | 3.66E+02 | 3.45E+02 | 3.50E+02 | 3.67E+02 | 4.08E+02 | 4.17E+02 | 3.94E+02 | 4.14E+02 | 4.18E+02 | 4.40E+02 | 4.19E+02 | 4.06E+02 | 4.15E+02 | 3.80E+02 | 3.87E+02 | 4.00E+02 | 4.03E+02 | 6.7 | x | x | x |
| 203.1504 | 1.90 | 3.33E+02 | 4.25E+02 | 3.32E+02 | 4.02E+02 | 4.44E+02 | 4.76E+02 | 3.40E+02 | 4.53E+02 | 4.66E+02 | 5.69E+02 | 4.62E+02 | 4.58E+02 | 6.13E+02 | 5.50E+02 | 5.62E+02 | 6.15E+02 | 5.75E+02 | 19.6 |  | x | x |
| 381.0800 | 1.90 | 4.13E+03 | 4.27E+03 | 3.88E+03 | 4.78E+03 | 5.39E+03 | 7.57E+03 | 5.27E+03 | 6.05E+03 | 6.23E+03 | 7.63E+03 | 6.74E+03 | 7.30E+03 | 8.33E+03 | 8.08E+03 | 8.43E+03 | 8.60E+03 | 8.83E+03 | 25.8 |  |  | x |
| 365.1057 | 1.90 | 1.41E+04 | 1.32E+04 | 1.23E+04 | 1.36E+04 | 1.48E+04 | 1.82E+04 | 1.48E+04 | 1.62E+04 | 1.52E+04 | 1.82E+04 | 1.64E+04 | 1.65E+04 | 1.79E+04 | 1.87E+04 | 1.61E+04 | 1.77E+04 | 1.76E+04 | 12.2 | x | x | x |
| 406.1357 | 1.90 | 3.15E+02 | 2.91E+02 | 2.52E+02 | 2.68E+02 | 3.23E+02 | 2.88E+02 | 2.71E+02 | 2.95E+02 | 2.71E+02 | 2.94E+02 | 2.21E+02 | 2.57E+02 | 2.83E+02 | 2.60E+02 | 2.63E+02 | 2.62E+02 | 2.69E+02 | 8.9 | x | x | x |
| 284.1341 | 1.94 | 3.13E+02 | 3.04E+02 | 2.81E+02 | 2.52E+02 | 2.52E+02 | 2.05E+02 | 2.64E+02 | 2.44E+02 | 2.22E+02 | 2.79E+02 | 2.01E+02 | 2.04E+02 | 1.78E+02 | 1.91E+02 | 1.97E+02 | 2.01E+02 | 2.08E+02 | 17.8 |  | x | x |
| 188.0915 | 1.95 | 1.20E+02 | 1.36E+02 | 1.18E+02 | 1.29E+02 | 1.40E+02 | 1.27E+02 | 1.33E+02 | 1.38E+02 | 1.24E+02 | 1.48E+02 | 1.15E+02 | 9.32E+01 | 1.11E+02 | 1.05E+02 | 8.99E+01 | 8.07E+01 | 9.92E+01 | 16.4 |  | x | x |
| 379.1207 | 1.95 | 2.29E+02 | 2.80E+02 | 2.22E+02 | 2.43E+02 | 2.50E+02 | 2.91E+02 | 2.61E+02 | 2.67E+02 | 2.52E+02 | 3.34E+02 | 2.84E+02 | 2.40E+02 | 2.58E+02 | 2.98E+02 | 1.89E+02 | 2.04E+02 | 2.39E+02 | 14.0 | x | x | x |
| 395.1744 | 1.96 | 1.28E+01 | 2.14E+01 | 1.41E+01 | 1.37E+01 | 1.73E+01 | 1.98E+01 | 1.57E+01 | 1.97E+01 | 1.11E+01 | 1.71E+01 | 1.89E+01 | 1.53E+01 | 2.09E+01 | 2.13E+01 | 1.91E+01 | 1.51E+01 | 2.13E+01 | 18.9 |  | x | x |
| 330.0594 | 1.98 | 6.78E+02 | 6.26E+02 | 6.02E+02 | 7.30E+02 | 7.76E+02 | 9.99E+02 | 7.72E+02 | 8.68E+02 | 9.01E+02 | 9.57E+02 | 9.01E+02 | 8.61E+02 | 1.04E+03 | 1.01E+03 | 9.99E+02 | 1.07E+03 | 9.42E+02 | 16.9 |  | x | x |
| 258.1099 | 2.01 | 9.74E+01 | 1.24E+02 | 1.15E+02 | 1.02E+02 | 1.07E+02 | 8.20E+01 | 9.00E+01 | 1.12E+02 | 1.11E+02 | 1.10E+02 | 1.17E+02 | 7.99E+01 | 1.13E+02 | 8.94E+01 | 9.39E+01 | 1.01E+02 | 9.04E+01 | 12.6 | x | x | x |
| 365.1057 | 2.01 | 1.31E+04 | 1.39E+04 | 1.30E+04 | 1.19E+04 | 1.21E+04 | 1.68E+04 | 1.34E+04 | 1.42E+04 | 1.24E+04 | 1.56E+04 | 1.37E+04 | 1.34E+04 | 1.36E+04 | 1.76E+04 | 1.38E+04 | 1.54E+04 | 1.53E+04 | 11.2 | x | x | x |
| 298.1141 | 2.02 | 5.00E+02 | 4.76E+02 | 4.73E+02 | 5.14E+02 | 5.88E+02 | 5.23E+02 | 5.21E+02 | 5.78E+02 | 6.46E+02 | 5.90E+02 | 5.76E+02 | 5.46E+02 | 6.71E+02 | 4.99E+02 | 5.62E+02 | 5.99E+02 | 1.07E+03 | 23.4 |  |  | x |
| 114.0906 | 2.05 | 4.36E+01 | 3.60E+01 | 3.57E+01 | 3.73E+01 | 5.22E+01 | 5.50E+01 | 4.17E+01 | 5.34E+01 | 5.47E+01 | 5.81E+01 | 4.27E+01 | 3.62E+01 | 4.19E+01 | 5.21E+01 | 3.01E+01 | 2.49E+01 | 2.23E+01 | 25.7 |  |  | x |
| 170.0925 | 2.07 | 2.03E+02 | 2.03E+02 | 1.97E+02 | 2.48E+02 | 2.71E+02 | 2.57E+02 | 2.26E+02 | 2.66E+02 | 2.77E+02 | 3.18E+02 | 2.71E+02 | 2.40E+02 | 3.52E+02 | 2.51E+02 | 3.01E+02 | 3.21E+02 | 2.74E+02 | 16.5 |  | x | x |
| 527.1587 | 2.14 | 4.87E+01 | 6.19E+01 | 3.86E+01 | 4.32E+01 | 8.55E+01 | 7.04E+01 | 7.48E+01 | 7.02E+01 | 5.20E+01 | 5.84E+01 | 5.71E+01 | 4.38E+01 | 8.10E+01 | 9.29E+01 | 6.87E+01 | 6.07E+01 | 6.27E+01 | 24.3 |  |  | x |
| 98.9625 | 2.23 | 6.88E+03 | 5.85E+03 | 5.76E+03 | 4.26E+03 | 3.34E+03 | 3.41E+03 | 3.42E+03 | 3.18E+03 | 3.17E+03 | 3.20E+03 | 3.24E+03 | 3.60E+03 | 5.05E+03 | 5.29E+03 | 5.95E+03 | 5.67E+03 | 5.17E+03 | 27.9 |  |  | x |
| 113.9642 | 2.25 | 4.62E+04 | 4.41E+04 | 4.68E+04 | 4.68E+04 | 4.13E+04 | 4.69E+04 | 4.01E+04 | 4.41E+04 | 4.17E+04 | 4.52E+04 | 4.08E+04 | 4.73E+04 | 5.48E+04 | 5.05E+04 | 5.39E+04 | 5.89E+04 | 5.74E+04 | 12.2 | x | x | x |
| 137.9870 | 2.25 | 7.17E+03 | 6.66E+03 | 6.88E+03 | 5.97E+03 | 4.95E+03 | 4.90E+03 | 4.89E+03 | 4.99E+03 | 4.82E+03 | 4.95E+03 | 4.36E+03 | 5.03E+03 | 5.47E+03 | 5.31E+03 | 5.51E+03 | 5.94E+03 | 5.61E+03 | 14.4 | x | x | x |
| 72.9377 | 2.25 | 1.29E+04 | 1.20E+04 | 1.25E+04 | 1.18E+04 | 9.82E+03 | 9.77E+03 | 9.19E+03 | 9.98E+03 | 9.45E+03 | 9.74E+03 | 8.57E+03 | 9.65E+03 | 1.14E+04 | 1.04E+04 | 1.10E+04 | 1.19E+04 | 1.14E+04 | 12.0 | x | x | x |
| 96.9615 | 2.25 | 1.87E+04 | 1.78E+04 | 1.85E+04 | 1.73E+04 | 1.45E+04 | 1.54E+04 | 1.43E+04 | 1.50E+04 | 1.44E+04 | 1.54E+04 | 1.34E+04 | 1.59E+04 | 1.74E+04 | 1.65E+04 | 1.78E+04 | 1.93E+04 | 1.86E+04 | 11.1 | x | x | x |
| 90.9034 | 2.25 | 3.41E+01 | 3.10E+01 | 2.49E+01 | 2.39E+01 | 2.88E+01 | 2.06E+01 | 2.36E+01 | 2.27E+01 | 2.33E+01 | 2.71E+01 | 2.71E+01 | 2.00E+01 | 2.65E+01 | 1.67E+01 | 1.78E+01 | 1.25E+01 | 9.60E+00 | 27.5 |  |  | x |
| 94.9660 | 2.25 | 1.07E+02 | 9.51E+01 | 1.10E+02 | 7.49E+01 | 6.79E+01 | 4.97E+01 | 5.74E+01 | 5.40E+01 | 6.55E+01 | 5.55E+01 | 4.70E+01 | 6.83E+01 | 8.60E+01 | 6.84E+01 | 8.07E+01 | 8.31E+01 | 7.04E+01 | 25.7 |  |  | x |
| 97.9690 | 2.25 | 2.35E+04 | 2.26E+04 | 2.36E+04 | 2.16E+04 | 1.75E+04 | 1.75E+04 | 1.70E+04 | 1.78E+04 | 1.65E+04 | 1.72E+04 | 1.53E+04 | 1.76E+04 | 2.10E+04 | 1.87E+04 | 1.98E+04 | 2.15E+04 | 2.10E+04 | 13.5 | x | x | x |
| 122.9642 | 2.25 | 3.71E+03 | 3.44E+03 | 3.82E+03 | 3.27E+03 | 2.72E+03 | 2.73E+03 | 2.48E+03 | 2.75E+03 | 2.57E+03 | 2.49E+03 | 2.19E+03 | 2.51E+03 | 3.22E+03 | 3.06E+03 | 3.08E+03 | 3.40E+03 | 3.03E+03 | 15.7 |  | x | x |
| 141.9593 | 2.25 | 7.63E+03 | 7.33E+03 | 7.96E+03 | 7.11E+03 | 6.19E+03 | 6.67E+03 | 6.06E+03 | 6.50E+03 | 6.18E+03 | 6.46E+03 | 5.75E+03 | 6.98E+03 | 8.11E+03 | 7.53E+03 | 8.15E+03 | 8.62E+03 | 8.39E+03 | 12.5 | x | x | x |
| 246.8629 | 2.25 | 2.26E+04 | 2.26E+04 | 2.58E+04 | 1.95E+04 | 1.52E+04 | 1.54E+04 | 1.46E+04 | 1.46E+04 | 1.30E+04 | 1.19E+04 | 1.10E+04 | 1.47E+04 | 2.14E+04 | 2.04E+04 | 2.06E+04 | 2.35E+04 | 2.17E+04 | 25.1 |  |  | x |
| 238.8317 | 2.25 | 2.11E+02 | 2.10E+02 | 2.42E+02 | 1.65E+02 | 1.59E+02 | 1.43E+02 | 1.54E+02 | 1.39E+02 | 1.32E+02 | 1.03E+02 | 9.96E+01 | 1.13E+02 | 1.67E+02 | 1.85E+02 | 1.65E+02 | 1.65E+02 | 1.60E+02 | 23.9 |  |  | x |
| 293.8477 | 2.25 | 4.08E+02 | 3.97E+02 | 3.65E+02 | 6.63E+02 | 5.04E+02 | 5.30E+02 | 3.26E+02 | 5.93E+02 | 6.84E+02 | 6.54E+02 | 4.58E+02 | 6.11E+02 | 6.86E+02 | 4.52E+02 | 7.23E+02 | 7.07E+02 | 7.03E+02 | 24.2 |  |  | x |
| 287.8835 | 2.25 | 8.91E+02 | 8.63E+02 | 9.80E+02 | 6.58E+02 | 5.87E+02 | 6.32E+02 | 5.83E+02 | 5.79E+02 | 5.33E+02 | 4.83E+02 | 4.38E+02 | 5.77E+02 | 7.64E+02 | 8.19E+02 | 8.09E+02 | 8.26E+02 | 7.83E+02 | 22.8 |  |  | x |
| 318.7928 | 2.25 | 1.73E+03 | 1.75E+03 | 2.11E+03 | 1.49E+03 | 1.22E+03 | 1.05E+03 | 1.17E+03 | 1.15E+03 | 1.02E+03 | 8.00E+02 | 6.93E+02 | 1.06E+03 | 1.44E+03 | 1.36E+03 | 1.33E+03 | 1.53E+03 | 1.32E+03 | 27.1 |  |  | x |
| 87.0046 | 2.26 | 7.35E+01 | 7.12E+01 | 8.83E+01 | 6.31E+01 | 5.71E+01 | 3.55E+01 | 4.60E+01 | 6.14E+01 | 6.75E+01 | 4.52E+01 | 4.28E+01 | 5.16E+01 | 5.73E+01 | 4.43E+01 | 4.87E+01 | 4.50E+01 | 3.66E+01 | 26.1 |  |  | x |
| 138.9726 | 2.29 | 8.51E+01 | 8.35E+01 | 8.11E+01 | 6.75E+01 | 6.06E+01 | 4.52E+01 | 6.71E+01 | 6.07E+01 | 6.23E+01 | 6.39E+01 | 5.04E+01 | 5.22E+01 | 5.96E+01 | 3.71E+01 | 5.42E+01 | 5.39E+01 | 3.99E+01 | 23.2 |  |  | x |
| 161.9791 | 2.29 | 2.17E+02 | 1.80E+02 | 1.66E+02 | 1.38E+02 | 1.01E+02 | 1.15E+02 | 9.09E+01 | 1.03E+02 | 1.16E+02 | 1.12E+02 | 9.58E+01 | 1.11E+02 | 1.53E+02 | 1.57E+02 | 1.69E+02 | 1.74E+02 | 1.21E+02 | 26.4 |  |  | x |
| 234.9285 | 2.29 | 1.04E+02 | 1.00E+02 | 1.09E+02 | 1.12E+02 | 1.18E+02 | 1.07E+02 | 1.12E+02 | 1.26E+02 | 1.23E+02 | 1.20E+02 | 9.30E+01 | 1.33E+02 | 5.92E+01 | 6.01E+01 | 1.09E+02 | 4.94E+01 | 4.75E+01 | 27.8 |  |  | x |
| 182.9629 | 2.30 | 2.23E+02 | 1.95E+02 | 1.90E+02 | 1.66E+02 | 1.23E+02 | 1.05E+02 | 1.18E+02 | 1.31E+02 | 1.25E+02 | 1.22E+02 | 1.17E+02 | 1.11E+02 | 1.18E+02 | 1.15E+02 | 1.05E+02 | 1.18E+02 | 9.47E+01 | 27.2 |  |  | x |
| 254.8575 | 2.32 | 4.27E+03 | 3.83E+03 | 4.10E+03 | 2.94E+03 | 2.68E+03 | 3.23E+03 | 3.00E+03 | 2.65E+03 | 2.26E+03 | 2.32E+03 | 2.12E+03 | 2.25E+03 | 2.03E+03 | 2.96E+03 | 2.37E+03 | 2.57E+03 | 2.81E+03 | 23.7 |  |  | x |
| 107.9597 | 2.33 | 2.68E+02 | 2.29E+02 | 2.33E+02 | 2.35E+02 | 2.56E+02 | 3.35E+02 | 2.66E+02 | 2.68E+02 | 2.47E+02 | 2.68E+02 | 2.51E+02 | 2.48E+02 | 2.69E+02 | 2.57E+02 | 2.51E+02 | 2.60E+02 | 2.42E+02 | 9.2 | x | x | x |
| 125.9548 | 2.33 | 8.03E+01 | 6.95E+01 | 7.60E+01 | 7.47E+01 | 8.66E+01 | 1.11E+02 | 8.26E+01 | 9.07E+01 | 9.19E+01 | 9.18E+01 | 8.08E+01 | 8.82E+01 | 1.01E+02 | 8.76E+01 | 9.77E+01 | 9.39E+01 | 8.53E+01 | 11.7 | x | x | x |
| 130.9585 | 2.33 | 2.91E+03 | 2.41E+03 | 2.42E+03 | 2.67E+03 | 2.83E+03 | 4.01E+03 | 3.01E+03 | 3.13E+03 | 2.99E+03 | 3.48E+03 | 3.16E+03 | 3.19E+03 | 3.18E+03 | 3.47E+03 | 3.20E+03 | 3.29E+03 | 3.12E+03 | 12.6 | x | x | x |
| 132.9553 | 2.33 | 2.36E+03 | 2.00E+03 | 2.07E+03 | 2.11E+03 | 2.22E+03 | 3.02E+03 | 2.33E+03 | 2.44E+03 | 2.36E+03 | 2.63E+03 | 2.33E+03 | 2.47E+03 | 2.42E+03 | 2.65E+03 | 2.44E+03 | 2.47E+03 | 2.44E+03 | 10.0 | x | x | x |
| 134.9541 | 2.33 | 7.46E+02 | 6.19E+02 | 6.36E+02 | 7.16E+02 | 7.29E+02 | 9.97E+02 | 7.95E+02 | 8.36E+02 | 7.92E+02 | 8.73E+02 | 8.16E+02 | 8.58E+02 | 9.47E+02 | 8.33E+02 | 9.02E+02 | 9.41E+02 | 7.70E+02 | 12.9 | x | x | x |
| 151.9500 | 2.33 | 4.90E+02 | 4.28E+02 | 4.16E+02 | 4.31E+02 | 4.58E+02 | 5.62E+02 | 4.79E+02 | 5.14E+02 | 4.90E+02 | 5.49E+02 | 4.44E+02 | 4.82E+02 | 5.18E+02 | 4.71E+02 | 4.61E+02 | 4.84E+02 | 3.66E+02 | 10.3 | x | x | x |
| 205.9202 | 2.33 | 6.28E+02 | 5.83E+02 | 6.86E+02 | 6.68E+02 | 6.72E+02 | 7.83E+02 | 7.29E+02 | 7.80E+02 | 7.52E+02 | 7.72E+02 | 7.14E+02 | 7.99E+02 | 5.98E+02 | 5.46E+02 | 7.42E+02 | 6.64E+02 | 5.60E+02 | 11.9 | x | x | x |
| 256.8544 | 2.33 | 2.09E+03 | 1.82E+03 | 1.93E+03 | 1.34E+03 | 1.26E+03 | 1.49E+03 | 1.43E+03 | 1.22E+03 | 1.02E+03 | 1.07E+03 | 9.70E+02 | 9.99E+02 | 9.02E+02 | 1.29E+03 | 1.05E+03 | 1.16E+03 | 1.19E+03 | 26.6 |  |  | x |
| 258.8630 | 2.33 | 1.70E+03 | 1.50E+03 | 1.60E+03 | 1.19E+03 | 1.07E+03 | 1.09E+03 | 1.07E+03 | 9.71E+02 | 9.45E+02 | 1.00E+03 | 8.28E+02 | 9.06E+02 | 6.40E+02 | 9.37E+02 | 8.26E+02 | 8.45E+02 | 8.05E+02 | 27.8 |  |  | x |
| 266.8503 | 2.33 | 2.77E+02 | 2.20E+02 | 2.52E+02 | 1.78E+02 | 2.16E+02 | 3.13E+02 | 2.75E+02 | 2.34E+02 | 2.00E+02 | 2.15E+02 | 2.01E+02 | 1.86E+02 | 1.25E+02 | 1.96E+02 | 1.30E+02 | 1.27E+02 | 1.05E+02 | 28.7 |  |  | x |
| 295.8812 | 2.33 | 5.91E+02 | 4.94E+02 | 5.34E+02 | 3.98E+02 | 3.87E+02 | 6.11E+02 | 4.98E+02 | 4.28E+02 | 3.92E+02 | 3.98E+02 | 3.47E+02 | 3.67E+02 | 3.18E+02 | 4.60E+02 | 3.67E+02 | 3.98E+02 | 4.10E+02 | 19.3 |  | x | x |
| 264.8487 | 2.33 | 4.99E+02 | 4.04E+02 | 4.36E+02 | 3.10E+02 | 3.29E+02 | 5.04E+02 | 4.23E+02 | 3.42E+02 | 3.13E+02 | 3.30E+02 | 2.93E+02 | 2.88E+02 | 1.90E+02 | 3.10E+02 | 2.16E+02 | 2.15E+02 | 2.20E+02 | 28.9 |  |  | x |
| 303.8801 | 2.33 | 1.59E+02 | 1.31E+02 | 1.32E+02 | 9.51E+01 | 1.18E+02 | 2.00E+02 | 1.57E+02 | 1.24E+02 | 1.16E+02 | 1.22E+02 | 1.07E+02 | 1.03E+02 | 8.03E+01 | 1.30E+02 | 9.01E+01 | 9.28E+01 | 1.06E+02 | 24.4 |  |  | x |
| 108.9609 | 2.38 | 1.52E+02 | 1.22E+02 | 1.35E+02 | 1.06E+02 | 9.53E+01 | 1.02E+02 | 8.50E+01 | 9.27E+01 | 8.80E+01 | 8.60E+01 | 8.09E+01 | 8.35E+01 | 1.09E+02 | 9.71E+01 | 1.07E+02 | 1.05E+02 | 9.88E+01 | 18.5 |  | x | x |
| 84.9600 | 2.40 | 1.31E+03 | 1.31E+03 | 1.47E+03 | 1.34E+03 | 1.41E+03 | 1.67E+03 | 1.54E+03 | 1.51E+03 | 1.48E+03 | 1.65E+03 | 1.52E+03 | 1.61E+03 | 1.70E+03 | 1.72E+03 | 1.61E+03 | 1.67E+03 | 1.71E+03 | 9.2 | x | x | x |
| 230.8901 | 2.40 | 2.03E+03 | 1.99E+03 | 2.33E+03 | 1.65E+03 | 1.66E+03 | 1.73E+03 | 1.86E+03 | 1.61E+03 | 1.41E+03 | 1.55E+03 | 1.40E+03 | 1.43E+03 | 1.28E+03 | 1.88E+03 | 1.36E+03 | 1.39E+03 | 1.56E+03 | 17.3 |  | x | x |
| 242.8853 | 2.40 | 7.99E+01 | 6.88E+01 | 8.21E+01 | 7.45E+01 | 8.45E+01 | 1.02E+02 | 7.81E+01 | 9.76E+01 | 7.84E+01 | 7.94E+01 | 6.71E+01 | 8.59E+01 | 5.06E+01 | 6.35E+01 | 5.40E+01 | 5.35E+01 | 5.17E+01 | 20.9 |  |  | x |
| 125.9834 | 2.41 | 1.78E+02 | 1.69E+02 | 1.60E+02 | 1.65E+02 | 1.80E+02 | 2.02E+02 | 1.81E+02 | 1.86E+02 | 1.83E+02 | 1.82E+02 | 1.85E+02 | 1.92E+02 | 2.36E+02 | 1.89E+02 | 1.99E+02 | 2.02E+02 | 1.89E+02 | 9.2 | x | x | x |
| 277.8714 | 2.42 | 5.14E+01 | 4.47E+01 | 4.77E+01 | 2.77E+01 | 3.21E+01 | 4.48E+01 | 3.68E+01 | 4.27E+01 | 4.06E+01 | 2.81E+01 | 2.78E+01 | 3.53E+01 | 3.64E+01 | 4.13E+01 | 4.61E+01 | 4.28E+01 | 6.45E+01 | 23.3 |  |  | x |
| 108.9614 | 2.64 | 1.13E+03 | 9.80E+02 | 9.01E+02 | 8.73E+02 | 6.84E+02 | 7.23E+02 | 7.18E+02 | 7.07E+02 | 7.28E+02 | 7.61E+02 | 6.79E+02 | 7.39E+02 | 7.91E+02 | 7.64E+02 | 7.59E+02 | 8.08E+02 | 6.78E+02 | 15.2 |  | x | x |
| 206.9172 | 2.72 | 1.33E+03 | 1.24E+03 | 1.31E+03 | 1.12E+03 | 1.09E+03 | 1.18E+03 | 1.31E+03 | 1.21E+03 | 1.09E+03 | 1.21E+03 | 1.03E+03 | 1.04E+03 | 4.83E+02 | 9.88E+02 | 7.55E+02 | 6.41E+02 | 8.34E+02 | 23.2 |  |  | x |
| 130.0080 | 2.74 | 1.43E+03 | 1.35E+03 | 1.41E+03 | 1.77E+03 | 2.02E+03 | 2.98E+03 | 2.54E+03 | 2.62E+03 | 2.57E+03 | 2.86E+03 | 2.86E+03 | 3.18E+03 | 3.27E+03 | 2.20E+03 | 3.54E+03 | 3.28E+03 | 2.61E+03 | 27.9 |  |  | x |
| 60.9869 | 2.75 | 2.76E+02 | 2.63E+02 | 2.69E+02 | 2.93E+02 | 3.34E+02 | 4.22E+02 | 4.01E+02 | 4.27E+02 | 4.25E+02 | 4.44E+02 | 4.17E+02 | 3.89E+02 | 1.93E+02 | 2.96E+02 | 3.46E+02 | 1.78E+02 | 2.30E+02 | 26.5 |  |  | x |
| 116.9766 | 2.75 | 3.03E+03 | 2.86E+03 | 3.05E+03 | 3.40E+03 | 3.98E+03 | 5.49E+03 | 4.87E+03 | 4.89E+03 | 4.69E+03 | 5.87E+03 | 4.90E+03 | 4.48E+03 | 2.07E+03 | 4.40E+03 | 3.47E+03 | 2.37E+03 | 3.90E+03 | 27.4 |  |  | x |
| 550.6285 | 2.96 | 2.55E+03 | 2.45E+03 | 2.55E+03 | 2.49E+03 | 2.57E+03 | 2.79E+03 | 2.69E+03 | 2.69E+03 | 2.52E+03 | 2.79E+03 | 2.67E+03 | 2.84E+03 | 2.90E+03 | 2.57E+03 | 2.75E+03 | 2.89E+03 | 2.87E+03 | 5.5 | x | x | x |
| 100.0759 | 3.01 | 5.08E+02 | 4.35E+02 | 4.80E+02 | 4.48E+02 | 4.19E+02 | 3.79E+02 | 4.45E+02 | 4.28E+02 | 3.95E+02 | 4.06E+02 | 3.89E+02 | 4.02E+02 | 3.48E+02 | 3.49E+02 | 3.52E+02 | 3.49E+02 | 3.63E+02 | 11.8 | x | x | x |
| 338.3411 | 3.01 | 3.25E+03 | 3.02E+03 | 3.30E+03 | 3.57E+03 | 3.35E+03 | 2.59E+03 | 3.41E+03 | 3.63E+03 | 3.02E+03 | 3.04E+03 | 3.13E+03 | 3.05E+03 | 2.65E+03 | 2.20E+03 | 2.48E+03 | 2.29E+03 | 2.29E+03 | 15.5 |  | x | x |
| 270.2789 | 3.14 | 4.67E+02 | 4.66E+02 | 5.64E+02 | 3.91E+02 | 3.83E+02 | 3.62E+02 | 3.80E+02 | 3.82E+02 | 3.71E+02 | 3.68E+02 | 3.59E+02 | 3.39E+02 | 3.67E+02 | 3.07E+02 | 3.24E+02 | 3.50E+02 | 3.38E+02 | 16.3 |  | x | x |
| 114.0915 | 3.17 | 4.15E+02 | 3.83E+02 | 3.89E+02 | 4.20E+02 | 4.37E+02 | 4.99E+02 | 4.77E+02 | 4.77E+02 | 4.83E+02 | 5.25E+02 | 4.82E+02 | 5.00E+02 | 4.60E+02 | 4.40E+02 | 4.73E+02 | 4.83E+02 | 4.73E+02 | 8.6 | x | x | x |
| 171.1423 | 3.19 | 2.73E+02 | 2.40E+02 | 2.45E+02 | 2.43E+02 | 2.48E+02 | 1.89E+02 | 2.42E+02 | 2.58E+02 | 2.59E+02 | 2.07E+02 | 2.20E+02 | 1.90E+02 | 1.70E+02 | 1.44E+02 | 1.65E+02 | 1.09E+02 | 1.22E+02 | 24.5 |  |  | x |
| 62.9291 | 3.25 | 9.83E+01 | 1.04E+02 | 9.75E+01 | 1.05E+02 | 9.84E+01 | 1.13E+02 | 9.04E+01 | 1.08E+02 | 1.05E+02 | 1.00E+02 | 1.05E+02 | 1.07E+02 | 1.12E+02 | 9.25E+01 | 1.18E+02 | 1.02E+02 | 8.06E+01 | 8.8 | x | x | x |
| 64.9273 | 3.25 | 1.58E+01 | 1.42E+01 | 1.59E+01 | 1.12E+01 | 1.20E+01 | 8.62E+00 | 1.26E+01 | 1.16E+01 | 1.57E+01 | 6.10E+00 | 1.15E+01 | 1.10E+01 | 1.28E+01 | 5.68E+00 | 1.10E+01 | 7.14E+00 | 1.10E+01 | 27.4 |  |  | x |
| 103.9559 | 3.25 | 5.29E+04 | 5.12E+04 | 5.23E+04 | 5.90E+04 | 5.91E+04 | 7.96E+04 | 6.30E+04 | 6.61E+04 | 6.42E+04 | 7.53E+04 | 7.16E+04 | 7.96E+04 | 8.39E+04 | 8.06E+04 | 8.43E+04 | 9.02E+04 | 9.04E+04 | 18.8 |  | x | x |
| 124.0873 | 3.25 | 1.38E+04 | 1.29E+04 | 1.31E+04 | 1.54E+04 | 1.56E+04 | 2.13E+04 | 1.68E+04 | 1.78E+04 | 1.72E+04 | 2.01E+04 | 1.93E+04 | 2.07E+04 | 2.18E+04 | 2.12E+04 | 2.23E+04 | 2.30E+04 | 2.29E+04 | 18.9 |  | x | x |
| 138.1024 | 3.25 | 1.76E+02 | 1.73E+02 | 1.88E+02 | 2.00E+02 | 2.01E+02 | 2.38E+02 | 2.15E+02 | 2.10E+02 | 2.22E+02 | 2.11E+02 | 2.22E+02 | 1.86E+02 | 1.72E+02 | 1.89E+02 | 1.99E+02 | 1.94E+02 | 1.82E+02 | 9.6 | x | x | x |
| 144.9825 | 3.25 | 2.81E+04 | 2.69E+04 | 2.74E+04 | 3.02E+04 | 3.03E+04 | 4.05E+04 | 3.24E+04 | 3.37E+04 | 3.29E+04 | 3.82E+04 | 3.63E+04 | 4.03E+04 | 4.14E+04 | 4.02E+04 | 4.19E+04 | 4.46E+04 | 4.48E+04 | 16.9 |  | x | x |
| 146.9807 | 3.25 | 1.04E+04 | 1.00E+04 | 1.01E+04 | 1.11E+04 | 1.12E+04 | 1.46E+04 | 1.19E+04 | 1.24E+04 | 1.21E+04 | 1.38E+04 | 1.32E+04 | 1.45E+04 | 1.48E+04 | 1.44E+04 | 1.51E+04 | 1.60E+04 | 1.60E+04 | 15.6 |  | x | x |
| 173.0794 | 3.25 | 1.94E+02 | 1.73E+02 | 1.71E+02 | 1.69E+02 | 1.65E+02 | 1.76E+02 | 1.94E+02 | 1.96E+02 | 1.77E+02 | 1.68E+02 | 1.81E+02 | 1.38E+02 | 1.05E+02 | 1.33E+02 | 1.62E+02 | 1.06E+02 | 7.91E+01 | 21.7 |  |  | x |
| 186.0087 | 3.25 | 1.06E+02 | 9.76E+01 | 1.06E+02 | 1.13E+02 | 9.56E+01 | 8.69E+01 | 1.04E+02 | 9.05E+01 | 1.09E+02 | 9.20E+01 | 9.26E+01 | 7.42E+01 | 7.77E+01 | 6.48E+01 | 8.96E+01 | 6.76E+01 | 5.87E+01 | 18.1 |  | x | x |
| 239.0897 | 3.25 | 2.74E+02 | 2.34E+02 | 2.53E+02 | 2.42E+02 | 2.64E+02 | 2.31E+02 | 2.93E+02 | 2.53E+02 | 2.46E+02 | 2.63E+02 | 2.24E+02 | 2.32E+02 | 1.37E+02 | 2.04E+02 | 1.77E+02 | 1.30E+02 | 1.40E+02 | 22.1 |  |  | x |
| 335.1253 | 3.25 | 6.44E+02 | 6.44E+02 | 7.44E+02 | 6.19E+02 | 6.29E+02 | 7.61E+02 | 7.38E+02 | 7.01E+02 | 6.53E+02 | 7.41E+02 | 6.90E+02 | 7.33E+02 | 6.46E+02 | 7.34E+02 | 6.40E+02 | 6.56E+02 | 6.50E+02 | 7.1 | x | x | x |
| 413.2651 | 3.25 | 1.33E+03 | 1.32E+03 | 1.45E+03 | 1.34E+03 | 1.26E+03 | 1.15E+03 | 1.40E+03 | 1.29E+03 | 1.17E+03 | 1.16E+03 | 1.17E+03 | 1.13E+03 | 8.51E+02 | 9.79E+02 | 8.57E+02 | 8.26E+02 | 8.70E+02 | 17.8 |  | x | x |
| 429.2403 | 3.25 | 1.45E+02 | 1.34E+02 | 1.23E+02 | 1.46E+02 | 1.36E+02 | 1.28E+02 | 1.45E+02 | 1.34E+02 | 1.31E+02 | 1.29E+02 | 1.23E+02 | 1.21E+02 | 1.03E+02 | 7.90E+01 | 1.19E+02 | 9.09E+01 | 5.81E+01 | 20.3 |  |  | x |
|  |  |  |  |  |  |  |  |  |  |  |  |  |  |  |  |  |  |  | **Total ions** | 429 | 575 | 750 |
|  |  |  |  |  |  |  |  |  |  |  |  |  |  |  |  |  |  |  | **% of ions** | 57 | 77 | - |

**Table S3.** List of features detected with %CV of abundance across QC sample injections during the Rapid HILIC IMS MS analysis.

|  |  | Normalised abundance | | | | | | | | | | | | | | | | |  |  |  |  |
| --- | --- | --- | --- | --- | --- | --- | --- | --- | --- | --- | --- | --- | --- | --- | --- | --- | --- | --- | --- | --- | --- | --- |
| m/z | RT (min) | QC 1 | QC 2 | QC 3 | QC 4 | QC 5 | QC 6 | QC 7 | QC 8 | QC 9 | QC 10 | QC 11 | QC 12 | QC 13 | QC 14 | QC 15 | QC 16 | QC 17 | CV% | CV < 15 | CV < 20 | CV <= 30 |
| 178.1583 | 0.15 | 9.81E+00 | 7.35E+00 | 1.16E+01 | 1.38E+01 | 8.56E+00 | 1.13E+01 | 1.25E+01 | 1.24E+01 | 1.37E+01 | 1.00E+01 | 1.20E+01 | 9.65E+00 | 1.11E+01 | 1.11E+01 | 9.43E+00 | 1.21E+01 | 1.06E+01 | 15.73 |  | x | x |
| 184.0179 | 0.15 | 4.59E+01 | 3.29E+01 | 3.73E+01 | 3.73E+01 | 3.55E+01 | 3.69E+01 | 3.44E+01 | 3.56E+01 | 3.76E+01 | 3.46E+01 | 5.44E+01 | 4.70E+01 | 3.00E+01 | 3.08E+01 | 3.01E+01 | 3.41E+01 | 3.23E+01 | 17.66 |  | x | x |
| 186.0148 | 0.15 | 3.26E+01 | 2.06E+01 | 1.84E+01 | 2.90E+01 | 2.57E+01 | 2.14E+01 | 1.96E+01 | 2.02E+01 | 2.27E+01 | 1.80E+01 | 3.15E+01 | 3.02E+01 | 1.88E+01 | 2.17E+01 | 1.81E+01 | 1.68E+01 | 1.93E+01 | 22.76 |  |  | x |
| 226.8584 | 0.17 | 2.00E+01 | 1.81E+01 | 1.98E+01 | 2.47E+01 | 1.88E+01 | 2.22E+01 | 1.85E+01 | 2.18E+01 | 1.65E+01 | 1.67E+01 | 2.41E+01 | 6.93E+00 | 1.77E+01 | 1.49E+01 | 1.57E+01 | 1.47E+01 | 1.92E+01 | 22.68 |  |  | x |
| 266.9994 | 0.17 | 1.59E+01 | 1.32E+01 | 1.39E+01 | 1.27E+01 | 1.19E+01 | 1.04E+01 | 9.86E+00 | 1.01E+01 | 1.40E+01 | 1.14E+01 | 1.62E+01 | 5.30E+00 | 1.09E+01 | 9.08E+00 | 1.34E+01 | 1.12E+01 | 1.33E+01 | 22.26 |  |  | x |
| 281.0513 | 0.17 | 2.92E+02 | 2.48E+02 | 2.81E+02 | 2.82E+02 | 2.81E+02 | 3.00E+02 | 2.70E+02 | 2.74E+02 | 3.01E+02 | 2.64E+02 | 4.24E+02 | 2.07E+02 | 2.75E+02 | 2.72E+02 | 2.60E+02 | 2.65E+02 | 2.72E+02 | 15.24 |  | x | x |
| 341.0176 | 0.17 | 2.35E+01 | 1.82E+01 | 1.92E+01 | 1.63E+01 | 1.56E+01 | 1.43E+01 | 9.40E+00 | 1.20E+01 | 1.65E+01 | 1.41E+01 | 1.50E+01 | 6.26E+00 | 1.49E+01 | 1.17E+01 | 1.25E+01 | 1.28E+01 | 1.13E+01 | 27.63 |  |  | x |
| 536.1665 | 0.17 | 7.23E+02 | 7.02E+02 | 6.93E+02 | 5.77E+02 | 5.96E+02 | 5.61E+02 | 5.10E+02 | 5.16E+02 | 5.17E+02 | 4.00E+02 | 6.90E+02 | 3.46E+02 | 4.00E+02 | 3.99E+02 | 3.68E+02 | 3.79E+02 | 3.66E+02 | 25.89 |  |  | x |
| 610.1853 | 0.17 | 1.82E+03 | 1.69E+03 | 1.59E+03 | 1.31E+03 | 1.29E+03 | 1.24E+03 | 1.17E+03 | 1.11E+03 | 1.21E+03 | 9.91E+02 | 1.76E+03 | 8.46E+02 | 1.00E+03 | 1.02E+03 | 9.10E+02 | 1.02E+03 | 9.53E+02 | 25.07 |  |  | x |
| 148.1112 | 0.18 | 8.52E+01 | 8.00E+01 | 8.26E+01 | 8.79E+01 | 8.50E+01 | 1.01E+02 | 9.53E+01 | 9.49E+01 | 9.51E+01 | 8.87E+01 | 1.39E+02 | 9.29E+01 | 9.25E+01 | 9.47E+01 | 8.84E+01 | 9.35E+01 | 9.15E+01 | 13.87 | x | x | x |
| 149.0255 | 0.18 | 4.17E+00 | 4.21E+00 | 5.22E+00 | 4.51E+00 | 4.63E+00 | 4.97E+00 | 6.15E+00 | 4.81E+00 | 7.28E+00 | 5.98E+00 | 8.87E+00 | 5.78E+00 | 6.36E+00 | 6.52E+00 | 6.01E+00 | 7.25E+00 | 6.66E+00 | 21.67 |  |  | x |
| 209.0202 | 0.18 | 1.54E+01 | 1.26E+01 | 1.31E+01 | 1.53E+01 | 1.36E+01 | 1.89E+01 | 1.25E+01 | 1.19E+01 | 1.47E+01 | 1.24E+01 | 1.70E+01 | 8.12E+00 | 1.42E+01 | 1.48E+01 | 1.13E+01 | 8.66E+00 | 1.04E+01 | 20.92 |  |  | x |
| 213.1459 | 0.18 | 9.82E+00 | 1.14E+01 | 1.20E+01 | 1.11E+01 | 1.45E+01 | 1.67E+01 | 1.48E+01 | 1.65E+01 | 1.56E+01 | 1.39E+01 | 2.78E+01 | 1.23E+01 | 1.53E+01 | 1.76E+01 | 1.48E+01 | 1.58E+01 | 1.94E+01 | 26.89 |  |  | x |
| 223.0637 | 0.18 | 5.10E+01 | 4.49E+01 | 4.83E+01 | 4.74E+01 | 5.13E+01 | 5.39E+01 | 5.09E+01 | 4.92E+01 | 5.43E+01 | 5.42E+01 | 8.17E+01 | 4.47E+01 | 5.29E+01 | 5.37E+01 | 4.66E+01 | 5.42E+01 | 5.02E+01 | 15.75 |  | x | x |
| 227.1246 | 0.18 | 1.95E+01 | 1.82E+01 | 1.75E+01 | 2.05E+01 | 2.35E+01 | 2.74E+01 | 2.58E+01 | 2.40E+01 | 3.02E+01 | 2.48E+01 | 4.40E+01 | 2.06E+01 | 2.74E+01 | 3.26E+01 | 2.51E+01 | 2.99E+01 | 2.99E+01 | 24.95 |  |  | x |
| 207.0318 | 0.18 | 1.06E+02 | 8.68E+01 | 9.85E+01 | 9.58E+01 | 1.06E+02 | 1.02E+02 | 9.57E+01 | 9.20E+01 | 1.11E+02 | 9.64E+01 | 1.53E+02 | 7.73E+01 | 1.01E+02 | 1.08E+02 | 9.76E+01 | 9.89E+01 | 9.93E+01 | 15.21 |  | x | x |
| 271.1853 | 0.18 | 8.84E+00 | 8.75E+00 | 1.01E+01 | 7.65E+00 | 1.04E+01 | 1.02E+01 | 1.12E+01 | 9.69E+00 | 1.13E+01 | 9.81E+00 | 1.61E+01 | 9.62E+00 | 1.34E+01 | 1.11E+01 | 1.13E+01 | 1.34E+01 | 1.07E+01 | 18.74 |  | x | x |
| 281.1946 | 0.18 | 3.10E+00 | 1.98E+00 | 1.94E+00 | 1.88E+00 | 1.58E+00 | 2.31E+00 | 1.72E+00 | 2.24E+00 | 1.47E+00 | 1.73E+00 | 2.93E+00 | 5.46E-01 | 2.00E+00 | 2.09E+00 | 2.31E+00 | 1.68E+00 | 2.03E+00 | 28.81 |  |  | x |
| 299.1103 | 0.18 | 3.71E+00 | 3.91E+00 | 3.95E+00 | 5.80E+00 | 4.99E+00 | 6.17E+00 | 6.03E+00 | 3.58E+00 | 5.82E+00 | 6.43E+00 | 6.47E+00 | 3.87E+00 | 7.12E+00 | 7.24E+00 | 4.22E+00 | 7.39E+00 | 7.35E+00 | 25.46 |  |  | x |
| 355.0697 | 0.18 | 3.96E+02 | 3.38E+02 | 3.64E+02 | 3.41E+02 | 3.21E+02 | 3.32E+02 | 2.99E+02 | 2.87E+02 | 2.98E+02 | 2.97E+02 | 4.84E+02 | 2.31E+02 | 3.09E+02 | 2.84E+02 | 2.70E+02 | 2.97E+02 | 2.94E+02 | 17.69 |  | x | x |
| 297.0824 | 0.18 | 2.48E+01 | 1.82E+01 | 2.19E+01 | 2.40E+01 | 2.27E+01 | 2.51E+01 | 2.15E+01 | 1.95E+01 | 2.50E+01 | 2.05E+01 | 3.43E+01 | 1.90E+01 | 2.48E+01 | 2.67E+01 | 2.31E+01 | 2.13E+01 | 2.35E+01 | 15.95 |  | x | x |
| 337.1043 | 0.18 | 1.02E+01 | 7.58E+00 | 1.03E+01 | 1.02E+01 | 9.62E+00 | 1.24E+01 | 9.86E+00 | 1.07E+01 | 1.10E+01 | 1.14E+01 | 1.43E+01 | 5.17E+00 | 1.07E+01 | 9.98E+00 | 7.60E+00 | 8.33E+00 | 1.06E+01 | 20.38 |  |  | x |
| 411.0942 | 0.18 | 1.99E+01 | 2.18E+01 | 2.12E+01 | 1.92E+01 | 2.37E+01 | 1.82E+01 | 2.23E+01 | 3.15E+01 | 1.52E+01 | 1.92E+01 | 2.14E+01 | 1.48E+01 | 1.76E+01 | 2.67E+01 | 1.67E+01 | 1.82E+01 | 2.01E+01 | 20.22 |  |  | x |
| 371.1015 | 0.18 | 3.27E+02 | 2.71E+02 | 3.04E+02 | 2.81E+02 | 2.69E+02 | 2.83E+02 | 2.61E+02 | 2.46E+02 | 2.66E+02 | 2.66E+02 | 4.19E+02 | 2.13E+02 | 2.52E+02 | 2.60E+02 | 2.41E+02 | 2.54E+02 | 2.64E+02 | 16.28 |  | x | x |
| 445.1202 | 0.18 | 1.22E+02 | 1.24E+02 | 1.40E+02 | 1.25E+02 | 1.10E+02 | 1.09E+02 | 1.04E+02 | 1.31E+02 | 1.09E+02 | 8.65E+01 | 1.39E+02 | 7.99E+01 | 8.93E+01 | 9.83E+01 | 8.59E+01 | 9.95E+01 | 1.08E+02 | 16.95 |  | x | x |
| 637.3056 | 0.18 | 1.32E+02 | 1.61E+02 | 1.47E+02 | 1.25E+02 | 1.14E+02 | 1.20E+02 | 9.99E+01 | 9.84E+01 | 1.21E+02 | 8.84E+01 | 1.38E+02 | 6.51E+01 | 1.03E+02 | 9.42E+01 | 7.44E+01 | 1.06E+02 | 8.32E+01 | 23.53 |  |  | x |
| 136.0215 | 0.19 | 4.36E+00 | 6.10E+00 | 6.77E+00 | 6.41E+00 | 8.20E+00 | 7.22E+00 | 5.56E+00 | 1.00E+01 | 8.50E+00 | 6.44E+00 | 9.23E+00 | 3.53E+00 | 4.06E+00 | 6.90E+00 | 7.75E+00 | 7.40E+00 | 8.31E+00 | 26.04 |  |  | x |
| 158.1537 | 0.19 | 1.23E+01 | 1.23E+01 | 1.21E+01 | 1.36E+01 | 1.36E+01 | 1.27E+01 | 1.67E+01 | 1.51E+01 | 1.82E+01 | 1.37E+01 | 2.45E+01 | 1.22E+01 | 1.33E+01 | 1.47E+01 | 1.56E+01 | 1.39E+01 | 1.45E+01 | 20.86 |  |  | x |
| 167.1541 | 0.19 | 3.19E+00 | 4.40E+00 | 3.99E+00 | 2.60E+00 | 3.29E+00 | 5.54E+00 | 5.54E+00 | 5.58E+00 | 4.61E+00 | 3.40E+00 | 8.42E+00 | 4.67E+00 | 4.84E+00 | 3.90E+00 | 4.13E+00 | 4.82E+00 | 3.79E+00 | 29.43 |  |  | x |
| 213.1095 | 0.19 | 2.78E+01 | 2.77E+01 | 2.90E+01 | 2.61E+01 | 2.67E+01 | 3.03E+01 | 3.01E+01 | 2.41E+01 | 2.98E+01 | 2.92E+01 | 5.12E+01 | 3.22E+01 | 2.87E+01 | 3.33E+01 | 3.25E+01 | 2.86E+01 | 3.07E+01 | 19.18 |  | x | x |
| 243.1555 | 0.19 | 4.36E+00 | 3.45E+00 | 3.86E+00 | 2.55E+00 | 3.41E+00 | 4.25E+00 | 2.15E+00 | 3.32E+00 | 3.24E+00 | 4.00E+00 | 6.44E+00 | 2.26E+00 | 4.24E+00 | 4.01E+00 | 3.60E+00 | 3.72E+00 | 4.48E+00 | 26.53 |  |  | x |
| 229.1425 | 0.19 | 7.89E+02 | 7.52E+02 | 8.22E+02 | 8.69E+02 | 9.58E+02 | 1.01E+03 | 9.95E+02 | 9.24E+02 | 1.04E+03 | 9.16E+02 | 1.61E+03 | 9.32E+02 | 1.01E+03 | 1.01E+03 | 9.37E+02 | 9.73E+02 | 1.07E+03 | 18.92 |  | x | x |
| 229.2727 | 0.19 | 3.09E+01 | 2.52E+01 | 2.75E+01 | 2.76E+01 | 3.16E+01 | 3.39E+01 | 3.42E+01 | 3.33E+01 | 3.50E+01 | 3.12E+01 | 5.27E+01 | 3.17E+01 | 3.71E+01 | 3.44E+01 | 3.25E+01 | 3.45E+01 | 3.78E+01 | 17.68 |  | x | x |
| 229.3357 | 0.19 | 2.31E+01 | 1.62E+01 | 1.71E+01 | 1.89E+01 | 2.15E+01 | 2.36E+01 | 2.65E+01 | 2.50E+01 | 2.34E+01 | 2.51E+01 | 3.58E+01 | 1.86E+01 | 2.68E+01 | 2.24E+01 | 1.97E+01 | 2.38E+01 | 2.31E+01 | 19.84 |  | x | x |
| 266.0784 | 0.19 | 1.26E+01 | 1.19E+01 | 1.23E+01 | 1.41E+01 | 1.49E+01 | 1.75E+01 | 1.15E+01 | 1.01E+01 | 1.46E+01 | 1.25E+01 | 2.22E+01 | 1.30E+01 | 1.45E+01 | 1.38E+01 | 1.47E+01 | 1.70E+01 | 1.57E+01 | 19.72 |  | x | x |
| 249.0647 | 0.19 | 6.57E+00 | 6.36E+00 | 6.84E+00 | 6.20E+00 | 5.89E+00 | 5.21E+00 | 4.66E+00 | 5.01E+00 | 7.09E+00 | 4.88E+00 | 9.07E+00 | 4.57E+00 | 6.10E+00 | 7.51E+00 | 7.64E+00 | 3.90E+00 | 6.40E+00 | 21.58 |  |  | x |
| 257.1356 | 0.19 | 1.85E+01 | 1.68E+01 | 1.89E+01 | 1.95E+01 | 1.86E+01 | 2.36E+01 | 2.27E+01 | 1.95E+01 | 2.60E+01 | 1.92E+01 | 4.13E+01 | 1.70E+01 | 2.26E+01 | 2.50E+01 | 2.18E+01 | 2.55E+01 | 2.30E+01 | 25.49 |  |  | x |
| 261.1100 | 0.19 | 3.10E+01 | 2.86E+01 | 2.75E+01 | 3.44E+01 | 3.83E+01 | 3.92E+01 | 3.84E+01 | 3.11E+01 | 4.24E+01 | 2.69E+01 | 5.86E+01 | 2.78E+01 | 4.14E+01 | 3.91E+01 | 3.49E+01 | 3.67E+01 | 4.46E+01 | 21.76 |  |  | x |
| 271.1514 | 0.19 | 1.49E+01 | 1.15E+01 | 1.22E+01 | 1.40E+01 | 1.40E+01 | 1.70E+01 | 1.45E+01 | 1.03E+01 | 1.27E+01 | 1.24E+01 | 2.38E+01 | 1.33E+01 | 1.59E+01 | 1.58E+01 | 1.17E+01 | 1.40E+01 | 1.55E+01 | 21.15 |  |  | x |
| 284.3299 | 0.19 | 1.53E+01 | 1.76E+01 | 1.51E+01 | 1.60E+01 | 1.95E+01 | 2.15E+01 | 2.08E+01 | 1.95E+01 | 1.90E+01 | 1.53E+01 | 2.33E+01 | 1.73E+01 | 1.85E+01 | 1.43E+01 | 1.15E+01 | 9.66E+00 | 1.78E+01 | 20.31 |  |  | x |
| 306.0755 | 0.19 | 1.43E+02 | 1.27E+02 | 1.33E+02 | 1.25E+02 | 1.24E+02 | 1.39E+02 | 1.44E+02 | 1.15E+02 | 1.49E+02 | 1.35E+02 | 2.29E+02 | 1.10E+02 | 1.41E+02 | 1.47E+02 | 1.28E+02 | 1.54E+02 | 1.40E+02 | 18.48 |  | x | x |
| 307.0878 | 0.19 | 2.46E+01 | 2.94E+01 | 3.03E+01 | 3.00E+01 | 3.03E+01 | 2.77E+01 | 3.32E+01 | 3.74E+01 | 2.83E+01 | 2.53E+01 | 3.75E+01 | 3.98E+01 | 3.03E+01 | 2.52E+01 | 3.31E+01 | 2.83E+01 | 3.27E+01 | 14.32 | x | x | x |
| 326.3793 | 0.19 | 1.38E+02 | 1.28E+02 | 1.49E+02 | 1.58E+02 | 1.70E+02 | 1.78E+02 | 1.61E+02 | 1.74E+02 | 1.64E+02 | 1.58E+02 | 2.45E+02 | 1.52E+02 | 1.58E+02 | 1.59E+02 | 1.52E+02 | 1.52E+02 | 1.68E+02 | 15.09 |  | x | x |
| 332.3318 | 0.19 | 8.45E+01 | 7.80E+01 | 8.76E+01 | 9.07E+01 | 9.40E+01 | 9.78E+01 | 9.84E+01 | 8.37E+01 | 9.73E+01 | 8.88E+01 | 1.47E+02 | 8.28E+01 | 8.95E+01 | 9.99E+01 | 8.26E+01 | 9.12E+01 | 8.38E+01 | 16.46 |  | x | x |
| 361.2337 | 0.19 | 6.47E+01 | 6.25E+01 | 7.71E+01 | 6.86E+01 | 7.85E+01 | 8.00E+01 | 8.07E+01 | 7.08E+01 | 7.66E+01 | 7.03E+01 | 1.10E+02 | 6.91E+01 | 7.40E+01 | 7.89E+01 | 6.24E+01 | 7.10E+01 | 7.27E+01 | 14.59 | x | x | x |
| 340.3586 | 0.19 | 2.92E+01 | 3.09E+01 | 2.43E+01 | 3.31E+01 | 2.64E+01 | 3.49E+01 | 3.16E+01 | 2.79E+01 | 2.90E+01 | 2.47E+01 | 4.51E+01 | 2.35E+01 | 2.52E+01 | 3.15E+01 | 2.61E+01 | 2.57E+01 | 3.22E+01 | 17.95 |  | x | x |
| 353.2085 | 0.19 | 1.88E+01 | 2.06E+01 | 1.46E+01 | 2.17E+01 | 2.01E+01 | 1.65E+01 | 1.85E+01 | 1.90E+01 | 1.37E+01 | 1.10E+01 | 2.14E+01 | 1.35E+01 | 1.50E+01 | 1.27E+01 | 1.39E+01 | 1.28E+01 | 1.37E+01 | 21.24 |  |  | x |
| 405.2606 | 0.19 | 3.70E+02 | 3.49E+02 | 3.59E+02 | 3.59E+02 | 3.52E+02 | 3.82E+02 | 3.74E+02 | 3.27E+02 | 3.64E+02 | 3.33E+02 | 5.56E+02 | 3.33E+02 | 3.43E+02 | 3.73E+02 | 3.09E+02 | 3.48E+02 | 3.42E+02 | 14.63 | x | x | x |
| 449.2869 | 0.19 | 4.34E+02 | 4.25E+02 | 4.21E+02 | 4.20E+02 | 4.03E+02 | 4.39E+02 | 3.92E+02 | 4.09E+02 | 4.36E+02 | 3.89E+02 | 6.24E+02 | 3.85E+02 | 4.17E+02 | 4.35E+02 | 3.43E+02 | 4.16E+02 | 3.96E+02 | 13.56 | x | x | x |
| 493.3128 | 0.19 | 2.11E+02 | 2.21E+02 | 2.08E+02 | 1.89E+02 | 1.88E+02 | 2.03E+02 | 1.79E+02 | 1.77E+02 | 2.02E+02 | 1.84E+02 | 3.13E+02 | 1.71E+02 | 2.08E+02 | 2.15E+02 | 1.64E+02 | 2.02E+02 | 2.06E+02 | 16.17 |  | x | x |
| 553.3138 | 0.19 | 3.37E+01 | 5.04E+01 | 4.24E+01 | 3.61E+01 | 3.34E+01 | 4.28E+01 | 3.85E+01 | 3.28E+01 | 3.36E+01 | 4.09E+01 | 6.30E+01 | 3.09E+01 | 4.01E+01 | 4.32E+01 | 3.11E+01 | 4.49E+01 | 4.49E+01 | 20.35 |  |  | x |
| 186.2223 | 0.20 | 1.65E+00 | 2.06E+00 | 2.66E+00 | 2.91E+00 | 2.12E+00 | 1.22E+00 | 2.73E+00 | 3.02E+00 | 2.91E+00 | 1.87E+00 | 2.51E+00 | 3.36E+00 | 3.07E+00 | 2.21E+00 | 1.94E+00 | 1.02E+00 | 2.23E+00 | 28.42 |  |  | x |
| 229.1055 | 0.20 | 1.28E+01 | 1.24E+01 | 1.52E+01 | 1.47E+01 | 1.77E+01 | 1.65E+01 | 1.74E+01 | 1.74E+01 | 1.93E+01 | 1.62E+01 | 3.09E+01 | 1.66E+01 | 1.78E+01 | 1.77E+01 | 1.71E+01 | 1.77E+01 | 2.20E+01 | 23.24 |  |  | x |
| 219.1737 | 0.20 | 3.96E+00 | 3.38E+00 | 3.93E+00 | 5.06E+00 | 5.62E+00 | 4.54E+00 | 4.37E+00 | 3.35E+00 | 4.58E+00 | 3.70E+00 | 5.45E+00 | 4.83E+00 | 4.14E+00 | 3.30E+00 | 5.72E+00 | 3.85E+00 | 4.01E+00 | 17.96 |  | x | x |
| 227.9689 | 0.20 | 3.64E+00 | 2.37E+00 | 3.53E+00 | 4.15E+00 | 3.16E+00 | 2.76E+00 | 3.67E+00 | 3.88E+00 | 4.11E+00 | 2.94E+00 | 5.43E+00 | 3.16E+00 | 2.71E+00 | 2.86E+00 | 1.72E+00 | 4.10E+00 | 3.19E+00 | 25.04 |  |  | x |
| 273.1287 | 0.20 | 4.82E+01 | 5.27E+01 | 5.28E+01 | 5.24E+01 | 5.14E+01 | 5.98E+01 | 5.86E+01 | 5.26E+01 | 5.35E+01 | 3.97E+01 | 8.59E+01 | 5.83E+01 | 5.57E+01 | 5.74E+01 | 5.82E+01 | 4.99E+01 | 5.86E+01 | 16.65 |  | x | x |
| 273.1675 | 0.20 | 9.47E+01 | 1.02E+02 | 1.02E+02 | 9.80E+01 | 9.51E+01 | 1.10E+02 | 1.15E+02 | 1.02E+02 | 1.13E+02 | 9.22E+01 | 1.70E+02 | 8.92E+01 | 1.00E+02 | 9.51E+01 | 9.19E+01 | 9.90E+01 | 1.13E+02 | 17.78 |  | x | x |
| 304.3023 | 0.20 | 7.31E+01 | 6.84E+01 | 7.57E+01 | 8.02E+01 | 9.64E+01 | 8.62E+01 | 8.83E+01 | 8.69E+01 | 9.25E+01 | 7.77E+01 | 1.32E+02 | 7.90E+01 | 8.69E+01 | 8.49E+01 | 7.77E+01 | 8.71E+01 | 8.14E+01 | 16.21 |  | x | x |
| 356.3519 | 0.20 | 6.91E+01 | 7.44E+01 | 7.76E+01 | 7.43E+01 | 7.79E+01 | 8.19E+01 | 7.97E+01 | 7.11E+01 | 8.74E+01 | 6.51E+01 | 1.19E+02 | 7.22E+01 | 6.61E+01 | 5.34E+01 | 5.34E+01 | 6.76E+01 | 6.68E+01 | 19.74 |  | x | x |
| 331.2087 | 0.20 | 2.12E+01 | 1.79E+01 | 1.79E+01 | 1.90E+01 | 1.99E+01 | 1.76E+01 | 1.98E+01 | 1.04E+01 | 2.11E+01 | 1.75E+01 | 3.50E+01 | 1.75E+01 | 1.97E+01 | 1.91E+01 | 1.70E+01 | 1.72E+01 | 2.19E+01 | 24.6 |  |  | x |
| 377.2267 | 0.20 | 1.96E+01 | 1.35E+01 | 1.90E+01 | 1.60E+01 | 2.16E+01 | 2.40E+01 | 2.71E+01 | 1.81E+01 | 2.35E+01 | 1.75E+01 | 2.97E+01 | 6.92E+00 | 2.16E+01 | 1.99E+01 | 1.20E+01 | 2.13E+01 | 2.20E+01 | 28.07 |  |  | x |
| 134.0960 | 0.21 | 1.13E+01 | 1.20E+01 | 1.07E+01 | 1.38E+01 | 1.25E+01 | 1.24E+01 | 1.20E+01 | 1.26E+01 | 1.34E+01 | 1.22E+01 | 2.17E+01 | 1.05E+01 | 1.23E+01 | 1.16E+01 | 1.23E+01 | 1.25E+01 | 1.23E+01 | 19.29 |  | x | x |
| 116.1069 | 0.21 | 2.28E+02 | 2.03E+02 | 2.24E+02 | 2.26E+02 | 2.41E+02 | 2.47E+02 | 2.37E+02 | 2.31E+02 | 2.54E+02 | 2.21E+02 | 3.72E+02 | 2.60E+02 | 2.36E+02 | 2.31E+02 | 2.32E+02 | 2.43E+02 | 2.51E+02 | 14.73 | x | x | x |
| 116.1984 | 0.21 | 2.52E+00 | 1.98E+00 | 2.09E+00 | 2.18E+00 | 2.32E+00 | 2.77E+00 | 2.00E+00 | 1.95E+00 | 2.63E+00 | 1.88E+00 | 3.86E+00 | 3.00E+00 | 1.98E+00 | 1.23E+00 | 2.36E+00 | 2.65E+00 | 2.31E+00 | 24.45 |  |  | x |
| 151.1228 | 0.21 | 1.96E+02 | 1.78E+02 | 1.88E+02 | 1.97E+02 | 2.02E+02 | 2.31E+02 | 2.08E+02 | 2.13E+02 | 2.08E+02 | 2.08E+02 | 3.27E+02 | 2.07E+02 | 2.07E+02 | 2.28E+02 | 2.21E+02 | 2.13E+02 | 2.24E+02 | 14.85 | x | x | x |
| 137.1072 | 0.21 | 3.05E+02 | 2.74E+02 | 2.97E+02 | 3.26E+02 | 3.19E+02 | 3.53E+02 | 3.39E+02 | 3.49E+02 | 3.55E+02 | 3.13E+02 | 5.50E+02 | 3.34E+02 | 3.26E+02 | 3.40E+02 | 3.17E+02 | 3.36E+02 | 3.47E+02 | 17.09 |  | x | x |
| 165.1383 | 0.21 | 3.20E+01 | 3.09E+01 | 2.88E+01 | 2.90E+01 | 3.13E+01 | 3.56E+01 | 3.16E+01 | 2.94E+01 | 3.50E+01 | 3.36E+01 | 5.24E+01 | 2.67E+01 | 3.02E+01 | 3.63E+01 | 3.42E+01 | 3.44E+01 | 3.38E+01 | 16.89 |  | x | x |
| 362.3050 | 0.21 | 1.67E+01 | 1.66E+01 | 1.99E+01 | 2.03E+01 | 1.65E+01 | 1.86E+01 | 1.75E+01 | 2.00E+01 | 1.50E+01 | 1.51E+01 | 2.48E+01 | 1.55E+01 | 1.14E+01 | 1.38E+01 | 1.47E+01 | 1.68E+01 | 1.53E+01 | 18.13 |  | x | x |
| 178.1585 | 0.21 | 3.05E+01 | 2.67E+01 | 2.86E+01 | 3.18E+01 | 2.46E+01 | 3.09E+01 | 2.81E+01 | 2.69E+01 | 3.55E+01 | 2.50E+01 | 4.18E+01 | 2.71E+01 | 2.71E+01 | 2.89E+01 | 2.83E+01 | 3.14E+01 | 2.78E+01 | 14.15 | x | x | x |
| 202.2169 | 0.21 | 1.25E+01 | 1.07E+01 | 1.24E+01 | 1.47E+01 | 1.31E+01 | 1.50E+01 | 1.70E+01 | 1.57E+01 | 1.83E+01 | 1.11E+01 | 2.35E+01 | 1.16E+01 | 1.16E+01 | 1.67E+01 | 1.48E+01 | 1.44E+01 | 1.39E+01 | 21.88 |  |  | x |
| 231.1196 | 0.21 | 2.00E+01 | 2.12E+01 | 1.94E+01 | 2.11E+01 | 2.12E+01 | 2.49E+01 | 2.13E+01 | 2.07E+01 | 1.77E+01 | 1.97E+01 | 3.49E+01 | 1.64E+01 | 2.37E+01 | 2.60E+01 | 1.48E+01 | 2.26E+01 | 2.18E+01 | 20.49 |  |  | x |
| 215.1251 | 0.21 | 6.16E+01 | 5.47E+01 | 5.96E+01 | 6.13E+01 | 7.23E+01 | 7.12E+01 | 6.41E+01 | 6.92E+01 | 7.26E+01 | 6.46E+01 | 1.02E+02 | 6.21E+01 | 6.81E+01 | 7.25E+01 | 6.74E+01 | 6.65E+01 | 6.86E+01 | 14.87 | x | x | x |
| 216.1952 | 0.21 | 5.57E+00 | 3.75E+00 | 5.10E+00 | 6.68E+00 | 6.25E+00 | 6.02E+00 | 6.71E+00 | 8.20E+00 | 5.41E+00 | 5.18E+00 | 8.23E+00 | 9.04E+00 | 5.38E+00 | 5.46E+00 | 4.54E+00 | 4.93E+00 | 4.06E+00 | 24.91 |  |  | x |
| 259.1149 | 0.21 | 7.30E+00 | 1.08E+01 | 9.30E+00 | 8.97E+00 | 1.01E+01 | 6.97E+00 | 8.19E+00 | 7.13E+00 | 7.48E+00 | 7.54E+00 | 1.43E+01 | 7.73E+00 | 9.44E+00 | 7.69E+00 | 6.60E+00 | 8.91E+00 | 7.95E+00 | 21.64 |  |  | x |
| 291.1204 | 0.21 | 1.38E+01 | 1.46E+01 | 1.18E+01 | 1.45E+01 | 1.45E+01 | 1.35E+01 | 1.15E+01 | 1.09E+01 | 1.30E+01 | 1.39E+01 | 2.22E+01 | 1.37E+01 | 1.37E+01 | 1.55E+01 | 1.27E+01 | 1.21E+01 | 1.30E+01 | 17.95 |  | x | x |
| 299.1756 | 0.21 | 2.16E+01 | 1.75E+01 | 1.92E+01 | 1.97E+01 | 1.63E+01 | 1.59E+01 | 1.77E+01 | 2.01E+01 | 1.60E+01 | 1.60E+01 | 2.84E+01 | 1.89E+01 | 1.87E+01 | 1.68E+01 | 1.52E+01 | 1.64E+01 | 2.41E+01 | 18.26 |  | x | x |
| 358.3671 | 0.21 | 1.58E+01 | 2.02E+01 | 2.61E+01 | 1.60E+01 | 2.08E+01 | 1.99E+01 | 2.05E+01 | 1.98E+01 | 1.87E+01 | 2.00E+01 | 2.47E+01 | 1.80E+01 | 1.68E+01 | 1.58E+01 | 1.52E+01 | 1.87E+01 | 2.13E+01 | 15.73 |  | x | x |
| 331.1297 | 0.21 | 4.12E+01 | 3.09E+01 | 3.16E+01 | 2.65E+01 | 2.65E+01 | 2.95E+01 | 2.44E+01 | 2.57E+01 | 2.46E+01 | 2.68E+01 | 4.24E+01 | 1.63E+01 | 2.79E+01 | 2.64E+01 | 2.06E+01 | 2.81E+01 | 2.64E+01 | 22.53 |  |  | x |
| 425.1474 | 0.21 | 7.14E+02 | 6.39E+02 | 7.43E+02 | 6.28E+02 | 6.31E+02 | 6.12E+02 | 6.09E+02 | 6.22E+02 | 5.95E+02 | 5.42E+02 | 9.31E+02 | 6.60E+02 | 5.50E+02 | 5.77E+02 | 5.48E+02 | 5.42E+02 | 5.73E+02 | 15.26 |  | x | x |
| 102.1280 | 0.22 | 5.12E+00 | 4.40E+00 | 3.95E+00 | 3.64E+00 | 3.89E+00 | 5.23E+00 | 2.45E+00 | 3.70E+00 | 4.57E+00 | 5.20E+00 | 4.80E+00 | 1.90E+00 | 4.41E+00 | 6.39E+00 | 4.34E+00 | 7.56E+00 | 5.17E+00 | 29.29 |  |  | x |
| 163.1206 | 0.22 | 2.56E+01 | 2.61E+01 | 2.61E+01 | 2.76E+01 | 3.12E+01 | 3.36E+01 | 3.08E+01 | 3.15E+01 | 3.20E+01 | 2.92E+01 | 5.04E+01 | 2.24E+01 | 3.23E+01 | 2.92E+01 | 3.20E+01 | 3.00E+01 | 3.00E+01 | 19.28 |  | x | x |
| 153.1382 | 0.22 | 2.86E+01 | 2.50E+01 | 2.74E+01 | 2.70E+01 | 2.72E+01 | 2.70E+01 | 2.85E+01 | 3.22E+01 | 2.92E+01 | 2.59E+01 | 4.87E+01 | 2.55E+01 | 2.91E+01 | 2.86E+01 | 2.76E+01 | 2.90E+01 | 3.24E+01 | 18.3 |  | x | x |
| 181.1327 | 0.22 | 1.10E+02 | 1.01E+02 | 1.14E+02 | 1.22E+02 | 1.26E+02 | 1.30E+02 | 1.30E+02 | 1.34E+02 | 1.31E+02 | 1.22E+02 | 2.13E+02 | 1.20E+02 | 1.31E+02 | 1.30E+02 | 1.19E+02 | 1.33E+02 | 1.34E+02 | 18.02 |  | x | x |
| 194.1898 | 0.22 | 1.38E+02 | 1.27E+02 | 1.41E+02 | 1.42E+02 | 1.52E+02 | 1.54E+02 | 1.49E+02 | 1.57E+02 | 1.65E+02 | 1.44E+02 | 2.47E+02 | 1.25E+02 | 1.58E+02 | 1.71E+02 | 1.56E+02 | 1.64E+02 | 1.61E+02 | 17.15 |  | x | x |
| 287.1456 | 0.22 | 2.04E+01 | 1.71E+01 | 1.90E+01 | 2.05E+01 | 2.24E+01 | 1.87E+01 | 1.77E+01 | 1.42E+01 | 1.76E+01 | 2.01E+01 | 2.92E+01 | 1.01E+01 | 2.38E+01 | 1.94E+01 | 1.76E+01 | 2.30E+01 | 2.39E+01 | 21.61 |  |  | x |
| 194.0920 | 0.23 | 4.32E+01 | 3.92E+01 | 4.10E+01 | 4.14E+01 | 3.71E+01 | 3.87E+01 | 4.07E+01 | 3.71E+01 | 4.26E+01 | 3.55E+01 | 6.43E+01 | 3.29E+01 | 3.87E+01 | 3.95E+01 | 3.87E+01 | 4.63E+01 | 4.30E+01 | 16.42 |  | x | x |
| 108.0803 | 0.23 | 8.04E+00 | 6.73E+00 | 7.27E+00 | 6.77E+00 | 5.73E+00 | 1.08E+01 | 7.50E+00 | 5.69E+00 | 7.21E+00 | 6.73E+00 | 1.32E+01 | 7.31E+00 | 7.75E+00 | 8.62E+00 | 5.68E+00 | 8.88E+00 | 8.07E+00 | 24.61 |  |  | x |
| 115.0539 | 0.23 | 1.29E+01 | 1.01E+01 | 1.09E+01 | 1.38E+01 | 1.22E+01 | 1.33E+01 | 1.05E+01 | 1.14E+01 | 9.53E+00 | 1.12E+01 | 1.93E+01 | 8.72E+00 | 1.08E+01 | 1.37E+01 | 1.29E+01 | 1.05E+01 | 1.33E+01 | 20.02 |  |  | x |
| 122.0968 | 0.23 | 2.70E+02 | 2.41E+02 | 2.47E+02 | 2.86E+02 | 2.79E+02 | 2.93E+02 | 2.90E+02 | 2.69E+02 | 2.97E+02 | 2.83E+02 | 4.66E+02 | 2.71E+02 | 3.02E+02 | 3.08E+02 | 3.05E+02 | 2.93E+02 | 3.11E+02 | 16.38 |  | x | x |
| 122.1527 | 0.23 | 2.17E+00 | 1.45E+00 | 1.76E+00 | 2.35E+00 | 2.08E+00 | 1.69E+00 | 2.37E+00 | 1.62E+00 | 1.93E+00 | 1.83E+00 | 3.03E+00 | 1.93E+00 | 1.66E+00 | 1.35E+00 | 2.17E+00 | 2.18E+00 | 1.53E+00 | 21.32 |  |  | x |
| 122.1910 | 0.23 | 5.51E+00 | 4.48E+00 | 4.25E+00 | 5.28E+00 | 5.38E+00 | 5.39E+00 | 4.35E+00 | 3.68E+00 | 4.60E+00 | 4.95E+00 | 8.08E+00 | 4.43E+00 | 5.87E+00 | 5.49E+00 | 5.35E+00 | 5.84E+00 | 5.97E+00 | 18.81 |  | x | x |
| 124.0886 | 0.23 | 3.67E+00 | 4.39E+00 | 2.86E+00 | 4.62E+00 | 3.76E+00 | 2.64E+00 | 3.12E+00 | 3.06E+00 | 3.42E+00 | 3.42E+00 | 4.26E+00 | 4.26E+00 | 4.06E+00 | 1.97E+00 | 3.23E+00 | 2.84E+00 | 5.24E+00 | 23.09 |  |  | x |
| 125.1075 | 0.23 | 6.32E+01 | 5.41E+01 | 5.81E+01 | 6.15E+01 | 5.92E+01 | 7.19E+01 | 6.65E+01 | 6.90E+01 | 7.31E+01 | 6.53E+01 | 1.14E+02 | 6.09E+01 | 7.50E+01 | 7.38E+01 | 6.58E+01 | 6.94E+01 | 7.39E+01 | 19.05 |  | x | x |
| 160.0763 | 0.23 | 6.13E+01 | 5.19E+01 | 5.97E+01 | 6.67E+01 | 6.46E+01 | 6.61E+01 | 5.59E+01 | 6.32E+01 | 5.94E+01 | 5.26E+01 | 8.66E+01 | 5.46E+01 | 5.77E+01 | 5.41E+01 | 6.71E+01 | 5.65E+01 | 5.82E+01 | 13.49 | x | x | x |
| 141.0597 | 0.23 | 9.86E+00 | 6.80E+00 | 7.67E+00 | 9.40E+00 | 7.34E+00 | 6.51E+00 | 9.50E+00 | 8.37E+00 | 5.05E+00 | 9.50E+00 | 1.44E+01 | 5.19E+00 | 8.58E+00 | 8.81E+00 | 7.02E+00 | 8.43E+00 | 7.55E+00 | 26.08 |  |  | x |
| 144.0792 | 0.23 | 1.42E+01 | 1.53E+01 | 1.45E+01 | 1.27E+01 | 1.31E+01 | 1.82E+01 | 1.71E+01 | 1.41E+01 | 2.08E+01 | 1.92E+01 | 2.81E+01 | 8.23E+00 | 2.04E+01 | 2.07E+01 | 1.54E+01 | 2.08E+01 | 1.47E+01 | 26.73 |  |  | x |
| 156.0807 | 0.23 | 8.21E+01 | 7.54E+01 | 8.50E+01 | 8.48E+01 | 8.81E+01 | 9.69E+01 | 9.30E+01 | 8.64E+01 | 9.11E+01 | 8.43E+01 | 1.51E+02 | 7.93E+01 | 8.67E+01 | 8.84E+01 | 8.61E+01 | 6.83E+01 | 8.76E+01 | 19.31 |  | x | x |
| 166.0497 | 0.23 | 1.74E+01 | 1.67E+01 | 1.68E+01 | 1.60E+01 | 2.05E+01 | 2.17E+01 | 2.38E+01 | 2.18E+01 | 2.20E+01 | 1.78E+01 | 4.01E+01 | 2.14E+01 | 1.86E+01 | 2.05E+01 | 2.06E+01 | 2.22E+01 | 2.66E+01 | 25.94 |  |  | x |
| 186.1125 | 0.23 | 1.34E+02 | 1.24E+02 | 1.33E+02 | 1.37E+02 | 1.44E+02 | 1.43E+02 | 1.46E+02 | 1.54E+02 | 1.58E+02 | 1.39E+02 | 2.36E+02 | 1.77E+02 | 1.45E+02 | 1.42E+02 | 1.35E+02 | 1.48E+02 | 1.48E+02 | 16.76 |  | x | x |
| 202.1222 | 0.23 | 6.33E+01 | 6.01E+01 | 6.69E+01 | 6.67E+01 | 7.05E+01 | 6.74E+01 | 7.79E+01 | 6.91E+01 | 6.91E+01 | 6.69E+01 | 9.59E+01 | 5.67E+01 | 6.89E+01 | 6.51E+01 | 6.63E+01 | 6.97E+01 | 6.73E+01 | 12.11 | x | x | x |
| 172.9767 | 0.23 | 2.33E+01 | 1.98E+01 | 2.06E+01 | 3.20E+01 | 3.84E+01 | 3.83E+01 | 4.08E+01 | 3.73E+01 | 3.47E+01 | 2.74E+01 | 5.53E+01 | 3.70E+01 | 2.65E+01 | 2.15E+01 | 2.91E+01 | 2.45E+01 | 2.56E+01 | 29.62 |  |  | x |
| 188.0706 | 0.23 | 1.98E+01 | 1.53E+01 | 1.52E+01 | 1.67E+01 | 1.88E+01 | 2.06E+01 | 1.60E+01 | 1.63E+01 | 1.99E+01 | 1.57E+01 | 2.99E+01 | 1.39E+01 | 2.02E+01 | 2.08E+01 | 1.49E+01 | 2.14E+01 | 1.99E+01 | 20.55 |  |  | x |
| 195.1116 | 0.23 | 3.53E+01 | 3.28E+01 | 3.50E+01 | 3.80E+01 | 3.55E+01 | 4.31E+01 | 3.63E+01 | 3.47E+01 | 3.89E+01 | 3.56E+01 | 5.92E+01 | 3.34E+01 | 4.16E+01 | 4.02E+01 | 3.36E+01 | 3.33E+01 | 3.78E+01 | 16.49 |  | x | x |
| 197.0600 | 0.23 | 1.52E+01 | 1.24E+01 | 1.46E+01 | 1.64E+01 | 1.46E+01 | 1.47E+01 | 1.18E+01 | 8.90E+00 | 1.53E+01 | 1.15E+01 | 1.99E+01 | 8.94E+00 | 1.10E+01 | 1.21E+01 | 1.05E+01 | 1.53E+01 | 1.62E+01 | 21.81 |  |  | x |
| 210.9951 | 0.23 | 1.52E+01 | 1.54E+01 | 1.81E+01 | 1.54E+01 | 1.55E+01 | 1.81E+01 | 1.40E+01 | 1.28E+01 | 1.34E+01 | 1.33E+01 | 2.42E+01 | 1.14E+01 | 1.28E+01 | 1.35E+01 | 1.25E+01 | 1.30E+01 | 1.17E+01 | 21.22 |  |  | x |
| 212.9956 | 0.23 | 1.37E+01 | 1.29E+01 | 1.22E+01 | 1.36E+01 | 1.23E+01 | 1.39E+01 | 1.38E+01 | 1.63E+01 | 1.26E+01 | 1.24E+01 | 2.08E+01 | 1.69E+01 | 1.17E+01 | 1.32E+01 | 1.37E+01 | 9.33E+00 | 1.30E+01 | 18.21 |  | x | x |
| 213.0565 | 0.23 | 4.43E+00 | 3.74E+00 | 3.72E+00 | 3.63E+00 | 2.96E+00 | 4.23E+00 | 3.80E+00 | 2.45E+00 | 5.54E+00 | 3.21E+00 | 4.48E+00 | 3.76E+00 | 2.29E+00 | 3.15E+00 | 2.98E+00 | 3.55E+00 | 2.00E+00 | 24.97 |  |  | x |
| 245.1354 | 0.23 | 6.01E+01 | 5.22E+01 | 6.22E+01 | 5.53E+01 | 6.23E+01 | 6.87E+01 | 5.91E+01 | 5.67E+01 | 6.43E+01 | 5.64E+01 | 8.52E+01 | 6.30E+01 | 6.52E+01 | 6.17E+01 | 5.55E+01 | 6.47E+01 | 6.06E+01 | 11.85 | x | x | x |
| 225.0544 | 0.23 | 4.18E+00 | 3.36E+00 | 3.30E+00 | 3.45E+00 | 4.36E+00 | 3.81E+00 | 4.73E+00 | 3.16E+00 | 3.61E+00 | 4.37E+00 | 6.67E+00 | 2.95E+00 | 3.98E+00 | 4.38E+00 | 1.86E+00 | 4.72E+00 | 3.39E+00 | 26.12 |  |  | x |
| 225.1094 | 0.23 | 6.26E+00 | 5.78E+00 | 5.91E+00 | 5.90E+00 | 7.46E+00 | 8.09E+00 | 5.38E+00 | 5.03E+00 | 6.73E+00 | 6.76E+00 | 9.81E+00 | 6.34E+00 | 5.17E+00 | 6.42E+00 | 2.43E+00 | 5.54E+00 | 4.70E+00 | 25.59 |  |  | x |
| 226.1425 | 0.23 | 1.20E+01 | 1.23E+01 | 1.30E+01 | 1.18E+01 | 9.13E+00 | 1.24E+01 | 1.23E+01 | 1.28E+01 | 1.46E+01 | 9.73E+00 | 1.61E+01 | 1.80E+01 | 1.17E+01 | 1.13E+01 | 1.19E+01 | 1.22E+01 | 1.18E+01 | 16.77 |  | x | x |
| 234.1482 | 0.23 | 3.13E+01 | 3.06E+01 | 3.42E+01 | 3.64E+01 | 3.03E+01 | 3.55E+01 | 3.45E+01 | 3.15E+01 | 3.40E+01 | 3.13E+01 | 5.32E+01 | 3.93E+01 | 3.37E+01 | 3.62E+01 | 3.12E+01 | 3.15E+01 | 3.40E+01 | 15.51 |  | x | x |
| 238.9913 | 0.23 | 1.50E+01 | 1.49E+01 | 1.74E+01 | 1.25E+01 | 1.26E+01 | 1.20E+01 | 1.55E+01 | 1.24E+01 | 1.33E+01 | 1.14E+01 | 2.42E+01 | 7.86E+00 | 1.42E+01 | 1.68E+01 | 1.08E+01 | 1.16E+01 | 1.09E+01 | 26.19 |  |  | x |
| 241.1041 | 0.23 | 2.79E+01 | 2.19E+01 | 2.21E+01 | 1.59E+01 | 1.69E+01 | 1.85E+01 | 1.75E+01 | 1.65E+01 | 1.87E+01 | 1.67E+01 | 2.39E+01 | 1.48E+01 | 1.94E+01 | 1.87E+01 | 1.27E+01 | 1.63E+01 | 1.24E+01 | 21.66 |  |  | x |
| 242.0580 | 0.23 | 1.83E+01 | 1.69E+01 | 2.14E+01 | 1.36E+01 | 1.63E+01 | 2.05E+01 | 1.52E+01 | 1.12E+01 | 1.52E+01 | 1.09E+01 | 1.96E+01 | 1.50E+01 | 1.60E+01 | 2.33E+01 | 1.66E+01 | 1.62E+01 | 1.72E+01 | 19.69 |  | x | x |
| 245.0973 | 0.23 | 6.66E+00 | 7.36E+00 | 7.80E+00 | 7.98E+00 | 8.88E+00 | 8.36E+00 | 8.79E+00 | 8.50E+00 | 1.05E+01 | 5.83E+00 | 1.41E+01 | 1.05E+01 | 8.39E+00 | 5.09E+00 | 3.98E+00 | 6.59E+00 | 7.55E+00 | 28.78 |  |  | x |
| 256.0136 | 0.23 | 3.33E+01 | 2.52E+01 | 3.01E+01 | 4.30E+01 | 4.76E+01 | 4.37E+01 | 4.06E+01 | 3.54E+01 | 4.19E+01 | 3.40E+01 | 5.97E+01 | 1.26E+01 | 4.25E+01 | 3.52E+01 | 4.05E+01 | 3.60E+01 | 2.85E+01 | 27.58 |  |  | x |
| 258.0121 | 0.23 | 1.14E+01 | 8.71E+00 | 9.34E+00 | 1.37E+01 | 1.36E+01 | 1.76E+01 | 1.32E+01 | 1.44E+01 | 1.28E+01 | 1.09E+01 | 1.69E+01 | 4.42E+00 | 1.47E+01 | 1.41E+01 | 1.27E+01 | 1.11E+01 | 1.03E+01 | 25.6 |  |  | x |
| 271.0604 | 0.23 | 2.46E+01 | 2.14E+01 | 2.35E+01 | 1.89E+01 | 2.42E+01 | 2.87E+01 | 2.10E+01 | 2.21E+01 | 2.16E+01 | 2.33E+01 | 3.52E+01 | 2.12E+01 | 2.24E+01 | 1.90E+01 | 2.28E+01 | 2.12E+01 | 2.14E+01 | 16.6 |  | x | x |
| 285.0769 | 0.23 | 1.75E+03 | 1.50E+03 | 1.60E+03 | 1.60E+03 | 1.69E+03 | 1.86E+03 | 1.71E+03 | 1.62E+03 | 1.78E+03 | 1.66E+03 | 2.77E+03 | 1.58E+03 | 1.72E+03 | 1.71E+03 | 1.62E+03 | 1.70E+03 | 1.80E+03 | 16 |  | x | x |
| 307.0598 | 0.23 | 2.65E+01 | 2.99E+01 | 2.81E+01 | 2.30E+01 | 3.72E+01 | 4.61E+01 | 2.64E+01 | 3.12E+01 | 2.76E+01 | 2.80E+01 | 4.61E+01 | 3.11E+01 | 3.51E+01 | 4.67E+01 | 3.53E+01 | 3.42E+01 | 3.52E+01 | 21.75 |  |  | x |
| 307.1466 | 0.23 | 4.54E+00 | 5.23E+00 | 6.46E+00 | 5.37E+00 | 7.32E+00 | 6.98E+00 | 5.13E+00 | 4.90E+00 | 7.94E+00 | 7.32E+00 | 9.20E+00 | 4.88E+00 | 7.77E+00 | 6.92E+00 | 4.47E+00 | 5.98E+00 | 6.26E+00 | 21.88 |  |  | x |
| 309.0869 | 0.23 | 1.54E+02 | 1.29E+02 | 1.55E+02 | 1.40E+02 | 1.34E+02 | 1.53E+02 | 1.44E+02 | 1.33E+02 | 1.44E+02 | 1.25E+02 | 2.13E+02 | 1.24E+02 | 1.26E+02 | 1.39E+02 | 1.19E+02 | 1.40E+02 | 1.31E+02 | 15.12 |  | x | x |
| 346.9970 | 0.23 | 7.08E+01 | 6.14E+01 | 6.49E+01 | 8.72E+01 | 9.96E+01 | 8.60E+01 | 8.52E+01 | 6.59E+01 | 6.86E+01 | 4.90E+01 | 8.53E+01 | 1.13E+02 | 5.74E+01 | 5.04E+01 | 6.06E+01 | 4.42E+01 | 3.69E+01 | 29.37 |  |  | x |
| 315.0857 | 0.23 | 4.61E+01 | 3.50E+01 | 3.35E+01 | 3.59E+01 | 2.91E+01 | 2.30E+01 | 3.80E+01 | 3.42E+01 | 3.60E+01 | 3.05E+01 | 5.84E+01 | 2.33E+01 | 3.44E+01 | 3.34E+01 | 2.72E+01 | 4.14E+01 | 2.82E+01 | 24.74 |  |  | x |
| 321.1622 | 0.23 | 7.28E+00 | 1.07E+01 | 8.68E+00 | 6.67E+00 | 7.58E+00 | 1.01E+01 | 8.64E+00 | 7.37E+00 | 8.08E+00 | 6.29E+00 | 9.19E+00 | 7.63E+00 | 7.05E+00 | 8.01E+00 | 6.75E+00 | 8.73E+00 | 6.11E+00 | 16.17 |  | x | x |
| 323.1796 | 0.23 | 1.94E+01 | 1.92E+01 | 1.92E+01 | 1.82E+01 | 1.80E+01 | 1.21E+01 | 1.80E+01 | 1.52E+01 | 1.53E+01 | 1.26E+01 | 1.50E+01 | 1.42E+01 | 1.14E+01 | 1.51E+01 | 1.32E+01 | 1.69E+01 | 1.57E+01 | 16.24 |  | x | x |
| 330.9594 | 0.23 | 5.49E+01 | 4.89E+01 | 5.39E+01 | 4.31E+01 | 4.79E+01 | 4.67E+01 | 4.45E+01 | 4.12E+01 | 4.40E+01 | 4.46E+01 | 6.55E+01 | 1.96E+01 | 4.10E+01 | 4.40E+01 | 4.00E+01 | 4.13E+01 | 4.14E+01 | 20.53 |  |  | x |
| 332.9568 | 0.23 | 3.43E+01 | 3.87E+01 | 3.47E+01 | 3.15E+01 | 3.14E+01 | 3.12E+01 | 3.36E+01 | 2.71E+01 | 2.94E+01 | 2.87E+01 | 4.84E+01 | 1.40E+01 | 3.05E+01 | 3.21E+01 | 2.79E+01 | 2.95E+01 | 2.48E+01 | 22.14 |  |  | x |
| 298.0033 | 0.23 | 3.64E+02 | 3.11E+02 | 3.51E+02 | 3.32E+02 | 3.35E+02 | 3.39E+02 | 3.45E+02 | 3.28E+02 | 3.59E+02 | 3.04E+02 | 5.36E+02 | 2.65E+02 | 3.39E+02 | 3.37E+02 | 3.04E+02 | 3.16E+02 | 3.31E+02 | 16.35 |  | x | x |
| 300.0010 | 0.23 | 2.62E+02 | 2.24E+02 | 2.47E+02 | 2.33E+02 | 2.42E+02 | 2.34E+02 | 2.43E+02 | 2.37E+02 | 2.59E+02 | 2.18E+02 | 3.75E+02 | 1.93E+02 | 2.38E+02 | 2.23E+02 | 2.15E+02 | 2.20E+02 | 2.39E+02 | 15.84 |  | x | x |
| 340.1182 | 0.23 | 1.32E+01 | 1.05E+01 | 1.25E+01 | 8.78E+00 | 1.30E+01 | 1.30E+01 | 1.04E+01 | 7.88E+00 | 9.34E+00 | 4.66E+00 | 1.20E+01 | 9.68E+00 | 9.54E+00 | 8.01E+00 | 7.50E+00 | 9.48E+00 | 5.40E+00 | 26.27 |  |  | x |
| 350.2323 | 0.23 | 2.80E+02 | 2.49E+02 | 2.82E+02 | 2.79E+02 | 2.89E+02 | 3.06E+02 | 2.79E+02 | 2.73E+02 | 2.88E+02 | 2.58E+02 | 4.48E+02 | 1.44E+02 | 2.63E+02 | 2.61E+02 | 2.50E+02 | 2.66E+02 | 2.58E+02 | 20.5 |  |  | x |
| 395.2353 | 0.23 | 1.01E+02 | 8.59E+01 | 9.17E+01 | 8.30E+01 | 7.68E+01 | 8.24E+01 | 7.28E+01 | 7.40E+01 | 6.51E+01 | 6.53E+01 | 1.09E+02 | 6.01E+01 | 6.80E+01 | 6.99E+01 | 5.86E+01 | 6.48E+01 | 5.85E+01 | 19.56 |  | x | x |
| 357.1689 | 0.23 | 8.65E+00 | 7.55E+00 | 7.68E+00 | 4.24E+00 | 3.82E+00 | 6.59E+00 | 7.81E+00 | 7.59E+00 | 7.86E+00 | 4.82E+00 | 7.98E+00 | 5.42E+00 | 6.10E+00 | 4.06E+00 | 5.78E+00 | 4.69E+00 | 7.22E+00 | 25.03 |  |  | x |
| 397.2513 | 0.23 | 4.90E+01 | 4.92E+01 | 5.13E+01 | 3.99E+01 | 3.76E+01 | 4.05E+01 | 3.15E+01 | 4.49E+01 | 3.70E+01 | 2.94E+01 | 4.64E+01 | 3.15E+01 | 3.64E+01 | 3.34E+01 | 2.78E+01 | 3.46E+01 | 2.66E+01 | 20.58 |  |  | x |
| 411.2278 | 0.23 | 4.39E+01 | 4.16E+01 | 4.16E+01 | 3.33E+01 | 3.17E+01 | 3.48E+01 | 2.90E+01 | 3.80E+01 | 2.75E+01 | 2.43E+01 | 5.03E+01 | 3.29E+01 | 2.41E+01 | 2.59E+01 | 2.41E+01 | 2.57E+01 | 2.98E+01 | 23.95 |  |  | x |
| 152.0644 | 0.24 | 3.71E+01 | 3.38E+01 | 4.09E+01 | 4.53E+01 | 3.73E+01 | 4.36E+01 | 3.51E+01 | 3.52E+01 | 3.74E+01 | 3.21E+01 | 5.90E+01 | 2.18E+01 | 3.82E+01 | 3.79E+01 | 3.33E+01 | 3.39E+01 | 3.38E+01 | 20.26 |  |  | x |
| 255.2042 | 0.24 | 1.10E+02 | 9.27E+01 | 9.72E+01 | 1.03E+02 | 9.58E+01 | 1.03E+02 | 9.76E+01 | 8.97E+01 | 1.03E+02 | 9.29E+01 | 1.58E+02 | 8.69E+01 | 9.40E+01 | 9.57E+01 | 9.85E+01 | 9.77E+01 | 9.54E+01 | 15.72 |  | x | x |
| 135.0917 | 0.24 | 5.32E+00 | 5.12E+00 | 5.04E+00 | 7.21E+00 | 6.58E+00 | 8.19E+00 | 6.82E+00 | 7.60E+00 | 6.26E+00 | 5.70E+00 | 1.07E+01 | 5.76E+00 | 7.55E+00 | 7.67E+00 | 8.71E+00 | 7.29E+00 | 4.25E+00 | 23.23 |  |  | x |
| 158.0272 | 0.24 | 8.51E+02 | 7.55E+02 | 8.57E+02 | 9.08E+02 | 9.10E+02 | 9.96E+02 | 1.01E+03 | 9.57E+02 | 1.04E+03 | 8.88E+02 | 1.55E+03 | 1.18E+03 | 9.06E+02 | 8.95E+02 | 8.73E+02 | 9.21E+02 | 9.56E+02 | 18.23 |  | x | x |
| 164.0705 | 0.24 | 2.39E+01 | 1.98E+01 | 2.10E+01 | 2.35E+01 | 1.98E+01 | 2.57E+01 | 2.15E+01 | 2.12E+01 | 2.17E+01 | 1.90E+01 | 3.17E+01 | 2.29E+01 | 2.18E+01 | 1.95E+01 | 2.12E+01 | 2.14E+01 | 1.90E+01 | 14 | x | x | x |
| 172.0399 | 0.24 | 4.04E+00 | 4.40E+00 | 4.49E+00 | 4.72E+00 | 4.48E+00 | 3.87E+00 | 4.68E+00 | 3.97E+00 | 5.92E+00 | 5.06E+00 | 7.45E+00 | 4.13E+00 | 4.52E+00 | 2.97E+00 | 4.84E+00 | 5.12E+00 | 3.30E+00 | 22.04 |  |  | x |
| 158.0926 | 0.24 | 3.87E+01 | 3.57E+01 | 3.90E+01 | 4.04E+01 | 4.29E+01 | 3.95E+01 | 4.07E+01 | 3.64E+01 | 3.87E+01 | 4.20E+01 | 7.09E+01 | 3.32E+01 | 4.43E+01 | 4.46E+01 | 4.51E+01 | 4.06E+01 | 4.16E+01 | 19.27 |  | x | x |
| 181.0639 | 0.24 | 5.19E+01 | 4.45E+01 | 4.97E+01 | 5.27E+01 | 4.37E+01 | 5.74E+01 | 4.75E+01 | 4.47E+01 | 4.76E+01 | 4.42E+01 | 7.32E+01 | 4.70E+01 | 4.11E+01 | 4.20E+01 | 4.17E+01 | 4.20E+01 | 4.48E+01 | 16.4 |  | x | x |
| 185.0709 | 0.24 | 4.88E+01 | 4.35E+01 | 4.87E+01 | 4.93E+01 | 4.67E+01 | 5.67E+01 | 4.83E+01 | 4.39E+01 | 5.39E+01 | 4.66E+01 | 7.85E+01 | 3.88E+01 | 4.56E+01 | 4.67E+01 | 4.87E+01 | 4.48E+01 | 4.98E+01 | 17.24 |  | x | x |
| 185.1064 | 0.24 | 5.99E+00 | 5.48E+00 | 6.46E+00 | 3.86E+00 | 5.74E+00 | 6.52E+00 | 3.83E+00 | 5.65E+00 | 6.06E+00 | 4.70E+00 | 1.06E+01 | 5.05E+00 | 5.72E+00 | 5.15E+00 | 5.91E+00 | 5.36E+00 | 4.23E+00 | 26.68 |  |  | x |
| 171.0935 | 0.24 | 1.79E+02 | 1.57E+02 | 1.80E+02 | 1.73E+02 | 1.79E+02 | 1.88E+02 | 1.76E+02 | 1.75E+02 | 1.86E+02 | 1.59E+02 | 2.73E+02 | 1.41E+02 | 1.66E+02 | 1.67E+02 | 1.64E+02 | 1.51E+02 | 1.73E+02 | 15.95 |  | x | x |
| 199.0836 | 0.24 | 2.02E+02 | 1.85E+02 | 2.10E+02 | 1.92E+02 | 1.99E+02 | 2.05E+02 | 1.94E+02 | 1.89E+02 | 2.08E+02 | 1.85E+02 | 3.34E+02 | 1.59E+02 | 1.96E+02 | 1.95E+02 | 1.87E+02 | 1.94E+02 | 2.01E+02 | 17.82 |  | x | x |
| 203.0818 | 0.24 | 9.52E+00 | 1.01E+01 | 1.01E+01 | 1.28E+01 | 1.21E+01 | 1.34E+01 | 1.05E+01 | 1.33E+01 | 1.18E+01 | 1.40E+01 | 2.29E+01 | 8.42E+00 | 1.33E+01 | 1.27E+01 | 1.23E+01 | 1.46E+01 | 1.05E+01 | 25.52 |  |  | x |
| 178.1222 | 0.24 | 1.04E+01 | 1.34E+01 | 9.87E+00 | 1.30E+01 | 1.05E+01 | 1.17E+01 | 1.29E+01 | 1.41E+01 | 1.47E+01 | 1.24E+01 | 2.15E+01 | 1.33E+01 | 1.63E+01 | 1.36E+01 | 1.29E+01 | 9.84E+00 | 1.87E+01 | 22.82 |  |  | x |
| 231.0805 | 0.24 | 2.77E+01 | 3.01E+01 | 3.17E+01 | 2.78E+01 | 3.09E+01 | 3.33E+01 | 3.28E+01 | 3.49E+01 | 3.42E+01 | 2.63E+01 | 5.18E+01 | 3.64E+01 | 2.69E+01 | 3.00E+01 | 3.02E+01 | 2.69E+01 | 3.43E+01 | 18.47 |  | x | x |
| 255.0665 | 0.24 | 2.36E+03 | 2.05E+03 | 2.13E+03 | 2.28E+03 | 2.21E+03 | 2.32E+03 | 2.27E+03 | 2.23E+03 | 2.37E+03 | 2.11E+03 | 3.58E+03 | 2.31E+03 | 2.13E+03 | 2.02E+03 | 2.15E+03 | 1.94E+03 | 2.17E+03 | 15.75 |  | x | x |
| 255.1437 | 0.24 | 5.18E+01 | 4.35E+01 | 4.87E+01 | 4.93E+01 | 4.19E+01 | 4.79E+01 | 4.65E+01 | 4.00E+01 | 4.14E+01 | 3.77E+01 | 6.95E+01 | 4.07E+01 | 3.68E+01 | 3.62E+01 | 3.94E+01 | 3.84E+01 | 4.03E+01 | 18.27 |  | x | x |
| 295.1495 | 0.24 | 1.65E+01 | 1.34E+01 | 1.51E+01 | 1.49E+01 | 1.24E+01 | 1.37E+01 | 1.14E+01 | 1.51E+01 | 1.02E+01 | 1.45E+01 | 1.71E+01 | 8.80E+00 | 1.01E+01 | 1.44E+01 | 8.36E+00 | 1.29E+01 | 1.19E+01 | 19.83 |  | x | x |
| 314.2318 | 0.24 | 2.26E+02 | 2.01E+02 | 2.29E+02 | 2.32E+02 | 2.42E+02 | 2.55E+02 | 2.39E+02 | 2.49E+02 | 2.58E+02 | 2.19E+02 | 3.82E+02 | 1.41E+02 | 2.39E+02 | 2.34E+02 | 2.25E+02 | 2.41E+02 | 2.39E+02 | 19.08 |  | x | x |
| 316.9860 | 0.24 | 6.97E+01 | 6.18E+01 | 7.28E+01 | 8.32E+01 | 8.22E+01 | 8.09E+01 | 7.95E+01 | 7.48E+01 | 7.32E+01 | 5.08E+01 | 9.70E+01 | 9.05E+01 | 5.58E+01 | 5.06E+01 | 5.61E+01 | 4.48E+01 | 4.31E+01 | 23.76 |  |  | x |
| 318.9850 | 0.24 | 2.70E+01 | 2.26E+01 | 2.67E+01 | 3.00E+01 | 3.02E+01 | 3.13E+01 | 2.56E+01 | 3.04E+01 | 2.78E+01 | 1.70E+01 | 3.52E+01 | 3.23E+01 | 2.13E+01 | 1.65E+01 | 2.02E+01 | 1.58E+01 | 1.43E+01 | 25.96 |  |  | x |
| 428.0121 | 0.24 | 4.70E+02 | 4.07E+02 | 3.92E+02 | 3.65E+02 | 3.58E+02 | 3.45E+02 | 3.53E+02 | 3.01E+02 | 3.64E+02 | 3.03E+02 | 4.89E+02 | 3.06E+02 | 3.22E+02 | 2.98E+02 | 2.59E+02 | 3.11E+02 | 2.84E+02 | 18.07 |  | x | x |
| 430.0122 | 0.24 | 3.53E+02 | 3.17E+02 | 3.05E+02 | 2.75E+02 | 2.71E+02 | 2.68E+02 | 2.47E+02 | 2.23E+02 | 2.74E+02 | 2.11E+02 | 3.33E+02 | 2.17E+02 | 2.35E+02 | 2.16E+02 | 1.88E+02 | 2.28E+02 | 2.06E+02 | 18.8 |  | x | x |
| 152.1083 | 0.25 | 2.33E+01 | 2.21E+01 | 2.58E+01 | 2.54E+01 | 2.42E+01 | 2.79E+01 | 2.78E+01 | 2.80E+01 | 3.18E+01 | 2.50E+01 | 4.15E+01 | 2.03E+01 | 2.86E+01 | 2.73E+01 | 2.52E+01 | 2.93E+01 | 2.74E+01 | 17.15 |  | x | x |
| 164.1072 | 0.25 | 2.20E+01 | 2.27E+01 | 1.96E+01 | 2.30E+01 | 2.69E+01 | 2.60E+01 | 2.28E+01 | 2.97E+01 | 2.56E+01 | 2.81E+01 | 4.40E+01 | 1.83E+01 | 2.64E+01 | 2.61E+01 | 3.23E+01 | 2.64E+01 | 2.71E+01 | 21.91 |  |  | x |
| 167.1175 | 0.25 | 6.89E+01 | 6.19E+01 | 6.99E+01 | 7.13E+01 | 7.14E+01 | 7.70E+01 | 7.78E+01 | 8.28E+01 | 8.39E+01 | 7.33E+01 | 1.24E+02 | 7.37E+01 | 7.61E+01 | 7.81E+01 | 6.74E+01 | 7.26E+01 | 7.52E+01 | 17.35 |  | x | x |
| 219.0770 | 0.25 | 3.69E+01 | 3.32E+01 | 3.01E+01 | 3.25E+01 | 2.97E+01 | 3.29E+01 | 2.81E+01 | 3.30E+01 | 2.81E+01 | 2.20E+01 | 4.32E+01 | 3.68E+01 | 3.14E+01 | 2.12E+01 | 2.48E+01 | 2.67E+01 | 2.69E+01 | 18.28 |  | x | x |
| 237.1574 | 0.25 | 1.85E+01 | 1.72E+01 | 1.89E+01 | 1.73E+01 | 2.06E+01 | 1.75E+01 | 1.68E+01 | 2.13E+01 | 1.78E+01 | 1.49E+01 | 2.58E+01 | 9.11E+00 | 1.43E+01 | 1.50E+01 | 1.36E+01 | 1.54E+01 | 1.31E+01 | 22.08 |  |  | x |
| 312.2160 | 0.25 | 1.87E+02 | 1.68E+02 | 2.01E+02 | 2.13E+02 | 2.30E+02 | 2.38E+02 | 2.32E+02 | 2.23E+02 | 2.16E+02 | 2.02E+02 | 3.36E+02 | 1.25E+02 | 2.04E+02 | 2.24E+02 | 2.13E+02 | 2.12E+02 | 2.20E+02 | 19.33 |  | x | x |
| 310.2010 | 0.25 | 2.11E+02 | 1.90E+02 | 2.17E+02 | 2.14E+02 | 2.45E+02 | 2.54E+02 | 2.26E+02 | 2.39E+02 | 2.49E+02 | 2.26E+02 | 3.80E+02 | 8.86E+01 | 2.28E+02 | 2.31E+02 | 2.14E+02 | 2.23E+02 | 2.26E+02 | 23.7 |  |  | x |
| 437.2356 | 0.25 | 1.81E+02 | 1.57E+02 | 1.55E+02 | 1.30E+02 | 1.52E+02 | 1.44E+02 | 1.30E+02 | 1.38E+02 | 1.47E+02 | 1.42E+02 | 2.18E+02 | 8.64E+01 | 1.54E+02 | 1.55E+02 | 1.14E+02 | 1.48E+02 | 1.42E+02 | 18.68 |  | x | x |
| 130.0650 | 0.26 | 1.20E+01 | 1.05E+01 | 1.48E+01 | 1.38E+01 | 1.17E+01 | 1.49E+01 | 1.36E+01 | 1.38E+01 | 1.35E+01 | 1.32E+01 | 2.61E+01 | 1.09E+01 | 1.57E+01 | 1.39E+01 | 1.47E+01 | 1.45E+01 | 1.28E+01 | 24.09 |  |  | x |
| 232.1603 | 0.26 | 4.71E+00 | 3.34E+00 | 4.66E+00 | 4.02E+00 | 6.67E+00 | 5.18E+00 | 7.86E+00 | 4.97E+00 | 5.91E+00 | 4.55E+00 | 8.28E+00 | 5.88E+00 | 6.70E+00 | 7.23E+00 | 4.75E+00 | 7.38E+00 | 7.83E+00 | 25.53 |  |  | x |
| 217.1052 | 0.27 | 1.75E+02 | 1.93E+02 | 2.12E+02 | 1.97E+02 | 2.15E+02 | 2.24E+02 | 2.31E+02 | 2.18E+02 | 2.35E+02 | 2.04E+02 | 3.45E+02 | 2.40E+02 | 2.28E+02 | 2.39E+02 | 2.21E+02 | 2.19E+02 | 2.29E+02 | 15.73 |  | x | x |
| 349.1831 | 0.27 | 4.40E+02 | 4.96E+02 | 4.83E+02 | 4.31E+02 | 4.69E+02 | 5.27E+02 | 4.98E+02 | 4.51E+02 | 4.97E+02 | 4.57E+02 | 7.45E+02 | 3.87E+02 | 5.19E+02 | 5.24E+02 | 4.39E+02 | 4.89E+02 | 4.96E+02 | 15.36 |  | x | x |
| 393.2090 | 0.27 | 3.16E+02 | 3.05E+02 | 3.06E+02 | 2.70E+02 | 3.00E+02 | 3.28E+02 | 2.81E+02 | 2.80E+02 | 3.19E+02 | 2.97E+02 | 4.68E+02 | 2.18E+02 | 3.27E+02 | 3.19E+02 | 2.70E+02 | 3.05E+02 | 3.03E+02 | 16.15 |  | x | x |
| 261.1312 | 0.28 | 2.90E+02 | 5.33E+02 | 4.66E+02 | 3.71E+02 | 4.35E+02 | 4.82E+02 | 4.43E+02 | 4.71E+02 | 4.25E+02 | 4.38E+02 | 6.91E+02 | 4.28E+02 | 4.97E+02 | 5.06E+02 | 4.45E+02 | 4.45E+02 | 4.81E+02 | 17.48 |  | x | x |
| 305.1570 | 0.28 | 6.70E+02 | 1.08E+03 | 9.80E+02 | 7.98E+02 | 8.94E+02 | 9.95E+02 | 9.40E+02 | 9.77E+02 | 8.99E+02 | 8.61E+02 | 1.47E+03 | 8.78E+02 | 9.52E+02 | 9.81E+02 | 8.67E+02 | 9.00E+02 | 9.56E+02 | 17.21 |  | x | x |
| 365.1569 | 0.28 | 2.36E+02 | 3.47E+02 | 3.53E+02 | 2.91E+02 | 2.99E+02 | 2.98E+02 | 3.11E+02 | 4.29E+02 | 2.99E+02 | 2.39E+02 | 4.81E+02 | 3.16E+02 | 2.78E+02 | 2.70E+02 | 2.76E+02 | 2.57E+02 | 2.98E+02 | 20.46 |  |  | x |
| 409.1831 | 0.28 | 2.18E+02 | 2.81E+02 | 2.97E+02 | 2.32E+02 | 2.54E+02 | 2.34E+02 | 2.52E+02 | 3.52E+02 | 2.19E+02 | 1.96E+02 | 3.60E+02 | 2.42E+02 | 2.19E+02 | 1.96E+02 | 2.18E+02 | 2.02E+02 | 2.29E+02 | 19.86 |  | x | x |
| 453.2096 | 0.28 | 1.35E+02 | 1.51E+02 | 1.58E+02 | 1.29E+02 | 1.33E+02 | 1.04E+02 | 1.25E+02 | 1.90E+02 | 1.25E+02 | 8.99E+01 | 1.76E+02 | 1.13E+02 | 1.11E+02 | 1.08E+02 | 1.09E+02 | 1.06E+02 | 1.17E+02 | 21.03 |  |  | x |
| 148.1319 | 0.29 | 7.49E+01 | 7.07E+01 | 7.67E+01 | 7.85E+01 | 8.10E+01 | 8.84E+01 | 7.80E+01 | 8.93E+01 | 8.83E+01 | 7.25E+01 | 1.37E+02 | 1.15E+02 | 8.01E+01 | 7.55E+01 | 8.14E+01 | 7.54E+01 | 8.73E+01 | 19.63 |  | x | x |
| 432.2808 | 0.29 | 1.66E+02 | 1.61E+02 | 1.64E+02 | 1.51E+02 | 1.51E+02 | 1.74E+02 | 1.55E+02 | 9.36E+01 | 1.56E+02 | 1.61E+02 | 2.38E+02 | 1.21E+02 | 1.57E+02 | 1.45E+02 | 1.50E+02 | 1.48E+02 | 1.45E+02 | 18.24 |  | x | x |
| 132.1016 | 0.30 | 5.88E+01 | 5.76E+01 | 6.13E+01 | 6.52E+01 | 6.71E+01 | 7.15E+01 | 7.11E+01 | 6.61E+01 | 7.10E+01 | 6.36E+01 | 1.16E+02 | 9.44E+01 | 6.34E+01 | 5.86E+01 | 5.62E+01 | 6.46E+01 | 6.50E+01 | 21.61 |  |  | x |
| 150.0912 | 0.30 | 4.77E+01 | 3.86E+01 | 4.21E+01 | 4.71E+01 | 4.79E+01 | 4.86E+01 | 5.15E+01 | 4.89E+01 | 5.07E+01 | 4.50E+01 | 7.99E+01 | 5.32E+01 | 4.26E+01 | 4.07E+01 | 4.45E+01 | 4.76E+01 | 4.83E+01 | 18.52 |  | x | x |
| 283.1743 | 0.30 | 2.73E+01 | 4.11E+01 | 3.71E+01 | 3.62E+01 | 4.50E+01 | 5.04E+01 | 4.11E+01 | 4.28E+01 | 4.00E+01 | 4.43E+01 | 6.62E+01 | 3.49E+01 | 4.42E+01 | 4.61E+01 | 4.61E+01 | 4.16E+01 | 3.61E+01 | 19.3 |  | x | x |
| 256.0683 | 0.30 | 2.75E+02 | 2.35E+02 | 2.79E+02 | 2.75E+02 | 2.67E+02 | 2.76E+02 | 2.87E+02 | 2.57E+02 | 2.90E+02 | 2.32E+02 | 4.00E+02 | 4.22E+02 | 2.43E+02 | 2.26E+02 | 2.13E+02 | 2.33E+02 | 2.46E+02 | 20.72 |  |  | x |
| 104.1069 | 0.31 | 2.34E+02 | 2.08E+02 | 2.23E+02 | 2.30E+02 | 2.42E+02 | 2.70E+02 | 2.62E+02 | 2.67E+02 | 2.82E+02 | 2.47E+02 | 4.31E+02 | 2.83E+02 | 2.62E+02 | 2.59E+02 | 2.48E+02 | 2.69E+02 | 2.90E+02 | 18.16 |  | x | x |
| 134.0960 | 0.31 | 1.76E+01 | 1.72E+01 | 1.79E+01 | 2.07E+01 | 1.79E+01 | 2.01E+01 | 1.89E+01 | 1.69E+01 | 2.15E+01 | 1.45E+01 | 2.94E+01 | 2.10E+01 | 2.01E+01 | 1.71E+01 | 1.76E+01 | 1.61E+01 | 1.59E+01 | 17.73 |  | x | x |
| 265.1113 | 0.31 | 1.18E+02 | 1.06E+02 | 1.16E+02 | 1.20E+02 | 1.24E+02 | 1.31E+02 | 1.31E+02 | 1.15E+02 | 1.37E+02 | 1.11E+02 | 1.97E+02 | 1.23E+02 | 1.20E+02 | 1.16E+02 | 1.17E+02 | 1.18E+02 | 1.18E+02 | 16.12 |  | x | x |
| 327.2011 | 0.31 | 6.91E+01 | 8.75E+01 | 8.66E+01 | 9.30E+01 | 9.19E+01 | 1.16E+02 | 9.42E+01 | 7.63E+01 | 9.48E+01 | 1.02E+02 | 1.65E+02 | 9.97E+01 | 1.00E+02 | 1.06E+02 | 9.91E+01 | 1.06E+02 | 1.03E+02 | 20.44 |  |  | x |
| 371.2272 | 0.31 | 1.66E+02 | 1.69E+02 | 1.86E+02 | 1.80E+02 | 1.76E+02 | 2.12E+02 | 1.91E+02 | 1.49E+02 | 1.97E+02 | 1.92E+02 | 3.13E+02 | 1.72E+02 | 1.96E+02 | 1.96E+02 | 1.91E+02 | 1.97E+02 | 2.06E+02 | 17.95 |  | x | x |
| 146.1175 | 0.32 | 8.31E+00 | 7.58E+00 | 8.90E+00 | 1.26E+01 | 1.19E+01 | 1.36E+01 | 1.44E+01 | 1.41E+01 | 1.08E+01 | 1.29E+01 | 1.94E+01 | 1.40E+01 | 1.40E+01 | 1.42E+01 | 1.45E+01 | 1.46E+01 | 1.35E+01 | 21.92 |  |  | x |
| 152.0800 | 0.32 | 2.07E+01 | 1.80E+01 | 1.75E+01 | 2.20E+01 | 2.23E+01 | 2.45E+01 | 2.47E+01 | 2.18E+01 | 2.35E+01 | 2.24E+01 | 3.82E+01 | 2.55E+01 | 2.34E+01 | 2.03E+01 | 1.62E+01 | 2.13E+01 | 2.43E+01 | 20.98 |  |  | x |
| 180.1012 | 0.32 | 3.71E+00 | 5.02E+00 | 6.59E+00 | 3.49E+00 | 5.79E+00 | 5.69E+00 | 4.70E+00 | 5.93E+00 | 5.17E+00 | 6.45E+00 | 9.46E+00 | 6.89E+00 | 5.30E+00 | 5.39E+00 | 4.76E+00 | 6.17E+00 | 7.88E+00 | 25.05 |  |  | x |
| 192.1582 | 0.32 | 5.43E+00 | 5.31E+00 | 7.51E+00 | 7.22E+00 | 7.69E+00 | 8.31E+00 | 6.75E+00 | 9.41E+00 | 9.45E+00 | 7.19E+00 | 1.33E+01 | 8.39E+00 | 7.88E+00 | 8.18E+00 | 7.47E+00 | 8.61E+00 | 1.04E+01 | 22.89 |  |  | x |
| 211.1429 | 0.32 | 7.43E+00 | 7.63E+00 | 6.37E+00 | 9.18E+00 | 6.80E+00 | 7.78E+00 | 8.56E+00 | 7.83E+00 | 8.90E+00 | 7.58E+00 | 1.45E+01 | 6.78E+00 | 7.37E+00 | 4.63E+00 | 4.44E+00 | 9.42E+00 | 7.04E+00 | 28.23 |  |  | x |
| 239.1384 | 0.32 | 4.25E+01 | 3.93E+01 | 3.84E+01 | 4.88E+01 | 4.18E+01 | 4.66E+01 | 5.07E+01 | 4.66E+01 | 5.58E+01 | 4.07E+01 | 6.97E+01 | 4.63E+01 | 4.39E+01 | 3.97E+01 | 4.62E+01 | 4.73E+01 | 4.36E+01 | 16.18 |  | x | x |
| 253.1536 | 0.32 | 3.37E+01 | 3.08E+01 | 3.18E+01 | 3.59E+01 | 3.05E+01 | 3.28E+01 | 3.39E+01 | 3.04E+01 | 3.63E+01 | 2.51E+01 | 4.86E+01 | 3.72E+01 | 2.87E+01 | 2.29E+01 | 2.24E+01 | 2.64E+01 | 3.02E+01 | 19.63 |  | x | x |
| 476.3070 | 0.32 | 7.14E+01 | 5.63E+01 | 7.70E+01 | 6.08E+01 | 6.14E+01 | 6.41E+01 | 6.29E+01 | 4.43E+01 | 6.29E+01 | 5.57E+01 | 8.09E+01 | 3.91E+01 | 4.69E+01 | 5.72E+01 | 4.26E+01 | 4.26E+01 | 5.31E+01 | 21.01 |  |  | x |
| 118.0647 | 0.33 | 1.06E+01 | 9.40E+00 | 1.29E+01 | 1.36E+01 | 1.07E+01 | 1.23E+01 | 1.68E+01 | 1.19E+01 | 1.52E+01 | 1.24E+01 | 2.37E+01 | 2.08E+01 | 1.18E+01 | 1.18E+01 | 1.13E+01 | 1.37E+01 | 1.33E+01 | 27.09 |  |  | x |
| 160.1332 | 0.33 | 7.33E+01 | 6.71E+01 | 6.71E+01 | 8.18E+01 | 8.72E+01 | 9.16E+01 | 9.35E+01 | 9.06E+01 | 9.42E+01 | 7.47E+01 | 1.34E+02 | 1.09E+02 | 8.45E+01 | 8.66E+01 | 7.99E+01 | 8.29E+01 | 9.11E+01 | 18.17 |  | x | x |
| 356.2417 | 0.33 | 1.33E+01 | 7.82E+00 | 1.15E+01 | 1.11E+01 | 1.07E+01 | 1.34E+01 | 9.98E+00 | 1.36E+01 | 9.13E+00 | 1.18E+01 | 1.40E+01 | 5.12E+00 | 7.14E+00 | 9.10E+00 | 7.11E+00 | 1.69E+01 | 1.24E+01 | 28.02 |  |  | x |
| 174.0549 | 0.34 | 1.30E+02 | 1.19E+02 | 1.30E+02 | 1.34E+02 | 1.36E+02 | 1.47E+02 | 1.48E+02 | 1.33E+02 | 1.46E+02 | 1.26E+02 | 2.27E+02 | 1.73E+02 | 1.41E+02 | 1.40E+02 | 1.18E+02 | 1.39E+02 | 1.45E+02 | 17.55 |  | x | x |
| 124.0395 | 0.35 | 1.04E+02 | 8.88E+01 | 9.94E+01 | 1.05E+02 | 1.08E+02 | 1.25E+02 | 1.26E+02 | 1.17E+02 | 1.29E+02 | 1.07E+02 | 1.93E+02 | 1.34E+02 | 1.12E+02 | 1.05E+02 | 9.93E+01 | 1.09E+02 | 1.22E+02 | 19.87 |  | x | x |
| 130.0649 | 0.35 | 6.34E+02 | 5.71E+02 | 6.27E+02 | 6.59E+02 | 7.16E+02 | 7.73E+02 | 7.11E+02 | 6.85E+02 | 7.55E+02 | 6.95E+02 | 1.14E+03 | 7.03E+02 | 7.23E+02 | 7.43E+02 | 7.08E+02 | 7.23E+02 | 7.39E+02 | 16.47 |  | x | x |
| 155.0806 | 0.35 | 9.27E+00 | 9.20E+00 | 1.04E+01 | 8.53E+00 | 1.02E+01 | 1.07E+01 | 1.18E+01 | 9.35E+00 | 1.28E+01 | 1.06E+01 | 1.66E+01 | 9.09E+00 | 1.37E+01 | 1.06E+01 | 1.08E+01 | 1.23E+01 | 1.25E+01 | 18.42 |  | x | x |
| 205.0608 | 0.35 | 1.13E+02 | 1.01E+02 | 1.14E+02 | 1.16E+02 | 1.19E+02 | 1.20E+02 | 1.27E+02 | 1.16E+02 | 1.28E+02 | 1.09E+02 | 1.93E+02 | 1.30E+02 | 1.14E+02 | 1.18E+02 | 1.04E+02 | 1.07E+02 | 1.15E+02 | 16.92 |  | x | x |
| 122.0861 | 0.42 | 1.39E+01 | 8.18E+00 | 1.04E+01 | 1.43E+01 | 1.28E+01 | 1.36E+01 | 1.39E+01 | 1.56E+01 | 1.27E+01 | 1.43E+01 | 2.33E+01 | 1.05E+01 | 1.52E+01 | 1.37E+01 | 1.43E+01 | 1.53E+01 | 1.49E+01 | 22.35 |  |  | x |
| 125.1073 | 0.42 | 4.20E+01 | 3.84E+01 | 3.52E+01 | 4.16E+01 | 4.66E+01 | 5.39E+01 | 5.04E+01 | 5.10E+01 | 5.29E+01 | 4.60E+01 | 8.63E+01 | 2.07E+01 | 5.26E+01 | 5.50E+01 | 5.08E+01 | 5.52E+01 | 5.63E+01 | 26.93 |  |  | x |
| 171.1125 | 0.45 | 1.04E+01 | 8.49E+00 | 9.71E+00 | 1.03E+01 | 1.07E+01 | 1.28E+01 | 9.89E+00 | 1.14E+01 | 1.06E+01 | 1.05E+01 | 1.92E+01 | 2.75E+00 | 1.22E+01 | 1.27E+01 | 1.07E+01 | 1.14E+01 | 1.29E+01 | 28.54 |  |  | x |
| 297.0986 | 0.45 | 2.78E+01 | 2.33E+01 | 2.59E+01 | 2.59E+01 | 2.52E+01 | 2.82E+01 | 2.78E+01 | 2.71E+01 | 2.85E+01 | 2.33E+01 | 4.00E+01 | 8.53E-01 | 2.63E+01 | 2.48E+01 | 2.39E+01 | 2.49E+01 | 2.55E+01 | 29.06 |  |  | x |
| 162.0553 | 0.46 | 1.34E+03 | 1.21E+03 | 1.33E+03 | 1.39E+03 | 1.50E+03 | 1.65E+03 | 1.58E+03 | 1.53E+03 | 1.72E+03 | 1.58E+03 | 2.68E+03 | 1.75E+03 | 1.72E+03 | 1.81E+03 | 1.68E+03 | 1.73E+03 | 1.79E+03 | 19.36 |  | x | x |
| 162.1631 | 0.46 | 1.90E+01 | 1.80E+01 | 2.06E+01 | 2.05E+01 | 2.18E+01 | 2.28E+01 | 2.20E+01 | 2.11E+01 | 2.63E+01 | 2.22E+01 | 4.15E+01 | 2.67E+01 | 2.65E+01 | 2.81E+01 | 2.50E+01 | 2.73E+01 | 2.88E+01 | 22.26 |  |  | x |
| 174.1235 | 0.46 | 4.39E+02 | 3.99E+02 | 5.01E+02 | 4.76E+02 | 5.31E+02 | 5.95E+02 | 5.90E+02 | 5.73E+02 | 6.14E+02 | 4.97E+02 | 8.32E+02 | 7.20E+02 | 4.84E+02 | 4.42E+02 | 4.41E+02 | 4.56E+02 | 5.22E+02 | 20.7 |  |  | x |
| 211.1074 | 0.46 | 3.93E+02 | 3.64E+02 | 4.01E+02 | 4.19E+02 | 4.39E+02 | 4.53E+02 | 4.36E+02 | 4.09E+02 | 4.53E+02 | 4.00E+02 | 6.82E+02 | 4.14E+02 | 4.17E+02 | 4.26E+02 | 3.97E+02 | 4.22E+02 | 4.21E+02 | 15.76 |  | x | x |
| 146.0601 | 0.48 | 2.60E+02 | 2.45E+02 | 2.77E+02 | 2.78E+02 | 3.00E+02 | 3.13E+02 | 3.08E+02 | 3.03E+02 | 3.34E+02 | 2.97E+02 | 5.34E+02 | 3.07E+02 | 3.22E+02 | 3.37E+02 | 3.00E+02 | 3.15E+02 | 3.33E+02 | 19.55 |  | x | x |
| 250.9173 | 0.48 | 5.19E+01 | 3.93E+01 | 4.09E+01 | 3.22E+01 | 4.16E+01 | 4.57E+01 | 4.25E+01 | 4.05E+01 | 4.64E+01 | 4.63E+01 | 7.88E+01 | 1.81E+01 | 4.66E+01 | 5.35E+01 | 4.52E+01 | 5.18E+01 | 5.06E+01 | 26.52 |  |  | x |
| 264.9334 | 0.48 | 4.53E+01 | 3.77E+01 | 4.33E+01 | 3.31E+01 | 3.89E+01 | 4.06E+01 | 4.15E+01 | 4.14E+01 | 4.50E+01 | 4.21E+01 | 7.43E+01 | 2.07E+01 | 5.09E+01 | 4.79E+01 | 4.32E+01 | 5.57E+01 | 4.93E+01 | 24.69 |  |  | x |
| 278.9488 | 0.48 | 3.02E+02 | 2.56E+02 | 2.61E+02 | 2.50E+02 | 2.71E+02 | 2.97E+02 | 2.74E+02 | 2.88E+02 | 3.22E+02 | 2.81E+02 | 4.93E+02 | 2.31E+02 | 3.13E+02 | 3.33E+02 | 2.77E+02 | 3.12E+02 | 3.21E+02 | 19.21 |  | x | x |
| 309.1539 | 0.48 | 4.04E+01 | 3.61E+01 | 3.85E+01 | 3.69E+01 | 4.49E+01 | 4.34E+01 | 3.75E+01 | 4.16E+01 | 4.18E+01 | 3.81E+01 | 7.14E+01 | 8.08E+00 | 4.00E+01 | 4.43E+01 | 4.00E+01 | 4.16E+01 | 4.18E+01 | 28.44 |  |  | x |
| 490.9035 | 0.48 | 6.12E+01 | 4.77E+01 | 4.80E+01 | 3.94E+01 | 3.85E+01 | 4.81E+01 | 3.76E+01 | 3.37E+01 | 3.71E+01 | 4.05E+01 | 5.83E+01 | 8.05E+00 | 4.41E+01 | 4.43E+01 | 3.41E+01 | 4.55E+01 | 3.57E+01 | 28.02 |  |  | x |
| 265.1316 | 0.50 | 6.05E+00 | 4.87E+00 | 6.54E+00 | 6.06E+00 | 5.25E+00 | 5.39E+00 | 7.01E+00 | 4.06E+00 | 5.85E+00 | 2.61E+00 | 5.64E+00 | 7.16E+00 | 6.85E+00 | 4.40E+00 | 3.61E+00 | 4.74E+00 | 3.01E+00 | 26.15 |  |  | x |
| 280.9328 | 0.50 | 1.23E+02 | 9.05E+01 | 9.97E+01 | 1.01E+02 | 1.01E+02 | 1.13E+02 | 1.10E+02 | 1.06E+02 | 1.21E+02 | 1.01E+02 | 1.64E+02 | 4.80E+01 | 1.06E+02 | 1.01E+02 | 9.42E+01 | 1.07E+02 | 1.10E+02 | 20.89 |  |  | x |
| 335.0999 | 0.50 | 1.07E+02 | 1.13E+02 | 1.35E+02 | 1.29E+02 | 1.33E+02 | 1.34E+02 | 1.43E+02 | 1.41E+02 | 1.46E+02 | 1.31E+02 | 2.46E+02 | 3.86E+01 | 1.44E+02 | 1.51E+02 | 1.44E+02 | 1.55E+02 | 1.44E+02 | 28.14 |  |  | x |
| 153.0656 | 0.51 | 3.68E+02 | 3.37E+02 | 3.65E+02 | 3.80E+02 | 3.96E+02 | 4.38E+02 | 4.18E+02 | 4.02E+02 | 4.69E+02 | 4.20E+02 | 7.31E+02 | 2.08E+02 | 4.56E+02 | 4.71E+02 | 4.44E+02 | 4.77E+02 | 4.69E+02 | 24.08 |  |  | x |
| 244.1095 | 0.51 | 3.32E+02 | 2.97E+02 | 3.43E+02 | 3.45E+02 | 3.19E+02 | 3.46E+02 | 3.64E+02 | 3.37E+02 | 3.67E+02 | 2.84E+02 | 5.36E+02 | 2.41E+02 | 2.96E+02 | 2.60E+02 | 2.58E+02 | 2.95E+02 | 2.96E+02 | 20.4 |  |  | x |
| 170.0603 | 0.52 | 4.36E+02 | 3.85E+02 | 4.34E+02 | 4.43E+02 | 4.48E+02 | 4.85E+02 | 4.69E+02 | 4.38E+02 | 4.85E+02 | 4.12E+02 | 7.36E+02 | 4.32E+02 | 4.26E+02 | 4.26E+02 | 3.91E+02 | 4.29E+02 | 4.37E+02 | 17.08 |  | x | x |
| 215.0797 | 0.53 | 1.00E+02 | 9.47E+01 | 1.05E+02 | 1.01E+02 | 1.06E+02 | 1.15E+02 | 1.07E+02 | 1.15E+02 | 1.18E+02 | 1.13E+02 | 1.73E+02 | 8.40E+01 | 1.21E+02 | 1.10E+02 | 1.16E+02 | 1.06E+02 | 1.19E+02 | 16.4 |  | x | x |
| 274.1172 | 0.53 | 8.24E+01 | 7.92E+01 | 9.19E+01 | 9.88E+01 | 9.90E+01 | 1.02E+02 | 1.09E+02 | 1.05E+02 | 1.16E+02 | 9.38E+01 | 1.67E+02 | 6.89E+01 | 1.08E+02 | 9.62E+01 | 9.66E+01 | 1.00E+02 | 1.09E+02 | 20.38 |  |  | x |
| 164.0700 | 0.54 | 3.58E+01 | 2.99E+01 | 3.66E+01 | 4.14E+01 | 3.49E+01 | 3.25E+01 | 3.49E+01 | 2.94E+01 | 3.78E+01 | 3.09E+01 | 5.33E+01 | 1.20E+01 | 2.99E+01 | 3.40E+01 | 3.04E+01 | 3.18E+01 | 2.88E+01 | 24.21 |  |  | x |
| 235.1077 | 0.54 | 2.73E+01 | 2.53E+01 | 3.23E+01 | 3.03E+01 | 3.44E+01 | 2.97E+01 | 2.99E+01 | 2.82E+01 | 3.06E+01 | 2.43E+01 | 3.45E+01 | 6.71E+00 | 2.85E+01 | 2.60E+01 | 2.17E+01 | 2.45E+01 | 2.66E+01 | 23.32 |  |  | x |
| 319.9382 | 0.54 | 9.56E+01 | 8.15E+01 | 8.54E+01 | 8.02E+01 | 8.15E+01 | 8.52E+01 | 8.47E+01 | 8.63E+01 | 9.08E+01 | 7.11E+01 | 1.32E+02 | 6.40E+00 | 7.58E+01 | 7.20E+01 | 7.82E+01 | 7.64E+01 | 8.05E+01 | 29.14 |  |  | x |
| 122.0825 | 0.56 | 2.08E+01 | 9.83E+00 | 1.45E+01 | 1.80E+01 | 1.66E+01 | 1.74E+01 | 1.51E+01 | 1.68E+01 | 1.47E+01 | 1.58E+01 | 2.87E+01 | 1.35E+01 | 1.54E+01 | 1.85E+01 | 1.40E+01 | 2.06E+01 | 1.60E+01 | 23.95 |  |  | x |
| 108.0440 | 0.57 | 4.42E+01 | 4.34E+01 | 4.81E+01 | 5.60E+01 | 6.12E+01 | 6.37E+01 | 6.07E+01 | 6.49E+01 | 6.52E+01 | 5.22E+01 | 1.04E+02 | 1.97E+01 | 6.63E+01 | 5.56E+01 | 6.69E+01 | 6.44E+01 | 6.62E+01 | 28.54 |  |  | x |
| 136.0395 | 0.57 | 4.73E+02 | 4.57E+02 | 5.06E+02 | 5.46E+02 | 6.07E+02 | 6.28E+02 | 6.19E+02 | 6.36E+02 | 6.58E+02 | 5.81E+02 | 1.03E+03 | 4.46E+02 | 6.40E+02 | 6.29E+02 | 6.74E+02 | 6.63E+02 | 6.77E+02 | 21.36 |  |  | x |
| 163.0859 | 0.57 | 6.61E+00 | 6.00E+00 | 8.15E+00 | 5.94E+00 | 9.65E+00 | 8.35E+00 | 1.07E+01 | 7.51E+00 | 1.19E+01 | 8.22E+00 | 9.72E+00 | 6.71E+00 | 9.21E+00 | 6.67E+00 | 8.01E+00 | 8.96E+00 | 7.29E+00 | 20.24 |  |  | x |
| 199.1072 | 0.57 | 6.41E+01 | 5.76E+01 | 6.89E+01 | 7.23E+01 | 7.33E+01 | 7.79E+01 | 8.09E+01 | 8.25E+01 | 9.01E+01 | 7.37E+01 | 1.32E+02 | 5.98E+01 | 8.26E+01 | 7.23E+01 | 7.62E+01 | 8.74E+01 | 7.89E+01 | 21.06 |  |  | x |
| 190.1225 | 0.58 | 1.14E+02 | 1.01E+02 | 1.17E+02 | 1.18E+02 | 1.29E+02 | 1.29E+02 | 1.27E+02 | 1.14E+02 | 1.25E+02 | 1.03E+02 | 1.76E+02 | 9.82E+01 | 1.11E+02 | 1.06E+02 | 1.04E+02 | 1.08E+02 | 1.03E+02 | 15.62 |  | x | x |
| 270.0794 | 0.58 | 1.89E+02 | 1.58E+02 | 1.93E+02 | 2.05E+02 | 2.03E+02 | 2.16E+02 | 2.07E+02 | 1.90E+02 | 2.11E+02 | 1.72E+02 | 3.08E+02 | 1.43E+02 | 1.80E+02 | 1.81E+02 | 1.52E+02 | 1.76E+02 | 1.76E+02 | 19.04 |  | x | x |
| 201.0651 | 0.61 | 2.26E+01 | 1.88E+01 | 2.36E+01 | 2.28E+01 | 2.41E+01 | 2.57E+01 | 2.55E+01 | 2.83E+01 | 2.40E+01 | 1.90E+01 | 4.24E+01 | 2.14E+01 | 2.49E+01 | 1.95E+01 | 1.98E+01 | 2.16E+01 | 2.34E+01 | 22.57 |  |  | x |
| 260.1023 | 0.61 | 2.45E+01 | 3.03E+01 | 3.50E+01 | 4.16E+01 | 3.70E+01 | 4.19E+01 | 4.41E+01 | 2.83E+01 | 4.32E+01 | 4.22E+01 | 7.33E+01 | 3.76E+01 | 4.32E+01 | 3.64E+01 | 4.10E+01 | 4.15E+01 | 4.64E+01 | 25.65 |  |  | x |
| 220.1180 | 0.62 | 2.20E+02 | 2.23E+02 | 2.50E+02 | 3.18E+02 | 3.51E+02 | 3.70E+02 | 3.37E+02 | 3.24E+02 | 3.73E+02 | 3.18E+02 | 5.61E+02 | 3.86E+02 | 3.60E+02 | 3.50E+02 | 3.75E+02 | 3.47E+02 | 3.51E+02 | 22.22 |  |  | x |
| 279.1297 | 0.62 | 3.99E+02 | 3.69E+02 | 4.38E+02 | 4.86E+02 | 5.18E+02 | 5.35E+02 | 5.30E+02 | 4.97E+02 | 5.36E+02 | 4.54E+02 | 7.99E+02 | 8.69E+01 | 4.73E+02 | 4.66E+02 | 4.56E+02 | 4.66E+02 | 4.81E+02 | 28.51 |  |  | x |
| 319.1258 | 0.62 | 1.58E+02 | 1.64E+02 | 1.76E+02 | 1.66E+02 | 2.28E+02 | 1.98E+02 | 1.92E+02 | 2.91E+02 | 1.98E+02 | 1.82E+02 | 3.00E+02 | 2.57E+02 | 2.02E+02 | 2.05E+02 | 1.71E+02 | 1.48E+02 | 1.89E+02 | 21.89 |  |  | x |
| 335.0998 | 0.63 | 7.29E+02 | 7.50E+02 | 8.80E+02 | 9.53E+02 | 1.09E+03 | 1.16E+03 | 1.15E+03 | 1.22E+03 | 1.19E+03 | 1.05E+03 | 1.82E+03 | 1.54E+03 | 1.13E+03 | 1.18E+03 | 1.03E+03 | 1.09E+03 | 1.19E+03 | 23.02 |  |  | x |
| 84.0806 | 0.65 | 6.01E+00 | 4.43E+00 | 4.19E+00 | 4.19E+00 | 9.05E+00 | 7.18E+00 | 6.56E+00 | 9.21E+00 | 6.68E+00 | 5.53E+00 | 8.47E+00 | 5.93E+00 | 6.14E+00 | 6.05E+00 | 4.49E+00 | 9.24E+00 | 6.02E+00 | 26.55 |  |  | x |
| 242.0998 | 0.65 | 5.97E+01 | 6.09E+01 | 6.54E+01 | 6.89E+01 | 9.31E+01 | 8.33E+01 | 7.41E+01 | 1.11E+02 | 8.53E+01 | 6.70E+01 | 1.24E+02 | 2.88E+01 | 8.32E+01 | 8.15E+01 | 7.18E+01 | 6.74E+01 | 8.58E+01 | 27.34 |  |  | x |
| 104.1070 | 0.68 | 2.00E+01 | 1.91E+01 | 2.26E+01 | 2.87E+01 | 3.15E+01 | 3.71E+01 | 2.80E+01 | 2.89E+01 | 3.39E+01 | 3.56E+01 | 5.29E+01 | 3.24E+01 | 3.45E+01 | 4.22E+01 | 3.61E+01 | 3.46E+01 | 3.69E+01 | 25.04 |  |  | x |
| 291.9268 | 0.68 | 1.35E+02 | 1.25E+02 | 1.30E+02 | 1.28E+02 | 1.32E+02 | 1.40E+02 | 1.26E+02 | 1.28E+02 | 1.41E+02 | 1.28E+02 | 2.18E+02 | 3.57E+01 | 1.44E+02 | 1.41E+02 | 1.35E+02 | 1.42E+02 | 1.45E+02 | 24.63 |  |  | x |
| 289.9284 | 0.69 | 1.18E+03 | 1.04E+03 | 1.14E+03 | 1.10E+03 | 1.22E+03 | 1.28E+03 | 1.22E+03 | 1.22E+03 | 1.29E+03 | 1.13E+03 | 1.93E+03 | 1.22E+03 | 1.20E+03 | 1.26E+03 | 1.16E+03 | 1.20E+03 | 1.24E+03 | 15.37 |  | x | x |
| 540.8952 | 0.69 | 1.60E+02 | 1.43E+02 | 1.35E+02 | 1.39E+02 | 1.40E+02 | 1.47E+02 | 1.43E+02 | 1.37E+02 | 1.28E+02 | 1.35E+02 | 1.97E+02 | 9.99E+01 | 1.37E+02 | 1.31E+02 | 1.10E+02 | 1.30E+02 | 1.23E+02 | 15.03 |  | x | x |
| 160.1330 | 0.72 | 9.85E+00 | 1.02E+01 | 1.24E+01 | 1.22E+01 | 1.36E+01 | 1.61E+01 | 1.50E+01 | 1.72E+01 | 1.55E+01 | 1.55E+01 | 2.23E+01 | 9.65E+00 | 1.49E+01 | 1.27E+01 | 1.65E+01 | 1.41E+01 | 2.10E+01 | 23.95 |  |  | x |
| 212.9831 | 0.73 | 9.55E+02 | 8.00E+02 | 8.68E+02 | 9.18E+02 | 9.33E+02 | 9.72E+02 | 9.42E+02 | 9.55E+02 | 1.01E+03 | 8.99E+02 | 1.52E+03 | 8.43E+02 | 9.62E+02 | 9.90E+02 | 8.90E+02 | 9.52E+02 | 9.78E+02 | 15.93 |  | x | x |
| 229.1549 | 0.73 | 4.46E+03 | 4.00E+03 | 4.61E+03 | 5.08E+03 | 5.32E+03 | 5.64E+03 | 5.53E+03 | 5.61E+03 | 5.73E+03 | 5.00E+03 | 8.77E+03 | 5.17E+03 | 5.43E+03 | 5.28E+03 | 5.28E+03 | 5.35E+03 | 5.61E+03 | 18.18 |  | x | x |
| 143.1179 | 0.74 | 2.03E+03 | 1.89E+03 | 2.11E+03 | 2.31E+03 | 2.43E+03 | 2.59E+03 | 2.58E+03 | 2.59E+03 | 2.66E+03 | 2.30E+03 | 4.05E+03 | 2.31E+03 | 2.47E+03 | 2.40E+03 | 2.43E+03 | 2.52E+03 | 2.64E+03 | 18.38 |  | x | x |
| 174.1237 | 0.74 | 1.16E+03 | 1.05E+03 | 1.21E+03 | 1.32E+03 | 1.33E+03 | 1.42E+03 | 1.44E+03 | 1.43E+03 | 1.47E+03 | 1.24E+03 | 2.25E+03 | 1.18E+03 | 1.33E+03 | 1.28E+03 | 1.27E+03 | 1.32E+03 | 1.40E+03 | 18.84 |  | x | x |
| 204.1234 | 0.74 | 5.68E+00 | 4.73E+00 | 8.07E+00 | 3.64E+00 | 8.08E+00 | 8.68E+00 | 8.79E+00 | 9.94E+00 | 7.34E+00 | 7.47E+00 | 1.41E+01 | 7.43E+00 | 8.60E+00 | 9.45E+00 | 8.15E+00 | 7.46E+00 | 5.83E+00 | 29.49 |  |  | x |
| 214.9818 | 0.74 | 3.80E+02 | 3.29E+02 | 3.51E+02 | 3.59E+02 | 3.66E+02 | 3.82E+02 | 3.69E+02 | 3.80E+02 | 3.91E+02 | 3.49E+02 | 5.91E+02 | 3.14E+02 | 3.70E+02 | 3.92E+02 | 3.53E+02 | 3.74E+02 | 3.84E+02 | 15.48 |  | x | x |
| 241.1545 | 0.74 | 6.36E+02 | 5.70E+02 | 6.37E+02 | 7.60E+02 | 7.52E+02 | 7.87E+02 | 7.79E+02 | 7.60E+02 | 7.64E+02 | 6.49E+02 | 1.19E+03 | 6.30E+02 | 7.15E+02 | 6.35E+02 | 7.00E+02 | 7.18E+02 | 7.50E+02 | 18.44 |  | x | x |
| 315.9309 | 0.74 | 6.30E+01 | 5.35E+01 | 5.36E+01 | 5.54E+01 | 4.74E+01 | 5.25E+01 | 4.87E+01 | 4.95E+01 | 5.22E+01 | 4.29E+01 | 7.60E+01 | 2.83E+01 | 3.77E+01 | 4.41E+01 | 3.76E+01 | 3.64E+01 | 2.78E+01 | 25.53 |  |  | x |
| 317.9299 | 0.74 | 2.05E+01 | 1.73E+01 | 1.92E+01 | 1.98E+01 | 1.93E+01 | 1.84E+01 | 2.07E+01 | 1.83E+01 | 1.67E+01 | 1.39E+01 | 2.24E+01 | 1.08E+01 | 1.28E+01 | 8.37E+00 | 1.13E+01 | 1.01E+01 | 1.09E+01 | 27.92 |  |  | x |
| 143.2200 | 0.75 | 2.38E+01 | 2.13E+01 | 2.41E+01 | 3.01E+01 | 2.84E+01 | 3.06E+01 | 3.03E+01 | 3.08E+01 | 3.34E+01 | 2.63E+01 | 5.13E+01 | 3.60E+01 | 3.02E+01 | 2.77E+01 | 3.01E+01 | 3.00E+01 | 2.91E+01 | 21.48 |  |  | x |
| 160.1330 | 0.77 | 1.86E+01 | 2.40E+01 | 2.42E+01 | 3.04E+01 | 3.88E+01 | 3.90E+01 | 2.76E+01 | 3.78E+01 | 3.25E+01 | 3.15E+01 | 5.67E+01 | 5.53E+01 | 3.89E+01 | 3.60E+01 | 5.38E+01 | 3.65E+01 | 3.97E+01 | 29.62 |  |  | x |
| 350.9339 | 0.83 | 1.27E+03 | 1.12E+03 | 1.20E+03 | 1.24E+03 | 1.29E+03 | 1.34E+03 | 1.30E+03 | 1.26E+03 | 1.35E+03 | 1.22E+03 | 2.07E+03 | 1.11E+03 | 1.29E+03 | 1.29E+03 | 1.20E+03 | 1.23E+03 | 1.26E+03 | 16.19 |  | x | x |
| 300.0844 | 0.90 | 2.53E+02 | 2.58E+02 | 2.85E+02 | 3.23E+02 | 3.48E+02 | 3.65E+02 | 3.63E+02 | 3.61E+02 | 3.62E+02 | 3.16E+02 | 5.62E+02 | 3.10E+02 | 3.46E+02 | 3.37E+02 | 3.10E+02 | 3.29E+02 | 3.53E+02 | 19.74 |  | x | x |
| 335.0213 | 0.94 | 8.44E+01 | 7.11E+01 | 8.09E+01 | 8.02E+01 | 8.55E+01 | 8.28E+01 | 8.39E+01 | 7.14E+01 | 9.09E+01 | 6.80E+01 | 1.26E+02 | 5.26E+01 | 7.66E+01 | 8.01E+01 | 6.91E+01 | 7.76E+01 | 7.84E+01 | 18.4 |  | x | x |
| 410.9342 | 0.95 | 1.51E+03 | 1.20E+03 | 1.30E+03 | 1.11E+03 | 1.08E+03 | 1.13E+03 | 1.08E+03 | 1.05E+03 | 1.14E+03 | 9.90E+02 | 1.67E+03 | 8.33E+02 | 1.05E+03 | 1.03E+03 | 9.20E+02 | 1.04E+03 | 1.01E+03 | 18.16 |  | x | x |
| 309.9191 | 0.96 | 6.43E+01 | 5.62E+01 | 6.36E+01 | 6.21E+01 | 6.47E+01 | 7.00E+01 | 6.52E+01 | 6.57E+01 | 6.74E+01 | 6.44E+01 | 9.91E+01 | 5.52E+01 | 7.01E+01 | 5.99E+01 | 5.68E+01 | 6.89E+01 | 6.55E+01 | 14.71 | x | x | x |
| 406.9965 | 0.97 | 1.00E+02 | 8.17E+01 | 8.16E+01 | 8.07E+01 | 6.63E+01 | 6.78E+01 | 7.16E+01 | 5.80E+01 | 6.73E+01 | 6.21E+01 | 9.71E+01 | 3.97E+01 | 6.21E+01 | 6.33E+01 | 5.54E+01 | 6.53E+01 | 5.42E+01 | 22.16 |  |  | x |
| 421.0124 | 0.97 | 5.25E+02 | 4.24E+02 | 4.31E+02 | 3.64E+02 | 3.84E+02 | 3.89E+02 | 3.63E+02 | 3.39E+02 | 3.77E+02 | 3.33E+02 | 5.88E+02 | 2.62E+02 | 3.34E+02 | 3.40E+02 | 3.05E+02 | 3.30E+02 | 3.23E+02 | 21.15 |  |  | x |
| 322.9392 | 0.98 | 9.22E+02 | 7.90E+02 | 8.64E+02 | 8.30E+02 | 8.73E+02 | 9.07E+02 | 8.73E+02 | 8.60E+02 | 9.01E+02 | 8.18E+02 | 1.37E+03 | 7.17E+02 | 8.77E+02 | 8.73E+02 | 7.89E+02 | 8.67E+02 | 8.58E+02 | 15.31 |  | x | x |
| 323.0941 | 0.98 | 9.15E+00 | 7.71E+00 | 6.15E+00 | 6.84E+00 | 6.16E+00 | 7.09E+00 | 8.31E+00 | 6.82E+00 | 6.63E+00 | 6.59E+00 | 1.18E+01 | 1.71E+00 | 9.70E+00 | 6.60E+00 | 8.81E+00 | 7.09E+00 | 8.99E+00 | 28.39 |  |  | x |
| 324.9371 | 0.98 | 1.25E+02 | 1.09E+02 | 1.16E+02 | 1.19E+02 | 1.24E+02 | 1.15E+02 | 1.17E+02 | 1.19E+02 | 1.17E+02 | 1.07E+02 | 1.88E+02 | 1.19E+02 | 1.13E+02 | 1.11E+02 | 9.86E+01 | 1.12E+02 | 1.18E+02 | 15.75 |  | x | x |
| 311.9269 | 0.99 | 1.27E+01 | 1.24E+01 | 1.57E+01 | 1.40E+01 | 1.84E+01 | 1.43E+01 | 1.40E+01 | 1.21E+01 | 1.25E+01 | 8.70E+00 | 1.87E+01 | 5.15E+00 | 1.51E+01 | 1.21E+01 | 9.04E+00 | 1.33E+01 | 9.02E+00 | 26.98 |  |  | x |
| 282.9079 | 1.00 | 3.93E+02 | 3.31E+02 | 3.61E+02 | 3.36E+02 | 3.66E+02 | 3.65E+02 | 3.59E+02 | 3.62E+02 | 3.73E+02 | 3.51E+02 | 6.11E+02 | 3.31E+02 | 3.78E+02 | 3.66E+02 | 3.33E+02 | 3.78E+02 | 3.63E+02 | 17.01 |  | x | x |
| 284.9065 | 1.00 | 5.23E+01 | 4.08E+01 | 4.37E+01 | 4.07E+01 | 4.66E+01 | 4.53E+01 | 4.04E+01 | 4.05E+01 | 4.81E+01 | 3.76E+01 | 7.14E+01 | 4.06E+01 | 4.43E+01 | 4.13E+01 | 3.69E+01 | 4.06E+01 | 4.66E+01 | 17.87 |  | x | x |
| 306.0148 | 1.01 | 2.00E+01 | 1.88E+01 | 1.99E+01 | 2.13E+01 | 2.60E+01 | 2.72E+01 | 1.46E+01 | 1.60E+01 | 1.54E+01 | 1.48E+01 | 2.38E+01 | 2.53E+01 | 1.50E+01 | 1.53E+01 | 1.32E+01 | 1.80E+01 | 1.77E+01 | 23.28 |  |  | x |
| 178.1588 | 1.02 | 8.29E+01 | 7.74E+01 | 8.68E+01 | 9.79E+01 | 1.03E+02 | 1.07E+02 | 9.97E+01 | 8.93E+01 | 1.10E+02 | 9.79E+01 | 1.51E+02 | 8.90E+01 | 1.00E+02 | 9.22E+01 | 9.39E+01 | 9.67E+01 | 1.01E+02 | 16.24 |  | x | x |
| 279.0142 | 1.02 | 5.20E+01 | 4.55E+01 | 4.48E+01 | 4.53E+01 | 4.85E+01 | 4.74E+01 | 4.38E+01 | 4.19E+01 | 4.74E+01 | 4.90E+01 | 8.46E+01 | 3.60E+01 | 4.35E+01 | 4.12E+01 | 4.60E+01 | 4.71E+01 | 4.71E+01 | 21.3 |  |  | x |
| 307.0450 | 1.02 | 6.65E+01 | 5.35E+01 | 5.91E+01 | 5.67E+01 | 5.86E+01 | 5.96E+01 | 5.55E+01 | 5.76E+01 | 6.00E+01 | 5.52E+01 | 9.64E+01 | 4.88E+01 | 5.67E+01 | 5.50E+01 | 4.82E+01 | 5.52E+01 | 5.52E+01 | 18.03 |  | x | x |
| 308.9234 | 1.02 | 6.11E+01 | 4.77E+01 | 5.35E+01 | 5.19E+01 | 4.97E+01 | 6.02E+01 | 5.11E+01 | 5.22E+01 | 5.56E+01 | 4.94E+01 | 7.60E+01 | 4.53E+01 | 5.60E+01 | 5.10E+01 | 4.48E+01 | 4.74E+01 | 5.82E+01 | 14.01 | x | x | x |
| 327.0481 | 1.02 | 1.93E+02 | 1.84E+02 | 2.10E+02 | 2.24E+02 | 2.47E+02 | 2.50E+02 | 2.34E+02 | 2.40E+02 | 2.60E+02 | 2.22E+02 | 3.86E+02 | 2.38E+02 | 2.31E+02 | 2.42E+02 | 2.15E+02 | 2.31E+02 | 2.44E+02 | 17.98 |  | x | x |
| 352.9493 | 1.02 | 2.23E+02 | 2.07E+02 | 2.17E+02 | 2.05E+02 | 2.14E+02 | 2.16E+02 | 1.96E+02 | 2.01E+02 | 2.11E+02 | 1.83E+02 | 3.29E+02 | 1.67E+02 | 2.05E+02 | 1.97E+02 | 1.84E+02 | 2.09E+02 | 1.92E+02 | 16.23 |  | x | x |
| 321.0822 | 1.03 | 5.44E+01 | 6.35E+01 | 5.30E+01 | 7.57E+01 | 6.50E+01 | 6.83E+01 | 5.88E+01 | 6.33E+01 | 6.71E+01 | 5.96E+01 | 1.06E+02 | 3.62E+01 | 7.07E+01 | 6.00E+01 | 6.85E+01 | 6.62E+01 | 6.05E+01 | 21.48 |  |  | x |
| 354.9478 | 1.03 | 1.32E+01 | 1.58E+01 | 1.69E+01 | 1.32E+01 | 1.57E+01 | 1.22E+01 | 1.47E+01 | 1.68E+01 | 1.36E+01 | 1.11E+01 | 1.59E+01 | 7.85E+00 | 1.52E+01 | 1.00E+01 | 8.37E+00 | 1.53E+01 | 8.82E+00 | 22.71 |  |  | x |
| 222.0204 | 1.05 | 1.13E+02 | 1.02E+02 | 1.03E+02 | 1.01E+02 | 1.04E+02 | 1.14E+02 | 1.08E+02 | 1.12E+02 | 1.16E+02 | 1.04E+02 | 1.77E+02 | 1.10E+02 | 1.14E+02 | 1.10E+02 | 1.01E+02 | 1.16E+02 | 1.18E+02 | 15.43 |  | x | x |
| 277.1044 | 1.05 | 1.80E+01 | 1.67E+01 | 2.75E+01 | 2.80E+01 | 2.60E+01 | 3.07E+01 | 3.05E+01 | 2.79E+01 | 2.98E+01 | 2.22E+01 | 5.08E+01 | 3.26E+01 | 2.49E+01 | 2.51E+01 | 2.46E+01 | 1.97E+01 | 2.91E+01 | 27.72 |  |  | x |
| 269.0899 | 1.05 | 4.90E+01 | 4.85E+01 | 5.55E+01 | 7.23E+01 | 8.57E+01 | 8.39E+01 | 8.52E+01 | 9.09E+01 | 8.93E+01 | 7.73E+01 | 1.41E+02 | 1.13E+02 | 9.06E+01 | 9.00E+01 | 9.35E+01 | 9.13E+01 | 8.97E+01 | 26.03 |  |  | x |
| 321.0612 | 1.05 | 1.69E+02 | 1.55E+02 | 1.85E+02 | 1.98E+02 | 2.10E+02 | 2.12E+02 | 2.21E+02 | 2.10E+02 | 2.22E+02 | 1.87E+02 | 3.50E+02 | 2.03E+02 | 1.95E+02 | 1.97E+02 | 1.77E+02 | 1.89E+02 | 2.02E+02 | 20.28 |  |  | x |
| 299.1005 | 1.07 | 2.54E+02 | 2.44E+02 | 2.77E+02 | 3.37E+02 | 3.71E+02 | 3.80E+02 | 3.82E+02 | 3.88E+02 | 4.00E+02 | 3.27E+02 | 5.98E+02 | 3.82E+02 | 3.58E+02 | 3.70E+02 | 3.53E+02 | 3.73E+02 | 3.91E+02 | 21.13 |  |  | x |
| 375.0251 | 1.07 | 2.68E+03 | 2.26E+03 | 2.47E+03 | 2.40E+03 | 2.46E+03 | 2.54E+03 | 2.38E+03 | 2.39E+03 | 2.48E+03 | 2.24E+03 | 3.71E+03 | 2.15E+03 | 2.38E+03 | 2.41E+03 | 2.23E+03 | 2.32E+03 | 2.33E+03 | 14.03 | x | x | x |
| 425.1580 | 1.07 | 1.48E+02 | 1.31E+02 | 1.41E+02 | 1.37E+02 | 1.38E+02 | 1.36E+02 | 1.22E+02 | 1.28E+02 | 1.33E+02 | 1.20E+02 | 1.98E+02 | 1.19E+02 | 1.16E+02 | 1.18E+02 | 1.17E+02 | 1.15E+02 | 1.23E+02 | 14.93 | x | x | x |
| 711.0863 | 1.07 | 7.67E+02 | 5.55E+02 | 5.85E+02 | 4.76E+02 | 4.20E+02 | 4.16E+02 | 3.79E+02 | 3.78E+02 | 3.87E+02 | 3.62E+02 | 5.47E+02 | 2.80E+02 | 3.63E+02 | 3.64E+02 | 3.09E+02 | 3.41E+02 | 3.45E+02 | 28.78 |  |  | x |
| 283.0629 | 1.08 | 1.63E+01 | 1.22E+01 | 1.78E+01 | 1.98E+01 | 1.80E+01 | 2.16E+01 | 2.30E+01 | 2.08E+01 | 2.10E+01 | 1.71E+01 | 3.14E+01 | 2.58E+01 | 1.60E+01 | 1.76E+01 | 2.18E+01 | 2.03E+01 | 1.84E+01 | 21.55 |  |  | x |
| 239.1005 | 1.09 | 2.90E+02 | 2.80E+02 | 3.04E+02 | 3.36E+02 | 3.53E+02 | 3.70E+02 | 3.56E+02 | 3.66E+02 | 3.89E+02 | 3.38E+02 | 5.74E+02 | 5.72E+02 | 3.79E+02 | 3.64E+02 | 3.44E+02 | 3.54E+02 | 3.73E+02 | 21.71 |  |  | x |
| 293.0300 | 1.09 | 5.40E+02 | 4.64E+02 | 5.00E+02 | 4.96E+02 | 5.24E+02 | 5.37E+02 | 5.19E+02 | 5.00E+02 | 5.38E+02 | 4.79E+02 | 8.19E+02 | 4.33E+02 | 5.09E+02 | 5.06E+02 | 4.59E+02 | 4.88E+02 | 5.06E+02 | 15.96 |  | x | x |
| 345.0347 | 1.10 | 9.45E+01 | 7.88E+01 | 8.82E+01 | 7.70E+01 | 7.65E+01 | 8.50E+01 | 8.22E+01 | 8.29E+01 | 8.72E+01 | 7.29E+01 | 1.29E+02 | 1.20E+02 | 8.01E+01 | 8.08E+01 | 6.34E+01 | 6.81E+01 | 7.15E+01 | 20.06 |  |  | x |
| 361.0091 | 1.10 | 9.82E+02 | 8.22E+02 | 9.14E+02 | 8.95E+02 | 8.87E+02 | 9.66E+02 | 8.81E+02 | 8.77E+02 | 8.76E+02 | 7.87E+02 | 1.29E+03 | 7.52E+02 | 8.17E+02 | 8.11E+02 | 7.74E+02 | 8.06E+02 | 7.89E+02 | 14.28 | x | x | x |
| 256.0784 | 1.11 | 3.13E+01 | 2.97E+01 | 4.20E+01 | 4.69E+01 | 5.08E+01 | 4.93E+01 | 4.89E+01 | 5.13E+01 | 5.50E+01 | 5.54E+01 | 8.47E+01 | 5.47E+01 | 5.31E+01 | 5.20E+01 | 5.51E+01 | 5.21E+01 | 5.63E+01 | 22.85 |  |  | x |
| 273.0873 | 1.11 | 2.02E+02 | 1.75E+02 | 1.87E+02 | 1.85E+02 | 1.89E+02 | 1.84E+02 | 1.89E+02 | 1.81E+02 | 1.99E+02 | 1.69E+02 | 2.86E+02 | 1.81E+02 | 1.88E+02 | 1.87E+02 | 1.77E+02 | 1.82E+02 | 1.93E+02 | 13.43 | x | x | x |
| 332.1240 | 1.11 | 2.82E+02 | 2.69E+02 | 2.88E+02 | 3.18E+02 | 3.31E+02 | 3.46E+02 | 3.45E+02 | 3.38E+02 | 3.58E+02 | 3.10E+02 | 5.36E+02 | 3.50E+02 | 3.35E+02 | 3.25E+02 | 3.03E+02 | 3.24E+02 | 3.26E+02 | 17.24 |  | x | x |
| 348.1195 | 1.11 | 2.01E+01 | 2.34E+01 | 2.25E+01 | 2.32E+01 | 2.43E+01 | 2.45E+01 | 2.60E+01 | 2.42E+01 | 2.24E+01 | 2.34E+01 | 3.51E+01 | 1.92E+01 | 2.30E+01 | 1.52E+01 | 1.72E+01 | 1.67E+01 | 2.01E+01 | 19.98 |  | x | x |
| 209.0714 | 1.12 | 1.43E+01 | 1.09E+01 | 1.29E+01 | 1.30E+01 | 1.56E+01 | 9.76E+00 | 1.26E+01 | 1.26E+01 | 1.28E+01 | 1.17E+01 | 1.26E+01 | 1.21E+01 | 1.16E+01 | 1.35E+01 | 1.24E+01 | 1.15E+01 | 1.65E+01 | 12.73 | x | x | x |
| 282.2643 | 1.12 | 3.45E+01 | 3.25E+01 | 3.67E+01 | 3.95E+01 | 4.21E+01 | 3.92E+01 | 3.98E+01 | 4.20E+01 | 4.20E+01 | 3.51E+01 | 6.26E+01 | 4.22E+01 | 4.21E+01 | 3.81E+01 | 4.14E+01 | 4.02E+01 | 4.15E+01 | 15.73 |  | x | x |
| 298.0979 | 1.12 | 7.03E+00 | 7.70E+00 | 9.41E+00 | 1.16E+01 | 8.69E+00 | 1.02E+01 | 1.04E+01 | 1.25E+01 | 7.18E+00 | 8.05E+00 | 1.16E+01 | 1.12E+01 | 9.51E+00 | 6.78E+00 | 1.11E+01 | 7.53E+00 | 9.71E+00 | 19.39 |  | x | x |
| 346.9932 | 1.12 | 8.90E+01 | 7.75E+01 | 8.59E+01 | 9.47E+01 | 8.20E+01 | 8.09E+01 | 8.07E+01 | 8.30E+01 | 8.11E+01 | 6.30E+01 | 1.30E+02 | 6.57E+01 | 7.24E+01 | 7.08E+01 | 6.28E+01 | 6.95E+01 | 7.09E+01 | 19.65 |  | x | x |
| 143.0813 | 1.13 | 5.53E+02 | 5.04E+02 | 5.43E+02 | 5.83E+02 | 6.85E+02 | 7.23E+02 | 7.30E+02 | 7.51E+02 | 7.78E+02 | 7.12E+02 | 1.25E+03 | 7.24E+02 | 8.18E+02 | 8.36E+02 | 8.28E+02 | 8.32E+02 | 8.81E+02 | 22.86 |  |  | x |
| 282.1204 | 1.13 | 2.58E+03 | 2.36E+03 | 2.65E+03 | 2.91E+03 | 3.05E+03 | 3.18E+03 | 3.05E+03 | 3.13E+03 | 3.21E+03 | 2.80E+03 | 4.82E+03 | 3.02E+03 | 3.03E+03 | 2.93E+03 | 3.00E+03 | 2.96E+03 | 3.10E+03 | 16.68 |  | x | x |
| 282.2030 | 1.13 | 9.71E+00 | 9.60E+00 | 1.17E+01 | 1.22E+01 | 8.30E+00 | 9.04E+00 | 1.06E+01 | 1.13E+01 | 1.02E+01 | 8.08E+00 | 1.39E+01 | 1.22E+01 | 1.15E+01 | 6.90E+00 | 1.15E+01 | 8.77E+00 | 1.05E+01 | 17.39 |  | x | x |
| 243.1339 | 1.14 | 3.85E+01 | 3.39E+01 | 3.72E+01 | 3.72E+01 | 4.83E+01 | 4.58E+01 | 4.18E+01 | 4.58E+01 | 4.78E+01 | 4.11E+01 | 7.52E+01 | 4.77E+01 | 4.96E+01 | 4.41E+01 | 4.93E+01 | 5.32E+01 | 4.76E+01 | 19.88 |  | x | x |
| 174.0890 | 1.15 | 8.52E+00 | 1.13E+01 | 7.58E+00 | 9.35E+00 | 1.28E+01 | 1.06E+01 | 1.32E+01 | 1.00E+01 | 1.05E+01 | 1.01E+01 | 1.74E+01 | 1.07E+01 | 1.14E+01 | 9.63E+00 | 9.60E+00 | 9.68E+00 | 1.03E+01 | 20.37 |  |  | x |
| 184.0964 | 1.15 | 8.61E+00 | 9.70E+00 | 5.98E+00 | 7.56E+00 | 7.66E+00 | 7.82E+00 | 8.68E+00 | 8.82E+00 | 9.48E+00 | 8.61E+00 | 1.79E+01 | 1.11E+01 | 9.11E+00 | 8.80E+00 | 9.15E+00 | 9.19E+00 | 9.25E+00 | 26.87 |  |  | x |
| 366.1549 | 1.15 | 8.05E+00 | 6.95E+00 | 6.40E+00 | 6.19E+00 | 4.52E+00 | 6.06E+00 | 6.79E+00 | 3.80E+00 | 5.50E+00 | 3.21E+00 | 8.77E+00 | 7.28E+00 | 7.01E+00 | 5.53E+00 | 3.32E+00 | 6.89E+00 | 3.47E+00 | 28.77 |  |  | x |
| 377.0050 | 1.15 | 1.13E+02 | 1.01E+02 | 1.05E+02 | 9.05E+01 | 8.46E+01 | 9.21E+01 | 8.90E+01 | 8.81E+01 | 9.37E+01 | 8.35E+01 | 1.34E+02 | 7.50E+01 | 9.05E+01 | 8.62E+01 | 6.07E+01 | 7.91E+01 | 8.67E+01 | 17.52 |  | x | x |
| 250.9832 | 1.16 | 3.28E+01 | 2.83E+01 | 2.94E+01 | 2.90E+01 | 2.60E+01 | 3.17E+01 | 3.15E+01 | 2.82E+01 | 3.56E+01 | 2.94E+01 | 4.81E+01 | 2.59E+01 | 2.79E+01 | 3.15E+01 | 2.26E+01 | 3.02E+01 | 3.60E+01 | 18.11 |  | x | x |
| 354.9337 | 1.17 | 7.44E+01 | 6.10E+01 | 7.22E+01 | 5.80E+01 | 5.81E+01 | 5.33E+01 | 6.39E+01 | 5.15E+01 | 6.02E+01 | 5.39E+01 | 9.97E+01 | 4.42E+01 | 5.45E+01 | 5.25E+01 | 4.70E+01 | 5.00E+01 | 5.76E+01 | 21.9 |  |  | x |
| 166.0502 | 1.18 | 9.07E+01 | 8.70E+01 | 1.01E+02 | 9.11E+01 | 1.01E+02 | 1.08E+02 | 1.12E+02 | 1.10E+02 | 1.19E+02 | 9.81E+01 | 1.78E+02 | 1.14E+02 | 1.12E+02 | 1.10E+02 | 9.16E+01 | 1.17E+02 | 1.23E+02 | 18.92 |  | x | x |
| 264.9991 | 1.18 | 1.86E+02 | 1.59E+02 | 1.84E+02 | 1.86E+02 | 1.94E+02 | 2.00E+02 | 1.95E+02 | 1.85E+02 | 2.03E+02 | 1.78E+02 | 2.96E+02 | 1.51E+02 | 1.89E+02 | 1.89E+02 | 1.57E+02 | 1.84E+02 | 1.90E+02 | 16.35 |  | x | x |
| 357.0967 | 1.18 | 1.12E+01 | 8.36E+00 | 9.87E+00 | 7.61E+00 | 7.02E+00 | 8.05E+00 | 4.97E+00 | 3.51E+00 | 1.18E+01 | 5.02E+00 | 7.75E+00 | 8.32E+00 | 6.59E+00 | 7.36E+00 | 5.62E+00 | 8.12E+00 | 6.94E+00 | 28.43 |  |  | x |
| 363.0408 | 1.18 | 3.55E+01 | 3.30E+01 | 3.55E+01 | 3.80E+01 | 4.04E+01 | 3.93E+01 | 3.79E+01 | 3.24E+01 | 3.89E+01 | 2.72E+01 | 4.85E+01 | 2.45E+01 | 3.70E+01 | 3.24E+01 | 2.45E+01 | 3.75E+01 | 2.84E+01 | 17.82 |  | x | x |
| 439.1363 | 1.18 | 4.73E+01 | 3.59E+01 | 4.28E+01 | 3.12E+01 | 2.46E+01 | 2.43E+01 | 2.69E+01 | 2.15E+01 | 2.62E+01 | 2.58E+01 | 3.62E+01 | 2.45E+01 | 1.98E+01 | 1.79E+01 | 2.63E+01 | 2.06E+01 | 2.40E+01 | 29.13 |  |  | x |
| 116.0498 | 1.19 | 1.14E+02 | 1.00E+02 | 1.04E+02 | 1.17E+02 | 1.23E+02 | 1.34E+02 | 1.30E+02 | 1.37E+02 | 1.36E+02 | 1.18E+02 | 2.09E+02 | 1.35E+02 | 1.38E+02 | 1.40E+02 | 1.41E+02 | 1.45E+02 | 1.53E+02 | 18.03 |  | x | x |
| 139.0507 | 1.19 | 1.35E+01 | 1.44E+01 | 1.27E+01 | 1.36E+01 | 1.95E+01 | 1.64E+01 | 1.73E+01 | 1.78E+01 | 1.93E+01 | 1.64E+01 | 2.61E+01 | 1.50E+01 | 1.98E+01 | 1.52E+01 | 1.85E+01 | 1.72E+01 | 1.98E+01 | 18.85 |  | x | x |
| 148.0404 | 1.19 | 8.66E+00 | 6.99E+00 | 1.09E+01 | 7.37E+00 | 5.97E+00 | 7.86E+00 | 9.43E+00 | 8.36E+00 | 1.02E+01 | 1.04E+01 | 1.16E+01 | 1.06E+01 | 1.06E+01 | 8.04E+00 | 6.38E+00 | 9.28E+00 | 1.42E+01 | 22.74 |  |  | x |
| 162.0557 | 1.19 | 1.20E+03 | 1.07E+03 | 1.19E+03 | 1.23E+03 | 1.36E+03 | 1.41E+03 | 1.40E+03 | 1.42E+03 | 1.48E+03 | 1.30E+03 | 2.15E+03 | 1.40E+03 | 1.42E+03 | 1.42E+03 | 1.41E+03 | 1.43E+03 | 1.49E+03 | 16.13 |  | x | x |
| 244.9015 | 1.19 | 2.76E+01 | 1.66E+01 | 2.18E+01 | 1.75E+01 | 1.83E+01 | 2.19E+01 | 2.10E+01 | 1.64E+01 | 1.93E+01 | 1.19E+01 | 2.36E+01 | 1.70E+01 | 1.65E+01 | 1.50E+01 | 1.56E+01 | 1.61E+01 | 1.37E+01 | 21.44 |  |  | x |
| 229.1552 | 1.19 | 6.51E+03 | 5.96E+03 | 6.60E+03 | 6.71E+03 | 7.24E+03 | 7.48E+03 | 7.35E+03 | 7.51E+03 | 7.69E+03 | 6.76E+03 | 1.02E+04 | 7.17E+03 | 7.33E+03 | 7.45E+03 | 7.47E+03 | 7.30E+03 | 7.55E+03 | 11.91 | x | x | x |
| 213.0981 | 1.19 | 6.59E+00 | 7.21E+00 | 9.69E+00 | 8.67E+00 | 8.78E+00 | 6.60E+00 | 5.65E+00 | 5.39E+00 | 1.15E+01 | 7.65E+00 | 1.01E+01 | 8.50E+00 | 5.47E+00 | 4.31E+00 | 8.44E+00 | 9.42E+00 | 9.41E+00 | 25.05 |  |  | x |
| 240.1224 | 1.19 | 7.23E+01 | 6.66E+01 | 6.80E+01 | 7.72E+01 | 7.91E+01 | 7.95E+01 | 8.97E+01 | 8.07E+01 | 9.12E+01 | 6.97E+01 | 1.16E+02 | 1.03E+02 | 7.68E+01 | 7.47E+01 | 7.93E+01 | 8.38E+01 | 8.66E+01 | 15.48 |  | x | x |
| 257.1135 | 1.19 | 7.70E+01 | 6.73E+01 | 7.17E+01 | 8.13E+01 | 8.29E+01 | 8.55E+01 | 8.85E+01 | 7.86E+01 | 9.09E+01 | 7.50E+01 | 1.24E+02 | 8.00E+01 | 8.55E+01 | 8.47E+01 | 7.87E+01 | 8.30E+01 | 8.71E+01 | 14.39 | x | x | x |
| 311.1003 | 1.19 | 2.61E+02 | 2.43E+02 | 2.74E+02 | 2.90E+02 | 3.05E+02 | 3.09E+02 | 3.08E+02 | 2.92E+02 | 3.23E+02 | 2.77E+02 | 4.60E+02 | 3.11E+02 | 2.92E+02 | 2.69E+02 | 2.59E+02 | 2.96E+02 | 3.01E+02 | 15.7 |  | x | x |
| 285.0845 | 1.19 | 4.41E+01 | 4.22E+01 | 4.77E+01 | 5.40E+01 | 5.20E+01 | 5.72E+01 | 5.34E+01 | 5.29E+01 | 5.95E+01 | 5.12E+01 | 8.55E+01 | 5.65E+01 | 5.26E+01 | 5.31E+01 | 4.50E+01 | 5.09E+01 | 5.65E+01 | 17.55 |  | x | x |
| 309.1543 | 1.19 | 1.05E+01 | 1.12E+01 | 1.22E+01 | 7.36E+00 | 7.65E+00 | 9.79E+00 | 9.47E+00 | 6.75E+00 | 9.09E+00 | 6.50E+00 | 1.20E+01 | 1.24E+01 | 7.34E+00 | 1.04E+01 | 6.58E+00 | 7.02E+00 | 9.56E+00 | 22.65 |  |  | x |
| 441.1514 | 1.19 | 1.94E+02 | 1.75E+02 | 1.74E+02 | 1.66E+02 | 1.53E+02 | 1.53E+02 | 1.45E+02 | 1.37E+02 | 1.46E+02 | 1.15E+02 | 2.01E+02 | 1.50E+02 | 1.31E+02 | 1.26E+02 | 1.22E+02 | 1.20E+02 | 1.27E+02 | 17.25 |  | x | x |
| 443.1670 | 1.19 | 9.51E+01 | 7.52E+01 | 8.56E+01 | 7.52E+01 | 6.79E+01 | 6.29E+01 | 6.27E+01 | 5.94E+01 | 6.26E+01 | 5.67E+01 | 6.75E+01 | 5.35E+01 | 5.85E+01 | 5.97E+01 | 4.43E+01 | 5.29E+01 | 5.35E+01 | 19.7 |  | x | x |
| 89.0388 | 1.20 | 1.95E+01 | 1.63E+01 | 1.97E+01 | 1.98E+01 | 2.39E+01 | 2.15E+01 | 2.26E+01 | 2.47E+01 | 2.38E+01 | 2.04E+01 | 3.73E+01 | 2.29E+01 | 2.45E+01 | 2.84E+01 | 2.63E+01 | 2.76E+01 | 2.56E+01 | 19.8 |  | x | x |
| 162.1638 | 1.20 | 9.88E+00 | 8.98E+00 | 9.31E+00 | 1.00E+01 | 1.26E+01 | 1.11E+01 | 1.08E+01 | 1.08E+01 | 1.22E+01 | 8.78E+00 | 1.68E+01 | 9.76E+00 | 1.23E+01 | 1.06E+01 | 1.12E+01 | 1.14E+01 | 1.12E+01 | 16.93 |  | x | x |
| 190.0506 | 1.20 | 7.03E+02 | 6.38E+02 | 7.08E+02 | 7.44E+02 | 8.07E+02 | 8.91E+02 | 8.74E+02 | 8.81E+02 | 9.43E+02 | 8.22E+02 | 1.35E+03 | 8.99E+02 | 9.28E+02 | 9.34E+02 | 9.24E+02 | 9.43E+02 | 9.89E+02 | 17.77 |  | x | x |
| 190.1677 | 1.20 | 5.68E+00 | 4.53E+00 | 6.01E+00 | 6.44E+00 | 6.11E+00 | 6.84E+00 | 6.49E+00 | 6.96E+00 | 7.55E+00 | 6.55E+00 | 1.04E+01 | 7.52E+00 | 7.22E+00 | 8.74E+00 | 9.18E+00 | 8.95E+00 | 7.40E+00 | 20.05 |  |  | x |
| 185.0711 | 1.20 | 1.32E+02 | 1.29E+02 | 1.45E+02 | 1.58E+02 | 1.77E+02 | 1.77E+02 | 1.73E+02 | 1.76E+02 | 1.78E+02 | 1.63E+02 | 2.65E+02 | 1.66E+02 | 1.75E+02 | 1.74E+02 | 1.82E+02 | 1.81E+02 | 1.91E+02 | 16.94 |  | x | x |
| 352.9499 | 1.20 | 1.30E+02 | 1.15E+02 | 1.17E+02 | 1.01E+02 | 9.58E+01 | 1.18E+02 | 1.04E+02 | 1.03E+02 | 1.03E+02 | 9.37E+01 | 1.57E+02 | 9.92E+01 | 9.77E+01 | 1.04E+02 | 8.84E+01 | 9.88E+01 | 9.84E+01 | 15.22 |  | x | x |
| 150.0776 | 1.21 | 9.31E+02 | 8.28E+02 | 9.48E+02 | 9.67E+02 | 1.09E+03 | 1.12E+03 | 1.13E+03 | 1.14E+03 | 1.19E+03 | 1.03E+03 | 1.74E+03 | 1.13E+03 | 1.14E+03 | 1.14E+03 | 1.08E+03 | 1.13E+03 | 1.21E+03 | 17.13 |  | x | x |
| 300.9184 | 1.21 | 8.26E+01 | 6.89E+01 | 7.56E+01 | 6.73E+01 | 6.95E+01 | 7.42E+01 | 7.08E+01 | 7.04E+01 | 7.25E+01 | 5.94E+01 | 1.06E+02 | 5.90E+01 | 6.32E+01 | 6.58E+01 | 5.70E+01 | 6.58E+01 | 6.25E+01 | 16.08 |  | x | x |
| 317.0543 | 1.21 | 5.73E+01 | 5.28E+01 | 6.40E+01 | 6.13E+01 | 7.11E+01 | 7.83E+01 | 6.99E+01 | 7.89E+01 | 7.07E+01 | 6.50E+01 | 1.06E+02 | 7.50E+01 | 7.78E+01 | 6.72E+01 | 6.43E+01 | 6.55E+01 | 6.81E+01 | 16.78 |  | x | x |
| 336.9187 | 1.21 | 2.19E+01 | 1.87E+01 | 2.03E+01 | 1.91E+01 | 2.12E+01 | 1.78E+01 | 1.84E+01 | 1.93E+01 | 2.03E+01 | 2.07E+01 | 2.47E+01 | 1.60E+01 | 2.06E+01 | 1.69E+01 | 2.04E+01 | 1.78E+01 | 1.79E+01 | 10.66 | x | x | x |
| 338.1105 | 1.21 | 1.66E+01 | 1.47E+01 | 1.97E+01 | 1.84E+01 | 2.12E+01 | 1.81E+01 | 1.62E+01 | 1.66E+01 | 1.75E+01 | 1.41E+01 | 1.96E+01 | 1.67E+01 | 1.01E+01 | 1.00E+01 | 8.91E+00 | 1.58E+01 | 1.48E+01 | 22.03 |  |  | x |
| 413.1576 | 1.21 | 3.82E+01 | 3.63E+01 | 3.53E+01 | 3.67E+01 | 3.58E+01 | 2.99E+01 | 3.43E+01 | 2.90E+01 | 3.35E+01 | 3.14E+01 | 4.88E+01 | 2.81E+01 | 3.28E+01 | 3.48E+01 | 2.54E+01 | 2.60E+01 | 3.13E+01 | 16.39 |  | x | x |
| 457.0915 | 1.21 | 2.61E+03 | 2.30E+03 | 2.48E+03 | 2.26E+03 | 2.20E+03 | 2.31E+03 | 2.19E+03 | 2.12E+03 | 2.28E+03 | 1.95E+03 | 3.05E+03 | 1.96E+03 | 2.09E+03 | 1.98E+03 | 1.88E+03 | 1.96E+03 | 2.00E+03 | 13.34 | x | x | x |
| 457.2779 | 1.21 | 2.07E+01 | 1.56E+01 | 2.33E+01 | 1.80E+01 | 1.57E+01 | 1.59E+01 | 1.63E+01 | 1.66E+01 | 1.77E+01 | 1.23E+01 | 2.20E+01 | 1.56E+01 | 1.72E+01 | 1.41E+01 | 1.46E+01 | 1.43E+01 | 1.68E+01 | 17.02 |  | x | x |
| 457.3800 | 1.21 | 9.43E+00 | 8.88E+00 | 1.26E+01 | 8.16E+00 | 8.33E+00 | 6.81E+00 | 7.57E+00 | 7.15E+00 | 5.65E+00 | 6.83E+00 | 8.82E+00 | 5.61E+00 | 6.74E+00 | 5.13E+00 | 4.86E+00 | 5.00E+00 | 5.25E+00 | 28.09 |  |  | x |
| 206.0825 | 1.22 | 1.02E+01 | 6.74E+00 | 1.12E+01 | 1.09E+01 | 1.32E+01 | 1.21E+01 | 1.16E+01 | 1.16E+01 | 1.05E+01 | 9.81E+00 | 1.86E+01 | 8.92E+00 | 1.20E+01 | 1.08E+01 | 1.05E+01 | 9.39E+00 | 1.28E+01 | 21.74 |  |  | x |
| 313.0819 | 1.22 | 2.42E+01 | 2.28E+01 | 3.20E+01 | 3.15E+01 | 3.77E+01 | 3.02E+01 | 3.23E+01 | 3.21E+01 | 3.91E+01 | 2.79E+01 | 3.79E+01 | 3.11E+01 | 3.30E+01 | 3.20E+01 | 2.58E+01 | 3.22E+01 | 3.72E+01 | 14.74 | x | x | x |
| 338.9345 | 1.22 | 9.77E+02 | 8.53E+02 | 9.19E+02 | 8.60E+02 | 8.72E+02 | 8.99E+02 | 8.41E+02 | 8.15E+02 | 8.67E+02 | 7.84E+02 | 1.12E+03 | 7.67E+02 | 8.30E+02 | 8.17E+02 | 7.46E+02 | 7.91E+02 | 8.11E+02 | 10.43 | x | x | x |
| 339.0959 | 1.22 | 1.94E+01 | 1.54E+01 | 1.70E+01 | 1.57E+01 | 1.45E+01 | 1.60E+01 | 1.24E+01 | 1.13E+01 | 1.37E+01 | 1.20E+01 | 1.76E+01 | 9.89E+00 | 1.44E+01 | 1.11E+01 | 7.59E+00 | 9.95E+00 | 1.11E+01 | 23.58 |  |  | x |
| 340.9383 | 1.22 | 2.51E+02 | 2.24E+02 | 2.44E+02 | 2.08E+02 | 2.15E+02 | 2.38E+02 | 2.17E+02 | 2.09E+02 | 2.12E+02 | 1.89E+02 | 3.26E+02 | 1.84E+02 | 2.09E+02 | 2.05E+02 | 1.83E+02 | 1.95E+02 | 2.12E+02 | 15.38 |  | x | x |
| 132.0446 | 1.23 | 3.22E+01 | 3.45E+01 | 3.76E+01 | 4.37E+01 | 4.53E+01 | 4.67E+01 | 4.21E+01 | 4.49E+01 | 4.21E+01 | 3.51E+01 | 6.05E+01 | 3.62E+01 | 4.11E+01 | 3.84E+01 | 4.86E+01 | 3.95E+01 | 4.25E+01 | 15.83 |  | x | x |
| 173.0728 | 1.23 | 2.08E+00 | 2.35E+00 | 3.11E+00 | 2.15E+00 | 2.44E+00 | 3.38E+00 | 2.02E+00 | 1.99E+00 | 2.39E+00 | 2.24E+00 | 3.61E+00 | 3.26E+00 | 2.03E+00 | 1.91E+00 | 1.99E+00 | 3.10E+00 | 1.40E+00 | 25.52 |  |  | x |
| 178.0503 | 1.23 | 6.42E+01 | 6.23E+01 | 6.42E+01 | 7.77E+01 | 7.78E+01 | 7.64E+01 | 7.53E+01 | 7.85E+01 | 7.01E+01 | 5.89E+01 | 1.02E+02 | 6.03E+01 | 7.15E+01 | 6.49E+01 | 7.97E+01 | 6.57E+01 | 6.53E+01 | 14.59 | x | x | x |
| 201.0657 | 1.23 | 5.69E+00 | 7.50E+00 | 7.20E+00 | 6.22E+00 | 6.92E+00 | 9.45E+00 | 5.92E+00 | 8.94E+00 | 6.77E+00 | 7.08E+00 | 1.35E+01 | 5.95E+00 | 7.65E+00 | 7.70E+00 | 1.13E+01 | 8.39E+00 | 6.37E+00 | 26.39 |  |  | x |
| 283.0603 | 1.23 | 9.12E+01 | 7.91E+01 | 9.46E+01 | 8.70E+01 | 9.04E+01 | 8.70E+01 | 9.05E+01 | 8.62E+01 | 9.29E+01 | 7.22E+01 | 1.16E+02 | 9.18E+01 | 8.22E+01 | 7.71E+01 | 8.02E+01 | 8.17E+01 | 8.63E+01 | 11.04 | x | x | x |
| 322.9601 | 1.23 | 5.04E+01 | 4.46E+01 | 4.57E+01 | 4.40E+01 | 4.83E+01 | 4.49E+01 | 4.55E+01 | 4.20E+01 | 4.63E+01 | 4.36E+01 | 6.49E+01 | 8.69E+01 | 4.28E+01 | 3.96E+01 | 3.16E+01 | 4.36E+01 | 3.68E+01 | 26.03 |  |  | x |
| 327.0979 | 1.23 | 2.59E+01 | 2.88E+01 | 3.46E+01 | 3.13E+01 | 3.15E+01 | 3.73E+01 | 2.08E+01 | 3.30E+01 | 3.05E+01 | 3.21E+01 | 4.02E+01 | 3.50E+01 | 2.95E+01 | 3.07E+01 | 2.62E+01 | 2.68E+01 | 3.23E+01 | 14.85 | x | x | x |
| 473.0858 | 1.23 | 1.40E+02 | 1.12E+02 | 1.17E+02 | 9.93E+01 | 8.56E+01 | 9.00E+01 | 9.40E+01 | 8.34E+01 | 8.56E+01 | 7.19E+01 | 1.09E+02 | 8.15E+01 | 7.77E+01 | 7.24E+01 | 6.74E+01 | 7.77E+01 | 7.75E+01 | 21.25 |  |  | x |
| 206.0452 | 1.24 | 2.38E+02 | 2.14E+02 | 2.32E+02 | 2.81E+02 | 2.88E+02 | 2.86E+02 | 2.85E+02 | 3.01E+02 | 2.83E+02 | 2.57E+02 | 4.02E+02 | 2.59E+02 | 2.85E+02 | 2.67E+02 | 3.34E+02 | 2.74E+02 | 2.90E+02 | 14.9 | x | x | x |
| 174.2365 | 1.24 | 1.35E+01 | 1.15E+01 | 1.33E+01 | 1.40E+01 | 1.50E+01 | 1.60E+01 | 1.45E+01 | 1.68E+01 | 1.80E+01 | 1.43E+01 | 2.53E+01 | 2.15E+01 | 1.83E+01 | 1.94E+01 | 1.75E+01 | 1.88E+01 | 1.94E+01 | 20.36 |  |  | x |
| 294.0847 | 1.24 | 1.16E+01 | 1.10E+01 | 1.13E+01 | 1.26E+01 | 1.20E+01 | 1.26E+01 | 1.32E+01 | 1.34E+01 | 1.73E+01 | 9.07E+00 | 2.13E+01 | 1.22E+01 | 1.28E+01 | 1.20E+01 | 1.09E+01 | 1.27E+01 | 1.32E+01 | 21.14 |  |  | x |
| 341.0279 | 1.24 | 1.25E+03 | 1.10E+03 | 1.24E+03 | 1.26E+03 | 1.33E+03 | 1.39E+03 | 1.36E+03 | 1.35E+03 | 1.42E+03 | 1.24E+03 | 1.89E+03 | 1.38E+03 | 1.29E+03 | 1.29E+03 | 1.25E+03 | 1.27E+03 | 1.32E+03 | 12.25 | x | x | x |
| 366.0593 | 1.24 | 6.91E+02 | 6.25E+02 | 6.83E+02 | 7.25E+02 | 7.38E+02 | 7.66E+02 | 7.42E+02 | 7.35E+02 | 7.65E+02 | 6.77E+02 | 9.37E+02 | 7.15E+02 | 7.01E+02 | 6.96E+02 | 6.52E+02 | 6.71E+02 | 7.07E+02 | 9.44 | x | x | x |
| 341.1861 | 1.24 | 7.79E+00 | 7.18E+00 | 7.52E+00 | 7.98E+00 | 9.29E+00 | 9.26E+00 | 7.58E+00 | 8.92E+00 | 9.45E+00 | 7.11E+00 | 1.05E+01 | 7.81E+00 | 7.77E+00 | 7.75E+00 | 7.60E+00 | 7.95E+00 | 8.89E+00 | 11.4 | x | x | x |
| 377.0041 | 1.24 | 2.85E+01 | 2.46E+01 | 2.71E+01 | 2.10E+01 | 1.88E+01 | 2.06E+01 | 1.89E+01 | 1.49E+01 | 1.95E+01 | 1.62E+01 | 2.05E+01 | 1.39E+01 | 1.65E+01 | 1.17E+01 | 1.25E+01 | 1.48E+01 | 1.54E+01 | 26.12 |  |  | x |
| 378.9835 | 1.24 | 1.12E+03 | 9.26E+02 | 9.87E+02 | 8.97E+02 | 8.45E+02 | 8.84E+02 | 8.15E+02 | 7.88E+02 | 8.41E+02 | 7.54E+02 | 1.10E+03 | 7.98E+02 | 7.60E+02 | 7.72E+02 | 6.45E+02 | 7.32E+02 | 7.39E+02 | 15.05 |  | x | x |
| 404.0148 | 1.24 | 2.58E+02 | 2.11E+02 | 2.31E+02 | 1.96E+02 | 1.80E+02 | 1.77E+02 | 1.65E+02 | 1.62E+02 | 1.78E+02 | 1.46E+02 | 2.51E+02 | 1.63E+02 | 1.54E+02 | 1.55E+02 | 1.13E+02 | 1.42E+02 | 1.55E+02 | 22.07 |  |  | x |
| 479.1279 | 1.24 | 8.85E+01 | 7.45E+01 | 6.48E+01 | 6.28E+01 | 4.97E+01 | 5.40E+01 | 5.51E+01 | 5.32E+01 | 5.40E+01 | 4.78E+01 | 6.41E+01 | 3.68E+01 | 4.53E+01 | 4.57E+01 | 3.77E+01 | 3.61E+01 | 4.44E+01 | 25.81 |  |  | x |
| 174.1242 | 1.25 | 1.22E+03 | 1.09E+03 | 1.22E+03 | 1.24E+03 | 1.40E+03 | 1.47E+03 | 1.47E+03 | 1.47E+03 | 1.57E+03 | 1.37E+03 | 2.32E+03 | 1.59E+03 | 1.50E+03 | 1.55E+03 | 1.45E+03 | 1.53E+03 | 1.58E+03 | 17.84 |  | x | x |
| 180.0886 | 1.25 | 9.41E+01 | 9.53E+01 | 1.05E+02 | 1.04E+02 | 1.22E+02 | 1.35E+02 | 1.30E+02 | 1.31E+02 | 1.31E+02 | 1.17E+02 | 1.84E+02 | 1.15E+02 | 1.32E+02 | 1.28E+02 | 1.32E+02 | 1.41E+02 | 1.46E+02 | 16.85 |  | x | x |
| 271.0702 | 1.25 | 1.51E+02 | 1.29E+02 | 1.52E+02 | 1.51E+02 | 1.56E+02 | 1.65E+02 | 1.62E+02 | 1.72E+02 | 1.76E+02 | 1.57E+02 | 2.83E+02 | 1.73E+02 | 1.64E+02 | 1.67E+02 | 1.63E+02 | 1.69E+02 | 1.79E+02 | 18.81 |  | x | x |
| 396.0699 | 1.25 | 6.06E+01 | 4.77E+01 | 6.17E+01 | 5.93E+01 | 4.80E+01 | 5.71E+01 | 6.20E+01 | 5.36E+01 | 5.78E+01 | 4.89E+01 | 6.26E+01 | 5.28E+01 | 4.97E+01 | 5.24E+01 | 5.49E+01 | 4.96E+01 | 5.27E+01 | 9.45 | x | x | x |
| 441.1526 | 1.25 | 1.87E+02 | 1.32E+02 | 1.55E+02 | 1.19E+02 | 1.02E+02 | 1.18E+02 | 1.20E+02 | 9.80E+01 | 1.05E+02 | 9.95E+01 | 1.54E+02 | 1.02E+02 | 1.03E+02 | 1.11E+02 | 9.84E+01 | 8.77E+01 | 1.02E+02 | 22.17 |  |  | x |
| 171.1123 | 1.26 | 4.05E+01 | 3.58E+01 | 4.08E+01 | 3.53E+01 | 4.05E+01 | 4.62E+01 | 3.88E+01 | 4.02E+01 | 4.94E+01 | 4.83E+01 | 6.70E+01 | 5.10E+01 | 5.04E+01 | 4.69E+01 | 4.97E+01 | 5.33E+01 | 4.72E+01 | 16.86 |  | x | x |
| 205.0972 | 1.26 | 5.04E+00 | 9.28E+00 | 9.04E+00 | 1.07E+01 | 9.79E+00 | 1.14E+01 | 7.91E+00 | 9.56E+00 | 9.54E+00 | 8.78E+00 | 1.39E+01 | 9.42E+00 | 1.02E+01 | 9.29E+00 | 9.97E+00 | 1.22E+01 | 9.52E+00 | 18.95 |  | x | x |
| 241.1548 | 1.26 | 2.06E+03 | 1.93E+03 | 2.14E+03 | 2.14E+03 | 2.23E+03 | 2.31E+03 | 2.25E+03 | 2.26E+03 | 2.36E+03 | 2.07E+03 | 3.03E+03 | 2.15E+03 | 2.26E+03 | 2.31E+03 | 2.42E+03 | 2.21E+03 | 2.28E+03 | 10.32 | x | x | x |
| 285.0852 | 1.26 | 3.90E+02 | 3.34E+02 | 3.79E+02 | 3.94E+02 | 3.96E+02 | 4.15E+02 | 4.12E+02 | 3.95E+02 | 4.33E+02 | 3.67E+02 | 5.50E+02 | 4.05E+02 | 3.99E+02 | 3.97E+02 | 3.78E+02 | 3.98E+02 | 4.19E+02 | 10.82 | x | x | x |
| 308.0411 | 1.26 | 1.78E+02 | 1.49E+02 | 1.67E+02 | 1.50E+02 | 1.56E+02 | 1.55E+02 | 1.54E+02 | 1.44E+02 | 1.58E+02 | 1.43E+02 | 1.96E+02 | 1.38E+02 | 1.43E+02 | 1.43E+02 | 1.19E+02 | 1.41E+02 | 1.40E+02 | 11.45 | x | x | x |
| 309.0263 | 1.26 | 1.97E+02 | 1.47E+02 | 1.73E+02 | 1.70E+02 | 1.67E+02 | 1.64E+02 | 1.65E+02 | 1.50E+02 | 1.61E+02 | 1.46E+02 | 2.36E+02 | 1.54E+02 | 1.51E+02 | 1.44E+02 | 1.32E+02 | 1.47E+02 | 1.47E+02 | 15.02 |  | x | x |
| 301.1395 | 1.27 | 4.55E+01 | 4.52E+01 | 4.77E+01 | 5.01E+01 | 5.03E+01 | 5.18E+01 | 5.38E+01 | 5.45E+01 | 6.02E+01 | 5.03E+01 | 7.50E+01 | 5.49E+01 | 5.51E+01 | 5.19E+01 | 5.27E+01 | 5.15E+01 | 5.16E+01 | 12.66 | x | x | x |
| 324.9182 | 1.27 | 1.80E+02 | 1.51E+02 | 1.62E+02 | 1.54E+02 | 1.60E+02 | 1.51E+02 | 1.48E+02 | 1.46E+02 | 1.67E+02 | 1.42E+02 | 1.89E+02 | 1.30E+02 | 1.53E+02 | 1.50E+02 | 1.26E+02 | 1.42E+02 | 1.46E+02 | 10.39 | x | x | x |
| 326.9165 | 1.27 | 1.42E+01 | 1.30E+01 | 1.46E+01 | 1.35E+01 | 1.54E+01 | 1.26E+01 | 1.11E+01 | 1.00E+01 | 1.08E+01 | 8.21E+00 | 1.72E+01 | 1.17E+01 | 1.09E+01 | 1.34E+01 | 9.71E+00 | 1.33E+01 | 1.48E+01 | 18.45 |  | x | x |
| 340.1266 | 1.27 | 1.93E+01 | 1.76E+01 | 1.72E+01 | 2.00E+01 | 2.14E+01 | 1.16E+01 | 2.02E+01 | 1.91E+01 | 1.36E+01 | 1.20E+01 | 3.21E+01 | 1.15E+01 | 1.91E+01 | 2.38E+01 | 2.09E+01 | 1.87E+01 | 2.03E+01 | 26.76 |  |  | x |
| 353.0638 | 1.27 | 8.19E+01 | 6.77E+01 | 9.14E+01 | 8.61E+01 | 9.43E+01 | 9.23E+01 | 9.56E+01 | 9.77E+01 | 8.92E+01 | 7.89E+01 | 1.12E+02 | 8.74E+01 | 9.07E+01 | 8.56E+01 | 8.63E+01 | 8.26E+01 | 9.52E+01 | 10.58 | x | x | x |
| 397.0872 | 1.27 | 2.30E+01 | 1.68E+01 | 2.42E+01 | 2.64E+01 | 2.30E+01 | 2.53E+01 | 2.13E+01 | 2.14E+01 | 2.22E+01 | 1.81E+01 | 2.59E+01 | 1.51E+01 | 2.00E+01 | 2.18E+01 | 2.02E+01 | 2.14E+01 | 2.07E+01 | 14.11 | x | x | x |
| 427.1374 | 1.27 | 1.32E+02 | 1.07E+02 | 1.13E+02 | 1.03E+02 | 9.80E+01 | 9.42E+01 | 8.55E+01 | 6.99E+01 | 8.51E+01 | 7.33E+01 | 1.34E+02 | 9.18E+01 | 8.30E+01 | 8.71E+01 | 7.21E+01 | 8.34E+01 | 7.58E+01 | 20.6 |  |  | x |
| 116.0500 | 1.28 | 8.62E+00 | 5.69E+00 | 7.74E+00 | 6.45E+00 | 9.48E+00 | 8.55E+00 | 1.00E+01 | 7.36E+00 | 1.14E+01 | 9.33E+00 | 1.45E+01 | 1.03E+01 | 1.08E+01 | 1.42E+01 | 7.11E+00 | 9.57E+00 | 1.35E+01 | 26.72 |  |  | x |
| 198.1594 | 1.28 | 2.36E+01 | 2.13E+01 | 2.72E+01 | 2.56E+01 | 2.94E+01 | 3.44E+01 | 3.12E+01 | 3.19E+01 | 2.98E+01 | 2.64E+01 | 4.51E+01 | 3.97E+01 | 2.83E+01 | 3.33E+01 | 2.89E+01 | 3.06E+01 | 3.52E+01 | 18.93 |  | x | x |
| 300.0063 | 1.28 | 1.73E+01 | 1.46E+01 | 1.71E+01 | 1.87E+01 | 1.82E+01 | 1.67E+01 | 1.74E+01 | 1.66E+01 | 2.08E+01 | 1.66E+01 | 2.62E+01 | 1.25E+01 | 1.78E+01 | 1.81E+01 | 1.33E+01 | 1.55E+01 | 1.46E+01 | 17.99 |  | x | x |
| 377.0041 | 1.28 | 5.34E+01 | 4.48E+01 | 4.90E+01 | 3.99E+01 | 3.93E+01 | 4.68E+01 | 3.33E+01 | 3.53E+01 | 3.91E+01 | 3.62E+01 | 4.28E+01 | 2.72E+01 | 3.15E+01 | 3.78E+01 | 2.20E+01 | 3.21E+01 | 3.01E+01 | 21.56 |  |  | x |
| 391.0182 | 1.28 | 1.33E+02 | 9.87E+01 | 9.68E+01 | 9.71E+01 | 8.92E+01 | 8.66E+01 | 7.65E+01 | 6.80E+01 | 8.24E+01 | 7.62E+01 | 9.65E+01 | 6.65E+01 | 7.44E+01 | 6.87E+01 | 5.69E+01 | 6.01E+01 | 7.16E+01 | 22.62 |  |  | x |
| 471.0702 | 1.28 | 2.75E+02 | 2.20E+02 | 2.33E+02 | 1.91E+02 | 1.91E+02 | 1.82E+02 | 1.64E+02 | 1.72E+02 | 1.70E+02 | 1.54E+02 | 2.14E+02 | 1.61E+02 | 1.70E+02 | 1.63E+02 | 1.54E+02 | 1.54E+02 | 1.51E+02 | 18.6 |  | x | x |
| 139.0507 | 1.29 | 2.14E+01 | 1.77E+01 | 2.30E+01 | 2.21E+01 | 2.46E+01 | 2.64E+01 | 2.63E+01 | 3.31E+01 | 2.88E+01 | 2.55E+01 | 4.40E+01 | 3.46E+01 | 3.03E+01 | 3.02E+01 | 3.36E+01 | 3.06E+01 | 2.86E+01 | 21.76 |  |  | x |
| 396.0702 | 1.29 | 6.03E+01 | 5.30E+01 | 6.47E+01 | 6.56E+01 | 6.32E+01 | 7.02E+01 | 6.44E+01 | 6.58E+01 | 6.41E+01 | 5.78E+01 | 8.94E+01 | 5.83E+01 | 5.58E+01 | 5.42E+01 | 5.63E+01 | 6.55E+01 | 5.45E+01 | 13.69 | x | x | x |
| 431.0985 | 1.29 | 4.24E+02 | 3.88E+02 | 4.35E+02 | 4.18E+02 | 4.16E+02 | 4.25E+02 | 3.64E+02 | 4.06E+02 | 3.92E+02 | 3.35E+02 | 3.06E+02 | 3.69E+02 | 3.37E+02 | 3.37E+02 | 3.56E+02 | 3.29E+02 | 3.32E+02 | 10.95 | x | x | x |
| 434.0349 | 1.29 | 4.99E+01 | 4.33E+01 | 4.86E+01 | 3.25E+01 | 3.16E+01 | 3.04E+01 | 3.24E+01 | 2.92E+01 | 3.57E+01 | 2.63E+01 | 2.76E+01 | 2.29E+01 | 2.94E+01 | 3.23E+01 | 2.26E+01 | 3.12E+01 | 2.53E+01 | 24.63 |  |  | x |
| 469.0546 | 1.29 | 2.38E+02 | 2.32E+02 | 2.60E+02 | 2.51E+02 | 2.56E+02 | 2.44E+02 | 2.21E+02 | 2.30E+02 | 2.40E+02 | 1.97E+02 | 1.60E+02 | 1.98E+02 | 2.11E+02 | 2.17E+02 | 2.07E+02 | 2.02E+02 | 2.02E+02 | 11.8 | x | x | x |
| 240.0968 | 1.30 | 7.55E+00 | 5.91E+00 | 8.77E+00 | 9.48E+00 | 9.09E+00 | 1.19E+01 | 1.28E+01 | 1.18E+01 | 1.30E+01 | 1.07E+01 | 1.36E+01 | 1.91E+01 | 8.68E+00 | 1.00E+01 | 1.45E+01 | 1.28E+01 | 8.31E+00 | 28.6 |  |  | x |
| 294.0255 | 1.30 | 3.42E+02 | 2.90E+02 | 3.12E+02 | 3.13E+02 | 3.22E+02 | 3.26E+02 | 3.27E+02 | 2.98E+02 | 3.44E+02 | 2.92E+02 | 4.12E+02 | 3.03E+02 | 3.08E+02 | 3.17E+02 | 2.67E+02 | 3.05E+02 | 3.18E+02 | 9.72 | x | x | x |
| 286.0817 | 1.30 | 1.84E+01 | 9.69E+00 | 1.39E+01 | 1.36E+01 | 1.25E+01 | 1.73E+01 | 1.36E+01 | 1.56E+01 | 1.81E+01 | 8.87E+00 | 2.48E+01 | 1.82E+01 | 1.48E+01 | 1.45E+01 | 1.61E+01 | 1.65E+01 | 1.44E+01 | 23.66 |  |  | x |
| 329.0591 | 1.30 | 5.14E+01 | 5.39E+01 | 5.99E+01 | 6.05E+01 | 6.56E+01 | 6.37E+01 | 6.69E+01 | 6.74E+01 | 6.28E+01 | 5.26E+01 | 9.40E+01 | 6.08E+01 | 6.60E+01 | 6.19E+01 | 6.12E+01 | 6.05E+01 | 6.52E+01 | 14.64 | x | x | x |
| 362.9898 | 1.30 | 8.62E+02 | 7.18E+02 | 8.23E+02 | 7.17E+02 | 7.58E+02 | 7.98E+02 | 7.52E+02 | 7.01E+02 | 7.73E+02 | 6.87E+02 | 1.10E+03 | 6.67E+02 | 6.92E+02 | 6.92E+02 | 5.73E+02 | 6.89E+02 | 6.80E+02 | 15.07 |  | x | x |
| 372.2383 | 1.30 | 2.17E+02 | 2.21E+02 | 2.21E+02 | 2.74E+02 | 2.54E+02 | 2.74E+02 | 2.18E+02 | 2.85E+02 | 2.22E+02 | 1.97E+02 | 2.34E+02 | 1.99E+02 | 2.45E+02 | 2.00E+02 | 4.35E+02 | 2.44E+02 | 2.37E+02 | 22.58 |  |  | x |
| 461.1089 | 1.30 | 8.32E+01 | 7.19E+01 | 7.64E+01 | 7.74E+01 | 7.68E+01 | 8.77E+01 | 7.25E+01 | 7.83E+01 | 7.86E+01 | 6.35E+01 | 6.32E+01 | 7.04E+01 | 6.56E+01 | 6.70E+01 | 6.43E+01 | 6.72E+01 | 5.63E+01 | 11.41 | x | x | x |
| 461.1792 | 1.30 | 6.92E+01 | 5.28E+01 | 6.06E+01 | 4.55E+01 | 4.34E+01 | 5.26E+01 | 5.35E+01 | 4.09E+01 | 4.85E+01 | 3.96E+01 | 4.12E+01 | 3.73E+01 | 4.02E+01 | 3.71E+01 | 3.47E+01 | 4.18E+01 | 3.93E+01 | 20.25 |  |  | x |
| 499.0652 | 1.30 | 2.08E+02 | 1.74E+02 | 1.88E+02 | 1.53E+02 | 1.32E+02 | 1.38E+02 | 1.30E+02 | 1.26E+02 | 1.35E+02 | 1.04E+02 | 8.29E+01 | 1.12E+02 | 1.05E+02 | 1.10E+02 | 9.54E+01 | 9.92E+01 | 1.10E+02 | 26.37 |  |  | x |
| 277.1047 | 1.31 | 7.70E+00 | 6.47E+00 | 8.88E+00 | 8.74E+00 | 1.21E+01 | 9.08E+00 | 8.21E+00 | 7.39E+00 | 1.24E+01 | 6.53E+00 | 1.04E+01 | 8.70E+00 | 6.71E+00 | 8.97E+00 | 8.64E+00 | 7.24E+00 | 7.98E+00 | 19.92 |  | x | x |
| 406.0500 | 1.31 | 9.69E+01 | 7.09E+01 | 8.84E+01 | 6.30E+01 | 6.70E+01 | 7.05E+01 | 6.57E+01 | 5.13E+01 | 6.58E+01 | 5.87E+01 | 6.04E+01 | 6.01E+01 | 6.40E+01 | 5.85E+01 | 3.97E+01 | 6.10E+01 | 6.04E+01 | 19.79 |  | x | x |
| 469.0544 | 1.31 | 7.27E+01 | 6.48E+01 | 7.11E+01 | 6.28E+01 | 5.66E+01 | 5.05E+01 | 5.18E+01 | 5.70E+01 | 5.24E+01 | 4.20E+01 | 2.66E+01 | 5.40E+01 | 5.13E+01 | 3.76E+01 | 5.04E+01 | 4.39E+01 | 4.73E+01 | 22.14 |  |  | x |
| 160.1326 | 1.32 | 2.26E+02 | 2.07E+02 | 2.31E+02 | 2.51E+02 | 2.86E+02 | 2.93E+02 | 2.93E+02 | 3.08E+02 | 3.07E+02 | 2.81E+02 | 4.31E+02 | 3.23E+02 | 3.08E+02 | 3.07E+02 | 3.41E+02 | 3.20E+02 | 3.33E+02 | 17.4 |  | x | x |
| 230.0352 | 1.32 | 8.92E+01 | 8.14E+01 | 8.71E+01 | 9.03E+01 | 8.63E+01 | 9.04E+01 | 8.47E+01 | 8.73E+01 | 8.35E+01 | 7.29E+01 | 1.18E+02 | 6.68E+01 | 7.94E+01 | 6.95E+01 | 7.72E+01 | 7.43E+01 | 7.87E+01 | 13.87 | x | x | x |
| 232.0331 | 1.32 | 2.85E+01 | 2.63E+01 | 3.48E+01 | 3.12E+01 | 2.85E+01 | 3.28E+01 | 3.00E+01 | 2.78E+01 | 2.69E+01 | 2.41E+01 | 4.46E+01 | 2.28E+01 | 2.55E+01 | 2.29E+01 | 2.94E+01 | 2.35E+01 | 2.62E+01 | 18.71 |  | x | x |
| 420.0286 | 1.32 | 1.45E+02 | 1.27E+02 | 1.28E+02 | 1.08E+02 | 1.08E+02 | 1.03E+02 | 1.04E+02 | 1.00E+02 | 1.17E+02 | 9.06E+01 | 1.23E+02 | 9.28E+01 | 9.65E+01 | 9.64E+01 | 8.08E+01 | 9.55E+01 | 9.94E+01 | 15.36 |  | x | x |
| 459.1074 | 1.32 | 1.24E+01 | 1.46E+01 | 1.80E+01 | 1.33E+01 | 1.37E+01 | 1.47E+01 | 1.20E+01 | 1.16E+01 | 1.49E+01 | 8.37E+00 | 1.37E+01 | 1.31E+01 | 1.34E+01 | 1.31E+01 | 1.28E+01 | 1.04E+01 | 1.13E+01 | 16.02 |  | x | x |
| 94.0655 | 1.33 | 2.48E+02 | 2.36E+02 | 2.60E+02 | 2.62E+02 | 3.06E+02 | 3.24E+02 | 3.09E+02 | 3.04E+02 | 3.50E+02 | 2.94E+02 | 4.86E+02 | 3.47E+02 | 3.40E+02 | 3.45E+02 | 2.83E+02 | 3.25E+02 | 3.41E+02 | 18.06 |  | x | x |
| 137.0723 | 1.33 | 5.00E+03 | 4.51E+03 | 5.09E+03 | 5.37E+03 | 5.80E+03 | 6.22E+03 | 6.14E+03 | 5.98E+03 | 6.25E+03 | 5.61E+03 | 8.13E+03 | 6.06E+03 | 6.07E+03 | 6.08E+03 | 5.66E+03 | 5.95E+03 | 6.25E+03 | 12.96 | x | x | x |
| 137.1289 | 1.33 | 5.90E+01 | 5.23E+01 | 5.74E+01 | 6.26E+01 | 6.62E+01 | 7.31E+01 | 7.11E+01 | 7.17E+01 | 7.51E+01 | 6.38E+01 | 9.88E+01 | 6.81E+01 | 7.00E+01 | 6.84E+01 | 6.23E+01 | 6.69E+01 | 7.12E+01 | 14.61 | x | x | x |
| 309.0463 | 1.33 | 5.12E+01 | 4.43E+01 | 4.54E+01 | 5.03E+01 | 4.81E+01 | 5.27E+01 | 5.10E+01 | 4.97E+01 | 5.18E+01 | 4.29E+01 | 4.83E+01 | 4.00E+01 | 4.57E+01 | 4.77E+01 | 4.62E+01 | 4.71E+01 | 4.21E+01 | 7.66 | x | x | x |
| 380.0363 | 1.33 | 1.88E+01 | 1.77E+01 | 2.30E+01 | 1.97E+01 | 1.83E+01 | 1.71E+01 | 1.98E+01 | 2.07E+01 | 2.17E+01 | 1.96E+01 | 2.81E+01 | 1.66E+01 | 1.78E+01 | 1.77E+01 | 1.96E+01 | 2.00E+01 | 2.06E+01 | 13.71 | x | x | x |
| 381.0592 | 1.33 | 5.42E+01 | 4.77E+01 | 5.70E+01 | 6.60E+01 | 6.64E+01 | 6.13E+01 | 6.48E+01 | 6.98E+01 | 6.01E+01 | 5.93E+01 | 6.52E+01 | 5.25E+01 | 6.08E+01 | 4.78E+01 | 5.41E+01 | 4.98E+01 | 5.64E+01 | 11.62 | x | x | x |
| 132.0449 | 1.34 | 1.26E+01 | 1.07E+01 | 1.20E+01 | 9.16E+00 | 1.47E+01 | 1.46E+01 | 1.69E+01 | 1.47E+01 | 1.82E+01 | 1.28E+01 | 2.17E+01 | 1.37E+01 | 1.55E+01 | 1.39E+01 | 1.33E+01 | 1.53E+01 | 1.47E+01 | 19.97 |  | x | x |
| 137.1720 | 1.34 | 1.94E+02 | 1.76E+02 | 2.01E+02 | 2.13E+02 | 2.24E+02 | 2.39E+02 | 2.31E+02 | 2.30E+02 | 2.44E+02 | 2.17E+02 | 3.25E+02 | 2.46E+02 | 2.44E+02 | 2.46E+02 | 2.22E+02 | 2.41E+02 | 2.52E+02 | 13.68 | x | x | x |
| 162.1126 | 1.34 | 4.49E+01 | 4.23E+01 | 4.81E+01 | 5.79E+01 | 6.71E+01 | 6.75E+01 | 6.81E+01 | 7.14E+01 | 6.58E+01 | 5.76E+01 | 7.37E+01 | 5.47E+01 | 6.80E+01 | 6.36E+01 | 8.61E+01 | 7.39E+01 | 7.43E+01 | 18.16 |  | x | x |
| 178.0506 | 1.34 | 3.20E+01 | 2.61E+01 | 2.96E+01 | 2.70E+01 | 2.94E+01 | 2.96E+01 | 3.29E+01 | 3.51E+01 | 3.42E+01 | 2.45E+01 | 4.90E+01 | 3.63E+01 | 3.12E+01 | 3.57E+01 | 3.21E+01 | 3.44E+01 | 3.17E+01 | 16.86 |  | x | x |
| 206.0457 | 1.34 | 1.49E+02 | 1.36E+02 | 1.58E+02 | 1.51E+02 | 1.79E+02 | 1.89E+02 | 2.00E+02 | 2.10E+02 | 2.07E+02 | 1.77E+02 | 3.05E+02 | 2.15E+02 | 2.04E+02 | 2.12E+02 | 1.91E+02 | 2.08E+02 | 2.21E+02 | 19.8 |  | x | x |
| 309.0480 | 1.34 | 8.13E+00 | 5.39E+00 | 7.46E+00 | 7.11E+00 | 9.11E+00 | 9.46E+00 | 9.61E+00 | 8.16E+00 | 5.40E+00 | 7.36E+00 | 6.19E+00 | 8.81E+00 | 7.29E+00 | 7.54E+00 | 5.16E+00 | 6.95E+00 | 7.35E+00 | 18.39 |  | x | x |
| 343.0196 | 1.34 | 5.23E+01 | 4.42E+01 | 4.19E+01 | 4.65E+01 | 4.18E+01 | 3.77E+01 | 3.39E+01 | 3.62E+01 | 3.24E+01 | 3.39E+01 | 2.74E+01 | 3.16E+01 | 3.82E+01 | 3.24E+01 | 2.34E+01 | 3.68E+01 | 2.86E+01 | 20.13 |  |  | x |
| 350.0864 | 1.34 | 1.30E+02 | 1.08E+02 | 1.32E+02 | 1.33E+02 | 1.25E+02 | 1.36E+02 | 1.24E+02 | 1.22E+02 | 1.22E+02 | 1.16E+02 | 1.03E+02 | 1.20E+02 | 1.07E+02 | 1.05E+02 | 1.07E+02 | 9.83E+01 | 1.05E+02 | 10.1 | x | x | x |
| 378.9100 | 1.34 | 4.21E+01 | 3.14E+01 | 3.04E+01 | 2.46E+01 | 2.19E+01 | 1.86E+01 | 2.23E+01 | 1.82E+01 | 2.80E+01 | 2.33E+01 | 2.58E+01 | 1.28E+01 | 2.41E+01 | 2.37E+01 | 1.17E+01 | 2.01E+01 | 2.40E+01 | 29.98 |  |  | x |
| 394.0902 | 1.34 | 2.24E+02 | 2.01E+02 | 2.30E+02 | 2.13E+02 | 2.05E+02 | 2.22E+02 | 2.00E+02 | 2.07E+02 | 2.10E+02 | 1.92E+02 | 1.99E+02 | 1.97E+02 | 1.97E+02 | 1.85E+02 | 2.02E+02 | 1.76E+02 | 1.82E+02 | 7.25 | x | x | x |
| 181.1070 | 1.35 | 1.05E+02 | 1.02E+02 | 1.14E+02 | 1.23E+02 | 1.36E+02 | 1.40E+02 | 1.27E+02 | 1.38E+02 | 1.45E+02 | 1.21E+02 | 1.82E+02 | 1.33E+02 | 1.35E+02 | 1.31E+02 | 1.59E+02 | 1.37E+02 | 1.37E+02 | 14.22 | x | x | x |
| 207.1128 | 1.35 | 3.37E+01 | 3.35E+01 | 3.74E+01 | 5.10E+01 | 4.78E+01 | 5.42E+01 | 4.23E+01 | 4.75E+01 | 4.23E+01 | 4.07E+01 | 6.10E+01 | 4.23E+01 | 4.51E+01 | 3.74E+01 | 5.69E+01 | 4.81E+01 | 4.88E+01 | 17.24 |  | x | x |
| 226.1080 | 1.35 | 1.28E+01 | 1.19E+01 | 1.40E+01 | 1.37E+01 | 1.44E+01 | 1.33E+01 | 1.31E+01 | 1.18E+01 | 1.45E+01 | 1.04E+01 | 1.06E+01 | 1.69E+01 | 1.21E+01 | 1.31E+01 | 1.93E+01 | 1.35E+01 | 1.69E+01 | 16.8 |  | x | x |
| 324.0966 | 1.35 | 1.15E+01 | 8.73E+00 | 7.63E+00 | 1.34E+01 | 1.08E+01 | 1.14E+01 | 7.72E+00 | 9.68E+00 | 7.11E+00 | 8.89E+00 | 1.13E+01 | 6.06E+00 | 1.19E+01 | 9.17E+00 | 1.06E+01 | 9.18E+00 | 6.91E+00 | 21.5 |  |  | x |
| 336.0707 | 1.35 | 1.42E+02 | 1.30E+02 | 1.44E+02 | 1.58E+02 | 1.51E+02 | 1.71E+02 | 1.45E+02 | 1.46E+02 | 1.59E+02 | 1.28E+02 | 1.46E+02 | 1.26E+02 | 1.33E+02 | 1.31E+02 | 1.15E+02 | 1.27E+02 | 1.30E+02 | 10.33 | x | x | x |
| 367.0435 | 1.35 | 2.11E+01 | 1.81E+01 | 1.51E+01 | 1.97E+01 | 2.00E+01 | 1.71E+01 | 1.52E+01 | 1.54E+01 | 9.76E+00 | 1.88E+01 | 1.09E+01 | 1.59E+01 | 1.62E+01 | 1.72E+01 | 1.66E+01 | 1.50E+01 | 1.52E+01 | 17.98 |  | x | x |
| 424.1017 | 1.35 | 2.73E+01 | 2.27E+01 | 2.46E+01 | 2.38E+01 | 1.97E+01 | 2.47E+01 | 1.86E+01 | 1.92E+01 | 2.23E+01 | 1.96E+01 | 2.34E+01 | 1.82E+01 | 1.72E+01 | 1.51E+01 | 2.10E+01 | 1.44E+01 | 2.13E+01 | 16.88 |  | x | x |
| 511.9991 | 1.35 | 4.34E+02 | 3.39E+02 | 3.56E+02 | 3.12E+02 | 3.07E+02 | 3.20E+02 | 2.82E+02 | 3.02E+02 | 3.18E+02 | 2.47E+02 | 1.04E+02 | 2.84E+02 | 2.87E+02 | 2.69E+02 | 2.98E+02 | 2.73E+02 | 2.81E+02 | 21.99 |  |  | x |
| 513.9966 | 1.35 | 3.16E+02 | 2.59E+02 | 2.59E+02 | 2.20E+02 | 2.19E+02 | 2.23E+02 | 1.99E+02 | 2.12E+02 | 2.22E+02 | 1.89E+02 | 6.81E+01 | 2.02E+02 | 1.99E+02 | 2.00E+02 | 1.98E+02 | 1.93E+02 | 1.93E+02 | 23.29 |  |  | x |
| 258.2068 | 1.36 | 8.33E+00 | 8.79E+00 | 9.80E+00 | 1.03E+01 | 1.15E+01 | 9.00E+00 | 1.18E+01 | 1.25E+01 | 1.19E+01 | 6.36E+00 | 1.24E+01 | 1.17E+01 | 8.44E+00 | 1.15E+01 | 1.34E+01 | 1.25E+01 | 1.21E+01 | 18.13 |  | x | x |
| 259.2019 | 1.36 | 1.39E+02 | 1.38E+02 | 1.45E+02 | 1.59E+02 | 1.68E+02 | 1.75E+02 | 1.71E+02 | 1.73E+02 | 1.78E+02 | 1.49E+02 | 2.57E+02 | 1.77E+02 | 1.66E+02 | 1.65E+02 | 1.70E+02 | 1.70E+02 | 1.73E+02 | 15.44 |  | x | x |
| 271.0608 | 1.36 | 6.44E+01 | 5.88E+01 | 6.56E+01 | 6.30E+01 | 7.08E+01 | 7.31E+01 | 6.21E+01 | 6.80E+01 | 7.21E+01 | 5.87E+01 | 1.07E+02 | 7.39E+01 | 6.35E+01 | 6.53E+01 | 7.07E+01 | 6.64E+01 | 6.88E+01 | 15.72 |  | x | x |
| 372.2384 | 1.36 | 2.63E+02 | 2.35E+02 | 2.55E+02 | 2.21E+02 | 2.28E+02 | 2.49E+02 | 2.31E+02 | 2.33E+02 | 2.47E+02 | 2.07E+02 | 1.45E+02 | 2.53E+02 | 2.16E+02 | 2.13E+02 | 2.56E+02 | 2.06E+02 | 2.22E+02 | 12.28 | x | x | x |
| 392.0746 | 1.36 | 4.16E+01 | 3.71E+01 | 4.84E+01 | 3.74E+01 | 3.79E+01 | 4.06E+01 | 3.63E+01 | 4.02E+01 | 4.55E+01 | 3.01E+01 | 2.87E+01 | 3.31E+01 | 3.35E+01 | 3.42E+01 | 3.69E+01 | 3.22E+01 | 3.70E+01 | 13.85 | x | x | x |
| 412.0653 | 1.36 | 6.69E+01 | 5.43E+01 | 6.21E+01 | 5.54E+01 | 5.83E+01 | 6.02E+01 | 5.38E+01 | 5.93E+01 | 6.44E+01 | 5.04E+01 | 4.94E+01 | 5.05E+01 | 5.77E+01 | 4.77E+01 | 4.86E+01 | 4.85E+01 | 5.77E+01 | 10.61 | x | x | x |
| 426.0924 | 1.36 | 4.13E+01 | 3.86E+01 | 3.18E+01 | 4.80E+01 | 4.09E+01 | 3.56E+01 | 2.72E+01 | 2.72E+01 | 3.29E+01 | 2.96E+01 | 2.38E+01 | 3.28E+01 | 3.19E+01 | 2.29E+01 | 4.73E+01 | 2.79E+01 | 3.05E+01 | 22.33 |  |  | x |
| 485.0495 | 1.36 | 7.00E+01 | 5.95E+01 | 5.20E+01 | 5.25E+01 | 5.23E+01 | 4.51E+01 | 4.70E+01 | 4.88E+01 | 5.32E+01 | 4.07E+01 | 2.18E+01 | 3.44E+01 | 4.70E+01 | 3.39E+01 | 3.84E+01 | 4.37E+01 | 4.67E+01 | 23.53 |  |  | x |
| 493.2828 | 1.36 | 7.41E+01 | 6.51E+01 | 5.94E+01 | 4.23E+01 | 5.40E+01 | 4.20E+01 | 4.08E+01 | 4.18E+01 | 4.57E+01 | 3.35E+01 | 3.47E+01 | 5.00E+01 | 4.56E+01 | 4.16E+01 | 7.00E+01 | 3.70E+01 | 3.38E+01 | 26.46 |  |  | x |
| 119.0358 | 1.37 | 1.71E+01 | 1.28E+01 | 1.47E+01 | 1.55E+01 | 1.93E+01 | 1.95E+01 | 1.80E+01 | 1.76E+01 | 1.77E+01 | 1.53E+01 | 2.25E+01 | 1.90E+01 | 1.68E+01 | 1.70E+01 | 1.77E+01 | 1.39E+01 | 1.93E+01 | 13.69 | x | x | x |
| 227.1371 | 1.37 | 1.83E+01 | 1.52E+01 | 2.09E+01 | 2.36E+01 | 2.04E+01 | 1.97E+01 | 2.18E+01 | 1.85E+01 | 2.23E+01 | 1.81E+01 | 2.71E+01 | 1.83E+01 | 1.94E+01 | 2.23E+01 | 1.65E+01 | 2.03E+01 | 1.76E+01 | 14.3 | x | x | x |
| 311.1612 | 1.37 | 4.25E+02 | 4.00E+02 | 4.38E+02 | 4.66E+02 | 4.83E+02 | 4.99E+02 | 4.87E+02 | 5.20E+02 | 5.16E+02 | 4.66E+02 | 3.53E+02 | 5.57E+02 | 4.98E+02 | 4.94E+02 | 5.41E+02 | 5.02E+02 | 5.01E+02 | 10.69 | x | x | x |
| 319.9244 | 1.37 | 2.35E+01 | 1.76E+01 | 2.44E+01 | 2.27E+01 | 2.41E+01 | 2.78E+01 | 2.63E+01 | 2.40E+01 | 1.93E+01 | 2.11E+01 | 3.34E+01 | 2.41E+01 | 2.65E+01 | 2.37E+01 | 2.13E+01 | 2.51E+01 | 2.49E+01 | 14.6 | x | x | x |
| 517.1482 | 1.37 | 2.56E+01 | 2.32E+01 | 2.30E+01 | 1.87E+01 | 1.23E+01 | 1.68E+01 | 1.96E+01 | 1.87E+01 | 1.80E+01 | 1.52E+01 | 7.30E+00 | 1.47E+01 | 1.70E+01 | 1.46E+01 | 1.76E+01 | 1.71E+01 | 1.29E+01 | 25.59 |  |  | x |
| 220.0967 | 1.38 | 2.66E+01 | 2.06E+01 | 2.50E+01 | 2.82E+01 | 3.20E+01 | 3.41E+01 | 3.17E+01 | 2.95E+01 | 3.13E+01 | 2.61E+01 | 2.52E+01 | 3.96E+01 | 2.90E+01 | 2.44E+01 | 3.52E+01 | 2.74E+01 | 2.73E+01 | 15.89 |  | x | x |
| 257.9603 | 1.38 | 4.15E+00 | 2.62E+00 | 3.40E+00 | 3.83E+00 | 4.26E+00 | 5.43E+00 | 5.51E+00 | 4.95E+00 | 4.72E+00 | 5.18E+00 | 6.18E+00 | 2.31E+00 | 4.01E+00 | 1.99E+00 | 3.42E+00 | 4.09E+00 | 4.35E+00 | 27.86 |  |  | x |
| 281.1509 | 1.38 | 4.68E+01 | 4.28E+01 | 5.26E+01 | 4.90E+01 | 5.09E+01 | 5.80E+01 | 5.51E+01 | 4.98E+01 | 5.59E+01 | 4.06E+01 | 6.31E+01 | 6.86E+01 | 5.11E+01 | 4.10E+01 | 5.64E+01 | 4.35E+01 | 4.66E+01 | 15.07 |  | x | x |
| 440.1064 | 1.38 | 2.40E+01 | 2.13E+01 | 2.19E+01 | 1.71E+01 | 1.59E+01 | 1.85E+01 | 1.38E+01 | 1.25E+01 | 1.47E+01 | 1.41E+01 | 1.31E+01 | 1.08E+01 | 1.19E+01 | 1.20E+01 | 1.52E+01 | 1.22E+01 | 9.91E+00 | 26.73 |  |  | x |
| 263.0972 | 1.39 | 1.54E+02 | 1.51E+02 | 1.57E+02 | 1.51E+02 | 1.71E+02 | 1.59E+02 | 1.52E+02 | 1.60E+02 | 1.49E+02 | 1.29E+02 | 1.63E+02 | 1.67E+02 | 1.49E+02 | 1.36E+02 | 1.79E+02 | 1.46E+02 | 1.51E+02 | 7.75 | x | x | x |
| 160.1300 | 1.39 | 3.35E+01 | 3.14E+01 | 3.40E+01 | 3.76E+01 | 4.02E+01 | 4.83E+01 | 4.32E+01 | 4.00E+01 | 5.40E+01 | 4.40E+01 | 6.81E+01 | 5.98E+01 | 4.22E+01 | 5.13E+01 | 4.43E+01 | 3.96E+01 | 4.67E+01 | 21.47 |  |  | x |
| 160.1112 | 1.39 | 1.54E+01 | 1.30E+01 | 1.89E+01 | 1.31E+01 | 2.28E+01 | 2.14E+01 | 1.80E+01 | 2.44E+01 | 2.86E+01 | 1.68E+01 | 2.47E+01 | 1.09E+01 | 1.83E+01 | 2.38E+01 | 1.88E+01 | 1.81E+01 | 2.12E+01 | 24.51 |  |  | x |
| 245.1843 | 1.39 | 2.51E+01 | 2.45E+01 | 3.44E+01 | 3.34E+01 | 3.91E+01 | 4.32E+01 | 3.72E+01 | 3.00E+01 | 3.59E+01 | 3.02E+01 | 5.70E+01 | 5.83E+01 | 3.01E+01 | 2.71E+01 | 2.90E+01 | 2.98E+01 | 2.61E+01 | 28.8 |  |  | x |
| 289.0481 | 1.39 | 7.04E+02 | 6.44E+02 | 7.09E+02 | 7.72E+02 | 8.46E+02 | 8.69E+02 | 8.05E+02 | 8.76E+02 | 8.59E+02 | 7.33E+02 | 7.12E+02 | 1.04E+03 | 7.85E+02 | 8.02E+02 | 1.42E+03 | 7.80E+02 | 8.02E+02 | 21.06 |  |  | x |
| 291.0456 | 1.39 | 3.24E+02 | 2.99E+02 | 3.31E+02 | 3.38E+02 | 3.95E+02 | 3.94E+02 | 3.70E+02 | 4.17E+02 | 3.86E+02 | 3.35E+02 | 3.87E+02 | 5.13E+02 | 3.60E+02 | 3.65E+02 | 7.24E+02 | 3.44E+02 | 3.61E+02 | 25.1 |  |  | x |
| 307.0443 | 1.39 | 5.04E+01 | 4.74E+01 | 5.39E+01 | 5.70E+01 | 6.51E+01 | 7.23E+01 | 6.54E+01 | 6.51E+01 | 6.34E+01 | 5.91E+01 | 7.09E+01 | 6.02E+01 | 6.70E+01 | 6.70E+01 | 6.12E+01 | 6.79E+01 | 6.39E+01 | 11.06 | x | x | x |
| 378.0590 | 1.39 | 1.75E+02 | 1.74E+02 | 1.93E+02 | 2.29E+02 | 2.38E+02 | 2.50E+02 | 2.33E+02 | 2.26E+02 | 2.42E+02 | 1.91E+02 | 7.39E+01 | 2.57E+02 | 2.34E+02 | 1.95E+02 | 2.27E+02 | 1.93E+02 | 2.11E+02 | 20.7 |  |  | x |
| 386.0440 | 1.39 | 1.88E+02 | 1.79E+02 | 1.87E+02 | 1.62E+02 | 1.53E+02 | 1.76E+02 | 1.66E+02 | 1.56E+02 | 1.70E+02 | 1.60E+02 | 1.32E+02 | 1.37E+02 | 1.66E+02 | 1.50E+02 | 1.35E+02 | 1.65E+02 | 1.73E+02 | 10.41 | x | x | x |
| 416.0152 | 1.39 | 3.25E+02 | 2.61E+02 | 2.84E+02 | 2.71E+02 | 2.67E+02 | 2.67E+02 | 2.53E+02 | 2.29E+02 | 2.74E+02 | 2.41E+02 | 4.93E+02 | 2.35E+02 | 2.60E+02 | 2.75E+02 | 2.19E+02 | 2.50E+02 | 2.55E+02 | 22.39 |  |  | x |
| 425.0854 | 1.39 | 6.71E+01 | 4.98E+01 | 7.48E+01 | 5.94E+01 | 6.04E+01 | 7.56E+01 | 6.53E+01 | 6.52E+01 | 7.37E+01 | 5.84E+01 | 3.78E+01 | 5.78E+01 | 5.92E+01 | 5.83E+01 | 4.00E+01 | 5.78E+01 | 5.51E+01 | 17.71 |  | x | x |
| 114.0663 | 1.40 | 3.55E+01 | 3.36E+01 | 4.08E+01 | 3.91E+01 | 4.61E+01 | 4.95E+01 | 4.82E+01 | 4.74E+01 | 5.35E+01 | 4.44E+01 | 6.63E+01 | 4.73E+01 | 5.30E+01 | 5.17E+01 | 5.35E+01 | 5.16E+01 | 5.00E+01 | 16.22 |  | x | x |
| 246.8927 | 1.40 | 8.54E+01 | 5.78E+01 | 7.21E+01 | 6.14E+01 | 5.86E+01 | 6.89E+01 | 7.43E+01 | 5.92E+01 | 9.30E+01 | 7.70E+01 | 6.89E+01 | 6.40E+01 | 7.77E+01 | 9.23E+01 | 2.95E+01 | 8.22E+01 | 7.66E+01 | 21.65 |  |  | x |
| 268.8690 | 1.40 | 1.39E+01 | 1.32E+01 | 1.33E+01 | 1.18E+01 | 1.37E+01 | 1.32E+01 | 1.22E+01 | 1.11E+01 | 1.67E+01 | 1.32E+01 | 1.88E+01 | 1.41E+01 | 1.48E+01 | 1.47E+01 | 1.53E+01 | 1.19E+01 | 1.50E+01 | 13.68 | x | x | x |
| 344.9999 | 1.40 | 2.56E+01 | 2.22E+01 | 2.89E+01 | 2.19E+01 | 1.87E+01 | 1.78E+01 | 1.94E+01 | 1.64E+01 | 2.40E+01 | 1.84E+01 | 2.47E+01 | 1.10E+01 | 1.90E+01 | 1.85E+01 | 7.77E+00 | 1.38E+01 | 1.86E+01 | 27.39 |  |  | x |
| 377.9665 | 1.40 | 3.33E+02 | 2.81E+02 | 3.14E+02 | 2.84E+02 | 2.80E+02 | 3.02E+02 | 3.10E+02 | 2.95E+02 | 3.21E+02 | 2.79E+02 | 9.51E+01 | 2.61E+02 | 2.88E+02 | 3.16E+02 | 2.46E+02 | 3.04E+02 | 2.79E+02 | 18.8 |  | x | x |
| 379.9646 | 1.40 | 5.34E+01 | 3.99E+01 | 4.64E+01 | 3.86E+01 | 3.80E+01 | 3.40E+01 | 4.00E+01 | 3.90E+01 | 4.23E+01 | 4.09E+01 | 1.58E+01 | 3.39E+01 | 3.55E+01 | 4.45E+01 | 3.11E+01 | 4.03E+01 | 3.60E+01 | 20.41 |  |  | x |
| 388.9255 | 1.40 | 1.03E+02 | 8.86E+01 | 9.82E+01 | 1.01E+02 | 1.01E+02 | 9.64E+01 | 8.55E+01 | 9.51E+01 | 9.20E+01 | 7.51E+01 | 3.37E+01 | 4.75E+01 | 7.92E+01 | 6.80E+01 | 3.67E+01 | 7.27E+01 | 7.95E+01 | 27.67 |  |  | x |
| 137.0724 | 1.41 | 1.86E+03 | 1.70E+03 | 1.90E+03 | 1.96E+03 | 2.12E+03 | 2.46E+03 | 2.37E+03 | 2.27E+03 | 2.44E+03 | 2.26E+03 | 2.59E+03 | 2.45E+03 | 2.40E+03 | 2.55E+03 | 2.21E+03 | 2.48E+03 | 2.57E+03 | 11.96 | x | x | x |
| 137.1722 | 1.41 | 4.16E+01 | 3.71E+01 | 4.11E+01 | 4.33E+01 | 4.69E+01 | 5.51E+01 | 5.16E+01 | 4.96E+01 | 5.33E+01 | 5.11E+01 | 6.06E+01 | 6.12E+01 | 5.46E+01 | 5.88E+01 | 4.61E+01 | 5.68E+01 | 5.95E+01 | 14.52 | x | x | x |
| 149.0464 | 1.41 | 1.59E+01 | 1.47E+01 | 1.61E+01 | 1.62E+01 | 2.00E+01 | 1.94E+01 | 2.01E+01 | 1.79E+01 | 1.93E+01 | 1.60E+01 | 2.12E+01 | 1.84E+01 | 2.02E+01 | 2.06E+01 | 1.68E+01 | 2.23E+01 | 2.13E+01 | 12.31 | x | x | x |
| 166.1837 | 1.41 | 3.23E+00 | 2.97E+00 | 3.39E+00 | 3.86E+00 | 4.26E+00 | 4.08E+00 | 4.91E+00 | 4.30E+00 | 4.18E+00 | 3.91E+00 | 3.15E+00 | 4.19E+00 | 3.72E+00 | 4.59E+00 | 4.59E+00 | 4.05E+00 | 4.02E+00 | 13.52 | x | x | x |
| 202.8919 | 1.41 | 3.62E+02 | 3.64E+02 | 4.12E+02 | 3.28E+02 | 3.81E+02 | 3.43E+02 | 2.53E+02 | 3.38E+02 | 2.53E+02 | 1.94E+02 | 2.40E+02 | 3.69E+02 | 2.38E+02 | 1.61E+02 | 4.07E+02 | 1.88E+02 | 2.49E+02 | 27.1 |  |  | x |
| 243.9448 | 1.41 | 4.26E+01 | 3.70E+01 | 4.36E+01 | 4.58E+01 | 4.79E+01 | 5.34E+01 | 4.70E+01 | 5.22E+01 | 5.52E+01 | 4.40E+01 | 2.98E+01 | 5.34E+01 | 5.21E+01 | 5.07E+01 | 4.73E+01 | 5.15E+01 | 5.94E+01 | 14.95 | x | x | x |
| 94.0656 | 1.42 | 1.41E+02 | 1.35E+02 | 1.46E+02 | 1.51E+02 | 1.72E+02 | 1.84E+02 | 1.76E+02 | 1.76E+02 | 1.84E+02 | 1.71E+02 | 2.71E+02 | 2.20E+02 | 1.78E+02 | 1.80E+02 | 1.71E+02 | 1.78E+02 | 1.88E+02 | 17.64 |  | x | x |
| 137.1303 | 1.42 | 1.44E+01 | 1.13E+01 | 1.23E+01 | 1.14E+01 | 1.41E+01 | 1.64E+01 | 1.72E+01 | 1.40E+01 | 1.50E+01 | 1.47E+01 | 1.48E+01 | 1.88E+01 | 9.86E+00 | 1.41E+01 | 1.27E+01 | 1.65E+01 | 1.58E+01 | 16.13 |  | x | x |
| 162.1127 | 1.42 | 7.10E+01 | 6.63E+01 | 6.87E+01 | 7.03E+01 | 7.67E+01 | 8.22E+01 | 7.59E+01 | 8.61E+01 | 8.93E+01 | 7.60E+01 | 7.86E+01 | 8.78E+01 | 8.34E+01 | 8.97E+01 | 8.17E+01 | 8.80E+01 | 8.73E+01 | 9.65 | x | x | x |
| 197.1286 | 1.42 | 2.83E+00 | 1.32E+00 | 1.90E+00 | 1.96E+00 | 3.11E+00 | 2.12E+00 | 1.69E+00 | 3.52E+00 | 1.70E+00 | 2.78E+00 | 2.49E+00 | 4.40E+00 | 2.58E+00 | 2.99E+00 | 3.23E+00 | 2.36E+00 | 2.83E+00 | 29.87 |  |  | x |
| 245.9426 | 1.42 | 4.82E+00 | 4.71E+00 | 3.72E+00 | 5.21E+00 | 3.80E+00 | 5.57E+00 | 5.37E+00 | 4.97E+00 | 4.30E+00 | 2.28E+00 | 3.45E+00 | 4.72E+00 | 5.63E+00 | 3.94E+00 | 6.05E+00 | 5.13E+00 | 5.31E+00 | 20.65 |  |  | x |
| 273.9558 | 1.42 | 3.88E+00 | 3.40E+00 | 4.96E+00 | 5.42E+00 | 4.19E+00 | 5.18E+00 | 6.45E+00 | 6.36E+00 | 4.96E+00 | 4.42E+00 | 2.08E+00 | 6.19E+00 | 4.84E+00 | 7.13E+00 | 2.96E+00 | 6.51E+00 | 4.31E+00 | 28.06 |  |  | x |
| 415.1016 | 1.42 | 2.57E+01 | 2.68E+01 | 2.67E+01 | 2.44E+01 | 2.04E+01 | 2.17E+01 | 2.39E+01 | 2.13E+01 | 2.13E+01 | 1.93E+01 | 1.38E+01 | 2.17E+01 | 2.14E+01 | 2.59E+01 | 2.07E+01 | 2.51E+01 | 2.30E+01 | 14.47 | x | x | x |
| 167.0916 | 1.43 | 2.61E+02 | 2.41E+02 | 2.66E+02 | 2.89E+02 | 3.22E+02 | 3.28E+02 | 3.05E+02 | 3.25E+02 | 3.05E+02 | 2.71E+02 | 3.17E+02 | 3.07E+02 | 3.02E+02 | 3.00E+02 | 3.34E+02 | 3.00E+02 | 3.08E+02 | 8.6 | x | x | x |
| 412.6221 | 1.43 | 5.75E+01 | 4.19E+01 | 4.73E+01 | 3.90E+01 | 4.55E+01 | 4.25E+01 | 4.85E+01 | 4.02E+01 | 4.31E+01 | 3.41E+01 | 7.82E+00 | 4.30E+01 | 4.11E+01 | 4.47E+01 | 3.81E+01 | 4.63E+01 | 3.92E+01 | 24.34 |  |  | x |
| 484.5576 | 1.43 | 4.57E+02 | 3.76E+02 | 3.77E+02 | 3.52E+02 | 3.31E+02 | 3.81E+02 | 3.59E+02 | 3.51E+02 | 3.84E+02 | 3.50E+02 | 8.10E+01 | 3.44E+02 | 3.83E+02 | 3.83E+02 | 3.19E+02 | 3.78E+02 | 3.74E+02 | 21.61 |  |  | x |
| 260.7604 | 1.43 | 1.19E+02 | 9.93E+01 | 1.06E+02 | 9.88E+01 | 9.98E+01 | 1.17E+02 | 1.04E+02 | 1.04E+02 | 1.09E+02 | 9.92E+01 | 6.58E+01 | 9.75E+01 | 1.07E+02 | 1.08E+02 | 9.40E+01 | 1.07E+02 | 1.11E+02 | 11.32 | x | x | x |
| 262.7579 | 1.43 | 1.68E+02 | 1.36E+02 | 1.39E+02 | 1.32E+02 | 1.38E+02 | 1.54E+02 | 1.50E+02 | 1.36E+02 | 1.55E+02 | 1.42E+02 | 1.04E+02 | 1.42E+02 | 1.47E+02 | 1.48E+02 | 1.29E+02 | 1.52E+02 | 1.44E+02 | 9.68 | x | x | x |
| 334.6939 | 1.43 | 2.50E+02 | 2.08E+02 | 2.28E+02 | 2.13E+02 | 2.20E+02 | 2.45E+02 | 2.40E+02 | 2.25E+02 | 2.57E+02 | 2.41E+02 | 1.14E+02 | 2.39E+02 | 2.49E+02 | 2.67E+02 | 2.25E+02 | 2.62E+02 | 2.70E+02 | 15.32 |  | x | x |
| 336.6913 | 1.43 | 4.74E+02 | 3.91E+02 | 4.13E+02 | 4.06E+02 | 4.15E+02 | 4.68E+02 | 4.45E+02 | 4.30E+02 | 4.85E+02 | 4.50E+02 | 1.14E+02 | 4.54E+02 | 4.82E+02 | 5.04E+02 | 4.24E+02 | 4.83E+02 | 4.96E+02 | 20.5 |  |  | x |
| 338.6888 | 1.43 | 3.19E+02 | 2.70E+02 | 2.83E+02 | 2.76E+02 | 2.80E+02 | 3.09E+02 | 2.90E+02 | 2.92E+02 | 3.27E+02 | 3.03E+02 | 6.59E+01 | 2.99E+02 | 3.22E+02 | 3.31E+02 | 2.83E+02 | 3.24E+02 | 3.37E+02 | 21.17 |  |  | x |
| 340.6862 | 1.43 | 1.05E+02 | 8.28E+01 | 7.70E+01 | 8.25E+01 | 8.03E+01 | 9.71E+01 | 9.04E+01 | 8.30E+01 | 1.04E+02 | 9.61E+01 | 2.62E+01 | 9.43E+01 | 9.98E+01 | 9.91E+01 | 8.00E+01 | 1.07E+02 | 1.01E+02 | 21.22 |  |  | x |
| 784.2833 | 1.43 | 1.53E+02 | 1.20E+02 | 1.17E+02 | 9.68E+01 | 9.95E+01 | 9.82E+01 | 9.27E+01 | 8.63E+01 | 9.73E+01 | 9.36E+01 | 9.98E+00 | 8.74E+01 | 9.50E+01 | 1.04E+02 | 8.13E+01 | 9.95E+01 | 8.48E+01 | 29.16 |  |  | x |
| 408.6274 | 1.43 | 1.91E+01 | 1.50E+01 | 1.42E+01 | 1.16E+01 | 8.19E+00 | 9.40E+00 | 1.66E+01 | 9.54E+00 | 1.26E+01 | 1.43E+01 | 5.09E+00 | 1.70E+01 | 2.02E+01 | 1.33E+01 | 1.53E+01 | 1.54E+01 | 1.17E+01 | 29.26 |  |  | x |
| 410.6243 | 1.43 | 6.64E+01 | 5.41E+01 | 4.85E+01 | 4.51E+01 | 4.45E+01 | 4.91E+01 | 5.18E+01 | 4.95E+01 | 5.41E+01 | 4.68E+01 | 1.95E+01 | 4.42E+01 | 5.40E+01 | 4.86E+01 | 4.68E+01 | 5.09E+01 | 5.47E+01 | 18.93 |  | x | x |
| 486.5554 | 1.43 | 5.05E+02 | 4.24E+02 | 4.62E+02 | 3.90E+02 | 3.97E+02 | 4.31E+02 | 4.15E+02 | 3.93E+02 | 4.23E+02 | 3.76E+02 | 3.83E+01 | 3.84E+02 | 3.86E+02 | 4.08E+02 | 3.66E+02 | 4.03E+02 | 4.39E+02 | 24.83 |  |  | x |
| 482.5605 | 1.43 | 1.16E+02 | 1.01E+02 | 9.30E+01 | 8.85E+01 | 9.08E+01 | 8.46E+01 | 9.98E+01 | 8.88E+01 | 9.86E+01 | 8.30E+01 | 1.64E+01 | 8.42E+01 | 9.80E+01 | 9.47E+01 | 7.91E+01 | 9.52E+01 | 9.11E+01 | 23.13 |  |  | x |
| 488.5528 | 1.43 | 2.42E+02 | 2.01E+02 | 2.08E+02 | 1.84E+02 | 1.81E+02 | 2.00E+02 | 1.91E+02 | 1.75E+02 | 2.12E+02 | 1.87E+02 | 1.60E+01 | 1.86E+02 | 1.98E+02 | 2.11E+02 | 1.75E+02 | 1.94E+02 | 1.95E+02 | 25.12 |  |  | x |
| 490.5501 | 1.43 | 6.34E+01 | 5.09E+01 | 5.67E+01 | 5.13E+01 | 5.42E+01 | 4.28E+01 | 4.31E+01 | 3.79E+01 | 5.56E+01 | 4.28E+01 | 6.77E+00 | 4.62E+01 | 4.39E+01 | 6.01E+01 | 4.41E+01 | 4.59E+01 | 4.68E+01 | 26.62 |  |  | x |
| 494.5858 | 1.43 | 1.06E+02 | 8.62E+01 | 9.66E+01 | 7.82E+01 | 8.98E+01 | 9.60E+01 | 8.97E+01 | 8.32E+01 | 8.51E+01 | 7.49E+01 | 4.23E+00 | 8.24E+01 | 9.63E+01 | 9.24E+01 | 7.27E+01 | 8.46E+01 | 8.34E+01 | 26.54 |  |  | x |
| 558.4907 | 1.43 | 4.22E+02 | 3.35E+02 | 3.55E+02 | 3.00E+02 | 3.12E+02 | 3.22E+02 | 3.14E+02 | 2.73E+02 | 3.12E+02 | 2.93E+02 | 2.67E+01 | 3.07E+02 | 3.05E+02 | 3.15E+02 | 2.78E+02 | 3.15E+02 | 3.12E+02 | 25.94 |  |  | x |
| 560.4882 | 1.43 | 5.68E+02 | 4.43E+02 | 4.58E+02 | 4.07E+02 | 3.68E+02 | 4.25E+02 | 4.26E+02 | 3.95E+02 | 4.26E+02 | 3.76E+02 | 3.28E+01 | 4.10E+02 | 4.03E+02 | 4.16E+02 | 3.65E+02 | 4.25E+02 | 4.01E+02 | 26.27 |  |  | x |
| 562.4853 | 1.43 | 3.94E+02 | 3.12E+02 | 3.15E+02 | 2.65E+02 | 2.62E+02 | 2.77E+02 | 2.83E+02 | 2.69E+02 | 2.90E+02 | 2.64E+02 | 3.44E+01 | 2.74E+02 | 2.65E+02 | 2.76E+02 | 2.47E+02 | 2.87E+02 | 2.83E+02 | 25.55 |  |  | x |
| 556.4930 | 1.43 | 9.51E+01 | 6.37E+01 | 7.39E+01 | 6.43E+01 | 5.99E+01 | 6.10E+01 | 4.96E+01 | 5.99E+01 | 5.66E+01 | 5.43E+01 | 1.92E+01 | 5.75E+01 | 5.86E+01 | 6.47E+01 | 5.25E+01 | 6.31E+01 | 5.74E+01 | 24.41 |  |  | x |
| 564.4828 | 1.43 | 1.52E+02 | 1.16E+02 | 1.29E+02 | 9.71E+01 | 9.71E+01 | 8.63E+01 | 1.07E+02 | 8.74E+01 | 1.08E+02 | 9.81E+01 | 8.09E+00 | 9.57E+01 | 9.69E+01 | 1.04E+02 | 9.19E+01 | 1.14E+02 | 1.07E+02 | 28.69 |  |  | x |
| 634.4209 | 1.43 | 5.09E+02 | 4.14E+02 | 4.38E+02 | 3.83E+02 | 3.54E+02 | 3.58E+02 | 3.70E+02 | 3.47E+02 | 3.81E+02 | 3.36E+02 | 1.14E+01 | 3.59E+02 | 3.64E+02 | 3.60E+02 | 3.19E+02 | 3.68E+02 | 3.80E+02 | 27.77 |  |  | x |
| 636.4183 | 1.43 | 4.43E+02 | 3.49E+02 | 3.60E+02 | 3.19E+02 | 2.97E+02 | 2.97E+02 | 3.12E+02 | 2.65E+02 | 3.20E+02 | 2.81E+02 | 9.11E+00 | 2.90E+02 | 3.05E+02 | 2.92E+02 | 2.62E+02 | 2.96E+02 | 2.93E+02 | 28.84 |  |  | x |
| 638.4154 | 1.43 | 2.08E+02 | 1.60E+02 | 1.75E+02 | 1.49E+02 | 1.49E+02 | 1.31E+02 | 1.28E+02 | 1.38E+02 | 1.34E+02 | 1.23E+02 | 6.00E+00 | 1.33E+02 | 1.37E+02 | 1.42E+02 | 1.33E+02 | 1.34E+02 | 1.44E+02 | 28.82 |  |  | x |
| 706.3563 | 1.43 | 3.00E+02 | 2.35E+02 | 2.51E+02 | 2.32E+02 | 1.96E+02 | 2.15E+02 | 2.22E+02 | 1.96E+02 | 2.11E+02 | 1.83E+02 | 6.29E+00 | 1.97E+02 | 2.03E+02 | 2.25E+02 | 1.86E+02 | 2.00E+02 | 1.94E+02 | 28.69 |  |  | x |
| 708.3535 | 1.43 | 5.49E+02 | 4.26E+02 | 4.25E+02 | 4.13E+02 | 3.76E+02 | 4.03E+02 | 3.90E+02 | 3.52E+02 | 3.95E+02 | 3.61E+02 | 2.16E+01 | 3.61E+02 | 3.88E+02 | 4.18E+02 | 3.11E+02 | 3.84E+02 | 3.93E+02 | 27.58 |  |  | x |
| 712.3482 | 1.43 | 3.00E+02 | 2.32E+02 | 2.29E+02 | 2.12E+02 | 2.10E+02 | 2.12E+02 | 2.17E+02 | 2.07E+02 | 2.22E+02 | 1.71E+02 | 8.38E+00 | 2.01E+02 | 2.07E+02 | 2.21E+02 | 1.82E+02 | 2.05E+02 | 2.07E+02 | 27.96 |  |  | x |
| 720.3785 | 1.43 | 6.74E+01 | 5.73E+01 | 5.67E+01 | 5.64E+01 | 4.91E+01 | 5.85E+01 | 4.71E+01 | 4.84E+01 | 4.59E+01 | 4.43E+01 | 1.48E+00 | 5.01E+01 | 4.32E+01 | 4.73E+01 | 3.91E+01 | 5.46E+01 | 5.29E+01 | 28.76 |  |  | x |
| 786.2815 | 1.43 | 8.76E+01 | 6.19E+01 | 6.78E+01 | 6.25E+01 | 5.38E+01 | 5.33E+01 | 6.04E+01 | 5.19E+01 | 5.44E+01 | 5.03E+01 | 7.67E+00 | 5.04E+01 | 6.40E+01 | 5.31E+01 | 4.77E+01 | 5.70E+01 | 5.16E+01 | 28.05 |  |  | x |
| 280.1654 | 1.44 | 9.37E+01 | 8.84E+01 | 9.99E+01 | 1.06E+02 | 1.10E+02 | 1.12E+02 | 1.04E+02 | 1.15E+02 | 1.08E+02 | 9.73E+01 | 1.39E+02 | 1.19E+02 | 1.06E+02 | 1.10E+02 | 1.25E+02 | 1.04E+02 | 1.12E+02 | 10.85 | x | x | x |
| 265.1542 | 1.44 | 2.26E+01 | 2.37E+01 | 2.56E+01 | 2.20E+01 | 2.26E+01 | 2.27E+01 | 1.66E+01 | 3.14E+01 | 2.09E+01 | 2.11E+01 | 2.42E+01 | 1.65E+01 | 2.36E+01 | 2.67E+01 | 2.33E+01 | 2.21E+01 | 2.59E+01 | 15.19 |  | x | x |
| 290.1516 | 1.44 | 9.16E+01 | 7.75E+01 | 8.77E+01 | 8.67E+01 | 8.88E+01 | 8.82E+01 | 8.04E+01 | 9.05E+01 | 9.56E+01 | 8.00E+01 | 1.36E+02 | 8.15E+01 | 8.74E+01 | 8.78E+01 | 8.65E+01 | 8.42E+01 | 8.93E+01 | 14.32 | x | x | x |
| 123.0567 | 1.45 | 1.13E+01 | 1.07E+01 | 1.31E+01 | 1.22E+01 | 1.47E+01 | 1.73E+01 | 1.43E+01 | 1.49E+01 | 1.80E+01 | 1.58E+01 | 1.97E+01 | 1.26E+01 | 1.53E+01 | 1.88E+01 | 2.00E+01 | 1.58E+01 | 1.57E+01 | 18.32 |  | x | x |
| 150.0777 | 1.45 | 3.58E+02 | 3.26E+02 | 3.53E+02 | 3.62E+02 | 3.99E+02 | 3.95E+02 | 3.77E+02 | 4.13E+02 | 4.02E+02 | 3.40E+02 | 4.27E+02 | 4.41E+02 | 3.73E+02 | 3.54E+02 | 4.04E+02 | 3.47E+02 | 3.86E+02 | 8.46 | x | x | x |
| 141.0660 | 1.45 | 1.49E+02 | 1.42E+02 | 1.43E+02 | 1.51E+02 | 1.57E+02 | 1.56E+02 | 1.42E+02 | 1.58E+02 | 1.47E+02 | 1.32E+02 | 8.91E+01 | 1.20E+02 | 1.36E+02 | 1.19E+02 | 1.81E+02 | 1.26E+02 | 1.39E+02 | 14.41 | x | x | x |
| 160.1066 | 1.45 | 1.47E+01 | 1.34E+01 | 1.45E+01 | 1.59E+01 | 1.91E+01 | 1.46E+01 | 1.14E+01 | 1.49E+01 | 1.45E+01 | 1.67E+01 | 2.06E+01 | 1.31E+01 | 1.50E+01 | 1.30E+01 | 1.98E+01 | 1.34E+01 | 1.73E+01 | 16.62 |  | x | x |
| 186.0880 | 1.45 | 2.25E+01 | 2.19E+01 | 2.40E+01 | 2.34E+01 | 2.61E+01 | 2.95E+01 | 2.70E+01 | 2.83E+01 | 3.22E+01 | 2.68E+01 | 2.49E+01 | 2.39E+01 | 2.54E+01 | 2.70E+01 | 2.47E+01 | 2.58E+01 | 2.82E+01 | 10.13 | x | x | x |
| 211.1195 | 1.45 | 1.51E+02 | 1.45E+02 | 1.61E+02 | 1.56E+02 | 1.65E+02 | 1.61E+02 | 1.49E+02 | 1.66E+02 | 1.63E+02 | 1.40E+02 | 2.03E+02 | 1.43E+02 | 1.54E+02 | 1.47E+02 | 1.64E+02 | 1.47E+02 | 1.46E+02 | 9.34 | x | x | x |
| 231.1714 | 1.45 | 2.75E+03 | 2.57E+03 | 2.77E+03 | 2.81E+03 | 3.21E+03 | 3.18E+03 | 3.03E+03 | 3.38E+03 | 3.37E+03 | 2.93E+03 | 2.95E+03 | 3.35E+03 | 3.27E+03 | 3.29E+03 | 3.56E+03 | 3.22E+03 | 3.42E+03 | 9.07 | x | x | x |
| 231.3722 | 1.45 | 1.76E+01 | 1.77E+01 | 1.85E+01 | 1.80E+01 | 2.47E+01 | 1.79E+01 | 1.57E+01 | 2.38E+01 | 1.90E+01 | 1.73E+01 | 5.09E+00 | 2.30E+01 | 1.83E+01 | 1.63E+01 | 2.57E+01 | 1.33E+01 | 2.13E+01 | 26.12 |  |  | x |
| 245.1615 | 1.45 | 2.83E+03 | 2.61E+03 | 2.83E+03 | 2.87E+03 | 3.19E+03 | 3.25E+03 | 3.09E+03 | 3.40E+03 | 3.44E+03 | 2.97E+03 | 9.86E+02 | 3.32E+03 | 3.30E+03 | 3.30E+03 | 3.47E+03 | 3.25E+03 | 3.49E+03 | 19.39 |  | x | x |
| 245.2964 | 1.45 | 6.84E+01 | 6.41E+01 | 6.92E+01 | 6.83E+01 | 7.78E+01 | 7.41E+01 | 6.85E+01 | 8.40E+01 | 7.84E+01 | 6.82E+01 | 2.54E+01 | 7.73E+01 | 8.13E+01 | 8.22E+01 | 8.98E+01 | 8.00E+01 | 8.52E+01 | 19.58 |  | x | x |
| 245.3678 | 1.45 | 3.00E+01 | 3.47E+01 | 3.35E+01 | 3.63E+01 | 4.17E+01 | 3.95E+01 | 3.39E+01 | 4.62E+01 | 4.65E+01 | 3.57E+01 | 3.29E+01 | 3.15E+01 | 3.76E+01 | 3.56E+01 | 4.24E+01 | 3.69E+01 | 4.11E+01 | 12.94 | x | x | x |
| 283.1167 | 1.45 | 1.34E+02 | 1.26E+02 | 1.41E+02 | 1.65E+02 | 1.75E+02 | 2.04E+02 | 1.99E+02 | 1.80E+02 | 2.38E+02 | 2.14E+02 | 2.08E+02 | 1.49E+02 | 2.17E+02 | 2.49E+02 | 1.56E+02 | 2.46E+02 | 2.42E+02 | 21.47 |  |  | x |
| 295.1683 | 1.45 | 1.19E+01 | 9.92E+00 | 1.22E+01 | 1.66E+01 | 1.56E+01 | 1.51E+01 | 1.29E+01 | 1.20E+01 | 1.25E+01 | 9.14E+00 | 8.87E+00 | 2.13E+01 | 1.21E+01 | 7.97E+00 | 1.22E+01 | 8.65E+00 | 1.03E+01 | 27.5 |  |  | x |
| 397.0543 | 1.45 | 2.80E+01 | 2.64E+01 | 3.46E+01 | 3.41E+01 | 3.04E+01 | 3.78E+01 | 3.58E+01 | 3.73E+01 | 3.77E+01 | 2.64E+01 | 1.18E+01 | 2.35E+01 | 3.39E+01 | 4.01E+01 | 2.88E+01 | 3.02E+01 | 3.10E+01 | 22.06 |  |  | x |
| 435.0101 | 1.45 | 1.01E+02 | 7.08E+01 | 8.94E+01 | 7.52E+01 | 7.44E+01 | 7.14E+01 | 7.36E+01 | 6.42E+01 | 7.40E+01 | 7.08E+01 | 1.86E+01 | 3.96E+01 | 6.96E+01 | 6.82E+01 | 4.92E+01 | 6.26E+01 | 5.47E+01 | 27.98 |  |  | x |
| 227.1390 | 1.46 | 1.08E+01 | 9.64E+00 | 1.25E+01 | 1.24E+01 | 8.34E+00 | 9.48E+00 | 8.19E+00 | 1.05E+01 | 1.14E+01 | 9.09E+00 | 4.84E+00 | 1.09E+01 | 9.43E+00 | 1.08E+01 | 8.38E+00 | 8.55E+00 | 1.07E+01 | 18.98 |  | x | x |
| 241.1561 | 1.46 | 1.42E+02 | 1.40E+02 | 1.37E+02 | 1.11E+02 | 1.35E+02 | 1.39E+02 | 1.32E+02 | 1.43E+02 | 1.45E+02 | 1.38E+02 | 9.50E+01 | 1.58E+02 | 1.58E+02 | 1.69E+02 | 1.54E+02 | 1.60E+02 | 1.71E+02 | 13.5 | x | x | x |
| 296.0646 | 1.46 | 4.91E+01 | 4.64E+01 | 5.17E+01 | 6.06E+01 | 6.83E+01 | 7.03E+01 | 7.60E+01 | 6.76E+01 | 8.06E+01 | 7.32E+01 | 8.18E+01 | 7.27E+01 | 7.53E+01 | 8.46E+01 | 6.21E+01 | 8.54E+01 | 7.68E+01 | 17.29 |  | x | x |
| 146.0926 | 1.47 | 2.34E+02 | 2.19E+02 | 2.43E+02 | 2.61E+02 | 2.90E+02 | 2.97E+02 | 2.82E+02 | 3.03E+02 | 3.11E+02 | 2.76E+02 | 2.77E+02 | 3.16E+02 | 2.94E+02 | 2.97E+02 | 2.96E+02 | 2.83E+02 | 2.98E+02 | 9.65 | x | x | x |
| 166.0790 | 1.47 | 5.00E+00 | 4.72E+00 | 5.39E+00 | 4.89E+00 | 4.68E+00 | 7.75E+00 | 6.31E+00 | 7.00E+00 | 8.01E+00 | 8.11E+00 | 9.06E+00 | 6.60E+00 | 4.79E+00 | 6.70E+00 | 5.97E+00 | 7.17E+00 | 6.60E+00 | 21.16 |  |  | x |
| 231.3017 | 1.47 | 5.41E+01 | 5.14E+01 | 5.35E+01 | 5.38E+01 | 6.06E+01 | 5.69E+01 | 5.41E+01 | 6.39E+01 | 6.32E+01 | 5.01E+01 | 1.12E+01 | 6.89E+01 | 5.94E+01 | 6.10E+01 | 7.05E+01 | 6.17E+01 | 6.85E+01 | 23.41 |  |  | x |
| 229.2870 | 1.47 | 1.36E+01 | 1.28E+01 | 1.23E+01 | 1.11E+01 | 1.21E+01 | 1.19E+01 | 1.02E+01 | 1.34E+01 | 1.34E+01 | 1.25E+01 | 1.17E+01 | 1.54E+01 | 1.22E+01 | 1.55E+01 | 1.23E+01 | 1.28E+01 | 1.60E+01 | 12.1 | x | x | x |
| 264.0379 | 1.47 | 9.28E+00 | 8.45E+00 | 9.98E+00 | 1.12E+01 | 1.13E+01 | 1.66E+01 | 1.49E+01 | 1.15E+01 | 1.55E+01 | 1.62E+01 | 6.49E+00 | 1.54E+01 | 1.52E+01 | 1.83E+01 | 1.09E+01 | 1.53E+01 | 1.62E+01 | 26.07 |  |  | x |
| 269.1261 | 1.47 | 1.01E+02 | 9.07E+01 | 9.24E+01 | 1.09E+02 | 1.14E+02 | 1.30E+02 | 1.47E+02 | 1.18E+02 | 1.54E+02 | 1.53E+02 | 6.19E+01 | 7.69E+01 | 1.49E+02 | 1.78E+02 | 1.01E+02 | 1.80E+02 | 1.68E+02 | 28.67 |  |  | x |
| 150.1827 | 1.48 | 2.13E+00 | 3.41E+00 | 2.58E+00 | 2.36E+00 | 2.91E+00 | 3.38E+00 | 3.36E+00 | 4.10E+00 | 1.96E+00 | 1.95E+00 | 3.54E+00 | 3.23E+00 | 2.84E+00 | 2.62E+00 | 2.03E+00 | 2.50E+00 | 3.32E+00 | 22.55 |  |  | x |
| 154.0974 | 1.48 | 3.25E+01 | 2.76E+01 | 3.41E+01 | 4.30E+01 | 4.44E+01 | 4.34E+01 | 4.63E+01 | 3.74E+01 | 4.23E+01 | 3.40E+01 | 2.51E+01 | 5.12E+01 | 3.96E+01 | 3.07E+01 | 3.76E+01 | 3.56E+01 | 4.10E+01 | 18.2 |  | x | x |
| 198.1600 | 1.48 | 1.65E+02 | 1.45E+02 | 1.54E+02 | 1.63E+02 | 2.04E+02 | 1.76E+02 | 1.55E+02 | 2.11E+02 | 2.03E+02 | 1.73E+02 | 1.25E+02 | 2.11E+02 | 1.88E+02 | 1.92E+02 | 2.18E+02 | 1.88E+02 | 2.01E+02 | 14.61 | x | x | x |
| 336.0670 | 1.48 | 1.78E+01 | 1.76E+01 | 1.83E+01 | 2.03E+01 | 2.10E+01 | 1.78E+01 | 2.03E+01 | 2.07E+01 | 2.54E+01 | 1.77E+01 | 1.55E+01 | 2.07E+01 | 2.63E+01 | 2.21E+01 | 2.25E+01 | 2.16E+01 | 1.96E+01 | 13.82 | x | x | x |
| 360.9953 | 1.48 | 4.56E+01 | 3.95E+01 | 4.45E+01 | 3.85E+01 | 3.72E+01 | 4.03E+01 | 3.48E+01 | 3.34E+01 | 4.23E+01 | 3.67E+01 | 3.04E+00 | 2.92E+01 | 3.98E+01 | 4.19E+01 | 3.72E+01 | 3.85E+01 | 3.66E+01 | 26.03 |  |  | x |
| 209.0560 | 1.49 | 2.84E+01 | 2.54E+01 | 2.79E+01 | 3.29E+01 | 3.72E+01 | 3.63E+01 | 3.12E+01 | 3.89E+01 | 4.15E+01 | 3.36E+01 | 2.20E+01 | 3.33E+01 | 3.52E+01 | 3.47E+01 | 3.35E+01 | 3.65E+01 | 3.68E+01 | 14.98 | x | x | x |
| 238.0941 | 1.49 | 8.68E+01 | 7.68E+01 | 8.23E+01 | 9.30E+01 | 9.64E+01 | 1.05E+02 | 1.01E+02 | 9.97E+01 | 1.03E+02 | 9.29E+01 | 5.48E+01 | 8.55E+01 | 9.86E+01 | 9.59E+01 | 8.97E+01 | 9.75E+01 | 1.02E+02 | 13.4 | x | x | x |
| 314.0059 | 1.49 | 3.16E+01 | 2.09E+01 | 3.04E+01 | 2.64E+01 | 2.75E+01 | 2.89E+01 | 3.04E+01 | 2.47E+01 | 3.06E+01 | 2.84E+01 | 6.51E+00 | 2.46E+01 | 3.29E+01 | 3.12E+01 | 2.64E+01 | 3.04E+01 | 3.07E+01 | 22.68 |  |  | x |
| 322.9879 | 1.49 | 2.13E+02 | 1.87E+02 | 2.18E+02 | 1.99E+02 | 1.99E+02 | 2.17E+02 | 2.19E+02 | 2.00E+02 | 2.18E+02 | 2.10E+02 | 1.81E+02 | 1.68E+02 | 2.11E+02 | 2.23E+02 | 1.84E+02 | 2.18E+02 | 2.27E+02 | 8.25 | x | x | x |
| 158.1183 | 1.50 | 1.23E+03 | 1.14E+03 | 1.26E+03 | 1.38E+03 | 1.46E+03 | 1.58E+03 | 1.49E+03 | 1.48E+03 | 1.48E+03 | 1.32E+03 | 1.35E+03 | 1.60E+03 | 1.44E+03 | 1.40E+03 | 1.48E+03 | 1.39E+03 | 1.47E+03 | 8.49 | x | x | x |
| 158.2258 | 1.50 | 3.00E+01 | 2.76E+01 | 3.03E+01 | 3.26E+01 | 3.59E+01 | 3.61E+01 | 3.43E+01 | 3.38E+01 | 3.46E+01 | 3.02E+01 | 3.43E+01 | 4.04E+01 | 3.43E+01 | 3.27E+01 | 3.37E+01 | 3.28E+01 | 3.46E+01 | 8.67 | x | x | x |
| 158.2850 | 1.50 | 1.06E+01 | 1.21E+01 | 1.27E+01 | 1.19E+01 | 1.84E+01 | 1.44E+01 | 1.55E+01 | 1.58E+01 | 1.54E+01 | 1.22E+01 | 1.34E+01 | 1.73E+01 | 1.22E+01 | 9.10E+00 | 1.10E+01 | 1.20E+01 | 1.17E+01 | 18.71 |  | x | x |
| 179.0046 | 1.50 | 1.70E+01 | 1.78E+01 | 1.75E+01 | 1.79E+01 | 2.15E+01 | 2.10E+01 | 2.02E+01 | 2.06E+01 | 2.06E+01 | 1.54E+01 | 1.17E+01 | 2.00E+01 | 2.07E+01 | 1.77E+01 | 1.97E+01 | 1.51E+01 | 2.10E+01 | 14.36 | x | x | x |
| 207.1127 | 1.50 | 8.89E+01 | 8.47E+01 | 8.52E+01 | 8.28E+01 | 8.74E+01 | 9.54E+01 | 8.93E+01 | 8.30E+01 | 9.28E+01 | 7.96E+01 | 6.79E+01 | 9.52E+01 | 7.83E+01 | 8.41E+01 | 7.72E+01 | 7.68E+01 | 7.66E+01 | 8.76 | x | x | x |
| 216.0198 | 1.50 | 1.09E+02 | 8.81E+01 | 8.53E+01 | 9.61E+01 | 9.12E+01 | 1.09E+02 | 1.11E+02 | 9.47E+01 | 1.18E+02 | 1.11E+02 | 1.27E+02 | 2.39E+01 | 1.11E+02 | 1.39E+02 | 8.31E+01 | 1.24E+02 | 1.12E+02 | 24.89 |  |  | x |
| 244.0793 | 1.50 | 4.70E+01 | 4.52E+01 | 4.28E+01 | 5.30E+01 | 5.09E+01 | 5.19E+01 | 5.20E+01 | 4.04E+01 | 5.11E+01 | 4.91E+01 | 5.17E+01 | 4.13E+01 | 4.49E+01 | 4.37E+01 | 4.00E+01 | 5.00E+01 | 5.60E+01 | 10.33 | x | x | x |
| 289.0412 | 1.50 | 4.73E+01 | 4.55E+01 | 4.08E+01 | 4.01E+01 | 3.52E+01 | 4.77E+01 | 4.44E+01 | 2.82E+01 | 3.65E+01 | 3.47E+01 | 2.59E+01 | 3.15E+01 | 2.86E+01 | 2.99E+01 | 2.30E+01 | 3.16E+01 | 3.48E+01 | 21.36 |  |  | x |
| 267.1268 | 1.50 | 3.17E+02 | 2.97E+02 | 3.34E+02 | 3.49E+02 | 3.66E+02 | 3.89E+02 | 3.60E+02 | 3.39E+02 | 3.81E+02 | 3.24E+02 | 1.33E+02 | 4.15E+02 | 3.53E+02 | 3.91E+02 | 3.42E+02 | 3.77E+02 | 3.89E+02 | 18.17 |  | x | x |
| 285.1553 | 1.50 | 7.09E+01 | 6.50E+01 | 8.07E+01 | 8.37E+01 | 8.51E+01 | 9.42E+01 | 8.78E+01 | 8.37E+01 | 9.36E+01 | 8.42E+01 | 2.38E+01 | 9.23E+01 | 8.20E+01 | 7.05E+01 | 8.16E+01 | 8.10E+01 | 8.70E+01 | 20.63 |  |  | x |
| 297.1447 | 1.50 | 9.68E+02 | 8.81E+02 | 9.90E+02 | 1.07E+03 | 1.11E+03 | 1.16E+03 | 1.05E+03 | 1.12E+03 | 1.09E+03 | 9.41E+02 | 6.85E+01 | 1.43E+03 | 1.05E+03 | 9.82E+02 | 1.12E+03 | 1.01E+03 | 1.09E+03 | 26.8 |  |  | x |
| 320.9894 | 1.50 | 1.45E+03 | 1.25E+03 | 1.41E+03 | 1.39E+03 | 1.45E+03 | 1.60E+03 | 1.53E+03 | 1.47E+03 | 1.58E+03 | 1.47E+03 | 4.09E+02 | 1.32E+03 | 1.54E+03 | 1.61E+03 | 1.42E+03 | 1.56E+03 | 1.58E+03 | 19.65 |  | x | x |
| 126.0666 | 1.51 | 8.74E+01 | 8.00E+01 | 8.71E+01 | 9.21E+01 | 1.12E+02 | 1.13E+02 | 1.18E+02 | 1.22E+02 | 1.29E+02 | 1.10E+02 | 1.13E+02 | 1.50E+02 | 1.26E+02 | 1.30E+02 | 1.30E+02 | 1.26E+02 | 1.34E+02 | 16.65 |  | x | x |
| 162.0553 | 1.51 | 4.45E+01 | 4.15E+01 | 4.37E+01 | 3.77E+01 | 4.59E+01 | 5.19E+01 | 4.65E+01 | 4.30E+01 | 4.37E+01 | 3.72E+01 | 3.57E+01 | 5.62E+01 | 4.01E+01 | 4.62E+01 | 4.59E+01 | 4.34E+01 | 4.06E+01 | 11.69 | x | x | x |
| 196.0743 | 1.51 | 7.91E+02 | 7.45E+02 | 8.47E+02 | 8.92E+02 | 9.81E+02 | 1.08E+03 | 1.06E+03 | 1.02E+03 | 1.12E+03 | 9.76E+02 | 6.98E+02 | 9.73E+02 | 1.07E+03 | 1.10E+03 | 1.01E+03 | 1.06E+03 | 1.11E+03 | 13.57 | x | x | x |
| 196.1940 | 1.51 | 1.43E+01 | 1.47E+01 | 1.52E+01 | 1.71E+01 | 1.85E+01 | 2.09E+01 | 1.97E+01 | 2.01E+01 | 2.18E+01 | 1.91E+01 | 2.01E+01 | 1.99E+01 | 2.24E+01 | 2.33E+01 | 2.11E+01 | 2.30E+01 | 2.38E+01 | 14.93 | x | x | x |
| 196.2612 | 1.51 | 2.67E+00 | 5.53E+00 | 4.96E+00 | 4.65E+00 | 6.83E+00 | 6.76E+00 | 8.45E+00 | 7.02E+00 | 8.28E+00 | 7.13E+00 | 6.52E+00 | 5.41E+00 | 8.58E+00 | 8.40E+00 | 7.43E+00 | 8.72E+00 | 8.48E+00 | 25 |  |  | x |
| 217.1549 | 1.51 | 4.82E+01 | 4.43E+01 | 5.25E+01 | 5.31E+01 | 5.95E+01 | 6.47E+01 | 5.73E+01 | 5.70E+01 | 5.49E+01 | 5.81E+01 | 2.79E+01 | 8.37E+01 | 5.73E+01 | 6.28E+01 | 5.54E+01 | 6.05E+01 | 6.48E+01 | 19.8 |  | x | x |
| 230.1132 | 1.51 | 1.21E+01 | 1.08E+01 | 1.37E+01 | 1.71E+01 | 1.87E+01 | 1.50E+01 | 1.53E+01 | 1.68E+01 | 1.79E+01 | 1.58E+01 | 7.66E+00 | 1.97E+01 | 1.62E+01 | 1.62E+01 | 1.56E+01 | 1.56E+01 | 1.72E+01 | 19.2 |  | x | x |
| 280.2142 | 1.51 | 8.60E+00 | 9.35E+00 | 8.26E+00 | 1.02E+01 | 1.37E+01 | 1.45E+01 | 1.46E+01 | 1.39E+01 | 1.61E+01 | 1.40E+01 | 6.03E+00 | 1.67E+01 | 1.66E+01 | 1.62E+01 | 1.54E+01 | 1.69E+01 | 1.84E+01 | 26.93 |  |  | x |
| 280.0711 | 1.51 | 6.13E+02 | 5.69E+02 | 6.05E+02 | 6.83E+02 | 8.61E+02 | 8.78E+02 | 8.54E+02 | 8.93E+02 | 9.46E+02 | 8.34E+02 | 3.14E+02 | 9.51E+02 | 9.24E+02 | 9.45E+02 | 9.26E+02 | 9.68E+02 | 1.03E+03 | 23.3 |  |  | x |
| 376.0435 | 1.51 | 3.65E+02 | 3.49E+02 | 4.09E+02 | 4.30E+02 | 4.55E+02 | 4.74E+02 | 4.51E+02 | 4.52E+02 | 4.45E+02 | 3.88E+02 | 1.35E+01 | 5.17E+02 | 4.43E+02 | 4.48E+02 | 4.49E+02 | 4.56E+02 | 4.37E+02 | 26.74 |  |  | x |
| 216.1701 | 1.52 | 7.28E+00 | 6.30E+00 | 9.20E+00 | 1.08E+01 | 1.06E+01 | 1.11E+01 | 1.01E+01 | 1.06E+01 | 1.07E+01 | 8.96E+00 | 6.30E+00 | 9.58E+00 | 1.05E+01 | 1.08E+01 | 1.03E+01 | 1.06E+01 | 1.15E+01 | 16.66 |  | x | x |
| 343.0464 | 1.52 | 2.78E+01 | 2.52E+01 | 2.36E+01 | 3.03E+01 | 2.93E+01 | 3.14E+01 | 2.34E+01 | 2.53E+01 | 2.40E+01 | 2.69E+01 | 1.40E+00 | 2.45E+01 | 3.39E+01 | 3.22E+01 | 3.10E+01 | 2.64E+01 | 3.81E+01 | 28.81 |  |  | x |
| 413.9998 | 1.52 | 5.61E+02 | 4.75E+02 | 5.30E+02 | 5.03E+02 | 4.96E+02 | 5.27E+02 | 4.85E+02 | 4.77E+02 | 5.21E+02 | 4.88E+02 | 1.90E+01 | 5.56E+02 | 4.95E+02 | 5.22E+02 | 4.79E+02 | 5.15E+02 | 5.36E+02 | 25.36 |  |  | x |
| 132.1016 | 1.53 | 1.64E+01 | 1.76E+01 | 1.72E+01 | 1.65E+01 | 1.80E+01 | 1.96E+01 | 2.27E+01 | 2.28E+01 | 2.53E+01 | 2.09E+01 | 2.94E+01 | 2.46E+01 | 2.18E+01 | 1.94E+01 | 2.24E+01 | 2.51E+01 | 2.53E+01 | 17.47 |  | x | x |
| 144.1627 | 1.53 | 2.14E+00 | 1.48E+00 | 2.43E+00 | 2.48E+00 | 2.17E+00 | 2.78E+00 | 2.88E+00 | 3.26E+00 | 2.73E+00 | 1.57E+00 | 2.34E+00 | 2.80E+00 | 2.61E+00 | 2.24E+00 | 2.63E+00 | 2.28E+00 | 2.72E+00 | 18.39 |  | x | x |
| 144.2043 | 1.53 | 9.04E+00 | 8.61E+00 | 9.56E+00 | 1.00E+01 | 9.62E+00 | 1.14E+01 | 1.06E+01 | 1.06E+01 | 1.05E+01 | 9.29E+00 | 1.07E+01 | 1.22E+01 | 1.05E+01 | 1.03E+01 | 9.68E+00 | 1.04E+01 | 1.06E+01 | 8.51 | x | x | x |
| 182.0587 | 1.53 | 6.10E+02 | 5.88E+02 | 6.80E+02 | 6.96E+02 | 7.50E+02 | 8.32E+02 | 8.43E+02 | 7.95E+02 | 8.06E+02 | 7.09E+02 | 3.42E+02 | 9.37E+02 | 7.86E+02 | 8.00E+02 | 7.90E+02 | 7.80E+02 | 8.11E+02 | 18.07 |  | x | x |
| 227.1379 | 1.53 | 9.28E+00 | 9.83E+00 | 1.03E+01 | 7.86E+00 | 1.14E+01 | 1.16E+01 | 1.02E+01 | 9.16E+00 | 1.35E+01 | 1.09E+01 | 4.15E+00 | 1.55E+01 | 1.25E+01 | 1.35E+01 | 1.39E+01 | 1.23E+01 | 1.03E+01 | 24.16 |  |  | x |
| 241.9984 | 1.53 | 3.43E+01 | 3.15E+01 | 3.02E+01 | 3.28E+01 | 3.94E+01 | 4.01E+01 | 3.59E+01 | 4.37E+01 | 3.88E+01 | 3.55E+01 | 1.89E+01 | 4.12E+01 | 3.62E+01 | 4.13E+01 | 4.76E+01 | 3.69E+01 | 4.27E+01 | 17.63 |  | x | x |
| 354.8954 | 1.53 | 1.26E+01 | 1.24E+01 | 1.46E+01 | 9.58E+00 | 5.51E+00 | 8.30E+00 | 9.14E+00 | 1.28E+01 | 1.19E+01 | 8.21E+00 | 6.99E+00 | 1.24E+01 | 1.59E+01 | 1.10E+01 | 9.06E+00 | 1.02E+01 | 9.20E+00 | 25.8 |  |  | x |
| 112.0507 | 1.54 | 5.87E+01 | 5.21E+01 | 5.87E+01 | 6.48E+01 | 6.17E+01 | 7.54E+01 | 7.80E+01 | 6.63E+01 | 7.57E+01 | 6.60E+01 | 6.67E+01 | 7.16E+01 | 6.96E+01 | 7.09E+01 | 6.48E+01 | 6.92E+01 | 7.08E+01 | 10.05 | x | x | x |
| 144.1016 | 1.54 | 6.21E+02 | 5.70E+02 | 6.61E+02 | 6.85E+02 | 6.87E+02 | 7.76E+02 | 7.68E+02 | 7.08E+02 | 7.36E+02 | 6.40E+02 | 6.21E+02 | 8.04E+02 | 7.13E+02 | 7.00E+02 | 6.94E+02 | 6.88E+02 | 7.22E+02 | 8.64 | x | x | x |
| 156.0426 | 1.54 | 1.72E+02 | 1.58E+02 | 1.91E+02 | 1.99E+02 | 2.13E+02 | 2.52E+02 | 2.51E+02 | 2.31E+02 | 2.45E+02 | 2.13E+02 | 1.56E+02 | 2.77E+02 | 2.46E+02 | 2.27E+02 | 2.32E+02 | 2.31E+02 | 2.46E+02 | 15.8 |  | x | x |
| 188.0706 | 1.54 | 2.60E+01 | 2.55E+01 | 3.21E+01 | 2.93E+01 | 3.39E+01 | 3.88E+01 | 3.54E+01 | 3.48E+01 | 4.06E+01 | 3.43E+01 | 3.15E+01 | 3.53E+01 | 4.27E+01 | 3.85E+01 | 3.75E+01 | 4.15E+01 | 4.19E+01 | 14.84 | x | x | x |
| 265.1543 | 1.54 | 8.13E+00 | 5.90E+00 | 6.54E+00 | 4.52E+00 | 8.13E+00 | 6.98E+00 | 5.76E+00 | 7.71E+00 | 6.31E+00 | 7.50E+00 | 6.37E+00 | 6.63E+00 | 6.21E+00 | 6.04E+00 | 8.37E+00 | 6.70E+00 | 5.60E+00 | 15.49 |  | x | x |
| 266.0538 | 1.54 | 3.27E+02 | 3.01E+02 | 3.33E+02 | 3.53E+02 | 3.87E+02 | 4.19E+02 | 4.05E+02 | 4.07E+02 | 4.27E+02 | 3.63E+02 | 7.77E+01 | 4.49E+02 | 4.06E+02 | 4.10E+02 | 3.92E+02 | 4.09E+02 | 4.32E+02 | 23.11 |  |  | x |
| 271.1402 | 1.54 | 3.02E+02 | 2.83E+02 | 3.21E+02 | 3.21E+02 | 3.26E+02 | 3.65E+02 | 3.48E+02 | 3.41E+02 | 3.31E+02 | 3.22E+02 | 1.17E+02 | 3.24E+02 | 3.53E+02 | 3.53E+02 | 3.08E+02 | 3.44E+02 | 3.53E+02 | 17.62 |  | x | x |
| 347.0528 | 1.54 | 1.53E+02 | 1.51E+02 | 1.49E+02 | 1.48E+02 | 1.83E+02 | 1.66E+02 | 1.63E+02 | 1.74E+02 | 1.83E+02 | 1.50E+02 | 2.42E+01 | 2.01E+02 | 1.77E+02 | 1.72E+02 | 1.88E+02 | 1.72E+02 | 1.80E+02 | 23.88 |  |  | x |
| 118.0863 | 1.55 | 4.66E+02 | 4.33E+02 | 4.93E+02 | 5.13E+02 | 5.49E+02 | 6.11E+02 | 5.98E+02 | 5.52E+02 | 6.21E+02 | 5.49E+02 | 4.64E+02 | 5.95E+02 | 5.85E+02 | 5.80E+02 | 5.27E+02 | 5.72E+02 | 5.98E+02 | 10.34 | x | x | x |
| 166.0837 | 1.55 | 9.99E+01 | 8.28E+01 | 8.33E+01 | 9.93E+01 | 9.17E+01 | 9.75E+01 | 1.05E+02 | 6.94E+01 | 9.18E+01 | 7.81E+01 | 5.16E+01 | 7.14E+01 | 7.48E+01 | 6.10E+01 | 6.54E+01 | 6.54E+01 | 6.93E+01 | 19.64 |  | x | x |
| 212.1042 | 1.55 | 6.82E+01 | 6.04E+01 | 7.12E+01 | 8.42E+01 | 7.42E+01 | 8.27E+01 | 8.05E+01 | 6.95E+01 | 6.80E+01 | 6.12E+01 | 1.17E+01 | 5.53E+01 | 6.34E+01 | 5.58E+01 | 5.30E+01 | 5.80E+01 | 5.78E+01 | 26.04 |  |  | x |
| 235.1189 | 1.55 | 1.47E+01 | 1.35E+01 | 1.40E+01 | 1.65E+01 | 1.70E+01 | 1.46E+01 | 1.35E+01 | 1.36E+01 | 1.39E+01 | 9.93E+00 | 5.26E+00 | 1.33E+01 | 1.17E+01 | 1.35E+01 | 1.49E+01 | 1.43E+01 | 1.56E+01 | 19.83 |  | x | x |
| 271.2815 | 1.55 | 3.31E+00 | 2.77E+00 | 3.78E+00 | 3.37E+00 | 3.25E+00 | 3.20E+00 | 3.27E+00 | 3.03E+00 | 3.48E+00 | 3.65E+00 | 1.75E+00 | 2.98E+00 | 4.10E+00 | 3.12E+00 | 3.09E+00 | 3.74E+00 | 4.21E+00 | 16.92 |  | x | x |
| 281.0092 | 1.55 | 2.18E+01 | 1.87E+01 | 2.14E+01 | 1.92E+01 | 2.78E+01 | 2.35E+01 | 2.14E+01 | 2.69E+01 | 2.42E+01 | 2.22E+01 | 1.44E+01 | 3.23E+01 | 2.39E+01 | 2.59E+01 | 3.34E+01 | 2.57E+01 | 2.94E+01 | 20.06 |  |  | x |
| 283.1628 | 1.55 | 9.67E+01 | 8.90E+01 | 1.01E+02 | 1.11E+02 | 1.20E+02 | 1.24E+02 | 1.32E+02 | 1.22E+02 | 1.43E+02 | 1.31E+02 | 4.77E+01 | 9.77E+01 | 1.24E+02 | 1.40E+02 | 9.16E+01 | 1.26E+02 | 1.26E+02 | 20.94 |  |  | x |
| 293.1216 | 1.55 | 9.73E+01 | 8.23E+01 | 1.02E+02 | 8.67E+01 | 8.24E+01 | 9.46E+01 | 9.05E+01 | 7.72E+01 | 8.69E+01 | 7.02E+01 | 1.54E+01 | 5.76E+01 | 8.91E+01 | 7.37E+01 | 6.12E+01 | 8.36E+01 | 8.30E+01 | 25.59 |  |  | x |
| 312.9101 | 1.55 | 4.03E+01 | 3.48E+01 | 3.65E+01 | 2.72E+01 | 2.50E+01 | 2.80E+01 | 2.47E+01 | 2.93E+01 | 2.79E+01 | 2.71E+01 | 4.34E+00 | 3.21E+01 | 3.75E+01 | 2.51E+01 | 2.32E+01 | 3.09E+01 | 3.56E+01 | 28.09 |  |  | x |
| 257.1478 | 1.56 | 4.13E+02 | 3.96E+02 | 4.62E+02 | 5.21E+02 | 5.57E+02 | 5.99E+02 | 6.20E+02 | 5.47E+02 | 6.53E+02 | 5.61E+02 | 5.43E+01 | 5.19E+02 | 5.89E+02 | 6.06E+02 | 4.53E+02 | 5.51E+02 | 5.68E+02 | 26.99 |  |  | x |
| 140.0680 | 1.57 | 2.46E+02 | 2.10E+02 | 2.35E+02 | 2.52E+02 | 2.60E+02 | 2.85E+02 | 2.80E+02 | 2.53E+02 | 2.97E+02 | 2.60E+02 | 1.66E+02 | 2.46E+02 | 2.65E+02 | 2.81E+02 | 2.21E+02 | 2.57E+02 | 2.66E+02 | 12.41 | x | x | x |
| 231.1473 | 1.57 | 7.03E+01 | 7.07E+01 | 7.63E+01 | 7.37E+01 | 8.64E+01 | 9.24E+01 | 8.79E+01 | 9.71E+01 | 8.34E+01 | 7.31E+01 | 4.57E+01 | 8.44E+01 | 8.66E+01 | 8.81E+01 | 8.41E+01 | 8.49E+01 | 9.23E+01 | 14.86 | x | x | x |
| 240.1067 | 1.57 | 6.44E+01 | 5.95E+01 | 6.63E+01 | 6.89E+01 | 7.87E+01 | 7.67E+01 | 6.99E+01 | 7.81E+01 | 8.63E+01 | 7.10E+01 | 5.51E+01 | 6.86E+01 | 7.03E+01 | 8.16E+01 | 6.63E+01 | 6.95E+01 | 7.32E+01 | 11.05 | x | x | x |
| 247.1644 | 1.57 | 1.75E+01 | 1.59E+01 | 1.73E+01 | 1.71E+01 | 1.85E+01 | 1.98E+01 | 1.82E+01 | 1.78E+01 | 1.92E+01 | 1.89E+01 | 5.12E+00 | 2.13E+01 | 1.86E+01 | 1.80E+01 | 2.02E+01 | 1.73E+01 | 2.11E+01 | 20.05 |  |  | x |
| 254.1608 | 1.57 | 8.83E+01 | 7.79E+01 | 8.62E+01 | 8.11E+01 | 9.17E+01 | 9.44E+01 | 8.49E+01 | 9.04E+01 | 9.19E+01 | 8.36E+01 | 2.35E+01 | 8.96E+01 | 8.64E+01 | 9.12E+01 | 8.31E+01 | 8.42E+01 | 9.29E+01 | 19.31 |  | x | x |
| 296.9362 | 1.57 | 6.22E+01 | 5.45E+01 | 5.91E+01 | 5.15E+01 | 4.81E+01 | 4.17E+01 | 4.48E+01 | 3.79E+01 | 4.99E+01 | 4.46E+01 | 1.95E+01 | 4.90E+01 | 4.93E+01 | 4.43E+01 | 3.72E+01 | 4.57E+01 | 5.34E+01 | 20.73 |  |  | x |
| 371.2403 | 1.57 | 3.13E+01 | 3.29E+01 | 3.34E+01 | 4.27E+01 | 4.68E+01 | 4.68E+01 | 4.87E+01 | 4.46E+01 | 5.15E+01 | 5.26E+01 | 4.02E+00 | 5.04E+01 | 4.56E+01 | 4.89E+01 | 4.39E+01 | 4.72E+01 | 4.93E+01 | 27.83 |  |  | x |
| 399.1268 | 1.57 | 2.62E+01 | 2.71E+01 | 2.51E+01 | 2.55E+01 | 2.65E+01 | 1.98E+01 | 2.45E+01 | 2.03E+01 | 2.39E+01 | 2.16E+01 | 1.29E+00 | 2.27E+01 | 2.15E+01 | 2.62E+01 | 1.81E+01 | 1.54E+01 | 1.69E+01 | 29.34 |  |  | x |
| 205.0685 | 1.58 | 1.64E+02 | 1.40E+02 | 1.65E+02 | 1.84E+02 | 2.15E+02 | 2.12E+02 | 2.09E+02 | 2.21E+02 | 2.32E+02 | 2.06E+02 | 1.51E+02 | 2.16E+02 | 2.20E+02 | 2.19E+02 | 2.18E+02 | 2.12E+02 | 2.35E+02 | 14.43 | x | x | x |
| 214.1547 | 1.58 | 2.55E+01 | 2.50E+01 | 2.44E+01 | 2.74E+01 | 2.64E+01 | 2.83E+01 | 3.00E+01 | 2.96E+01 | 3.51E+01 | 2.59E+01 | 1.48E+01 | 2.83E+01 | 3.12E+01 | 2.96E+01 | 2.39E+01 | 2.80E+01 | 3.14E+01 | 15.92 |  | x | x |
| 217.1052 | 1.58 | 7.35E+01 | 6.45E+01 | 7.56E+01 | 8.39E+01 | 8.67E+01 | 9.19E+01 | 8.45E+01 | 8.65E+01 | 8.69E+01 | 6.98E+01 | 3.39E+01 | 7.71E+01 | 7.90E+01 | 8.31E+01 | 7.11E+01 | 7.33E+01 | 8.23E+01 | 17.28 |  | x | x |
| 285.1023 | 1.58 | 6.53E+01 | 5.65E+01 | 6.49E+01 | 6.66E+01 | 6.66E+01 | 6.80E+01 | 6.36E+01 | 6.44E+01 | 7.15E+01 | 6.61E+01 | 5.54E+01 | 6.75E+01 | 5.94E+01 | 6.18E+01 | 5.54E+01 | 5.99E+01 | 6.70E+01 | 7.44 | x | x | x |
| 188.1751 | 1.59 | 3.74E+00 | 3.62E+00 | 3.35E+00 | 2.78E+00 | 5.49E+00 | 3.63E+00 | 4.70E+00 | 6.30E+00 | 6.48E+00 | 4.08E+00 | 5.40E+00 | 4.01E+00 | 2.61E+00 | 3.21E+00 | 4.21E+00 | 5.14E+00 | 7.09E+00 | 29.79 |  |  | x |
| 229.1222 | 1.59 | 8.30E+01 | 6.78E+01 | 9.31E+01 | 8.17E+01 | 1.07E+02 | 9.72E+01 | 1.03E+02 | 9.35E+01 | 1.03E+02 | 7.32E+01 | 6.28E+01 | 1.05E+02 | 9.48E+01 | 1.07E+02 | 8.91E+01 | 1.04E+02 | 1.02E+02 | 15.17 |  | x | x |
| 273.0828 | 1.59 | 8.25E+00 | 6.80E+00 | 5.07E+00 | 7.71E+00 | 5.93E+00 | 8.30E+00 | 8.50E+00 | 7.36E+00 | 6.68E+00 | 5.13E+00 | 6.70E+00 | 5.93E+00 | 7.98E+00 | 6.56E+00 | 6.66E+00 | 6.14E+00 | 4.53E+00 | 17.7 |  | x | x |
| 301.1159 | 1.59 | 1.08E+01 | 8.72E+00 | 9.22E+00 | 8.33E+00 | 1.38E+01 | 8.50E+00 | 1.15E+01 | 9.93E+00 | 9.49E+00 | 8.67E+00 | 2.92E+00 | 9.72E+00 | 8.39E+00 | 7.26E+00 | 9.25E+00 | 6.99E+00 | 1.13E+01 | 25.21 |  |  | x |
| 94.0656 | 1.60 | 1.11E+02 | 1.09E+02 | 1.22E+02 | 1.32E+02 | 1.39E+02 | 1.46E+02 | 1.39E+02 | 1.44E+02 | 1.47E+02 | 1.31E+02 | 1.77E+02 | 1.42E+02 | 1.41E+02 | 1.42E+02 | 1.40E+02 | 1.37E+02 | 1.45E+02 | 11.09 | x | x | x |
| 136.0395 | 1.60 | 9.94E+00 | 8.75E+00 | 8.42E+00 | 9.50E+00 | 1.00E+01 | 8.30E+00 | 8.30E+00 | 8.82E+00 | 7.53E+00 | 8.90E+00 | 7.89E+00 | 9.04E+00 | 8.46E+00 | 7.62E+00 | 1.05E+01 | 1.09E+01 | 1.14E+01 | 12.5 | x | x | x |
| 138.0563 | 1.60 | 4.41E+03 | 4.03E+03 | 4.39E+03 | 4.79E+03 | 4.89E+03 | 5.38E+03 | 5.30E+03 | 5.18E+03 | 5.44E+03 | 4.83E+03 | 4.55E+03 | 5.38E+03 | 5.24E+03 | 5.15E+03 | 5.13E+03 | 5.20E+03 | 5.31E+03 | 8.39 | x | x | x |
| 138.1149 | 1.60 | 4.55E+01 | 4.21E+01 | 4.72E+01 | 4.95E+01 | 5.04E+01 | 5.11E+01 | 4.80E+01 | 5.37E+01 | 5.07E+01 | 4.67E+01 | 3.01E+01 | 5.24E+01 | 5.20E+01 | 5.23E+01 | 5.06E+01 | 5.06E+01 | 5.29E+01 | 11.59 | x | x | x |
| 138.1568 | 1.60 | 9.53E+01 | 9.04E+01 | 9.75E+01 | 1.06E+02 | 1.09E+02 | 1.20E+02 | 1.12E+02 | 1.10E+02 | 1.18E+02 | 1.05E+02 | 5.75E+01 | 1.20E+02 | 1.19E+02 | 1.21E+02 | 1.19E+02 | 1.18E+02 | 1.24E+02 | 15.15 |  | x | x |
| 138.2073 | 1.60 | 7.23E+01 | 6.63E+01 | 7.36E+01 | 7.96E+01 | 8.21E+01 | 9.15E+01 | 7.94E+01 | 7.97E+01 | 9.57E+01 | 8.09E+01 | 1.30E+02 | 8.32E+01 | 8.66E+01 | 8.51E+01 | 7.67E+01 | 7.82E+01 | 8.23E+01 | 16.5 |  | x | x |
| 160.0369 | 1.60 | 2.07E+02 | 1.86E+02 | 2.01E+02 | 2.08E+02 | 2.28E+02 | 2.46E+02 | 2.42E+02 | 2.31E+02 | 2.47E+02 | 2.19E+02 | 2.15E+02 | 2.36E+02 | 2.39E+02 | 2.46E+02 | 2.48E+02 | 2.46E+02 | 2.58E+02 | 8.85 | x | x | x |
| 253.0953 | 1.60 | 1.67E+02 | 1.51E+02 | 1.74E+02 | 1.95E+02 | 2.14E+02 | 2.16E+02 | 2.17E+02 | 2.09E+02 | 2.25E+02 | 1.94E+02 | 4.79E+01 | 2.16E+02 | 2.14E+02 | 2.20E+02 | 2.09E+02 | 2.17E+02 | 2.27E+02 | 22.34 |  |  | x |
| 297.0848 | 1.60 | 3.87E+02 | 3.43E+02 | 4.02E+02 | 4.47E+02 | 4.92E+02 | 5.20E+02 | 5.12E+02 | 5.20E+02 | 5.35E+02 | 4.63E+02 | 1.21E+01 | 5.00E+02 | 5.28E+02 | 5.05E+02 | 5.18E+02 | 5.16E+02 | 5.25E+02 | 27.96 |  |  | x |
| 304.1029 | 1.60 | 4.44E+01 | 3.58E+01 | 3.90E+01 | 4.86E+01 | 3.81E+01 | 4.17E+01 | 4.03E+01 | 4.18E+01 | 3.99E+01 | 4.08E+01 | 6.41E+01 | 4.68E+01 | 4.43E+01 | 4.07E+01 | 4.20E+01 | 4.37E+01 | 4.83E+01 | 14.52 | x | x | x |
| 306.0818 | 1.60 | 7.58E+01 | 6.95E+01 | 7.32E+01 | 6.99E+01 | 7.75E+01 | 7.63E+01 | 7.05E+01 | 7.53E+01 | 8.43E+01 | 7.43E+01 | 4.02E+01 | 6.95E+01 | 6.98E+01 | 7.16E+01 | 7.57E+01 | 7.44E+01 | 7.36E+01 | 12.49 | x | x | x |
| 344.0462 | 1.60 | 1.91E+01 | 1.71E+01 | 1.81E+01 | 2.01E+01 | 1.80E+01 | 2.17E+01 | 1.90E+01 | 1.79E+01 | 1.45E+01 | 1.77E+01 | 6.22E+00 | 1.92E+01 | 2.08E+01 | 1.82E+01 | 2.16E+01 | 1.88E+01 | 1.98E+01 | 19.45 |  | x | x |
| 158.0924 | 1.61 | 1.15E+00 | 1.64E+00 | 1.62E+00 | 1.02E+00 | 2.72E+00 | 1.54E+00 | 1.59E+00 | 1.82E+00 | 1.98E+00 | 1.88E+00 | 1.33E+00 | 1.94E+00 | 2.24E+00 | 1.63E+00 | 1.29E+00 | 1.89E+00 | 1.82E+00 | 23.87 |  |  | x |
| 205.0027 | 1.61 | 2.83E+00 | 2.62E+00 | 2.06E+00 | 1.99E+00 | 1.25E+00 | 3.12E+00 | 2.26E+00 | 2.68E+00 | 1.25E+00 | 2.11E+00 | 1.31E+00 | 2.41E+00 | 2.51E+00 | 1.91E+00 | 2.97E+00 | 2.25E+00 | 3.62E+00 | 28.67 |  |  | x |
| 236.9988 | 1.61 | 2.49E+02 | 2.40E+02 | 2.51E+02 | 2.23E+02 | 2.27E+02 | 2.22E+02 | 2.40E+02 | 2.34E+02 | 2.81E+02 | 2.55E+02 | 1.14E+02 | 2.20E+02 | 2.63E+02 | 2.80E+02 | 1.77E+02 | 2.60E+02 | 2.91E+02 | 17.66 |  | x | x |
| 280.9624 | 1.61 | 6.57E+01 | 5.78E+01 | 6.40E+01 | 5.40E+01 | 5.28E+01 | 5.41E+01 | 6.39E+01 | 5.88E+01 | 7.48E+01 | 6.90E+01 | 2.93E+01 | 6.01E+01 | 7.85E+01 | 8.96E+01 | 4.77E+01 | 7.93E+01 | 8.88E+01 | 23.86 |  |  | x |
| 347.0775 | 1.61 | 5.13E+01 | 4.49E+01 | 5.43E+01 | 5.38E+01 | 5.46E+01 | 5.86E+01 | 4.94E+01 | 5.99E+01 | 4.93E+01 | 4.99E+01 | 5.07E+00 | 5.82E+01 | 5.47E+01 | 5.50E+01 | 6.98E+01 | 5.83E+01 | 5.69E+01 | 25.57 |  |  | x |
| 154.9694 | 1.62 | 2.42E+01 | 1.88E+01 | 1.85E+01 | 1.11E+01 | 1.46E+01 | 1.20E+01 | 1.76E+01 | 1.45E+01 | 1.98E+01 | 1.88E+01 | 2.42E+01 | 1.42E+01 | 1.75E+01 | 2.13E+01 | 1.01E+01 | 2.02E+01 | 2.42E+01 | 25.13 |  |  | x |
| 218.1490 | 1.62 | 2.11E+01 | 2.25E+01 | 1.78E+01 | 2.90E+01 | 2.21E+01 | 2.44E+01 | 2.10E+01 | 2.37E+01 | 2.40E+01 | 3.19E+01 | 4.88E+01 | 2.67E+01 | 2.52E+01 | 2.74E+01 | 2.77E+01 | 3.25E+01 | 2.89E+01 | 25.89 |  |  | x |
| 317.0720 | 1.62 | 9.51E+00 | 7.90E+00 | 7.54E+00 | 8.94E+00 | 6.45E+00 | 9.13E+00 | 6.90E+00 | 9.72E+00 | 5.48E+00 | 7.27E+00 | 2.55E+00 | 4.21E+00 | 9.41E+00 | 8.51E+00 | 6.77E+00 | 6.71E+00 | 8.99E+00 | 26.7 |  |  | x |
| 348.9502 | 1.62 | 1.59E+02 | 1.37E+02 | 1.31E+02 | 9.84E+01 | 1.19E+02 | 1.16E+02 | 1.26E+02 | 1.30E+02 | 1.41E+02 | 1.33E+02 | 2.35E+01 | 1.26E+02 | 1.38E+02 | 1.51E+02 | 8.86E+01 | 1.52E+02 | 1.62E+02 | 26.07 |  |  | x |
| 124.0871 | 1.63 | 3.97E+02 | 3.55E+02 | 3.97E+02 | 4.09E+02 | 4.38E+02 | 4.47E+02 | 4.48E+02 | 4.41E+02 | 4.88E+02 | 4.30E+02 | 4.66E+02 | 4.59E+02 | 4.46E+02 | 4.47E+02 | 4.40E+02 | 4.62E+02 | 4.60E+02 | 7.32 | x | x | x |
| 329.0925 | 1.63 | 4.53E+02 | 3.71E+02 | 4.31E+02 | 4.21E+02 | 4.23E+02 | 4.50E+02 | 4.35E+02 | 4.01E+02 | 4.29E+02 | 3.79E+02 | 1.65E+02 | 4.55E+02 | 4.05E+02 | 4.08E+02 | 3.77E+02 | 3.76E+02 | 4.02E+02 | 16.61 |  | x | x |
| 160.9938 | 1.63 | 1.43E+01 | 1.46E+01 | 1.36E+01 | 1.32E+01 | 1.77E+01 | 1.55E+01 | 1.33E+01 | 1.84E+01 | 1.48E+01 | 1.47E+01 | 1.91E+01 | 1.64E+01 | 1.63E+01 | 1.59E+01 | 1.74E+01 | 1.63E+01 | 1.71E+01 | 11.22 | x | x | x |
| 221.0263 | 1.63 | 1.79E+02 | 1.53E+02 | 1.73E+02 | 1.66E+02 | 1.74E+02 | 1.79E+02 | 1.75E+02 | 1.71E+02 | 1.79E+02 | 1.68E+02 | 1.34E+02 | 1.85E+02 | 1.77E+02 | 1.79E+02 | 1.65E+02 | 1.64E+02 | 1.77E+02 | 7.09 | x | x | x |
| 187.0457 | 1.63 | 5.20E+01 | 4.82E+01 | 5.16E+01 | 5.38E+01 | 5.51E+01 | 5.78E+01 | 5.36E+01 | 5.61E+01 | 5.91E+01 | 5.35E+01 | 5.67E+01 | 5.69E+01 | 5.55E+01 | 5.06E+01 | 5.22E+01 | 5.61E+01 | 5.57E+01 | 5.19 | x | x | x |
| 430.9155 | 1.63 | 1.07E+02 | 9.45E+01 | 9.02E+01 | 7.29E+01 | 9.01E+01 | 8.36E+01 | 8.75E+01 | 9.64E+01 | 9.13E+01 | 9.74E+01 | 1.18E+01 | 9.91E+01 | 1.07E+02 | 1.02E+02 | 1.09E+02 | 1.10E+02 | 1.11E+02 | 25.19 |  |  | x |
| 217.1297 | 1.63 | 1.71E+02 | 1.62E+02 | 1.78E+02 | 2.04E+02 | 2.19E+02 | 2.22E+02 | 2.18E+02 | 2.23E+02 | 2.34E+02 | 2.14E+02 | 1.54E+02 | 2.13E+02 | 2.22E+02 | 2.12E+02 | 2.12E+02 | 2.20E+02 | 2.25E+02 | 11.7 | x | x | x |
| 256.0681 | 1.63 | 2.09E+03 | 1.81E+03 | 2.04E+03 | 2.06E+03 | 2.15E+03 | 2.23E+03 | 2.20E+03 | 2.06E+03 | 2.20E+03 | 1.95E+03 | 1.32E+03 | 2.18E+03 | 2.05E+03 | 2.02E+03 | 1.85E+03 | 1.97E+03 | 2.04E+03 | 10.64 | x | x | x |
| 272.1345 | 1.63 | 7.43E+01 | 7.22E+01 | 6.87E+01 | 7.80E+01 | 8.03E+01 | 8.20E+01 | 7.70E+01 | 7.54E+01 | 7.68E+01 | 6.96E+01 | 4.69E+01 | 7.95E+01 | 6.87E+01 | 6.66E+01 | 6.60E+01 | 6.97E+01 | 7.09E+01 | 11.29 | x | x | x |
| 245.0763 | 1.63 | 1.26E+02 | 1.14E+02 | 1.27E+02 | 1.29E+02 | 1.43E+02 | 1.38E+02 | 1.30E+02 | 1.31E+02 | 1.36E+02 | 1.17E+02 | 1.23E+02 | 1.30E+02 | 1.34E+02 | 1.37E+02 | 1.33E+02 | 1.28E+02 | 1.37E+02 | 5.71 | x | x | x |
| 256.2792 | 1.63 | 2.03E+01 | 2.18E+01 | 2.24E+01 | 2.15E+01 | 2.33E+01 | 2.16E+01 | 2.26E+01 | 2.36E+01 | 2.01E+01 | 2.06E+01 | 1.52E+01 | 1.69E+01 | 2.00E+01 | 1.82E+01 | 2.12E+01 | 1.60E+01 | 1.86E+01 | 12.3 | x | x | x |
| 261.0935 | 1.63 | 1.11E+02 | 1.04E+02 | 1.14E+02 | 1.05E+02 | 1.13E+02 | 1.21E+02 | 1.16E+02 | 1.16E+02 | 1.16E+02 | 1.02E+02 | 1.02E+02 | 1.12E+02 | 1.10E+02 | 1.09E+02 | 1.05E+02 | 1.05E+02 | 1.11E+02 | 5.05 | x | x | x |
| 312.0157 | 1.63 | 4.28E+01 | 3.44E+01 | 3.39E+01 | 3.02E+01 | 2.73E+01 | 3.03E+01 | 3.14E+01 | 2.55E+01 | 3.23E+01 | 2.31E+01 | 2.01E+00 | 3.19E+01 | 2.80E+01 | 2.63E+01 | 2.63E+01 | 2.60E+01 | 2.64E+01 | 29.05 |  |  | x |
| 329.2499 | 1.63 | 2.84E+00 | 2.47E+00 | 3.02E+00 | 2.10E+00 | 2.05E+00 | 2.94E+00 | 2.55E+00 | 2.08E+00 | 2.66E+00 | 2.18E+00 | 1.24E-01 | 2.26E+00 | 3.04E+00 | 1.97E+00 | 2.45E+00 | 2.50E+00 | 2.64E+00 | 28.48 |  |  | x |
| 374.0781 | 1.63 | 1.06E+02 | 8.10E+01 | 8.65E+01 | 8.99E+01 | 8.87E+01 | 8.90E+01 | 8.84E+01 | 8.47E+01 | 8.32E+01 | 7.76E+01 | 8.91E+00 | 8.09E+01 | 8.29E+01 | 7.67E+01 | 7.74E+01 | 8.61E+01 | 8.76E+01 | 24.43 |  |  | x |
| 392.0429 | 1.63 | 1.84E+02 | 1.76E+02 | 1.69E+02 | 1.58E+02 | 1.69E+02 | 1.73E+02 | 1.58E+02 | 1.54E+02 | 1.69E+02 | 1.38E+02 | 1.23E+02 | 1.66E+02 | 1.64E+02 | 1.55E+02 | 1.59E+02 | 1.61E+02 | 1.62E+02 | 8.87 | x | x | x |
| 419.0642 | 1.63 | 1.61E+02 | 1.29E+02 | 1.36E+02 | 1.27E+02 | 1.24E+02 | 1.25E+02 | 1.10E+02 | 1.20E+02 | 1.15E+02 | 1.03E+02 | 3.74E+01 | 1.20E+02 | 1.05E+02 | 1.13E+02 | 9.58E+01 | 9.34E+01 | 1.02E+02 | 22.52 |  |  | x |
| 454.1049 | 1.63 | 1.29E+02 | 9.74E+01 | 9.92E+01 | 8.66E+01 | 9.06E+01 | 9.73E+01 | 9.32E+01 | 9.12E+01 | 9.94E+01 | 8.15E+01 | 2.07E+01 | 8.86E+01 | 7.99E+01 | 8.87E+01 | 7.65E+01 | 8.72E+01 | 8.70E+01 | 23.63 |  |  | x |
| 482.1446 | 1.63 | 1.44E+02 | 1.21E+02 | 1.14E+02 | 1.06E+02 | 8.71E+01 | 1.01E+02 | 8.81E+01 | 8.50E+01 | 8.67E+01 | 7.89E+01 | 2.29E+01 | 8.38E+01 | 8.41E+01 | 7.68E+01 | 6.61E+01 | 8.31E+01 | 7.36E+01 | 29.1 |  |  | x |
| 492.9785 | 1.63 | 2.76E+01 | 2.17E+01 | 2.44E+01 | 1.95E+01 | 2.00E+01 | 2.36E+01 | 1.82E+01 | 2.71E+01 | 1.91E+01 | 2.08E+01 | 4.04E-01 | 2.19E+01 | 1.97E+01 | 2.53E+01 | 2.38E+01 | 2.45E+01 | 2.57E+01 | 28.63 |  |  | x |
| 560.9663 | 1.63 | 7.93E+01 | 7.28E+01 | 7.08E+01 | 7.16E+01 | 6.09E+01 | 6.67E+01 | 6.36E+01 | 6.72E+01 | 6.71E+01 | 6.19E+01 | 7.22E-01 | 6.44E+01 | 6.24E+01 | 7.11E+01 | 7.02E+01 | 6.03E+01 | 7.34E+01 | 26.74 |  |  | x |
| 109.0763 | 1.64 | 5.35E+01 | 4.72E+01 | 5.60E+01 | 5.18E+01 | 5.42E+01 | 5.80E+01 | 5.91E+01 | 5.63E+01 | 5.73E+01 | 5.41E+01 | 6.89E+01 | 6.11E+01 | 5.95E+01 | 6.17E+01 | 5.59E+01 | 5.87E+01 | 6.34E+01 | 8.56 | x | x | x |
| 158.0297 | 1.64 | 5.09E+00 | 2.28E+00 | 3.72E+00 | 4.99E+00 | 3.77E+00 | 4.10E+00 | 4.21E+00 | 3.69E+00 | 4.32E+00 | 3.80E+00 | 4.37E+00 | 7.01E+00 | 2.74E+00 | 5.25E+00 | 3.94E+00 | 4.04E+00 | 4.18E+00 | 24.68 |  |  | x |
| 132.0772 | 1.64 | 1.58E+02 | 1.43E+02 | 1.63E+02 | 1.83E+02 | 1.97E+02 | 2.12E+02 | 2.09E+02 | 2.06E+02 | 2.11E+02 | 1.91E+02 | 2.13E+02 | 2.24E+02 | 1.99E+02 | 2.07E+02 | 1.96E+02 | 2.01E+02 | 2.18E+02 | 11.46 | x | x | x |
| 153.0660 | 1.64 | 1.12E+02 | 1.08E+02 | 1.17E+02 | 1.19E+02 | 1.25E+02 | 1.23E+02 | 1.20E+02 | 1.18E+02 | 1.28E+02 | 1.14E+02 | 1.33E+02 | 1.23E+02 | 1.14E+02 | 1.19E+02 | 1.12E+02 | 1.15E+02 | 1.22E+02 | 5.39 | x | x | x |
| 233.2181 | 1.64 | 3.99E+00 | 3.12E+00 | 3.88E+00 | 2.92E+00 | 3.79E+00 | 4.34E+00 | 5.45E+00 | 4.48E+00 | 5.20E+00 | 5.17E+00 | 4.30E+00 | 4.83E+00 | 5.29E+00 | 4.76E+00 | 5.15E+00 | 5.09E+00 | 4.65E+00 | 16.74 |  | x | x |
| 259.0928 | 1.64 | 1.38E+02 | 1.22E+02 | 1.38E+02 | 1.40E+02 | 1.49E+02 | 1.57E+02 | 1.47E+02 | 1.39E+02 | 1.51E+02 | 1.33E+02 | 1.96E+02 | 1.58E+02 | 1.42E+02 | 1.41E+02 | 1.40E+02 | 1.39E+02 | 1.49E+02 | 10.76 | x | x | x |
| 226.0459 | 1.64 | 3.60E+01 | 2.91E+01 | 3.80E+01 | 2.66E+01 | 2.83E+01 | 2.60E+01 | 2.69E+01 | 2.66E+01 | 2.49E+01 | 2.42E+01 | 1.22E+01 | 2.70E+01 | 2.22E+01 | 2.05E+01 | 2.34E+01 | 2.33E+01 | 2.41E+01 | 21.98 |  |  | x |
| 272.0417 | 1.64 | 7.16E+01 | 6.21E+01 | 7.73E+01 | 7.87E+01 | 7.31E+01 | 8.94E+01 | 8.63E+01 | 7.68E+01 | 8.12E+01 | 6.54E+01 | 5.01E+01 | 8.53E+01 | 7.33E+01 | 6.73E+01 | 6.33E+01 | 6.19E+01 | 6.63E+01 | 14.34 | x | x | x |
| 315.0770 | 1.64 | 1.83E+01 | 1.86E+01 | 1.67E+01 | 1.81E+01 | 1.69E+01 | 1.87E+01 | 1.80E+01 | 2.04E+01 | 2.03E+01 | 1.45E+01 | 7.60E+00 | 1.77E+01 | 2.00E+01 | 1.68E+01 | 1.62E+01 | 1.76E+01 | 1.85E+01 | 16.9 |  | x | x |
| 326.0841 | 1.64 | 1.08E+01 | 9.45E+00 | 1.14E+01 | 1.32E+01 | 1.35E+01 | 1.03E+01 | 1.23E+01 | 1.12E+01 | 1.20E+01 | 1.10E+01 | 5.49E+00 | 1.34E+01 | 1.24E+01 | 1.09E+01 | 9.82E+00 | 1.07E+01 | 1.26E+01 | 17.06 |  | x | x |
| 327.1036 | 1.64 | 5.24E+01 | 4.31E+01 | 4.18E+01 | 4.20E+01 | 4.01E+01 | 4.75E+01 | 4.41E+01 | 4.33E+01 | 4.47E+01 | 4.48E+01 | 4.09E+01 | 5.02E+01 | 4.52E+01 | 4.78E+01 | 3.75E+01 | 4.05E+01 | 4.91E+01 | 8.88 | x | x | x |
| 369.0701 | 1.64 | 4.03E+01 | 3.47E+01 | 3.80E+01 | 3.14E+01 | 3.46E+01 | 3.34E+01 | 3.38E+01 | 3.59E+01 | 3.64E+01 | 3.15E+01 | 2.22E+00 | 3.55E+01 | 3.49E+01 | 2.75E+01 | 3.03E+01 | 3.12E+01 | 3.31E+01 | 25.77 |  |  | x |
| 386.1377 | 1.64 | 5.25E+01 | 5.14E+01 | 5.40E+01 | 5.66E+01 | 5.35E+01 | 5.41E+01 | 5.10E+01 | 4.31E+01 | 5.02E+01 | 4.36E+01 | 1.78E+01 | 5.77E+01 | 5.10E+01 | 4.13E+01 | 3.78E+01 | 4.39E+01 | 4.65E+01 | 20.06 |  |  | x |
| 395.1011 | 1.64 | 4.93E+01 | 4.92E+01 | 5.97E+01 | 2.99E+01 | 5.64E+01 | 4.82E+01 | 5.97E+01 | 5.39E+01 | 6.70E+01 | 5.41E+01 | 2.69E+01 | 5.61E+01 | 6.44E+01 | 6.13E+01 | 4.66E+01 | 6.38E+01 | 5.97E+01 | 20.84 |  |  | x |
| 406.1064 | 1.64 | 1.21E+01 | 1.21E+01 | 1.75E+01 | 1.25E+01 | 1.25E+01 | 1.11E+01 | 1.43E+01 | 1.10E+01 | 1.06E+01 | 9.40E+00 | 4.42E-01 | 1.42E+01 | 1.30E+01 | 1.31E+01 | 1.02E+01 | 1.33E+01 | 1.33E+01 | 29.5 |  |  | x |
| 407.1271 | 1.64 | 3.72E+01 | 3.03E+01 | 3.11E+01 | 3.20E+01 | 3.00E+01 | 2.20E+01 | 2.89E+01 | 2.69E+01 | 2.97E+01 | 2.86E+01 | 1.03E+01 | 2.54E+01 | 3.02E+01 | 2.70E+01 | 2.90E+01 | 2.97E+01 | 2.74E+01 | 19.8 |  | x | x |
| 409.1190 | 1.64 | 4.63E+02 | 3.93E+02 | 4.48E+02 | 4.03E+02 | 3.80E+02 | 3.72E+02 | 3.82E+02 | 3.59E+02 | 3.76E+02 | 3.41E+02 | 5.32E+01 | 3.68E+02 | 3.60E+02 | 3.48E+02 | 3.24E+02 | 3.43E+02 | 3.49E+02 | 24.17 |  |  | x |
| 198.0869 | 1.65 | 4.44E+01 | 3.62E+01 | 4.27E+01 | 4.81E+01 | 5.12E+01 | 5.23E+01 | 5.51E+01 | 5.11E+01 | 5.23E+01 | 4.62E+01 | 4.73E+01 | 5.64E+01 | 5.17E+01 | 4.97E+01 | 4.68E+01 | 4.87E+01 | 5.17E+01 | 9.96 | x | x | x |
| 202.0824 | 1.65 | 1.11E+01 | 1.17E+01 | 1.43E+01 | 1.30E+01 | 1.35E+01 | 1.48E+01 | 1.37E+01 | 1.34E+01 | 1.46E+01 | 1.36E+01 | 1.73E+01 | 1.78E+01 | 1.31E+01 | 1.27E+01 | 1.29E+01 | 1.40E+01 | 1.55E+01 | 12.45 | x | x | x |
| 223.1909 | 1.65 | 4.67E+00 | 6.79E+00 | 6.54E+00 | 5.63E+00 | 7.99E+00 | 7.35E+00 | 4.32E+00 | 5.75E+00 | 8.31E+00 | 5.87E+00 | 2.23E+00 | 6.90E+00 | 7.82E+00 | 6.82E+00 | 4.13E+00 | 6.15E+00 | 7.89E+00 | 26.32 |  |  | x |
| 239.0138 | 1.65 | 7.84E+01 | 7.27E+01 | 7.54E+01 | 8.35E+01 | 8.59E+01 | 8.90E+01 | 8.07E+01 | 9.11E+01 | 8.93E+01 | 7.60E+01 | 3.79E+01 | 8.92E+01 | 8.85E+01 | 8.89E+01 | 1.01E+02 | 8.63E+01 | 9.80E+01 | 16.75 |  | x | x |
| 266.0756 | 1.65 | 1.80E+02 | 1.60E+02 | 1.81E+02 | 1.86E+02 | 2.01E+02 | 2.17E+02 | 1.97E+02 | 2.19E+02 | 2.15E+02 | 1.86E+02 | 6.18E+01 | 2.08E+02 | 2.15E+02 | 2.29E+02 | 2.21E+02 | 2.17E+02 | 2.25E+02 | 20.18 |  |  | x |
| 243.1700 | 1.65 | 2.36E+02 | 2.11E+02 | 2.23E+02 | 2.57E+02 | 2.76E+02 | 2.80E+02 | 2.63E+02 | 2.79E+02 | 2.66E+02 | 2.15E+02 | 1.00E+02 | 2.77E+02 | 2.42E+02 | 2.54E+02 | 2.92E+02 | 2.38E+02 | 2.66E+02 | 18.1 |  | x | x |
| 270.1554 | 1.65 | 4.85E+01 | 5.05E+01 | 5.35E+01 | 5.54E+01 | 5.05E+01 | 6.15E+01 | 5.54E+01 | 5.38E+01 | 4.78E+01 | 4.85E+01 | 1.75E+01 | 6.46E+01 | 5.15E+01 | 4.92E+01 | 5.93E+01 | 5.23E+01 | 5.50E+01 | 19.33 |  | x | x |
| 279.6854 | 1.65 | 1.18E+01 | 1.13E+01 | 1.35E+01 | 1.06E+01 | 9.11E+00 | 1.13E+01 | 9.38E+00 | 1.13E+01 | 7.70E+00 | 7.21E+00 | 4.26E+00 | 1.31E+01 | 9.96E+00 | 9.70E+00 | 8.55E+00 | 8.44E+00 | 7.55E+00 | 24.01 |  |  | x |
| 281.0754 | 1.65 | 2.13E+02 | 2.02E+02 | 2.24E+02 | 2.37E+02 | 2.29E+02 | 2.44E+02 | 2.32E+02 | 2.40E+02 | 2.44E+02 | 2.19E+02 | 2.35E+02 | 2.47E+02 | 2.29E+02 | 2.27E+02 | 2.24E+02 | 2.31E+02 | 2.25E+02 | 5.06 | x | x | x |
| 283.1387 | 1.65 | 1.60E+02 | 1.46E+02 | 1.60E+02 | 1.58E+02 | 1.52E+02 | 1.69E+02 | 1.65E+02 | 1.71E+02 | 1.69E+02 | 1.48E+02 | 3.33E+01 | 1.72E+02 | 1.58E+02 | 1.64E+02 | 1.58E+02 | 1.58E+02 | 1.63E+02 | 20.75 |  |  | x |
| 302.2438 | 1.65 | 2.45E+01 | 2.26E+01 | 2.01E+01 | 2.80E+01 | 3.58E+01 | 3.88E+01 | 3.51E+01 | 4.00E+01 | 4.16E+01 | 2.85E+01 | 1.02E+01 | 3.80E+01 | 3.75E+01 | 4.07E+01 | 3.67E+01 | 3.63E+01 | 3.93E+01 | 27.07 |  |  | x |
| 411.2973 | 1.65 | 1.08E+01 | 8.79E+00 | 1.08E+01 | 8.58E+00 | 8.13E+00 | 9.10E+00 | 9.03E+00 | 1.02E+01 | 9.85E+00 | 7.00E+00 | 6.37E-01 | 1.04E+01 | 9.20E+00 | 1.02E+01 | 8.66E+00 | 9.29E+00 | 8.47E+00 | 26.52 |  |  | x |
| 455.0394 | 1.65 | 5.75E+01 | 5.72E+01 | 5.99E+01 | 5.62E+01 | 4.71E+01 | 5.15E+01 | 4.87E+01 | 5.39E+01 | 5.96E+01 | 3.99E+01 | 4.71E-01 | 5.26E+01 | 5.19E+01 | 5.74E+01 | 5.60E+01 | 5.31E+01 | 5.52E+01 | 27.37 |  |  | x |
| 166.0957 | 1.66 | 7.82E+01 | 7.84E+01 | 8.50E+01 | 9.22E+01 | 9.54E+01 | 1.03E+02 | 9.01E+01 | 9.83E+01 | 1.00E+02 | 9.17E+01 | 1.19E+02 | 9.47E+01 | 9.35E+01 | 9.06E+01 | 8.98E+01 | 9.39E+01 | 9.72E+01 | 9.96 | x | x | x |
| 169.9865 | 1.66 | 5.66E+00 | 3.65E+00 | 5.26E+00 | 1.95E+00 | 3.43E+00 | 2.75E+00 | 4.56E+00 | 2.43E+00 | 4.03E+00 | 4.93E+00 | 3.88E+00 | 3.70E+00 | 4.35E+00 | 5.15E+00 | 3.69E+00 | 3.89E+00 | 5.22E+00 | 25.85 |  |  | x |
| 206.1517 | 1.66 | 4.49E+01 | 4.91E+01 | 4.90E+01 | 5.27E+01 | 4.43E+01 | 5.50E+01 | 6.18E+01 | 6.67E+01 | 5.33E+01 | 4.66E+01 | 4.01E+01 | 5.40E+01 | 5.01E+01 | 5.57E+01 | 5.98E+01 | 6.15E+01 | 5.29E+01 | 13.27 | x | x | x |
| 212.1039 | 1.66 | 6.26E+03 | 5.88E+03 | 6.67E+03 | 6.93E+03 | 7.34E+03 | 7.92E+03 | 7.77E+03 | 7.54E+03 | 7.99E+03 | 7.16E+03 | 4.74E+03 | 7.72E+03 | 7.61E+03 | 7.80E+03 | 7.19E+03 | 7.61E+03 | 7.90E+03 | 12.09 | x | x | x |
| 212.1758 | 1.66 | 3.86E+01 | 3.45E+01 | 4.37E+01 | 4.49E+01 | 5.03E+01 | 4.82E+01 | 4.85E+01 | 4.60E+01 | 5.36E+01 | 4.51E+01 | 2.16E+01 | 5.12E+01 | 4.82E+01 | 4.70E+01 | 4.11E+01 | 4.73E+01 | 4.91E+01 | 16.91 |  | x | x |
| 212.2286 | 1.66 | 5.52E+01 | 4.83E+01 | 5.84E+01 | 5.66E+01 | 6.17E+01 | 6.69E+01 | 6.59E+01 | 6.81E+01 | 7.27E+01 | 6.22E+01 | 3.07E+01 | 6.75E+01 | 7.10E+01 | 7.83E+01 | 6.57E+01 | 7.14E+01 | 7.50E+01 | 17.99 |  | x | x |
| 232.0933 | 1.66 | 1.40E+02 | 1.19E+02 | 1.43E+02 | 1.39E+02 | 1.40E+02 | 1.44E+02 | 1.40E+02 | 1.34E+02 | 1.43E+02 | 1.28E+02 | 6.00E+01 | 1.49E+02 | 1.30E+02 | 1.34E+02 | 1.33E+02 | 1.30E+02 | 1.33E+02 | 15.06 |  | x | x |
| 269.1248 | 1.66 | 1.01E+03 | 9.95E+02 | 1.14E+03 | 1.22E+03 | 1.17E+03 | 1.39E+03 | 1.33E+03 | 1.28E+03 | 1.28E+03 | 1.16E+03 | 3.36E+02 | 1.41E+03 | 1.32E+03 | 1.31E+03 | 1.32E+03 | 1.30E+03 | 1.37E+03 | 21.08 |  |  | x |
| 269.2660 | 1.66 | 1.08E+01 | 1.12E+01 | 1.38E+01 | 1.41E+01 | 1.17E+01 | 1.52E+01 | 1.44E+01 | 1.35E+01 | 1.33E+01 | 1.15E+01 | 1.02E+00 | 1.74E+01 | 1.54E+01 | 1.42E+01 | 1.61E+01 | 1.53E+01 | 1.70E+01 | 28.01 |  |  | x |
| 293.1702 | 1.66 | 1.95E+01 | 2.28E+01 | 2.68E+01 | 2.41E+01 | 1.80E+01 | 2.55E+01 | 2.64E+01 | 2.35E+01 | 1.84E+01 | 2.08E+01 | 1.09E+01 | 3.48E+01 | 2.46E+01 | 2.04E+01 | 2.84E+01 | 2.38E+01 | 2.71E+01 | 22.43 |  |  | x |
| 321.1157 | 1.66 | 3.75E+01 | 3.54E+01 | 3.53E+01 | 3.55E+01 | 3.42E+01 | 3.68E+01 | 3.67E+01 | 3.57E+01 | 3.33E+01 | 3.10E+01 | 1.25E+01 | 4.07E+01 | 3.39E+01 | 3.76E+01 | 4.21E+01 | 3.79E+01 | 3.78E+01 | 18.16 |  | x | x |
| 339.1188 | 1.66 | 4.34E+01 | 4.07E+01 | 5.11E+01 | 4.12E+01 | 3.70E+01 | 4.61E+01 | 4.37E+01 | 3.48E+01 | 2.57E+01 | 2.35E+01 | 5.49E+00 | 4.65E+01 | 3.85E+01 | 3.50E+01 | 4.06E+01 | 3.42E+01 | 4.24E+01 | 29.06 |  |  | x |
| 383.2022 | 1.66 | 8.00E+01 | 7.38E+01 | 7.86E+01 | 6.97E+01 | 6.29E+01 | 7.37E+01 | 6.51E+01 | 5.89E+01 | 6.05E+01 | 5.34E+01 | 1.59E+00 | 6.72E+01 | 6.69E+01 | 5.79E+01 | 5.93E+01 | 5.82E+01 | 6.49E+01 | 27.91 |  |  | x |
| 169.0368 | 1.67 | 7.83E+00 | 7.49E+00 | 7.92E+00 | 7.91E+00 | 9.19E+00 | 9.08E+00 | 1.08E+01 | 6.82E+00 | 8.51E+00 | 8.17E+00 | 8.57E+00 | 8.18E+00 | 1.02E+01 | 6.88E+00 | 6.88E+00 | 6.43E+00 | 7.35E+00 | 14.56 | x | x | x |
| 331.0464 | 1.67 | 3.66E+01 | 3.32E+01 | 3.67E+01 | 3.28E+01 | 2.55E+01 | 3.29E+01 | 3.27E+01 | 2.87E+01 | 2.26E+01 | 2.03E+01 | 2.70E+00 | 3.61E+01 | 3.14E+01 | 2.68E+01 | 2.68E+01 | 2.81E+01 | 2.99E+01 | 28.62 |  |  | x |
| 124.0394 | 1.68 | 6.02E+01 | 5.56E+01 | 6.05E+01 | 6.13E+01 | 6.79E+01 | 7.15E+01 | 7.08E+01 | 6.99E+01 | 7.80E+01 | 6.57E+01 | 6.82E+01 | 7.26E+01 | 6.52E+01 | 7.22E+01 | 6.20E+01 | 6.58E+01 | 7.50E+01 | 8.87 | x | x | x |
| 146.0803 | 1.68 | 6.85E+00 | 6.62E+00 | 7.79E+00 | 1.03E+01 | 9.62E+00 | 9.48E+00 | 7.12E+00 | 6.65E+00 | 9.14E+00 | 7.42E+00 | 1.53E+01 | 9.08E+00 | 8.64E+00 | 7.45E+00 | 6.82E+00 | 1.07E+01 | 9.25E+00 | 24.71 |  |  | x |
| 178.0865 | 1.68 | 1.88E+01 | 1.68E+01 | 2.00E+01 | 1.70E+01 | 1.96E+01 | 1.81E+01 | 1.48E+01 | 1.60E+01 | 1.51E+01 | 1.46E+01 | 1.24E+01 | 1.95E+01 | 1.39E+01 | 1.41E+01 | 1.48E+01 | 1.33E+01 | 1.53E+01 | 14.68 | x | x | x |
| 230.1097 | 1.68 | 5.11E+00 | 3.82E+00 | 6.45E+00 | 3.25E+00 | 4.55E+00 | 5.47E+00 | 5.97E+00 | 3.72E+00 | 4.74E+00 | 6.25E+00 | 5.12E+00 | 5.48E+00 | 2.88E+00 | 6.21E+00 | 5.79E+00 | 5.03E+00 | 6.12E+00 | 21.68 |  |  | x |
| 276.1201 | 1.68 | 3.70E+02 | 3.57E+02 | 3.57E+02 | 3.60E+02 | 3.68E+02 | 3.99E+02 | 4.06E+02 | 3.78E+02 | 3.93E+02 | 4.39E+02 | 7.22E+02 | 4.02E+02 | 3.82E+02 | 3.58E+02 | 3.95E+02 | 3.90E+02 | 4.17E+02 | 20.91 |  |  | x |
| 266.1606 | 1.68 | 1.63E+03 | 1.49E+03 | 1.62E+03 | 1.68E+03 | 1.71E+03 | 1.93E+03 | 1.85E+03 | 1.83E+03 | 1.86E+03 | 1.67E+03 | 6.26E+02 | 1.95E+03 | 1.83E+03 | 1.85E+03 | 1.90E+03 | 1.86E+03 | 1.92E+03 | 18.04 |  | x | x |
| 266.2404 | 1.68 | 1.09E+01 | 9.53E+00 | 1.09E+01 | 9.56E+00 | 1.09E+01 | 1.13E+01 | 7.13E+00 | 1.09E+01 | 1.14E+01 | 7.22E+00 | 1.91E+00 | 1.06E+01 | 1.03E+01 | 1.03E+01 | 9.96E+00 | 1.07E+01 | 1.11E+01 | 24.39 |  |  | x |
| 288.2271 | 1.68 | 1.76E+02 | 1.71E+02 | 1.85E+02 | 1.80E+02 | 1.83E+02 | 2.14E+02 | 2.14E+02 | 2.08E+02 | 1.98E+02 | 1.87E+02 | 6.36E+01 | 2.13E+02 | 2.12E+02 | 2.02E+02 | 2.12E+02 | 2.12E+02 | 2.26E+02 | 19.18 |  | x | x |
| 333.0444 | 1.68 | 1.09E+01 | 1.10E+01 | 1.19E+01 | 8.20E+00 | 8.30E+00 | 1.09E+01 | 1.03E+01 | 9.04E+00 | 5.40E+00 | 6.68E+00 | 2.12E+00 | 1.16E+01 | 9.86E+00 | 7.45E+00 | 7.12E+00 | 7.55E+00 | 9.33E+00 | 29.09 |  |  | x |
| 354.1180 | 1.68 | 1.13E+02 | 9.48E+01 | 1.16E+02 | 9.94E+01 | 1.03E+02 | 1.13E+02 | 8.99E+01 | 9.75E+01 | 1.02E+02 | 8.95E+01 | 7.98E+00 | 1.01E+02 | 9.70E+01 | 9.67E+01 | 8.96E+01 | 9.49E+01 | 9.62E+01 | 25.05 |  |  | x |
| 304.9559 | 1.69 | 2.12E+02 | 1.56E+02 | 1.66E+02 | 1.49E+02 | 1.66E+02 | 1.52E+02 | 1.46E+02 | 1.28E+02 | 1.47E+02 | 1.31E+02 | 8.23E+01 | 1.13E+02 | 1.06E+02 | 1.14E+02 | 8.80E+01 | 9.67E+01 | 1.07E+02 | 25.24 |  |  | x |
| 168.0443 | 1.69 | 2.64E+01 | 2.68E+01 | 3.16E+01 | 3.53E+01 | 3.42E+01 | 3.44E+01 | 3.41E+01 | 3.41E+01 | 3.70E+01 | 3.03E+01 | 2.75E+01 | 4.16E+01 | 3.90E+01 | 3.80E+01 | 3.68E+01 | 3.82E+01 | 4.12E+01 | 13.67 | x | x | x |
| 220.9678 | 1.69 | 1.73E+01 | 1.41E+01 | 1.26E+01 | 1.90E+01 | 1.85E+01 | 2.30E+01 | 1.81E+01 | 1.65E+01 | 1.83E+01 | 1.35E+01 | 4.85E+00 | 1.42E+01 | 1.17E+01 | 1.29E+01 | 1.28E+01 | 1.11E+01 | 8.90E+00 | 29.86 |  |  | x |
| 218.1050 | 1.69 | 1.50E+01 | 1.11E+01 | 1.19E+01 | 1.01E+01 | 1.63E+01 | 1.13E+01 | 1.27E+01 | 1.00E+01 | 1.50E+01 | 8.33E+00 | 8.72E+00 | 1.10E+01 | 9.47E+00 | 1.22E+01 | 8.67E+00 | 1.34E+01 | 1.23E+01 | 20.18 |  |  | x |
| 241.1191 | 1.69 | 7.40E+01 | 6.09E+01 | 6.88E+01 | 6.59E+01 | 7.42E+01 | 6.97E+01 | 7.49E+01 | 6.66E+01 | 8.07E+01 | 7.09E+01 | 5.97E+01 | 8.00E+01 | 7.40E+01 | 7.10E+01 | 6.78E+01 | 7.23E+01 | 8.20E+01 | 8.77 | x | x | x |
| 245.1121 | 1.69 | 1.10E+02 | 9.75E+01 | 1.09E+02 | 1.10E+02 | 1.13E+02 | 1.22E+02 | 1.31E+02 | 1.20E+02 | 1.28E+02 | 1.19E+02 | 2.17E+02 | 1.30E+02 | 1.15E+02 | 1.17E+02 | 1.08E+02 | 1.22E+02 | 1.23E+02 | 20.98 |  |  | x |
| 277.0901 | 1.69 | 4.19E+02 | 3.67E+02 | 4.11E+02 | 4.09E+02 | 4.28E+02 | 4.45E+02 | 4.29E+02 | 4.20E+02 | 4.67E+02 | 4.00E+02 | 5.18E+02 | 4.35E+02 | 4.25E+02 | 4.33E+02 | 3.92E+02 | 4.27E+02 | 4.38E+02 | 7.56 | x | x | x |
| 301.0156 | 1.69 | 6.00E+01 | 5.12E+01 | 5.35E+01 | 5.79E+01 | 5.24E+01 | 6.42E+01 | 4.80E+01 | 4.99E+01 | 4.35E+01 | 4.03E+01 | 8.13E+00 | 6.05E+01 | 3.40E+01 | 4.03E+01 | 4.68E+01 | 3.93E+01 | 4.65E+01 | 27.75 |  |  | x |
| 286.1100 | 1.69 | 2.47E+01 | 1.74E+01 | 2.21E+01 | 1.65E+01 | 2.26E+01 | 2.02E+01 | 1.83E+01 | 1.71E+01 | 3.18E+01 | 1.41E+01 | 9.37E+00 | 2.30E+01 | 2.11E+01 | 1.76E+01 | 1.16E+01 | 2.14E+01 | 2.10E+01 | 26.85 |  |  | x |
| 297.2277 | 1.69 | 8.09E+00 | 1.13E+01 | 1.53E+01 | 1.20E+01 | 1.14E+01 | 1.49E+01 | 1.17E+01 | 1.29E+01 | 1.38E+01 | 1.14E+01 | 1.11E+01 | 1.78E+01 | 1.47E+01 | 1.52E+01 | 1.68E+01 | 1.37E+01 | 1.11E+01 | 18.7 |  | x | x |
| 204.1256 | 1.70 | 1.30E+02 | 1.08E+02 | 1.21E+02 | 1.30E+02 | 1.27E+02 | 1.41E+02 | 1.45E+02 | 1.29E+02 | 1.55E+02 | 1.17E+02 | 6.10E+01 | 1.41E+02 | 1.31E+02 | 1.49E+02 | 1.26E+02 | 1.35E+02 | 1.60E+02 | 17.13 |  | x | x |
| 224.1282 | 1.70 | 8.27E+00 | 5.89E+00 | 8.21E+00 | 7.32E+00 | 6.86E+00 | 9.32E+00 | 8.50E+00 | 7.72E+00 | 9.56E+00 | 7.12E+00 | 4.55E+00 | 9.58E+00 | 7.93E+00 | 1.01E+01 | 6.12E+00 | 9.56E+00 | 7.50E+00 | 19.1 |  | x | x |
| 263.0741 | 1.70 | 1.13E+02 | 9.53E+01 | 1.12E+02 | 1.12E+02 | 1.13E+02 | 1.16E+02 | 1.22E+02 | 1.13E+02 | 1.21E+02 | 1.08E+02 | 9.79E+01 | 1.14E+02 | 1.18E+02 | 1.26E+02 | 1.01E+02 | 1.15E+02 | 1.09E+02 | 7.25 | x | x | x |
| 259.0551 | 1.70 | 1.84E+02 | 1.75E+02 | 1.98E+02 | 1.78E+02 | 1.96E+02 | 1.87E+02 | 1.74E+02 | 2.05E+02 | 1.94E+02 | 1.53E+02 | 6.95E+01 | 2.27E+02 | 1.79E+02 | 1.53E+02 | 2.24E+02 | 1.73E+02 | 1.96E+02 | 19.42 |  | x | x |
| 275.0279 | 1.70 | 4.18E+01 | 3.03E+01 | 4.13E+01 | 4.70E+01 | 5.63E+01 | 5.10E+01 | 5.51E+01 | 4.62E+01 | 5.98E+01 | 5.46E+01 | 4.53E+01 | 5.00E+01 | 5.77E+01 | 5.58E+01 | 4.69E+01 | 6.38E+01 | 5.81E+01 | 16.48 |  | x | x |
| 293.0625 | 1.70 | 3.13E+01 | 2.39E+01 | 3.14E+01 | 3.71E+01 | 3.86E+01 | 3.98E+01 | 3.88E+01 | 3.68E+01 | 5.03E+01 | 3.81E+01 | 1.39E+01 | 3.51E+01 | 3.53E+01 | 3.74E+01 | 3.29E+01 | 3.04E+01 | 2.97E+01 | 22.46 |  |  | x |
| 328.1075 | 1.70 | 1.34E+02 | 1.39E+02 | 1.39E+02 | 1.32E+02 | 1.29E+02 | 1.53E+02 | 1.44E+02 | 1.46E+02 | 1.68E+02 | 1.46E+02 | 1.16E+02 | 1.40E+02 | 1.44E+02 | 1.44E+02 | 1.33E+02 | 1.52E+02 | 1.50E+02 | 8.07 | x | x | x |
| 198.0877 | 1.71 | 1.73E+01 | 1.49E+01 | 2.02E+01 | 2.55E+01 | 2.55E+01 | 2.46E+01 | 2.47E+01 | 2.78E+01 | 3.11E+01 | 2.49E+01 | 2.54E+01 | 2.75E+01 | 2.58E+01 | 2.93E+01 | 2.45E+01 | 2.38E+01 | 2.92E+01 | 16.72 |  | x | x |
| 252.0979 | 1.71 | 1.81E+01 | 1.95E+01 | 2.45E+01 | 2.54E+01 | 1.89E+01 | 2.39E+01 | 1.92E+01 | 2.42E+01 | 2.02E+01 | 2.11E+01 | 2.05E+01 | 2.64E+01 | 2.70E+01 | 2.35E+01 | 2.40E+01 | 2.51E+01 | 2.42E+01 | 12.5 | x | x | x |
| 314.1226 | 1.71 | 3.99E+02 | 3.68E+02 | 3.97E+02 | 3.86E+02 | 3.94E+02 | 4.16E+02 | 4.17E+02 | 4.03E+02 | 4.44E+02 | 3.80E+02 | 2.83E+02 | 4.26E+02 | 4.07E+02 | 4.17E+02 | 4.09E+02 | 4.03E+02 | 4.37E+02 | 8.94 | x | x | x |
| 547.1079 | 1.71 | 5.58E+02 | 4.86E+02 | 5.18E+02 | 4.28E+02 | 4.22E+02 | 4.03E+02 | 3.88E+02 | 3.89E+02 | 4.41E+02 | 3.76E+02 | 1.67E+01 | 3.70E+02 | 3.95E+02 | 3.97E+02 | 3.39E+02 | 3.86E+02 | 3.96E+02 | 28.42 |  |  | x |
| 171.0765 | 1.72 | 5.40E+01 | 5.00E+01 | 5.44E+01 | 6.82E+01 | 7.05E+01 | 7.62E+01 | 7.71E+01 | 7.17E+01 | 8.29E+01 | 7.49E+01 | 6.46E+01 | 6.97E+01 | 7.98E+01 | 8.21E+01 | 5.93E+01 | 8.12E+01 | 7.66E+01 | 14.84 | x | x | x |
| 183.1122 | 1.72 | 1.43E+01 | 1.36E+01 | 1.59E+01 | 1.56E+01 | 1.63E+01 | 2.04E+01 | 1.78E+01 | 1.65E+01 | 2.07E+01 | 1.92E+01 | 2.69E+01 | 1.76E+01 | 1.67E+01 | 1.90E+01 | 2.01E+01 | 2.06E+01 | 1.87E+01 | 17.17 |  | x | x |
| 196.0628 | 1.72 | 1.43E+01 | 1.25E+01 | 1.60E+01 | 1.11E+01 | 1.68E+01 | 1.25E+01 | 1.59E+01 | 1.11E+01 | 1.30E+01 | 1.49E+01 | 1.32E+01 | 1.05E+01 | 1.26E+01 | 9.85E+00 | 1.73E+01 | 1.34E+01 | 1.42E+01 | 16.23 |  | x | x |
| 321.1160 | 1.72 | 3.90E+01 | 4.05E+01 | 4.54E+01 | 4.33E+01 | 3.79E+01 | 3.95E+01 | 3.89E+01 | 3.92E+01 | 4.24E+01 | 4.16E+01 | 1.44E+01 | 4.04E+01 | 4.20E+01 | 4.28E+01 | 3.49E+01 | 4.02E+01 | 4.24E+01 | 17.41 |  | x | x |
| 141.0500 | 1.73 | 1.15E+01 | 1.45E+01 | 1.58E+01 | 1.64E+01 | 1.70E+01 | 1.58E+01 | 1.54E+01 | 1.78E+01 | 1.49E+01 | 1.35E+01 | 2.14E+01 | 1.49E+01 | 1.46E+01 | 1.65E+01 | 1.22E+01 | 1.42E+01 | 1.80E+01 | 14.85 | x | x | x |
| 156.1494 | 1.73 | 6.12E+00 | 5.80E+00 | 6.86E+00 | 8.31E+00 | 8.07E+00 | 1.14E+01 | 7.38E+00 | 1.02E+01 | 8.67E+00 | 8.48E+00 | 1.00E+01 | 1.22E+01 | 8.94E+00 | 9.31E+00 | 1.19E+01 | 1.13E+01 | 9.19E+00 | 21.3 |  |  | x |
| 200.1215 | 1.73 | 9.25E+01 | 8.86E+01 | 9.17E+01 | 1.04E+02 | 1.10E+02 | 1.14E+02 | 1.05E+02 | 1.17E+02 | 1.18E+02 | 1.04E+02 | 8.23E+01 | 1.20E+02 | 1.13E+02 | 1.13E+02 | 1.19E+02 | 1.12E+02 | 1.16E+02 | 10.97 | x | x | x |
| 271.0602 | 1.73 | 3.14E+01 | 2.84E+01 | 3.14E+01 | 2.27E+01 | 3.02E+01 | 3.03E+01 | 3.21E+01 | 2.84E+01 | 3.08E+01 | 2.47E+01 | 2.04E+01 | 2.76E+01 | 3.31E+01 | 2.49E+01 | 2.95E+01 | 2.94E+01 | 3.26E+01 | 12.54 | x | x | x |
| 340.0640 | 1.73 | 3.07E+01 | 3.12E+01 | 3.31E+01 | 3.53E+01 | 4.16E+01 | 4.63E+01 | 4.01E+01 | 4.29E+01 | 3.77E+01 | 3.52E+01 | 1.71E+00 | 4.61E+01 | 4.34E+01 | 4.23E+01 | 3.84E+01 | 4.91E+01 | 4.63E+01 | 28.66 |  |  | x |
| 160.0760 | 1.74 | 3.31E+01 | 3.05E+01 | 2.94E+01 | 3.41E+01 | 3.19E+01 | 3.81E+01 | 3.78E+01 | 3.49E+01 | 3.98E+01 | 3.43E+01 | 3.83E+01 | 3.93E+01 | 3.49E+01 | 3.79E+01 | 3.62E+01 | 3.90E+01 | 3.31E+01 | 8.95 | x | x | x |
| 291.1540 | 1.74 | 7.77E+01 | 7.15E+01 | 8.23E+01 | 9.08E+01 | 7.87E+01 | 9.09E+01 | 9.10E+01 | 9.15E+01 | 8.75E+01 | 7.87E+01 | 1.40E+02 | 9.17E+01 | 8.34E+01 | 8.45E+01 | 8.50E+01 | 8.65E+01 | 9.12E+01 | 16.57 |  | x | x |
| 303.1500 | 1.74 | 1.05E+01 | 1.13E+01 | 1.00E+01 | 1.31E+01 | 1.31E+01 | 1.22E+01 | 1.46E+01 | 1.47E+01 | 1.03E+01 | 1.03E+01 | 1.77E+01 | 1.01E+01 | 1.18E+01 | 1.28E+01 | 1.53E+01 | 1.52E+01 | 1.33E+01 | 17.49 |  | x | x |
| 353.1347 | 1.74 | 2.14E+02 | 1.87E+02 | 1.96E+02 | 2.11E+02 | 2.02E+02 | 2.24E+02 | 2.16E+02 | 2.06E+02 | 2.16E+02 | 1.92E+02 | 3.80E+01 | 2.27E+02 | 2.12E+02 | 2.25E+02 | 2.09E+02 | 2.10E+02 | 2.28E+02 | 21.71 |  |  | x |
| 343.1698 | 1.74 | 1.07E+01 | 1.11E+01 | 1.26E+01 | 9.11E+00 | 1.16E+01 | 1.16E+01 | 1.59E+01 | 1.25E+01 | 1.07E+01 | 1.02E+01 | 1.43E+00 | 1.04E+01 | 1.46E+01 | 1.30E+01 | 1.19E+01 | 1.48E+01 | 1.46E+01 | 27.76 |  |  | x |
| 356.1373 | 1.74 | 1.18E+01 | 1.16E+01 | 1.18E+01 | 9.00E+00 | 1.09E+01 | 1.22E+01 | 1.04E+01 | 1.12E+01 | 1.04E+01 | 1.34E+01 | 1.64E+01 | 7.89E+00 | 1.11E+01 | 1.04E+01 | 1.23E+01 | 1.30E+01 | 1.38E+01 | 16.67 |  | x | x |
| 220.0934 | 1.75 | 1.56E+01 | 1.78E+01 | 1.54E+01 | 2.05E+01 | 1.76E+01 | 2.05E+01 | 2.17E+01 | 2.49E+01 | 2.17E+01 | 1.75E+01 | 1.89E+01 | 2.04E+01 | 1.62E+01 | 2.00E+01 | 1.93E+01 | 1.95E+01 | 2.53E+01 | 14.48 | x | x | x |
| 267.1227 | 1.75 | 8.45E+01 | 7.42E+01 | 8.42E+01 | 8.04E+01 | 8.19E+01 | 8.83E+01 | 8.93E+01 | 8.72E+01 | 9.57E+01 | 8.40E+01 | 1.05E+02 | 9.11E+01 | 9.46E+01 | 9.29E+01 | 8.13E+01 | 1.01E+02 | 1.02E+02 | 9.37 | x | x | x |
| 357.2502 | 1.75 | 9.05E+02 | 7.91E+02 | 8.68E+02 | 8.78E+02 | 8.94E+02 | 9.59E+02 | 9.31E+02 | 8.67E+02 | 9.62E+02 | 7.95E+02 | 1.96E+02 | 9.26E+02 | 8.90E+02 | 8.53E+02 | 8.63E+02 | 8.37E+02 | 8.97E+02 | 20.57 |  |  | x |
| 312.1301 | 1.75 | 1.46E+01 | 1.52E+01 | 9.63E+00 | 2.10E+01 | 1.43E+01 | 1.23E+01 | 1.14E+01 | 1.23E+01 | 1.58E+01 | 1.43E+01 | 1.73E+01 | 1.94E+01 | 1.17E+01 | 9.04E+00 | 2.51E+01 | 1.46E+01 | 1.49E+01 | 27.45 |  |  | x |
| 369.1294 | 1.75 | 2.85E+01 | 2.29E+01 | 2.91E+01 | 2.23E+01 | 2.42E+01 | 3.11E+01 | 2.94E+01 | 2.38E+01 | 3.18E+01 | 2.21E+01 | 4.38E+00 | 2.58E+01 | 2.35E+01 | 2.80E+01 | 2.18E+01 | 2.30E+01 | 2.04E+01 | 25.63 |  |  | x |
| 369.2465 | 1.75 | 7.71E+01 | 6.47E+01 | 7.70E+01 | 7.52E+01 | 6.37E+01 | 7.58E+01 | 7.63E+01 | 7.82E+01 | 8.32E+01 | 6.79E+01 | 2.34E+01 | 7.34E+01 | 7.57E+01 | 6.19E+01 | 6.58E+01 | 6.01E+01 | 7.35E+01 | 19.54 |  | x | x |
| 134.0606 | 1.76 | 1.96E+01 | 1.65E+01 | 1.60E+01 | 1.73E+01 | 1.61E+01 | 1.55E+01 | 1.63E+01 | 1.93E+01 | 1.73E+01 | 1.86E+01 | 2.48E+01 | 1.70E+01 | 1.95E+01 | 1.84E+01 | 1.70E+01 | 1.89E+01 | 1.68E+01 | 12.3 | x | x | x |
| 224.1186 | 1.76 | 5.67E+01 | 5.14E+01 | 6.32E+01 | 6.83E+01 | 6.96E+01 | 7.17E+01 | 7.46E+01 | 6.88E+01 | 7.78E+01 | 6.19E+01 | 7.39E+01 | 7.59E+01 | 6.79E+01 | 7.38E+01 | 7.23E+01 | 6.61E+01 | 7.21E+01 | 10.18 | x | x | x |
| 276.1181 | 1.76 | 9.87E+01 | 1.00E+02 | 1.07E+02 | 1.07E+02 | 1.05E+02 | 1.21E+02 | 1.22E+02 | 1.21E+02 | 1.19E+02 | 1.06E+02 | 3.84E+01 | 1.21E+02 | 1.30E+02 | 1.28E+02 | 1.25E+02 | 1.26E+02 | 1.35E+02 | 19.53 |  | x | x |
| 274.0963 | 1.76 | 9.48E+01 | 8.35E+01 | 9.99E+01 | 1.26E+02 | 1.31E+02 | 1.47E+02 | 1.51E+02 | 1.41E+02 | 1.58E+02 | 1.33E+02 | 8.92E+01 | 1.40E+02 | 1.55E+02 | 1.63E+02 | 1.30E+02 | 1.56E+02 | 1.62E+02 | 19.7 |  | x | x |
| 312.1080 | 1.76 | 3.43E+01 | 2.77E+01 | 3.28E+01 | 3.35E+01 | 3.15E+01 | 3.30E+01 | 3.61E+01 | 3.57E+01 | 4.00E+01 | 3.41E+01 | 1.33E+01 | 3.62E+01 | 3.92E+01 | 3.51E+01 | 3.60E+01 | 3.72E+01 | 3.71E+01 | 17.82 |  | x | x |
| 391.0271 | 1.76 | 2.51E+01 | 1.98E+01 | 2.13E+01 | 2.53E+01 | 2.44E+01 | 2.19E+01 | 2.13E+01 | 2.40E+01 | 3.73E+01 | 2.64E+01 | 9.19E+00 | 2.87E+01 | 3.05E+01 | 3.24E+01 | 2.37E+01 | 2.63E+01 | 2.47E+01 | 24.02 |  |  | x |
| 453.1047 | 1.76 | 8.23E+01 | 7.01E+01 | 7.81E+01 | 7.36E+01 | 5.98E+01 | 5.89E+01 | 6.37E+01 | 5.91E+01 | 6.55E+01 | 5.92E+01 | 8.08E+01 | 6.06E+01 | 5.07E+01 | 6.45E+01 | 5.12E+01 | 6.29E+01 | 5.79E+01 | 14.57 | x | x | x |
| 332.1343 | 1.77 | 2.33E+03 | 2.07E+03 | 2.23E+03 | 2.40E+03 | 2.37E+03 | 2.60E+03 | 2.49E+03 | 2.42E+03 | 2.59E+03 | 2.37E+03 | 2.96E+03 | 2.53E+03 | 2.41E+03 | 2.40E+03 | 2.48E+03 | 2.42E+03 | 2.53E+03 | 7.53 | x | x | x |
| 218.0818 | 1.77 | 5.08E+01 | 4.66E+01 | 5.42E+01 | 5.39E+01 | 5.15E+01 | 5.21E+01 | 5.95E+01 | 5.24E+01 | 5.30E+01 | 4.63E+01 | 2.58E+01 | 5.86E+01 | 5.53E+01 | 5.29E+01 | 5.04E+01 | 5.79E+01 | 5.57E+01 | 14.72 | x | x | x |
| 256.2130 | 1.77 | 5.02E+01 | 5.06E+01 | 5.23E+01 | 6.25E+01 | 5.96E+01 | 6.66E+01 | 6.11E+01 | 6.61E+01 | 6.17E+01 | 5.08E+01 | 3.35E+01 | 6.63E+01 | 6.55E+01 | 5.72E+01 | 6.87E+01 | 6.04E+01 | 5.92E+01 | 15.03 |  | x | x |
| 412.1250 | 1.77 | 2.78E+02 | 2.51E+02 | 2.74E+02 | 2.78E+02 | 2.56E+02 | 3.01E+02 | 2.84E+02 | 2.77E+02 | 2.93E+02 | 2.48E+02 | 2.97E+01 | 2.86E+02 | 2.71E+02 | 2.68E+02 | 2.58E+02 | 2.66E+02 | 2.79E+02 | 23.47 |  |  | x |
| 245.0767 | 1.78 | 2.76E+01 | 2.50E+01 | 2.96E+01 | 2.75E+01 | 3.00E+01 | 2.77E+01 | 2.85E+01 | 2.84E+01 | 3.01E+01 | 2.68E+01 | 2.93E+01 | 2.49E+01 | 2.63E+01 | 3.01E+01 | 2.28E+01 | 2.98E+01 | 3.18E+01 | 8.24 | x | x | x |
| 277.0526 | 1.78 | 4.84E+01 | 4.51E+01 | 4.81E+01 | 4.80E+01 | 4.45E+01 | 4.87E+01 | 4.87E+01 | 5.09E+01 | 4.93E+01 | 4.27E+01 | 2.78E+01 | 5.39E+01 | 5.01E+01 | 4.88E+01 | 5.74E+01 | 4.94E+01 | 5.87E+01 | 13.81 | x | x | x |
| 286.1147 | 1.78 | 3.32E+01 | 3.26E+01 | 2.66E+01 | 2.90E+01 | 2.45E+01 | 2.43E+01 | 2.44E+01 | 3.76E+01 | 2.49E+01 | 2.20E+01 | 1.14E+01 | 3.23E+01 | 2.42E+01 | 2.42E+01 | 2.78E+01 | 2.82E+01 | 2.83E+01 | 21.41 |  |  | x |
| 418.1578 | 1.78 | 1.50E+03 | 1.32E+03 | 1.43E+03 | 1.39E+03 | 1.37E+03 | 1.44E+03 | 1.40E+03 | 1.36E+03 | 1.37E+03 | 1.25E+03 | 5.44E+02 | 1.38E+03 | 1.32E+03 | 1.35E+03 | 1.27E+03 | 1.31E+03 | 1.37E+03 | 15.84 |  | x | x |
| 260.0893 | 1.79 | 1.93E+03 | 1.80E+03 | 2.01E+03 | 2.31E+03 | 2.39E+03 | 2.63E+03 | 2.60E+03 | 2.48E+03 | 2.74E+03 | 2.56E+03 | 4.04E+03 | 2.60E+03 | 2.69E+03 | 2.82E+03 | 2.46E+03 | 2.75E+03 | 2.85E+03 | 18.99 |  | x | x |
| 283.0426 | 1.79 | 2.59E+01 | 2.50E+01 | 3.21E+01 | 3.35E+01 | 3.80E+01 | 4.10E+01 | 4.22E+01 | 3.99E+01 | 4.07E+01 | 3.97E+01 | 8.51E+00 | 4.55E+01 | 4.61E+01 | 4.33E+01 | 4.49E+01 | 4.63E+01 | 4.98E+01 | 27.14 |  |  | x |
| 301.1398 | 1.79 | 1.16E+01 | 8.34E+00 | 1.30E+01 | 1.48E+01 | 1.46E+01 | 2.05E+01 | 1.47E+01 | 1.47E+01 | 1.56E+01 | 1.09E+01 | 6.17E+00 | 1.40E+01 | 1.49E+01 | 1.43E+01 | 1.50E+01 | 1.48E+01 | 1.54E+01 | 23.02 |  |  | x |
| 335.0953 | 1.79 | 2.24E+02 | 2.05E+02 | 2.11E+02 | 2.27E+02 | 2.32E+02 | 2.64E+02 | 2.30E+02 | 2.17E+02 | 2.41E+02 | 2.08E+02 | 4.01E+01 | 2.30E+02 | 2.33E+02 | 2.21E+02 | 2.38E+02 | 2.37E+02 | 2.34E+02 | 21.98 |  |  | x |
| 114.0664 | 1.80 | 2.19E+02 | 2.03E+02 | 2.18E+02 | 2.28E+02 | 2.42E+02 | 2.62E+02 | 2.66E+02 | 2.59E+02 | 2.98E+02 | 2.60E+02 | 3.53E+02 | 2.85E+02 | 2.84E+02 | 3.08E+02 | 2.69E+02 | 2.97E+02 | 3.08E+02 | 14.49 | x | x | x |
| 206.0456 | 1.80 | 1.50E+01 | 1.38E+01 | 1.84E+01 | 1.29E+01 | 1.82E+01 | 1.50E+01 | 1.54E+01 | 1.81E+01 | 2.12E+01 | 1.76E+01 | 1.47E+01 | 1.89E+01 | 1.65E+01 | 2.02E+01 | 1.78E+01 | 1.47E+01 | 2.26E+01 | 15.72 |  | x | x |
| 382.0776 | 1.80 | 5.24E+01 | 5.61E+01 | 5.61E+01 | 5.73E+01 | 5.49E+01 | 6.59E+01 | 5.83E+01 | 6.32E+01 | 6.13E+01 | 5.80E+01 | 1.50E+01 | 6.85E+01 | 6.48E+01 | 5.66E+01 | 5.68E+01 | 6.57E+01 | 5.83E+01 | 20.56 |  |  | x |
| 168.0806 | 1.81 | 1.06E+01 | 9.38E+00 | 1.00E+01 | 1.15E+01 | 1.09E+01 | 1.41E+01 | 1.18E+01 | 1.03E+01 | 1.29E+01 | 9.42E+00 | 1.77E+01 | 1.04E+01 | 9.17E+00 | 1.04E+01 | 1.22E+01 | 1.06E+01 | 1.32E+01 | 18.65 |  | x | x |
| 183.0803 | 1.81 | 1.28E+01 | 1.26E+01 | 1.11E+01 | 1.41E+01 | 1.22E+01 | 1.44E+01 | 1.54E+01 | 1.44E+01 | 1.23E+01 | 1.46E+01 | 2.61E+01 | 1.12E+01 | 1.65E+01 | 1.52E+01 | 1.31E+01 | 1.43E+01 | 1.76E+01 | 23.64 |  |  | x |
| 202.0833 | 1.81 | 1.05E+01 | 8.51E+00 | 9.24E+00 | 1.15E+01 | 1.07E+01 | 1.06E+01 | 1.06E+01 | 1.02E+01 | 1.32E+01 | 9.64E+00 | 6.94E+00 | 1.13E+01 | 1.30E+01 | 1.10E+01 | 9.98E+00 | 1.25E+01 | 1.37E+01 | 16.05 |  | x | x |
| 206.1526 | 1.81 | 4.99E+00 | 2.62E+00 | 2.81E+00 | 3.98E+00 | 3.68E+00 | 3.76E+00 | 3.89E+00 | 5.28E+00 | 2.76E+00 | 3.82E+00 | 3.73E+00 | 6.41E+00 | 3.96E+00 | 6.17E+00 | 3.16E+00 | 3.95E+00 | 5.19E+00 | 27.2 |  |  | x |
| 251.1076 | 1.81 | 1.19E+01 | 1.14E+01 | 1.23E+01 | 1.79E+01 | 1.52E+01 | 1.30E+01 | 1.48E+01 | 1.60E+01 | 1.64E+01 | 1.50E+01 | 9.93E+00 | 1.74E+01 | 1.41E+01 | 1.77E+01 | 1.71E+01 | 1.80E+01 | 1.44E+01 | 16.72 |  | x | x |
| 288.2041 | 1.81 | 1.20E+02 | 1.22E+02 | 1.27E+02 | 1.37E+02 | 1.27E+02 | 1.43E+02 | 1.32E+02 | 1.39E+02 | 1.31E+02 | 1.24E+02 | 1.90E+02 | 1.36E+02 | 1.29E+02 | 1.32E+02 | 1.32E+02 | 1.25E+02 | 1.32E+02 | 11.67 | x | x | x |
| 303.1533 | 1.81 | 2.18E+01 | 1.79E+01 | 2.59E+01 | 2.27E+01 | 2.38E+01 | 2.73E+01 | 2.62E+01 | 2.57E+01 | 2.26E+01 | 2.26E+01 | 3.53E+01 | 2.59E+01 | 2.61E+01 | 2.60E+01 | 2.66E+01 | 2.82E+01 | 3.15E+01 | 15.27 |  | x | x |
| 322.1880 | 1.81 | 1.41E+01 | 1.97E+01 | 1.62E+01 | 2.18E+01 | 1.38E+01 | 1.97E+01 | 1.24E+01 | 1.72E+01 | 1.41E+01 | 1.33E+01 | 3.01E+00 | 1.58E+01 | 1.03E+01 | 1.84E+01 | 1.66E+01 | 1.73E+01 | 1.94E+01 | 28.43 |  |  | x |
| 367.1507 | 1.81 | 8.25E+02 | 7.49E+02 | 8.18E+02 | 8.26E+02 | 8.59E+02 | 9.56E+02 | 9.00E+02 | 9.22E+02 | 9.61E+02 | 8.54E+02 | 1.32E+03 | 9.40E+02 | 9.17E+02 | 9.16E+02 | 8.68E+02 | 9.18E+02 | 9.01E+02 | 13.32 | x | x | x |
| 367.3157 | 1.81 | 3.12E+00 | 5.49E+00 | 4.62E+00 | 5.08E+00 | 5.21E+00 | 6.07E+00 | 5.51E+00 | 6.48E+00 | 5.58E+00 | 5.16E+00 | 4.65E-01 | 5.24E+00 | 5.93E+00 | 6.25E+00 | 5.14E+00 | 5.90E+00 | 5.90E+00 | 27.74 |  |  | x |
| 109.0763 | 1.82 | 2.82E+02 | 2.60E+02 | 2.92E+02 | 2.73E+02 | 3.01E+02 | 3.28E+02 | 3.18E+02 | 3.03E+02 | 3.65E+02 | 3.06E+02 | 4.57E+02 | 3.17E+02 | 3.12E+02 | 3.58E+02 | 2.65E+02 | 3.17E+02 | 3.45E+02 | 14.68 | x | x | x |
| 323.0743 | 1.82 | 2.31E+01 | 2.08E+01 | 2.35E+01 | 2.92E+01 | 2.67E+01 | 3.25E+01 | 2.66E+01 | 2.83E+01 | 3.13E+01 | 2.73E+01 | 1.55E+01 | 3.05E+01 | 3.09E+01 | 2.69E+01 | 2.95E+01 | 3.20E+01 | 2.86E+01 | 16.26 |  | x | x |
| 349.1240 | 1.82 | 2.10E+02 | 1.78E+02 | 1.99E+02 | 1.81E+02 | 1.73E+02 | 1.86E+02 | 1.81E+02 | 1.64E+02 | 1.80E+02 | 1.69E+02 | 2.75E+02 | 1.87E+02 | 1.81E+02 | 1.83E+02 | 1.75E+02 | 1.80E+02 | 1.92E+02 | 13.32 | x | x | x |
| 171.1492 | 1.83 | 2.52E+01 | 2.00E+01 | 2.58E+01 | 2.26E+01 | 3.16E+01 | 3.16E+01 | 3.01E+01 | 3.47E+01 | 4.47E+01 | 3.14E+01 | 2.69E+01 | 3.20E+01 | 3.37E+01 | 3.59E+01 | 2.26E+01 | 2.75E+01 | 3.03E+01 | 19.91 |  | x | x |
| 238.0498 | 1.83 | 2.49E+01 | 2.43E+01 | 2.78E+01 | 3.08E+01 | 3.31E+01 | 3.53E+01 | 3.54E+01 | 2.97E+01 | 3.52E+01 | 3.26E+01 | 3.12E+01 | 3.13E+01 | 3.66E+01 | 3.39E+01 | 3.34E+01 | 3.49E+01 | 3.68E+01 | 11.66 | x | x | x |
| 246.1809 | 1.83 | 7.67E+01 | 6.46E+01 | 7.74E+01 | 6.95E+01 | 8.03E+01 | 7.79E+01 | 7.63E+01 | 8.10E+01 | 8.89E+01 | 8.05E+01 | 4.72E+01 | 7.58E+01 | 8.48E+01 | 8.55E+01 | 6.24E+01 | 7.68E+01 | 8.31E+01 | 13.34 | x | x | x |
| 262.1296 | 1.83 | 1.50E+02 | 1.61E+02 | 1.67E+02 | 1.70E+02 | 1.48E+02 | 1.79E+02 | 1.68E+02 | 1.75E+02 | 1.76E+02 | 1.76E+02 | 3.96E+02 | 1.79E+02 | 1.97E+02 | 1.86E+02 | 1.94E+02 | 1.98E+02 | 1.91E+02 | 29.27 |  |  | x |
| 292.1136 | 1.83 | 1.46E+02 | 1.33E+02 | 1.35E+02 | 1.75E+02 | 1.75E+02 | 2.04E+02 | 1.95E+02 | 1.87E+02 | 1.98E+02 | 1.81E+02 | 2.89E+02 | 1.86E+02 | 2.00E+02 | 2.01E+02 | 1.88E+02 | 2.05E+02 | 2.06E+02 | 18.51 |  | x | x |
| 210.0972 | 1.84 | 2.49E+01 | 2.55E+01 | 2.59E+01 | 3.22E+01 | 3.41E+01 | 3.93E+01 | 3.75E+01 | 3.97E+01 | 4.29E+01 | 2.95E+01 | 4.88E+01 | 4.11E+01 | 3.93E+01 | 3.82E+01 | 3.70E+01 | 4.17E+01 | 3.95E+01 | 18.46 |  | x | x |
| 223.1926 | 1.84 | 9.96E+00 | 9.86E+00 | 1.08E+01 | 1.08E+01 | 1.36E+01 | 1.39E+01 | 1.41E+01 | 1.63E+01 | 1.68E+01 | 1.29E+01 | 9.41E+00 | 1.29E+01 | 1.60E+01 | 1.43E+01 | 1.26E+01 | 1.57E+01 | 1.46E+01 | 17.88 |  | x | x |
| 335.0956 | 1.84 | 3.62E+02 | 3.04E+02 | 3.29E+02 | 3.25E+02 | 3.31E+02 | 3.62E+02 | 3.63E+02 | 3.54E+02 | 3.93E+02 | 3.42E+02 | 1.97E+02 | 3.51E+02 | 3.65E+02 | 3.63E+02 | 3.26E+02 | 3.53E+02 | 3.68E+02 | 12.53 | x | x | x |
| 377.1668 | 1.84 | 1.23E+01 | 1.19E+01 | 1.62E+01 | 2.04E+01 | 1.25E+01 | 1.40E+01 | 9.31E+00 | 1.26E+01 | 1.06E+01 | 1.17E+01 | 5.79E+00 | 1.62E+01 | 1.19E+01 | 1.82E+01 | 1.82E+01 | 1.36E+01 | 1.38E+01 | 26.48 |  |  | x |
| 490.2638 | 1.84 | 4.64E+01 | 3.56E+01 | 5.16E+01 | 4.45E+01 | 3.27E+01 | 3.24E+01 | 4.15E+01 | 3.57E+01 | 3.65E+01 | 2.41E+01 | 3.52E+00 | 3.71E+01 | 3.71E+01 | 3.68E+01 | 3.75E+01 | 2.85E+01 | 3.42E+01 | 29.63 |  |  | x |
| 154.1341 | 1.86 | 3.14E+01 | 2.69E+01 | 3.50E+01 | 3.41E+01 | 4.11E+01 | 4.01E+01 | 4.22E+01 | 3.91E+01 | 4.68E+01 | 4.22E+01 | 4.25E+01 | 4.41E+01 | 4.06E+01 | 4.58E+01 | 3.86E+01 | 4.66E+01 | 4.64E+01 | 13.98 | x | x | x |
| 236.0786 | 1.86 | 5.74E+00 | 5.66E+00 | 4.47E+00 | 6.74E+00 | 5.49E+00 | 4.81E+00 | 6.19E+00 | 6.90E+00 | 8.17E+00 | 6.25E+00 | 4.35E+00 | 6.44E+00 | 6.33E+00 | 4.76E+00 | 4.57E+00 | 5.35E+00 | 6.51E+00 | 17.75 |  | x | x |
| 244.0136 | 1.86 | 9.78E+00 | 1.01E+01 | 1.06E+01 | 1.20E+01 | 1.06E+01 | 8.95E+00 | 1.09E+01 | 1.00E+01 | 1.10E+01 | 7.55E+00 | 7.57E+00 | 1.01E+01 | 9.48E+00 | 9.91E+00 | 1.11E+01 | 1.20E+01 | 1.12E+01 | 12.51 | x | x | x |
| 305.1274 | 1.86 | 7.64E+02 | 6.95E+02 | 7.42E+02 | 8.06E+02 | 8.74E+02 | 8.78E+02 | 8.56E+02 | 8.18E+02 | 9.23E+02 | 7.85E+02 | 1.24E+03 | 8.59E+02 | 8.41E+02 | 8.29E+02 | 7.52E+02 | 8.33E+02 | 8.74E+02 | 13.93 | x | x | x |
| 422.1065 | 1.86 | 3.81E+01 | 3.20E+01 | 2.70E+01 | 3.21E+01 | 2.75E+01 | 2.49E+01 | 2.78E+01 | 1.90E+01 | 3.52E+01 | 2.49E+01 | 2.36E+01 | 2.58E+01 | 2.76E+01 | 3.44E+01 | 2.44E+01 | 3.06E+01 | 2.31E+01 | 17.76 |  | x | x |
| 178.0714 | 1.87 | 1.36E+01 | 1.59E+01 | 1.55E+01 | 1.85E+01 | 2.28E+01 | 2.30E+01 | 2.22E+01 | 2.35E+01 | 2.81E+01 | 2.51E+01 | 2.72E+01 | 2.59E+01 | 2.75E+01 | 2.35E+01 | 2.31E+01 | 2.53E+01 | 2.90E+01 | 19.98 |  | x | x |
| 236.1140 | 1.87 | 8.29E+00 | 8.01E+00 | 6.97E+00 | 5.23E+00 | 1.01E+01 | 7.99E+00 | 8.29E+00 | 4.37E+00 | 8.76E+00 | 8.31E+00 | 1.28E+01 | 8.02E+00 | 4.60E+00 | 6.47E+00 | 6.56E+00 | 6.93E+00 | 8.78E+00 | 26.41 |  |  | x |
| 274.1871 | 1.87 | 1.45E+01 | 1.22E+01 | 1.29E+01 | 1.56E+01 | 1.66E+01 | 1.77E+01 | 1.44E+01 | 1.40E+01 | 1.26E+01 | 1.32E+01 | 1.12E+01 | 1.69E+01 | 1.71E+01 | 1.30E+01 | 1.52E+01 | 1.75E+01 | 1.71E+01 | 14.02 | x | x | x |
| 302.1347 | 1.87 | 2.63E+00 | 2.88E+00 | 3.48E+00 | 2.86E+00 | 1.27E+00 | 2.16E+00 | 2.86E+00 | 4.50E+00 | 1.89E+00 | 1.68E+00 | 2.60E+00 | 3.26E+00 | 2.03E+00 | 2.10E+00 | 2.99E+00 | 2.99E+00 | 3.44E+00 | 29.24 |  |  | x |
| 158.1278 | 1.89 | 1.05E+01 | 1.04E+01 | 1.41E+01 | 1.28E+01 | 1.20E+01 | 1.48E+01 | 1.52E+01 | 1.43E+01 | 1.60E+01 | 1.48E+01 | 1.48E+01 | 1.46E+01 | 1.46E+01 | 1.60E+01 | 1.20E+01 | 1.50E+01 | 1.48E+01 | 12.52 | x | x | x |
| 194.1040 | 1.89 | 7.59E+01 | 6.35E+01 | 7.37E+01 | 7.30E+01 | 8.87E+01 | 8.98E+01 | 8.94E+01 | 8.45E+01 | 9.48E+01 | 7.31E+01 | 7.29E+01 | 9.09E+01 | 8.75E+01 | 8.78E+01 | 5.65E+01 | 9.23E+01 | 9.29E+01 | 13.83 | x | x | x |
| 214.1085 | 1.89 | 1.23E+02 | 1.11E+02 | 1.25E+02 | 1.29E+02 | 1.42E+02 | 1.51E+02 | 1.41E+02 | 1.36E+02 | 1.55E+02 | 1.26E+02 | 2.30E+02 | 1.41E+02 | 1.39E+02 | 1.48E+02 | 1.35E+02 | 1.42E+02 | 1.47E+02 | 17.69 |  | x | x |
| 234.1009 | 1.89 | 1.88E+01 | 1.23E+01 | 1.88E+01 | 1.61E+01 | 1.79E+01 | 1.79E+01 | 1.87E+01 | 1.76E+01 | 1.82E+01 | 1.46E+01 | 1.08E+01 | 1.82E+01 | 1.40E+01 | 1.67E+01 | 1.58E+01 | 1.93E+01 | 2.20E+01 | 16.28 |  | x | x |
| 276.1908 | 1.89 | 1.56E+01 | 1.52E+01 | 1.70E+01 | 2.07E+01 | 1.92E+01 | 2.16E+01 | 1.80E+01 | 2.04E+01 | 2.23E+01 | 1.43E+01 | 2.67E+01 | 2.38E+01 | 2.19E+01 | 1.84E+01 | 1.35E+01 | 2.31E+01 | 2.24E+01 | 18.82 |  | x | x |
| 351.1757 | 1.89 | 3.54E+01 | 3.33E+01 | 3.18E+01 | 3.07E+01 | 3.23E+01 | 3.97E+01 | 3.98E+01 | 3.59E+01 | 4.16E+01 | 4.27E+01 | 5.85E+01 | 4.05E+01 | 3.87E+01 | 3.80E+01 | 3.31E+01 | 4.53E+01 | 4.05E+01 | 17.11 |  | x | x |
| 377.1655 | 1.89 | 2.13E+01 | 1.86E+01 | 1.63E+01 | 1.49E+01 | 1.73E+01 | 1.17E+01 | 1.37E+01 | 1.76E+01 | 2.12E+01 | 1.05E+01 | 2.09E+00 | 1.55E+01 | 1.92E+01 | 1.89E+01 | 1.38E+01 | 1.40E+01 | 1.41E+01 | 30 |  |  | x |
| 203.1505 | 1.90 | 5.09E+02 | 4.59E+02 | 5.05E+02 | 5.39E+02 | 5.79E+02 | 6.26E+02 | 6.32E+02 | 5.56E+02 | 6.78E+02 | 6.01E+02 | 4.42E+02 | 5.96E+02 | 6.15E+02 | 6.64E+02 | 4.35E+02 | 6.15E+02 | 6.36E+02 | 13.43 | x | x | x |
| 202.1145 | 1.90 | 1.23E+01 | 1.27E+01 | 1.46E+01 | 1.65E+01 | 1.71E+01 | 2.20E+01 | 1.62E+01 | 1.53E+01 | 2.32E+01 | 1.88E+01 | 2.05E+01 | 1.44E+01 | 2.12E+01 | 2.16E+01 | 1.41E+01 | 1.73E+01 | 2.01E+01 | 19.6 |  | x | x |
| 203.2224 | 1.90 | 1.28E+00 | 1.70E+00 | 2.00E+00 | 1.84E+00 | 2.18E+00 | 1.08E+00 | 1.71E+00 | 1.28E+00 | 1.46E+00 | 1.41E+00 | 1.73E+00 | 1.64E+00 | 2.58E+00 | 2.14E+00 | 1.48E+00 | 2.17E+00 | 2.87E+00 | 26.61 |  |  | x |
| 203.2723 | 1.90 | 5.60E+00 | 5.79E+00 | 6.78E+00 | 6.11E+00 | 8.19E+00 | 7.79E+00 | 7.40E+00 | 7.25E+00 | 8.48E+00 | 6.90E+00 | 8.45E+00 | 7.64E+00 | 8.83E+00 | 8.39E+00 | 5.63E+00 | 9.39E+00 | 8.72E+00 | 16.01 |  | x | x |
| 365.1076 | 1.90 | 5.06E+03 | 4.37E+03 | 4.84E+03 | 4.71E+03 | 5.04E+03 | 5.45E+03 | 5.33E+03 | 4.84E+03 | 5.42E+03 | 4.83E+03 | 1.01E+03 | 5.16E+03 | 5.27E+03 | 5.30E+03 | 4.48E+03 | 5.11E+03 | 5.27E+03 | 21.38 |  |  | x |
| 365.2736 | 1.90 | 9.34E+01 | 7.87E+01 | 8.69E+01 | 7.94E+01 | 7.63E+01 | 8.77E+01 | 8.48E+01 | 8.15E+01 | 8.86E+01 | 7.72E+01 | 6.79E+00 | 8.71E+01 | 9.09E+01 | 8.48E+01 | 7.34E+01 | 8.90E+01 | 8.40E+01 | 24.57 |  |  | x |
| 362.0967 | 1.90 | 1.08E+01 | 9.68E+00 | 1.11E+01 | 1.27E+01 | 1.01E+01 | 1.17E+01 | 7.59E+00 | 9.64E+00 | 7.98E+00 | 8.78E+00 | 9.70E+00 | 9.32E+00 | 1.08E+01 | 7.81E+00 | 8.10E+00 | 8.43E+00 | 7.39E+00 | 16.26 |  | x | x |
| 381.3426 | 1.90 | 8.33E+00 | 7.67E+00 | 7.54E+00 | 1.00E+01 | 1.13E+01 | 9.82E+00 | 1.24E+01 | 8.59E+00 | 1.26E+01 | 8.29E+00 | 2.29E+00 | 7.89E+00 | 8.31E+00 | 8.03E+00 | 4.98E+00 | 7.27E+00 | 7.75E+00 | 29.55 |  |  | x |
| 406.1357 | 1.90 | 3.81E+02 | 3.16E+02 | 3.31E+02 | 2.84E+02 | 2.72E+02 | 2.99E+02 | 2.95E+02 | 2.42E+02 | 3.16E+02 | 2.95E+02 | 2.63E+02 | 2.70E+02 | 2.70E+02 | 2.96E+02 | 1.50E+02 | 2.83E+02 | 2.46E+02 | 16.82 |  | x | x |
| 308.0744 | 1.91 | 1.46E+01 | 1.28E+01 | 1.56E+01 | 1.81E+01 | 1.45E+01 | 1.70E+01 | 1.78E+01 | 1.70E+01 | 1.57E+01 | 1.44E+01 | 6.77E+00 | 1.70E+01 | 1.70E+01 | 1.47E+01 | 1.59E+01 | 1.58E+01 | 1.74E+01 | 17.17 |  | x | x |
| 188.0674 | 1.92 | 2.60E+01 | 2.26E+01 | 2.60E+01 | 4.98E+01 | 5.04E+01 | 5.43E+01 | 4.87E+01 | 5.38E+01 | 5.48E+01 | 5.11E+01 | 4.19E+01 | 4.84E+01 | 5.80E+01 | 5.70E+01 | 5.69E+01 | 5.72E+01 | 6.34E+01 | 25.21 |  |  | x |
| 429.1128 | 1.92 | 2.73E+01 | 2.57E+01 | 3.36E+01 | 2.17E+01 | 2.28E+01 | 2.18E+01 | 2.25E+01 | 1.54E+01 | 2.97E+01 | 2.63E+01 | 9.17E+00 | 2.01E+01 | 2.18E+01 | 2.29E+01 | 1.55E+01 | 2.26E+01 | 1.94E+01 | 25.55 |  |  | x |
| 198.9822 | 1.93 | 8.55E+01 | 7.70E+01 | 8.16E+01 | 4.75E+01 | 5.24E+01 | 4.92E+01 | 7.62E+01 | 6.81E+01 | 9.32E+01 | 7.80E+01 | 6.20E+01 | 6.24E+01 | 6.56E+01 | 9.65E+01 | 3.20E+01 | 7.15E+01 | 7.83E+01 | 24.64 |  |  | x |
| 421.1463 | 1.93 | 7.55E+01 | 7.94E+01 | 8.63E+01 | 1.03E+02 | 1.03E+02 | 1.16E+02 | 1.15E+02 | 1.08E+02 | 1.18E+02 | 1.01E+02 | 3.07E+01 | 1.12E+02 | 1.03E+02 | 9.86E+01 | 8.60E+01 | 1.15E+02 | 1.13E+02 | 22.24 |  |  | x |
| 306.1162 | 1.94 | 1.67E+01 | 1.84E+01 | 1.89E+01 | 1.23E+01 | 1.48E+01 | 1.91E+01 | 1.53E+01 | 1.32E+01 | 1.56E+01 | 1.91E+01 | 2.86E+01 | 1.17E+01 | 1.48E+01 | 2.05E+01 | 1.02E+01 | 1.69E+01 | 1.41E+01 | 25.87 |  |  | x |
| 197.0229 | 1.95 | 2.20E+01 | 2.01E+01 | 2.26E+01 | 2.35E+01 | 2.48E+01 | 2.54E+01 | 2.38E+01 | 2.53E+01 | 2.85E+01 | 2.85E+01 | 4.66E+01 | 2.72E+01 | 2.66E+01 | 2.94E+01 | 2.63E+01 | 2.79E+01 | 3.22E+01 | 21.6 |  |  | x |
| 248.0789 | 1.95 | 4.99E+00 | 7.01E+00 | 5.91E+00 | 7.56E+00 | 8.01E+00 | 1.10E+01 | 9.26E+00 | 8.75E+00 | 1.43E+01 | 9.29E+00 | 1.56E+01 | 1.03E+01 | 1.04E+01 | 7.97E+00 | 9.99E+00 | 1.20E+01 | 9.75E+00 | 28.58 |  |  | x |
| 266.1255 | 1.95 | 4.50E+01 | 4.65E+01 | 5.94E+01 | 6.00E+01 | 4.47E+01 | 5.21E+01 | 9.18E+01 | 4.83E+01 | 6.04E+01 | 5.16E+01 | 9.34E+01 | 6.09E+01 | 5.39E+01 | 5.50E+01 | 4.24E+01 | 5.77E+01 | 6.40E+01 | 24.99 |  |  | x |
| 377.1656 | 1.96 | 4.71E+01 | 5.32E+01 | 6.34E+01 | 6.25E+01 | 6.74E+01 | 7.47E+01 | 5.95E+01 | 4.68E+01 | 7.83E+01 | 5.90E+01 | 2.28E+01 | 5.74E+01 | 5.74E+01 | 6.96E+01 | 4.96E+01 | 6.73E+01 | 6.04E+01 | 21.86 |  |  | x |
| 465.2079 | 1.96 | 3.77E+01 | 3.97E+01 | 5.21E+01 | 4.60E+01 | 4.22E+01 | 4.99E+01 | 4.70E+01 | 3.44E+01 | 5.65E+01 | 4.02E+01 | 1.53E+01 | 3.61E+01 | 3.23E+01 | 4.48E+01 | 2.54E+01 | 4.32E+01 | 3.53E+01 | 25.03 |  |  | x |
| 169.0361 | 1.97 | 3.10E+01 | 2.83E+01 | 2.71E+01 | 2.86E+01 | 3.50E+01 | 3.46E+01 | 3.51E+01 | 3.03E+01 | 3.29E+01 | 3.40E+01 | 3.47E+01 | 3.45E+01 | 3.60E+01 | 3.32E+01 | 3.48E+01 | 3.66E+01 | 4.09E+01 | 10.34 | x | x | x |
| 603.1043 | 1.97 | 8.08E+00 | 9.86E+00 | 1.15E+01 | 1.04E+01 | 1.10E+01 | 1.51E+01 | 1.37E+01 | 1.60E+01 | 1.51E+01 | 1.35E+01 | 1.07E+01 | 1.66E+01 | 1.56E+01 | 1.61E+01 | 1.60E+01 | 1.45E+01 | 1.44E+01 | 19.57 |  | x | x |
| 330.0601 | 1.97 | 6.88E+02 | 6.47E+02 | 6.96E+02 | 6.50E+02 | 6.93E+02 | 7.48E+02 | 7.17E+02 | 7.07E+02 | 7.69E+02 | 6.96E+02 | 4.88E+02 | 7.33E+02 | 7.28E+02 | 7.61E+02 | 6.75E+02 | 7.43E+02 | 7.69E+02 | 9.48 | x | x | x |
| 425.2449 | 1.97 | 3.25E+01 | 3.10E+01 | 2.86E+01 | 2.75E+01 | 3.12E+01 | 2.57E+01 | 2.70E+01 | 2.50E+01 | 2.62E+01 | 3.02E+01 | 2.45E+01 | 3.20E+01 | 2.51E+01 | 3.52E+01 | 2.50E+01 | 2.47E+01 | 2.60E+01 | 11.83 | x | x | x |
| 166.0518 | 1.98 | 4.67E+01 | 4.64E+01 | 6.38E+01 | 5.11E+01 | 5.29E+01 | 6.18E+01 | 7.45E+01 | 6.00E+01 | 8.37E+01 | 6.55E+01 | 1.12E+02 | 6.25E+01 | 6.83E+01 | 8.39E+01 | 4.15E+01 | 8.26E+01 | 8.11E+01 | 26.83 |  |  | x |
| 285.1918 | 2.00 | 1.78E+01 | 1.71E+01 | 1.51E+01 | 1.33E+01 | 1.03E+01 | 1.53E+01 | 1.45E+01 | 1.02E+01 | 1.54E+01 | 1.75E+01 | 7.31E+00 | 9.03E+00 | 1.03E+01 | 1.79E+01 | 1.32E+01 | 1.37E+01 | 8.36E+00 | 26.07 |  |  | x |
| 270.0668 | 2.00 | 2.96E+01 | 2.63E+01 | 2.55E+01 | 2.42E+01 | 2.85E+01 | 3.15E+01 | 3.03E+01 | 2.88E+01 | 3.12E+01 | 3.03E+01 | 4.05E+01 | 3.41E+01 | 3.14E+01 | 3.44E+01 | 3.11E+01 | 3.06E+01 | 2.68E+01 | 12.63 | x | x | x |
| 124.0874 | 2.01 | 1.70E+02 | 1.39E+02 | 1.42E+02 | 1.41E+02 | 1.48E+02 | 1.69E+02 | 1.58E+02 | 1.49E+02 | 1.74E+02 | 1.59E+02 | 2.09E+02 | 1.64E+02 | 1.63E+02 | 1.86E+02 | 1.48E+02 | 1.63E+02 | 1.68E+02 | 11 | x | x | x |
| 170.0912 | 2.01 | 9.77E+01 | 8.22E+01 | 8.56E+01 | 8.62E+01 | 8.70E+01 | 9.41E+01 | 9.41E+01 | 7.47E+01 | 9.40E+01 | 9.23E+01 | 1.03E+02 | 7.36E+01 | 7.99E+01 | 9.59E+01 | 7.15E+01 | 8.23E+01 | 8.17E+01 | 10.53 | x | x | x |
| 306.0887 | 2.01 | 1.51E+01 | 1.22E+01 | 2.39E+01 | 1.48E+01 | 2.04E+01 | 2.02E+01 | 1.57E+01 | 1.38E+01 | 2.12E+01 | 1.31E+01 | 3.82E+00 | 1.63E+01 | 1.38E+01 | 1.60E+01 | 1.26E+01 | 1.51E+01 | 1.58E+01 | 28.75 |  |  | x |
| 365.1074 | 2.01 | 5.49E+03 | 4.66E+03 | 5.08E+03 | 4.71E+03 | 4.85E+03 | 5.49E+03 | 5.22E+03 | 5.03E+03 | 5.77E+03 | 5.10E+03 | 3.88E+03 | 5.33E+03 | 5.25E+03 | 5.61E+03 | 4.69E+03 | 5.39E+03 | 5.41E+03 | 8.94 | x | x | x |
| 365.2731 | 2.01 | 8.34E+01 | 7.08E+01 | 7.17E+01 | 6.05E+01 | 6.13E+01 | 7.42E+01 | 6.66E+01 | 6.52E+01 | 7.22E+01 | 6.64E+01 | 3.55E+01 | 6.65E+01 | 7.17E+01 | 7.58E+01 | 6.07E+01 | 7.25E+01 | 7.00E+01 | 15 |  | x | x |
| 362.0967 | 2.01 | 2.34E+01 | 2.44E+01 | 1.56E+01 | 1.74E+01 | 2.28E+01 | 2.37E+01 | 1.77E+01 | 1.61E+01 | 2.05E+01 | 2.41E+01 | 7.96E+00 | 1.69E+01 | 1.74E+01 | 1.57E+01 | 1.04E+01 | 2.24E+01 | 2.32E+01 | 25.95 |  |  | x |
| 495.0189 | 2.01 | 2.24E+02 | 2.10E+02 | 2.47E+02 | 2.06E+02 | 1.97E+02 | 2.23E+02 | 1.90E+02 | 2.08E+02 | 1.90E+02 | 1.58E+02 | 9.67E+01 | 2.04E+02 | 1.78E+02 | 1.37E+02 | 1.56E+02 | 1.61E+02 | 1.73E+02 | 19.72 |  | x | x |
| 164.0722 | 2.02 | 5.93E+00 | 5.05E+00 | 3.88E+00 | 4.84E+00 | 2.88E+00 | 5.12E+00 | 5.81E+00 | 4.17E+00 | 4.58E+00 | 6.23E+00 | 9.20E+00 | 6.70E+00 | 5.97E+00 | 4.40E+00 | 3.14E+00 | 6.36E+00 | 5.98E+00 | 28.33 |  |  | x |
| 279.0939 | 2.02 | 3.81E+03 | 3.36E+03 | 3.78E+03 | 3.62E+03 | 3.50E+03 | 3.66E+03 | 3.61E+03 | 3.39E+03 | 3.60E+03 | 3.01E+03 | 2.03E+03 | 3.45E+03 | 3.27E+03 | 3.18E+03 | 3.16E+03 | 3.19E+03 | 3.35E+03 | 12.18 | x | x | x |
| 479.0444 | 2.02 | 4.37E+01 | 3.21E+01 | 4.68E+01 | 3.32E+01 | 3.11E+01 | 2.95E+01 | 3.10E+01 | 2.66E+01 | 3.62E+01 | 1.93E+01 | 1.51E+01 | 3.66E+01 | 2.68E+01 | 2.32E+01 | 1.82E+01 | 3.09E+01 | 2.96E+01 | 27.81 |  |  | x |
| 479.1683 | 2.02 | 3.51E+02 | 2.37E+02 | 2.49E+02 | 2.00E+02 | 1.76E+02 | 2.17E+02 | 1.87E+02 | 1.64E+02 | 1.95E+02 | 1.75E+02 | 6.32E+01 | 1.91E+02 | 1.64E+02 | 1.97E+02 | 1.51E+02 | 1.72E+02 | 1.74E+02 | 29.78 |  |  | x |
| 302.1355 | 2.03 | 3.48E+01 | 2.86E+01 | 3.20E+01 | 2.78E+01 | 2.63E+01 | 2.45E+01 | 2.01E+01 | 1.76E+01 | 2.52E+01 | 2.07E+01 | 1.33E+01 | 3.28E+01 | 2.89E+01 | 3.56E+01 | 2.88E+01 | 2.69E+01 | 3.21E+01 | 22.84 |  |  | x |
| 284.1356 | 2.03 | 5.88E+01 | 5.76E+01 | 6.45E+01 | 7.32E+01 | 5.88E+01 | 7.06E+01 | 9.97E+01 | 7.21E+01 | 7.22E+01 | 6.39E+01 | 1.28E+02 | 6.74E+01 | 6.13E+01 | 7.12E+01 | 6.17E+01 | 6.04E+01 | 6.56E+01 | 24.98 |  |  | x |
| 379.1341 | 2.03 | 1.64E+01 | 1.19E+01 | 1.92E+01 | 1.31E+01 | 1.70E+01 | 1.76E+01 | 1.52E+01 | 1.47E+01 | 1.91E+01 | 1.75E+01 | 1.38E+01 | 1.46E+01 | 1.45E+01 | 1.65E+01 | 1.11E+01 | 1.88E+01 | 1.86E+01 | 15.76 |  | x | x |
| 184.0725 | 2.04 | 5.68E+01 | 5.67E+01 | 5.63E+01 | 5.96E+01 | 7.04E+01 | 7.49E+01 | 6.19E+01 | 5.32E+01 | 6.36E+01 | 5.88E+01 | 1.06E+02 | 6.22E+01 | 6.41E+01 | 6.76E+01 | 5.54E+01 | 6.63E+01 | 7.17E+01 | 18.7 |  | x | x |
| 182.0908 | 2.04 | 9.39E+00 | 7.64E+00 | 8.39E+00 | 8.52E+00 | 6.96E+00 | 6.73E+00 | 6.46E+00 | 4.20E+00 | 7.38E+00 | 2.91E+00 | 1.01E+01 | 5.04E+00 | 6.65E+00 | 6.22E+00 | 6.39E+00 | 7.11E+00 | 6.26E+00 | 25.82 |  |  | x |
| 258.1107 | 2.04 | 1.58E+03 | 1.42E+03 | 1.52E+03 | 1.59E+03 | 1.68E+03 | 1.91E+03 | 1.83E+03 | 1.66E+03 | 1.81E+03 | 1.75E+03 | 1.48E+03 | 1.76E+03 | 1.76E+03 | 1.92E+03 | 1.65E+03 | 1.81E+03 | 1.89E+03 | 8.87 | x | x | x |
| 258.2488 | 2.04 | 2.37E+01 | 2.15E+01 | 2.21E+01 | 2.18E+01 | 2.37E+01 | 2.81E+01 | 2.56E+01 | 2.15E+01 | 2.46E+01 | 2.50E+01 | 1.05E+01 | 2.60E+01 | 2.44E+01 | 2.89E+01 | 2.19E+01 | 2.82E+01 | 2.77E+01 | 17.8 |  | x | x |
| 252.1356 | 2.05 | 1.27E+01 | 9.06E+00 | 1.27E+01 | 8.26E+00 | 1.45E+01 | 1.17E+01 | 1.02E+01 | 1.14E+01 | 1.27E+01 | 1.06E+01 | 1.64E+01 | 8.85E+00 | 1.18E+01 | 1.29E+01 | 1.31E+01 | 1.04E+01 | 1.17E+01 | 17.66 |  | x | x |
| 258.1900 | 2.05 | 4.81E+00 | 4.60E+00 | 3.80E+00 | 2.33E+00 | 2.86E+00 | 3.78E+00 | 4.27E+00 | 2.14E+00 | 4.41E+00 | 3.36E+00 | 2.35E+00 | 3.62E+00 | 4.40E+00 | 2.56E+00 | 2.55E+00 | 3.36E+00 | 3.47E+00 | 25.17 |  |  | x |
| 292.1072 | 2.05 | 1.22E+02 | 1.13E+02 | 1.31E+02 | 1.62E+02 | 1.98E+02 | 1.88E+02 | 1.79E+02 | 1.55E+02 | 1.79E+02 | 1.71E+02 | 2.13E+02 | 1.66E+02 | 2.08E+02 | 1.92E+02 | 3.04E+02 | 2.09E+02 | 1.96E+02 | 23.91 |  |  | x |
| 346.0550 | 2.05 | 1.80E+01 | 2.11E+01 | 2.21E+01 | 2.14E+01 | 2.61E+01 | 2.51E+01 | 2.33E+01 | 2.04E+01 | 2.98E+01 | 2.38E+01 | 1.80E+01 | 3.02E+01 | 2.92E+01 | 2.62E+01 | 1.85E+01 | 2.21E+01 | 2.48E+01 | 16.6 |  | x | x |
| 170.0923 | 2.07 | 1.12E+02 | 1.03E+02 | 1.19E+02 | 1.30E+02 | 1.35E+02 | 1.49E+02 | 1.44E+02 | 1.29E+02 | 1.31E+02 | 1.30E+02 | 1.49E+02 | 1.32E+02 | 1.35E+02 | 1.34E+02 | 1.29E+02 | 1.41E+02 | 1.44E+02 | 9.3 | x | x | x |
| 175.1193 | 2.07 | 9.07E+01 | 7.86E+01 | 8.59E+01 | 1.21E+02 | 1.27E+02 | 1.32E+02 | 1.15E+02 | 1.17E+02 | 1.10E+02 | 9.93E+01 | 1.44E+02 | 1.08E+02 | 1.12E+02 | 1.40E+02 | 1.69E+02 | 1.20E+02 | 1.33E+02 | 19.14 |  | x | x |
| 379.0853 | 2.08 | 5.29E+01 | 5.24E+01 | 5.19E+01 | 3.78E+01 | 4.10E+01 | 5.07E+01 | 5.33E+01 | 4.66E+01 | 5.22E+01 | 5.02E+01 | 3.40E+01 | 5.03E+01 | 5.80E+01 | 4.92E+01 | 3.88E+01 | 5.54E+01 | 5.85E+01 | 14.48 | x | x | x |
| 439.1444 | 2.10 | 5.37E+01 | 4.10E+01 | 4.31E+01 | 3.10E+01 | 3.04E+01 | 4.15E+01 | 3.39E+01 | 3.87E+01 | 3.36E+01 | 3.77E+01 | 4.09E+01 | 3.86E+01 | 3.56E+01 | 3.57E+01 | 2.60E+01 | 3.60E+01 | 4.48E+01 | 16.92 |  | x | x |
| 168.0770 | 2.12 | 3.14E+01 | 3.09E+01 | 3.55E+01 | 4.14E+01 | 7.93E+01 | 4.90E+01 | 4.47E+01 | 4.63E+01 | 4.88E+01 | 4.33E+01 | 4.59E+01 | 4.42E+01 | 4.74E+01 | 4.86E+01 | 5.45E+01 | 4.68E+01 | 4.89E+01 | 22.89 |  |  | x |
| 290.1456 | 2.12 | 2.02E+01 | 2.28E+01 | 2.14E+01 | 1.83E+01 | 2.43E+01 | 2.45E+01 | 2.35E+01 | 2.91E+01 | 2.51E+01 | 2.29E+01 | 3.19E+01 | 2.83E+01 | 2.80E+01 | 2.65E+01 | 1.94E+01 | 3.16E+01 | 2.75E+01 | 16.17 |  | x | x |
| 527.1605 | 2.13 | 8.48E+02 | 7.20E+02 | 7.62E+02 | 5.53E+02 | 4.99E+02 | 5.82E+02 | 5.67E+02 | 5.34E+02 | 6.50E+02 | 5.65E+02 | 5.42E+02 | 5.95E+02 | 5.73E+02 | 6.07E+02 | 4.39E+02 | 5.88E+02 | 5.80E+02 | 16.38 |  | x | x |
| 288.1157 | 2.14 | 2.11E+01 | 1.91E+01 | 2.21E+01 | 2.50E+01 | 2.78E+01 | 3.30E+01 | 3.36E+01 | 3.25E+01 | 3.37E+01 | 3.01E+01 | 2.41E+01 | 2.75E+01 | 3.04E+01 | 3.50E+01 | 3.14E+01 | 3.31E+01 | 3.61E+01 | 17.91 |  | x | x |
| 194.1063 | 2.18 | 1.08E+01 | 9.26E+00 | 8.39E+00 | 1.18E+01 | 1.38E+01 | 1.58E+01 | 9.00E+00 | 1.41E+01 | 1.54E+01 | 1.15E+01 | 8.34E+00 | 1.54E+01 | 1.34E+01 | 1.61E+01 | 1.07E+01 | 1.38E+01 | 1.40E+01 | 21.44 |  |  | x |
| 277.1399 | 2.19 | 3.29E+00 | 3.15E+00 | 3.46E+00 | 3.37E+00 | 3.99E+00 | 3.42E+00 | 4.30E+00 | 2.95E+00 | 3.85E+00 | 3.25E+00 | 3.74E+00 | 4.08E+00 | 4.02E+00 | 4.36E+00 | 3.59E+00 | 2.23E+00 | 3.87E+00 | 15 | x | x | x |
| 527.1604 | 2.19 | 1.01E+03 | 8.24E+02 | 8.29E+02 | 6.49E+02 | 6.42E+02 | 7.81E+02 | 7.51E+02 | 7.12E+02 | 8.00E+02 | 7.08E+02 | 2.60E+02 | 7.38E+02 | 7.86E+02 | 7.99E+02 | 5.85E+02 | 8.31E+02 | 8.21E+02 | 21.16 |  |  | x |
| 57.9356 | 2.24 | 2.70E+01 | 2.09E+01 | 2.43E+01 | 3.66E+01 | 3.59E+01 | 3.93E+01 | 3.80E+01 | 3.83E+01 | 3.53E+01 | 4.51E+01 | 5.65E+01 | 4.20E+01 | 3.76E+01 | 4.25E+01 | 3.45E+01 | 3.91E+01 | 4.65E+01 | 22.29 |  |  | x |
| 59.9311 | 2.24 | 8.49E+00 | 6.93E+00 | 7.47E+00 | 1.23E+01 | 1.38E+01 | 1.21E+01 | 1.32E+01 | 1.21E+01 | 1.28E+01 | 1.35E+01 | 2.03E+01 | 1.50E+01 | 1.31E+01 | 1.31E+01 | 1.24E+01 | 1.23E+01 | 1.44E+01 | 24.22 |  |  | x |
| 87.9369 | 2.24 | 7.07E+01 | 5.95E+01 | 6.72E+01 | 7.98E+01 | 8.52E+01 | 9.23E+01 | 9.04E+01 | 8.78E+01 | 8.70E+01 | 1.02E+02 | 1.23E+02 | 9.42E+01 | 8.70E+01 | 8.56E+01 | 7.81E+01 | 9.03E+01 | 9.15E+01 | 16.24 |  | x | x |
| 182.9635 | 2.24 | 5.18E+01 | 4.22E+01 | 5.62E+01 | 3.85E+01 | 4.21E+01 | 5.09E+01 | 5.23E+01 | 3.63E+01 | 4.96E+01 | 5.29E+01 | 8.36E+01 | 4.76E+01 | 5.68E+01 | 6.03E+01 | 3.45E+01 | 4.70E+01 | 6.28E+01 | 23 |  |  | x |
| 55.9357 | 2.26 | 1.42E+02 | 1.28E+02 | 1.45E+02 | 1.70E+02 | 1.76E+02 | 1.87E+02 | 1.81E+02 | 1.72E+02 | 1.83E+02 | 1.78E+02 | 2.77E+02 | 1.84E+02 | 1.79E+02 | 1.80E+02 | 1.75E+02 | 1.76E+02 | 1.90E+02 | 17.34 |  | x | x |
| 72.9380 | 2.26 | 6.55E+02 | 5.71E+02 | 6.31E+02 | 6.93E+02 | 7.43E+02 | 7.85E+02 | 7.62E+02 | 7.51E+02 | 7.97E+02 | 7.30E+02 | 1.05E+03 | 7.67E+02 | 7.46E+02 | 7.47E+02 | 7.11E+02 | 7.44E+02 | 7.89E+02 | 13.22 | x | x | x |
| 172.8622 | 2.26 | 2.21E+02 | 1.74E+02 | 1.99E+02 | 1.80E+02 | 1.89E+02 | 1.97E+02 | 1.65E+02 | 1.42E+02 | 1.48E+02 | 1.82E+02 | 1.42E+02 | 1.72E+02 | 1.53E+02 | 1.44E+02 | 1.26E+02 | 1.59E+02 | 1.73E+02 | 14.71 | x | x | x |
| 152.9037 | 2.26 | 1.08E+03 | 8.04E+02 | 7.94E+02 | 1.27E+03 | 1.10E+03 | 8.58E+02 | 1.15E+03 | 1.55E+03 | 1.61E+03 | 7.40E+02 | 1.17E+03 | 1.20E+03 | 8.76E+02 | 9.76E+02 | 1.02E+03 | 1.02E+03 | 6.87E+02 | 24.84 |  |  | x |
| 175.9198 | 2.26 | 2.46E+02 | 1.87E+02 | 1.94E+02 | 3.01E+02 | 2.72E+02 | 2.29E+02 | 2.78E+02 | 3.99E+02 | 4.14E+02 | 2.05E+02 | 2.57E+02 | 3.29E+02 | 2.39E+02 | 2.81E+02 | 2.80E+02 | 2.90E+02 | 1.99E+02 | 24.06 |  |  | x |
| 170.8664 | 2.26 | 9.73E+00 | 7.01E+00 | 8.72E+00 | 9.10E+00 | 8.18E+00 | 9.72E+00 | 6.12E+00 | 5.79E+00 | 6.59E+00 | 8.69E+00 | 6.19E+00 | 7.41E+00 | 4.89E+00 | 6.31E+00 | 4.43E+00 | 4.88E+00 | 8.63E+00 | 23.99 |  |  | x |
| 193.9303 | 2.26 | 7.49E+02 | 5.90E+02 | 6.21E+02 | 9.19E+02 | 8.24E+02 | 7.15E+02 | 9.14E+02 | 1.16E+03 | 1.25E+03 | 6.33E+02 | 5.99E+02 | 9.22E+02 | 7.36E+02 | 8.31E+02 | 7.56E+02 | 8.17E+02 | 6.04E+02 | 23.61 |  |  | x |
| 236.8340 | 2.26 | 7.58E+01 | 6.06E+01 | 6.96E+01 | 6.18E+01 | 5.87E+01 | 6.71E+01 | 5.95E+01 | 4.63E+01 | 5.20E+01 | 6.45E+01 | 3.87E+01 | 5.81E+01 | 5.34E+01 | 4.99E+01 | 3.55E+01 | 5.20E+01 | 6.34E+01 | 18.56 |  | x | x |
| 238.8315 | 2.26 | 1.98E+01 | 1.49E+01 | 1.93E+01 | 1.39E+01 | 1.25E+01 | 1.58E+01 | 1.31E+01 | 8.04E+00 | 9.83E+00 | 1.38E+01 | 1.08E+01 | 1.35E+01 | 1.34E+01 | 1.15E+01 | 6.96E+00 | 1.32E+01 | 1.41E+01 | 25.28 |  |  | x |
| 244.8672 | 2.26 | 5.63E+01 | 4.31E+01 | 5.20E+01 | 4.72E+01 | 4.54E+01 | 5.05E+01 | 4.47E+01 | 3.78E+01 | 3.92E+01 | 4.68E+01 | 2.47E+01 | 4.28E+01 | 3.99E+01 | 3.95E+01 | 3.11E+01 | 3.89E+01 | 4.45E+01 | 17.72 |  | x | x |
| 246.9971 | 2.26 | 6.68E+00 | 4.91E+00 | 5.93E+00 | 5.60E+00 | 4.19E+00 | 4.90E+00 | 4.28E+00 | 3.58E+00 | 3.54E+00 | 5.34E+00 | 2.20E+00 | 3.00E+00 | 4.33E+00 | 4.13E+00 | 2.52E+00 | 4.10E+00 | 4.31E+00 | 27.26 |  |  | x |
| 250.8298 | 2.26 | 4.91E+00 | 4.81E+00 | 4.73E+00 | 4.26E+00 | 3.14E+00 | 2.61E+00 | 3.41E+00 | 2.90E+00 | 3.54E+00 | 3.20E+00 | 4.36E+00 | 3.31E+00 | 2.29E+00 | 3.11E+00 | 3.33E+00 | 2.81E+00 | 3.64E+00 | 22.32 |  |  | x |
| 246.8634 | 2.26 | 8.94E+02 | 7.53E+02 | 8.73E+02 | 8.41E+02 | 8.65E+02 | 9.22E+02 | 7.98E+02 | 6.81E+02 | 7.59E+02 | 8.58E+02 | 6.52E+02 | 7.91E+02 | 7.73E+02 | 7.29E+02 | 5.39E+02 | 7.43E+02 | 8.14E+02 | 12.38 | x | x | x |
| 252.8252 | 2.26 | 1.61E+02 | 1.34E+02 | 1.35E+02 | 1.35E+02 | 1.30E+02 | 1.07E+02 | 1.15E+02 | 1.34E+02 | 1.41E+02 | 1.12E+02 | 1.29E+02 | 1.14E+02 | 9.66E+01 | 1.07E+02 | 7.91E+01 | 1.12E+02 | 1.05E+02 | 16.24 |  | x | x |
| 291.8507 | 2.26 | 8.95E+00 | 8.38E+00 | 8.07E+00 | 9.02E+00 | 9.12E+00 | 6.99E+00 | 8.02E+00 | 7.86E+00 | 6.11E+00 | 9.85E+00 | 9.65E+00 | 7.81E+00 | 6.61E+00 | 8.83E+00 | 5.89E+00 | 8.02E+00 | 8.04E+00 | 14.22 | x | x | x |
| 293.8462 | 2.26 | 2.55E+02 | 2.30E+02 | 2.49E+02 | 2.33E+02 | 2.43E+02 | 2.38E+02 | 2.38E+02 | 2.14E+02 | 2.22E+02 | 2.42E+02 | 2.05E+02 | 2.22E+02 | 2.16E+02 | 2.16E+02 | 1.54E+02 | 2.20E+02 | 2.43E+02 | 10.24 | x | x | x |
| 81.9377 | 2.27 | 5.47E+00 | 4.80E+00 | 4.20E+00 | 3.41E+00 | 5.35E+00 | 4.89E+00 | 5.88E+00 | 6.01E+00 | 6.93E+00 | 5.84E+00 | 1.06E+01 | 6.39E+00 | 5.95E+00 | 5.75E+00 | 5.01E+00 | 5.57E+00 | 6.50E+00 | 25.95 |  |  | x |
| 218.8675 | 2.27 | 1.76E+01 | 1.55E+01 | 2.04E+01 | 1.62E+01 | 1.63E+01 | 2.17E+01 | 1.28E+01 | 1.28E+01 | 1.23E+01 | 1.48E+01 | 1.04E+01 | 1.24E+01 | 1.41E+01 | 1.40E+01 | 1.41E+01 | 1.08E+01 | 1.09E+01 | 21.92 |  |  | x |
| 108.9142 | 2.28 | 1.81E+01 | 1.63E+01 | 1.68E+01 | 1.94E+01 | 1.59E+01 | 1.99E+01 | 1.69E+01 | 1.30E+01 | 1.72E+01 | 1.47E+01 | 1.68E+01 | 1.17E+01 | 1.17E+01 | 1.02E+01 | 1.16E+01 | 1.12E+01 | 1.07E+01 | 21.72 |  |  | x |
| 286.8432 | 2.28 | 4.61E+01 | 3.94E+01 | 4.62E+01 | 3.28E+01 | 3.80E+01 | 4.33E+01 | 3.14E+01 | 2.39E+01 | 3.00E+01 | 2.74E+01 | 2.53E+01 | 2.56E+01 | 3.44E+01 | 2.46E+01 | 1.71E+01 | 2.42E+01 | 2.88E+01 | 26.74 |  |  | x |
| 218.9304 | 2.29 | 6.00E+00 | 5.10E+00 | 3.63E+00 | 4.08E+00 | 2.79E+00 | 3.46E+00 | 3.86E+00 | 3.51E+00 | 4.65E+00 | 5.23E+00 | 4.36E+00 | 3.44E+00 | 4.35E+00 | 1.91E+00 | 2.76E+00 | 4.52E+00 | 3.95E+00 | 25.17 |  |  | x |
| 214.9134 | 2.29 | 1.87E+02 | 1.60E+02 | 1.94E+02 | 1.55E+02 | 1.69E+02 | 2.08E+02 | 1.84E+02 | 1.51E+02 | 1.67E+02 | 1.71E+02 | 2.11E+02 | 1.82E+02 | 1.84E+02 | 1.61E+02 | 1.20E+02 | 1.62E+02 | 1.99E+02 | 13.17 | x | x | x |
| 261.8964 | 2.29 | 3.72E+01 | 3.75E+01 | 3.62E+01 | 3.34E+01 | 3.41E+01 | 3.91E+01 | 4.09E+01 | 3.50E+01 | 3.83E+01 | 3.57E+01 | 3.22E+01 | 3.54E+01 | 3.83E+01 | 3.91E+01 | 2.74E+01 | 3.46E+01 | 4.12E+01 | 9.4 | x | x | x |
| 220.8732 | 2.31 | 3.77E+01 | 3.28E+01 | 3.20E+01 | 2.81E+01 | 2.98E+01 | 2.75E+01 | 3.01E+01 | 2.96E+01 | 3.72E+01 | 2.74E+01 | 4.77E+01 | 2.91E+01 | 3.08E+01 | 2.97E+01 | 1.67E+01 | 2.90E+01 | 3.30E+01 | 20.11 |  |  | x |
| 254.8577 | 2.33 | 2.27E+02 | 1.82E+02 | 2.14E+02 | 1.81E+02 | 1.84E+02 | 2.13E+02 | 1.91E+02 | 1.49E+02 | 1.92E+02 | 1.89E+02 | 1.31E+02 | 2.17E+02 | 1.88E+02 | 1.90E+02 | 1.29E+02 | 1.76E+02 | 1.95E+02 | 14.87 | x | x | x |
| 256.8546 | 2.33 | 1.18E+02 | 9.94E+01 | 1.18E+02 | 8.67E+01 | 1.02E+02 | 1.12E+02 | 1.02E+02 | 7.75E+01 | 9.62E+01 | 1.02E+02 | 8.24E+01 | 1.09E+02 | 1.00E+02 | 9.36E+01 | 6.49E+01 | 9.12E+01 | 1.10E+02 | 14.59 | x | x | x |
| 292.1026 | 2.33 | 6.57E+01 | 5.90E+01 | 6.81E+01 | 6.82E+01 | 6.79E+01 | 7.73E+01 | 7.24E+01 | 7.31E+01 | 8.04E+01 | 6.78E+01 | 7.43E+01 | 7.69E+01 | 8.20E+01 | 7.60E+01 | 7.34E+01 | 8.16E+01 | 7.78E+01 | 8.56 | x | x | x |
| 264.8483 | 2.34 | 6.31E+01 | 5.29E+01 | 5.91E+01 | 3.89E+01 | 4.11E+01 | 5.38E+01 | 4.68E+01 | 3.55E+01 | 5.08E+01 | 4.48E+01 | 2.72E+01 | 6.51E+01 | 4.77E+01 | 4.23E+01 | 2.78E+01 | 4.52E+01 | 4.75E+01 | 23.14 |  |  | x |
| 266.8466 | 2.34 | 5.68E+01 | 4.90E+01 | 5.94E+01 | 3.72E+01 | 3.88E+01 | 5.17E+01 | 4.06E+01 | 3.41E+01 | 4.72E+01 | 4.22E+01 | 5.06E+01 | 6.11E+01 | 4.56E+01 | 4.47E+01 | 2.50E+01 | 3.98E+01 | 4.90E+01 | 20.48 |  |  | x |
| 379.0335 | 2.34 | 1.05E+01 | 8.85E+00 | 9.66E+00 | 1.38E+01 | 1.26E+01 | 1.26E+01 | 8.84E+00 | 1.07E+01 | 9.53E+00 | 9.57E+00 | 2.68E+00 | 1.04E+01 | 6.16E+00 | 8.45E+00 | 7.96E+00 | 1.44E+01 | 1.05E+01 | 28.58 |  |  | x |
| 130.9584 | 2.35 | 1.23E+02 | 1.15E+02 | 1.19E+02 | 1.14E+02 | 1.28E+02 | 1.41E+02 | 1.45E+02 | 1.36E+02 | 1.59E+02 | 1.37E+02 | 2.10E+02 | 1.62E+02 | 1.47E+02 | 1.52E+02 | 1.14E+02 | 1.45E+02 | 1.54E+02 | 16.81 |  | x | x |
| 132.9555 | 2.35 | 1.07E+02 | 9.54E+01 | 1.04E+02 | 1.05E+02 | 1.17E+02 | 1.24E+02 | 1.26E+02 | 1.12E+02 | 1.39E+02 | 1.11E+02 | 1.82E+02 | 1.36E+02 | 1.31E+02 | 1.34E+02 | 1.12E+02 | 1.26E+02 | 1.32E+02 | 16.15 |  | x | x |
| 205.9205 | 2.35 | 8.44E+01 | 7.14E+01 | 8.64E+01 | 7.29E+01 | 6.96E+01 | 7.48E+01 | 7.02E+01 | 8.89E+01 | 8.15E+01 | 5.14E+01 | 6.83E+01 | 9.98E+01 | 6.07E+01 | 6.15E+01 | 1.07E+02 | 4.71E+01 | 5.45E+01 | 22.38 |  |  | x |
| 262.8510 | 2.35 | 4.76E+01 | 4.04E+01 | 4.59E+01 | 2.85E+01 | 3.18E+01 | 3.15E+01 | 3.96E+01 | 2.56E+01 | 3.45E+01 | 3.22E+01 | 2.79E+01 | 4.32E+01 | 3.53E+01 | 3.63E+01 | 2.04E+01 | 3.23E+01 | 3.65E+01 | 20.76 |  |  | x |
| 301.8409 | 2.35 | 1.27E+01 | 1.23E+01 | 1.27E+01 | 8.11E+00 | 7.58E+00 | 8.00E+00 | 1.20E+01 | 9.21E+00 | 1.55E+01 | 9.85E+00 | 8.56E+00 | 1.33E+01 | 1.19E+01 | 1.25E+01 | 3.57E+00 | 1.17E+01 | 1.24E+01 | 26.91 |  |  | x |
| 98.8988 | 2.36 | 2.67E+01 | 3.08E+01 | 3.10E+01 | 2.24E+01 | 2.35E+01 | 3.64E+01 | 3.01E+01 | 3.08E+01 | 3.31E+01 | 2.89E+01 | 5.63E+01 | 3.92E+01 | 3.11E+01 | 3.04E+01 | 3.74E+01 | 2.86E+01 | 3.43E+01 | 23.38 |  |  | x |
| 100.8947 | 2.36 | 1.71E+01 | 1.58E+01 | 1.70E+01 | 1.51E+01 | 1.44E+01 | 1.65E+01 | 1.29E+01 | 1.26E+01 | 1.92E+01 | 1.47E+01 | 2.89E+01 | 2.00E+01 | 1.59E+01 | 1.52E+01 | 1.31E+01 | 1.38E+01 | 1.63E+01 | 23.3 |  |  | x |
| 139.9243 | 2.36 | 7.05E+01 | 6.64E+01 | 7.64E+01 | 6.35E+01 | 6.95E+01 | 8.28E+01 | 8.27E+01 | 7.23E+01 | 1.01E+02 | 7.30E+01 | 1.16E+02 | 1.01E+02 | 9.11E+01 | 9.73E+01 | 7.50E+01 | 9.97E+01 | 9.64E+01 | 18.18 |  | x | x |
| 141.9215 | 2.36 | 3.24E+01 | 2.51E+01 | 3.21E+01 | 2.98E+01 | 3.17E+01 | 3.63E+01 | 3.70E+01 | 3.16E+01 | 4.34E+01 | 3.30E+01 | 4.71E+01 | 4.31E+01 | 3.89E+01 | 4.21E+01 | 2.98E+01 | 4.03E+01 | 4.13E+01 | 16.8 |  | x | x |
| 303.8385 | 2.36 | 1.36E+01 | 1.33E+01 | 1.01E+01 | 9.78E+00 | 8.11E+00 | 1.08E+01 | 1.25E+01 | 8.29E+00 | 1.10E+01 | 8.27E+00 | 1.28E+01 | 1.15E+01 | 7.93E+00 | 9.20E+00 | 3.27E+00 | 6.32E+00 | 9.43E+00 | 27.39 |  |  | x |
| 379.0333 | 2.40 | 4.28E+01 | 4.11E+01 | 4.55E+01 | 3.96E+01 | 3.25E+01 | 4.03E+01 | 2.86E+01 | 2.89E+01 | 2.97E+01 | 2.51E+01 | 3.16E+01 | 2.83E+01 | 2.67E+01 | 2.33E+01 | 3.36E+01 | 2.50E+01 | 2.08E+01 | 23.14 |  |  | x |
| 439.0548 | 2.40 | 7.09E+01 | 6.38E+01 | 6.48E+01 | 6.33E+01 | 5.61E+01 | 6.25E+01 | 3.94E+01 | 4.76E+01 | 4.50E+01 | 3.88E+01 | 4.23E+01 | 4.52E+01 | 4.65E+01 | 3.64E+01 | 4.62E+01 | 3.76E+01 | 4.30E+01 | 22.27 |  |  | x |
| 705.1872 | 2.40 | 4.67E+01 | 4.05E+01 | 4.43E+01 | 3.14E+01 | 2.30E+01 | 2.31E+01 | 2.30E+01 | 2.81E+01 | 3.07E+01 | 2.99E+01 | 2.90E+01 | 2.81E+01 | 3.28E+01 | 3.05E+01 | 9.47E+00 | 2.74E+01 | 3.22E+01 | 28.73 |  |  | x |
| 220.8774 | 2.42 | 8.23E+01 | 7.41E+01 | 7.50E+01 | 6.58E+01 | 6.59E+01 | 6.12E+01 | 8.19E+01 | 5.79E+01 | 7.52E+01 | 6.63E+01 | 9.63E+01 | 5.85E+01 | 6.03E+01 | 7.18E+01 | 3.18E+01 | 6.44E+01 | 6.54E+01 | 20.15 |  |  | x |
| 230.8906 | 2.42 | 8.82E+01 | 7.37E+01 | 8.42E+01 | 7.83E+01 | 8.56E+01 | 8.75E+01 | 8.92E+01 | 6.27E+01 | 8.35E+01 | 8.58E+01 | 8.84E+01 | 7.48E+01 | 8.39E+01 | 7.81E+01 | 4.36E+01 | 7.43E+01 | 7.72E+01 | 14.54 | x | x | x |
| 240.8810 | 2.42 | 2.73E+00 | 2.87E+00 | 4.28E+00 | 3.61E+00 | 3.49E+00 | 4.07E+00 | 3.50E+00 | 3.81E+00 | 3.28E+00 | 3.49E+00 | 3.60E+00 | 7.04E+00 | 4.94E+00 | 2.90E+00 | 3.24E+00 | 2.43E+00 | 2.93E+00 | 29.13 |  |  | x |
| 487.2163 | 2.43 | 1.83E+01 | 1.30E+01 | 1.90E+01 | 1.52E+01 | 1.16E+01 | 1.39E+01 | 1.41E+01 | 1.57E+01 | 1.55E+01 | 1.08E+01 | 8.99E+00 | 1.74E+01 | 1.46E+01 | 1.13E+01 | 7.17E+00 | 1.48E+01 | 1.22E+01 | 23.08 |  |  | x |
| 504.1953 | 2.50 | 2.17E+01 | 1.32E+01 | 1.80E+01 | 1.43E+01 | 1.24E+01 | 1.14E+01 | 1.59E+01 | 1.28E+01 | 1.27E+01 | 1.39E+01 | 1.41E+01 | 1.23E+01 | 1.79E+01 | 1.75E+01 | 1.26E+01 | 1.31E+01 | 1.67E+01 | 18.75 |  | x | x |
| 434.1200 | 2.52 | 4.90E+01 | 3.40E+01 | 3.90E+01 | 2.96E+01 | 3.22E+01 | 3.12E+01 | 3.02E+01 | 2.65E+01 | 3.10E+01 | 3.25E+01 | 3.86E+01 | 2.88E+01 | 3.06E+01 | 3.61E+01 | 1.52E+01 | 3.36E+01 | 3.90E+01 | 21.3 |  |  | x |
| 67.9351 | 2.71 | 5.89E+00 | 4.39E+00 | 5.20E+00 | 4.10E+00 | 7.15E+00 | 5.91E+00 | 6.53E+00 | 5.81E+00 | 8.42E+00 | 6.81E+00 | 9.10E+00 | 6.14E+00 | 7.56E+00 | 7.34E+00 | 5.73E+00 | 6.95E+00 | 7.83E+00 | 20.48 |  |  | x |
| 206.9175 | 2.76 | 1.95E+02 | 1.70E+02 | 2.04E+02 | 1.33E+02 | 1.46E+02 | 1.61E+02 | 1.72E+02 | 1.60E+02 | 1.93E+02 | 1.83E+02 | 1.90E+02 | 1.63E+02 | 1.72E+02 | 1.87E+02 | 1.33E+02 | 1.67E+02 | 1.78E+02 | 11.98 | x | x | x |
| 103.9559 | 2.94 | 1.55E+01 | 1.04E+01 | 1.39E+01 | 1.43E+01 | 1.64E+01 | 1.12E+01 | 1.62E+01 | 1.67E+01 | 2.01E+01 | 2.01E+01 | 2.49E+01 | 2.25E+01 | 2.03E+01 | 1.82E+01 | 1.88E+01 | 2.20E+01 | 2.38E+01 | 23.26 |  |  | x |
| 149.0236 | 3.01 | 6.08E+00 | 6.30E+00 | 5.75E+00 | 4.62E+00 | 4.98E+00 | 8.67E+00 | 6.34E+00 | 4.55E+00 | 6.73E+00 | 7.29E+00 | 9.71E+00 | 1.01E+01 | 5.35E+00 | 8.51E+00 | 5.09E+00 | 7.21E+00 | 4.87E+00 | 26.66 |  |  | x |
| 279.0942 | 3.01 | 1.74E+03 | 1.56E+03 | 1.73E+03 | 1.75E+03 | 1.69E+03 | 1.74E+03 | 1.69E+03 | 1.63E+03 | 1.70E+03 | 1.43E+03 | 1.34E+03 | 1.74E+03 | 1.56E+03 | 1.54E+03 | 1.50E+03 | 1.48E+03 | 1.53E+03 | 7.87 | x | x | x |
| 429.2415 | 3.01 | 1.45E+02 | 1.44E+02 | 1.57E+02 | 1.41E+02 | 1.45E+02 | 1.59E+02 | 1.23E+02 | 1.31E+02 | 1.52E+02 | 1.16E+02 | 6.47E+01 | 1.36E+02 | 1.18E+02 | 1.12E+02 | 7.19E+01 | 8.70E+01 | 8.22E+01 | 24.48 |  |  | x |
| 550.6291 | 3.01 | 1.03E+03 | 1.34E+03 | 1.79E+03 | 2.11E+03 | 1.98E+03 | 1.97E+03 | 1.87E+03 | 1.70E+03 | 1.88E+03 | 1.59E+03 | 7.00E+02 | 1.65E+03 | 1.61E+03 | 1.55E+03 | 1.41E+03 | 1.45E+03 | 1.44E+03 | 22.36 |  |  | x |
| 270.2790 | 3.12 | 5.95E+02 | 5.30E+02 | 5.81E+02 | 5.66E+02 | 5.55E+02 | 5.20E+02 | 4.80E+02 | 4.41E+02 | 4.60E+02 | 3.83E+02 | 3.73E+02 | 3.62E+02 | 3.50E+02 | 3.19E+02 | 2.93E+02 | 2.86E+02 | 2.72E+02 | 25.71 |  |  | x |
| 224.8605 | 3.24 | 1.86E+01 | 1.83E+01 | 1.62E+01 | 1.40E+01 | 1.78E+01 | 1.18E+01 | 1.20E+01 | 1.48E+01 | 1.66E+01 | 1.33E+01 | 1.81E+01 | 1.69E+01 | 1.13E+01 | 7.65E+00 | 1.14E+01 | 1.60E+01 | 1.32E+01 | 21.22 |  |  | x |
| 226.8587 | 3.24 | 1.93E+01 | 1.46E+01 | 1.89E+01 | 1.66E+01 | 1.35E+01 | 1.21E+01 | 1.30E+01 | 1.37E+01 | 1.24E+01 | 1.57E+01 | 1.78E+01 | 1.48E+01 | 1.41E+01 | 1.14E+01 | 1.10E+01 | 1.36E+01 | 9.14E+00 | 19.5 |  | x | x |
| 335.1260 | 3.27 | 5.85E+02 | 5.16E+02 | 5.93E+02 | 5.58E+02 | 5.63E+02 | 6.44E+02 | 5.73E+02 | 5.30E+02 | 6.50E+02 | 5.70E+02 | 5.09E+02 | 5.77E+02 | 5.71E+02 | 5.99E+02 | 4.58E+02 | 5.59E+02 | 5.48E+02 | 8.23 | x | x | x |
| 301.1409 | 3.28 | 9.91E+01 | 8.29E+01 | 1.12E+02 | 9.44E+01 | 1.05E+02 | 1.10E+02 | 1.01E+02 | 8.54E+01 | 1.09E+02 | 9.67E+01 | 9.13E+01 | 9.09E+01 | 1.02E+02 | 1.06E+02 | 8.80E+01 | 1.07E+02 | 1.07E+02 | 9.22 | x | x | x |
| 121.0883 | 3.29 | 6.21E+01 | 6.02E+01 | 6.87E+01 | 6.81E+01 | 6.91E+01 | 7.15E+01 | 6.88E+01 | 7.10E+01 | 7.35E+01 | 6.72E+01 | 1.06E+02 | 7.43E+01 | 7.31E+01 | 7.33E+01 | 6.21E+01 | 7.35E+01 | 7.17E+01 | 13.77 | x | x | x |
| 301.0754 | 3.29 | 6.96E+01 | 6.50E+01 | 7.42E+01 | 7.15E+01 | 6.75E+01 | 6.24E+01 | 6.52E+01 | 5.72E+01 | 6.77E+01 | 5.34E+01 | 6.04E+01 | 6.07E+01 | 4.99E+01 | 6.16E+01 | 4.37E+01 | 5.91E+01 | 5.71E+01 | 12.78 | x | x | x |
| 413.2670 | 3.29 | 1.65E+03 | 1.44E+03 | 1.64E+03 | 1.31E+03 | 1.31E+03 | 1.39E+03 | 1.24E+03 | 1.09E+03 | 1.31E+03 | 1.11E+03 | 8.88E+02 | 1.14E+03 | 1.11E+03 | 1.13E+03 | 7.68E+02 | 9.59E+02 | 9.26E+02 | 20.72 |  |  | x |
| 249.0483 | 3.30 | 2.82E+01 | 2.21E+01 | 2.68E+01 | 1.74E+01 | 1.55E+01 | 1.64E+01 | 1.62E+01 | 1.69E+01 | 2.10E+01 | 1.58E+01 | 1.95E+01 | 1.65E+01 | 2.05E+01 | 1.47E+01 | 1.13E+01 | 1.26E+01 | 2.02E+01 | 24.58 |  |  | x |
| 102.8920 | 3.31 | 7.58E+01 | 7.42E+01 | 7.68E+01 | 8.14E+01 | 8.61E+01 | 7.69E+01 | 8.67E+01 | 8.46E+01 | 9.56E+01 | 8.55E+01 | 1.45E+02 | 9.71E+01 | 9.22E+01 | 8.48E+01 | 8.30E+01 | 7.43E+01 | 8.57E+01 | 18.76 |  | x | x |
| 273.1670 | 3.31 | 9.68E+00 | 8.86E+00 | 9.31E+00 | 1.11E+01 | 1.05E+01 | 1.06E+01 | 1.22E+01 | 1.19E+01 | 9.10E+00 | 9.96E+00 | 1.35E+01 | 1.30E+01 | 7.37E+00 | 1.26E+01 | 9.25E+00 | 9.29E+00 | 9.85E+00 | 16.08 |  | x | x |
| 178.1591 | 3.32 | 2.79E+00 | 3.66E+00 | 4.81E+00 | 4.47E+00 | 2.57E+00 | 3.15E+00 | 5.57E+00 | 6.01E+00 | 4.42E+00 | 5.05E+00 | 4.35E+00 | 4.59E+00 | 3.96E+00 | 4.08E+00 | 4.58E+00 | 4.06E+00 | 6.03E+00 | 22.72 |  |  | x |
|  |  |  |  |  |  |  |  |  |  |  |  |  |  |  |  |  |  |  | **Total ions** | 211 | 526 | 955 |
|  |  |  |  |  |  |  |  |  |  |  |  |  |  |  |  |  |  |  | **% of ions** | 22 | 55 |  |
